# Supplementary material for: Small molecule disruption of RARα/NCoR1 interaction inhibits chaperone-mediated autophagy in cancer
Source: EMBO Mol Med. 2025 Jun 9;17(7):1716–55. doi: 10.1038/s44321-025-00254-y (PMC12254369; doi:10.1038/s44321-025-00254-y)
Supplement: Supplementary file 1 — Appendix [file 44321_2025_254_MOESM1_ESM.pdf]

Appendix

Small Molecule Disruption of RARα/NCoR1 Interaction Inhibits Chaperone-Mediated Autophagy in Cancer

Mericka McCabe[1,2,3,4], Rajanya Bhattacharyya[1,3,4], Rebecca Sereda[2,3,4], Olaya Santiago-Fernández[2,3,4], Rabia R. Khawaja[2,3,4], Antonio Diaz[2,3,4], Kristen Lindenau[2,3,4], Deniz Gulfem Ozturk[1,3,4], Thomas P. Garner[1,3,4], Simone Sidoli[1,4], Ana Maria Cuervo[2,3,4,5]*, Evripidis Gavathiotis[1,3,4,5,6]*

Appendix Figure S1 .... **Page 2**
Appendix Figure S2 .... **Page 3**
Appendix Figure S3 .... **Page 4**
Appendix Figure S4 .... **Page 5**
Appendix Figure S5 .... **Page 6**
Appendix Figure S6 .... **Page 7**
Appendix Figure S7 .... **Page 8**
Appendix Figure S8 .... **Page 9**
Appendix Figure S9 .... **Page 10**
Appendix Figure S10 .... **Page 11**
Appendix Figure S11 .... **Page 12**
Appendix Figure S12 .... **Page 13**
Appendix Figure S13 .... **Page 14**
Appendix Note S1 .... **Page 15**
Appendix Table S1 .... **Page 16**
Appendix Table S2 .... **Page 17**
Appendix Table S3 .... **Page 20**
Appendix Methods .... **Page 21**
  Chemical Synthesis of CIM7 .... **Page 21**
  Chemical Synthesis of Biotin-CIM7 .... **Page 23**
  PolyA mRNA Sequencing .... **Page 26**

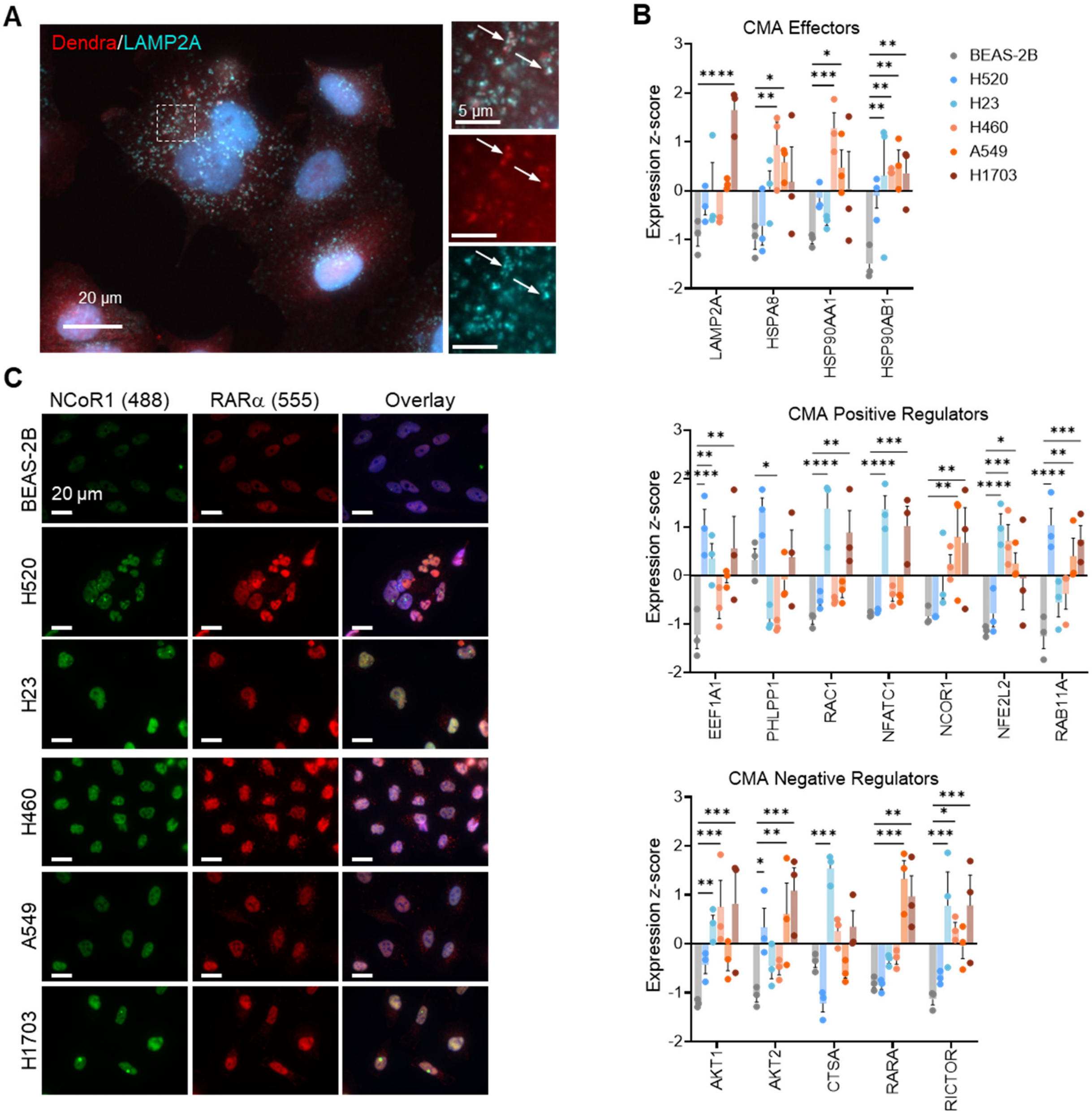

Appendix Figure S1 - Validation of the Dendra reporter, expansion of CMA score calculations in cell lines and immunofluorescence imaging for NCoR1 and RARα.
(**A**) Colocalization of LAMP2A with the expressed Dendra reporter in A549 cells. Full field overlay (left) and higher magnification insets for each channel and the overlay (right). White arrows illustrate areas of colocalization. LAMP2A is in cyan and Dendra in red. Nuclei are highlighted in with DAPI in overlay. (**B**) Expression (as z-score) of CMA effectors, positive regulators, and negative regulators in the indicated cell lines. n = 3 independent experiments. Data is shown as mean + SEM. Two-way ANOVA followed by Bonferroni's multiple comparisons post-hoc test was used. *p≤0.05, **p≤0.01, ***p≤0.001, ****p≤0.0001. (**C**) Full field images of the insets shown in main Fig. 1c of immunofluorescence staining for NCoR1 and RARα in the indicated cell lines. Images of individual and merged (overlay) channels are shown. Nuclei are highlighted with DAPI in overlay.

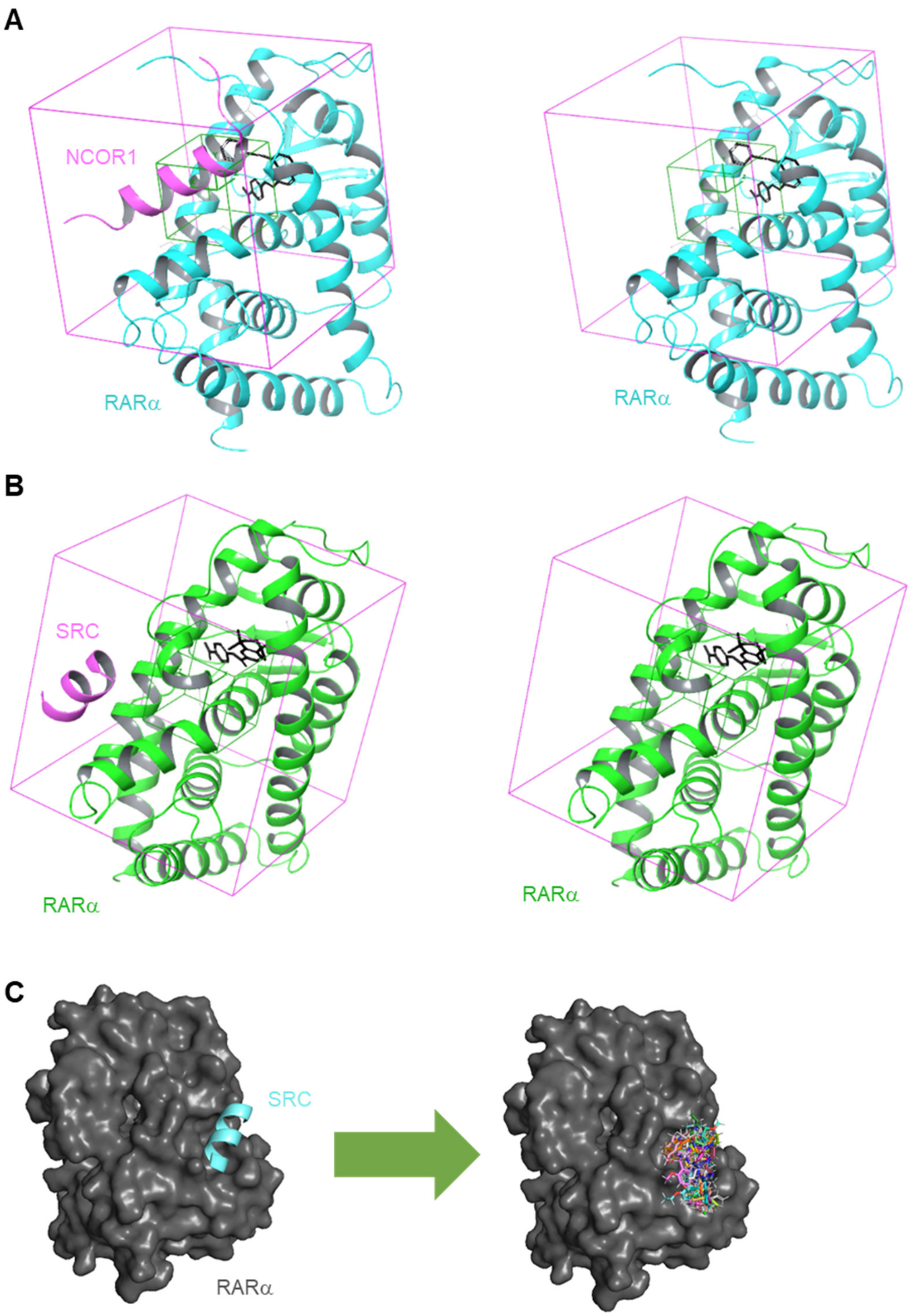

Appendix Figure S2 – Setup for *in silico* glide docking screen.

(**A**) The binding box for the inactive (NCoR1-bound) RARα conformation marked in thin pink lines. Left shows NCoR1 in pink overlayed into the binding box of RARα while right shows the actual screened structure. (**B**) The binding box for the active (SRC-bound) RARα conformation as in A. (**C**) A few selected compounds for screening bound into the same site of SRC

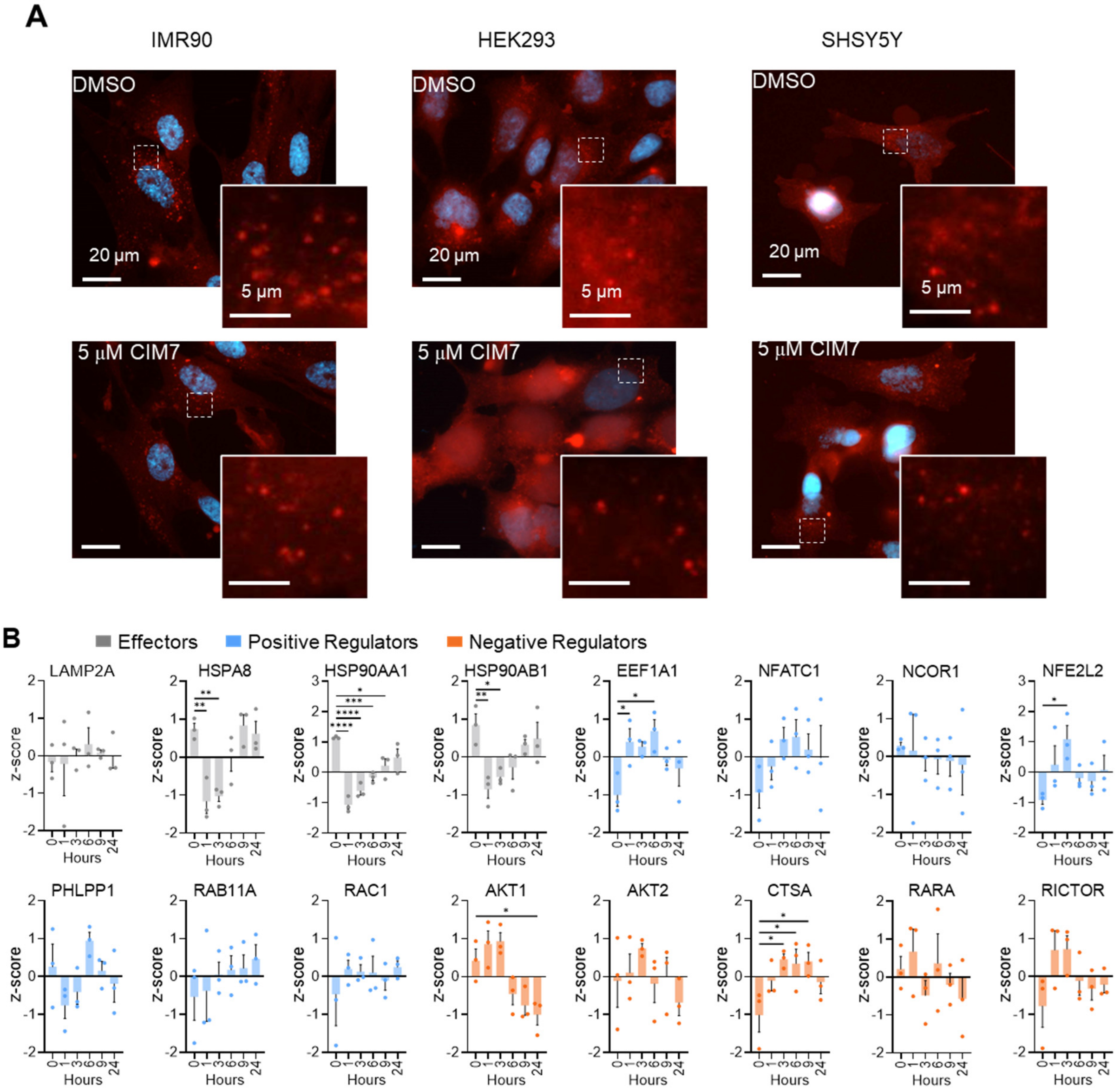

Appendix Figure S3 – CIM7 effect in healthy cells and expansion on CMA score calculations.

(**A**) Representative fluorescence images of IMR90, HEK293, and SHSY5Y cells expressing KFERQ-PS-Dendra and treated with 5 µM CIM7 or equal volume DMSO for 24 hours. Quantification is in Fig. 2F. (**B**) Transcriptional expression (as z-score) of CMA-related genes in A549 cells treated with 5 µM CIM7 for 0, 1, 3, 6, 9, or 24 hours. CMA effectors are in grey, positive regulators in blue, and negative regulators in orange. n = 3 independent experiments.

Data information: All values are mean + SEM with individual data points to represent individual experiments. Ordinary one-way ANOVA followed by Bonferroni's multiple comparisons post-hoc test was used. *p≤0.05, **p≤0.01, ***p≤0.001, ****p≤0.0001.

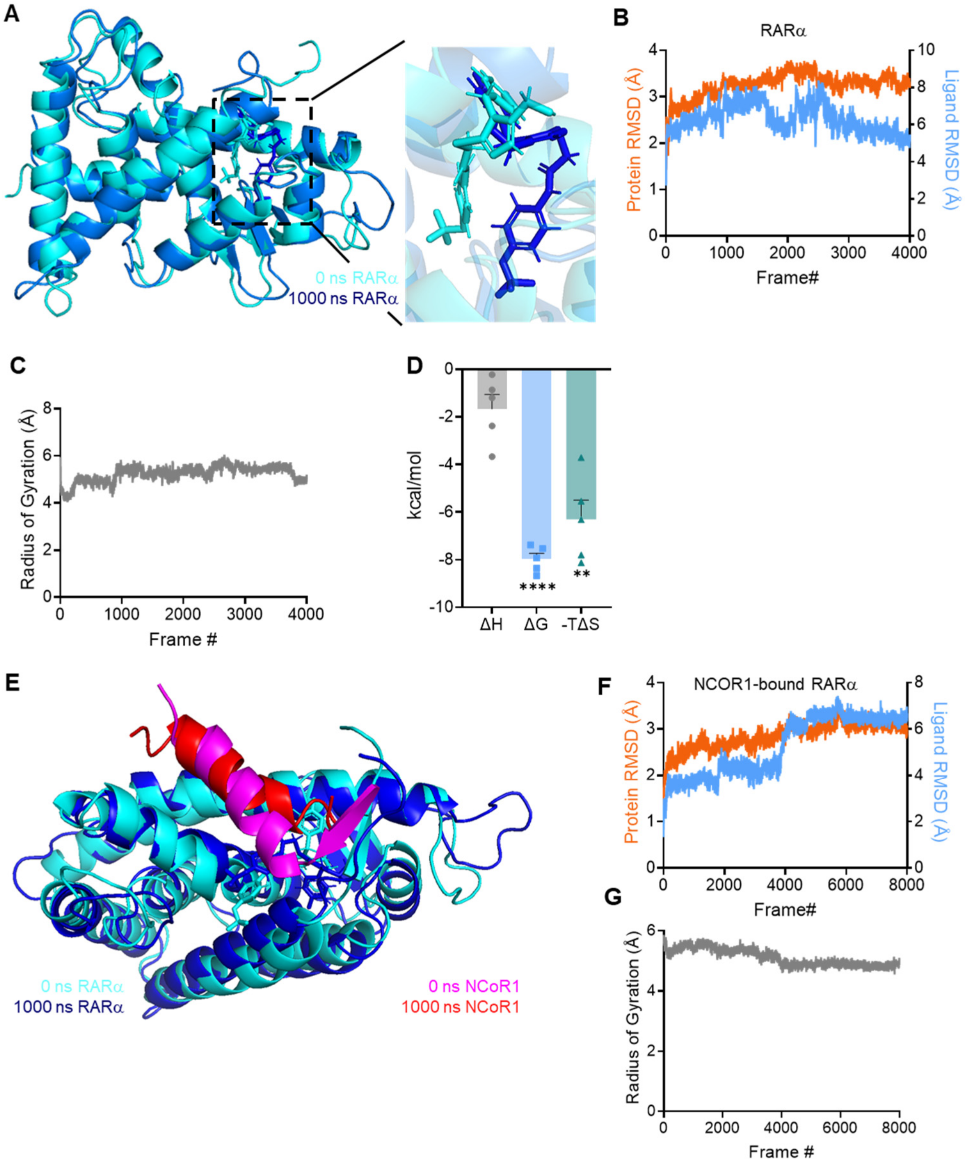

Appendix Figure S4 – Molecular dynamics simulations and isothermal titration calorimetry. (**A-C**) Representative molecular dynamics simulation of RARα in the presence of CIM7 for 1000 ns, with structural changes illustrated by the overlay of the structures at 0 (cyan) and 1000 (blue) ns (**A**), protein (orange) and ligand (blue) root mean square deviation (RMSD) over time (**B**), and radius of gyration over time (**C**). (**D**) Changes in enthalpy (ΔH), free energy (ΔG), and entropy(-TΔS), upon the binding of CIM7 to recombinant RARα, determined by isothermal titration calorimetry. n = 5 independent experiments. Values are mean + SEM. One sample t-test was used. **p≤0.01, ****p≤0.0001. (**E-G**) Representative molecular dynamics simulation of NCOR1-bound RARα in the presence of CIM7 for 1000 ns, with structural changes illustrated by the overlay of the structures at 0 (RARα in cyan and NCoR1 in magenta) and 1000 (RARα in blue and NCoR1 in red) ns (**E**) protein (orange) and ligand (blue) RMSDs over a 2000 ns simulation (**F**), and radius of gyration over time (**G**).

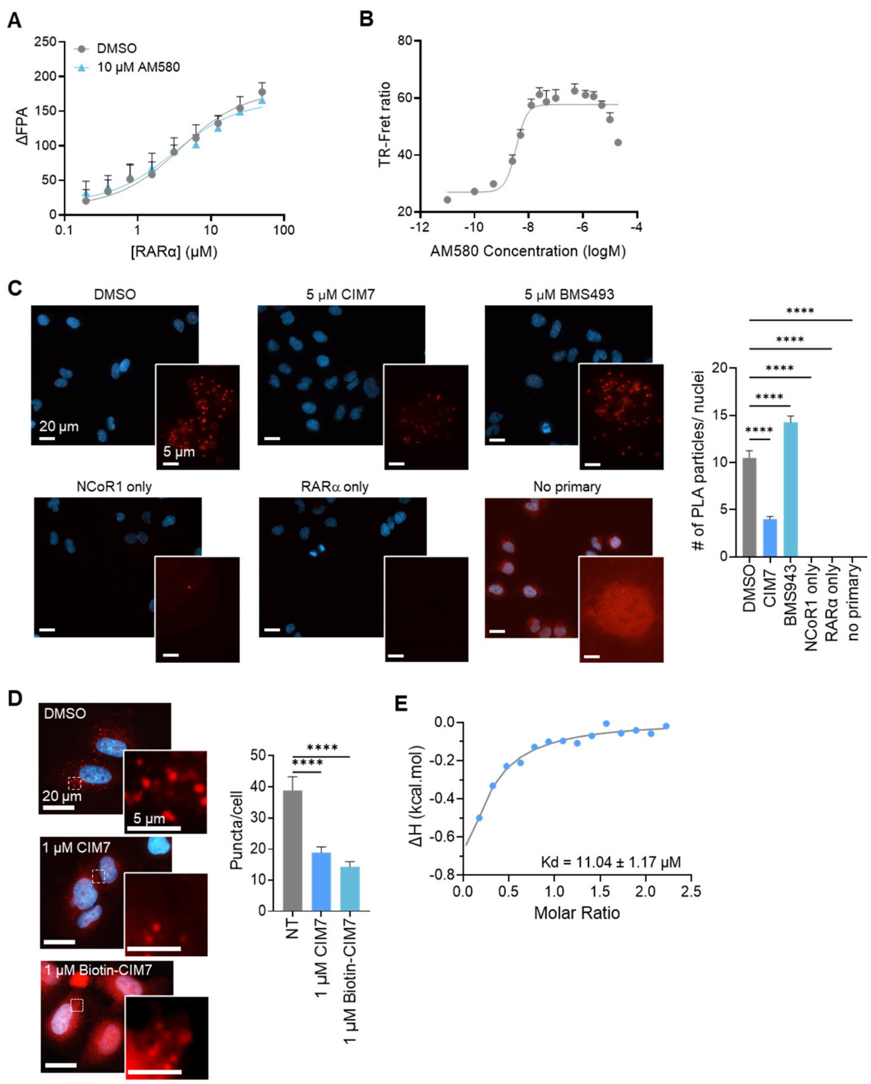

Appendix Figure S5 – Evaluation of AM580 and validation of proximity ligation assay (PLA) and biotin-CIM7.

(**A**) Fluorescence polarization anisotropy (FPA) to determine the binding affinity of NCoR1 to recombinant RARα in the presence of 10 μM AM580 or equal volume DMSO. n = 3 technical replicates. (**B**) TR-FRET evaluation of SRC binding to recombinant RARα with increasing concentrations of AM580. n = 3 technical replicates. (**C**) Validation of the Proximity ligation assay (PLA) from Fig. 4B. BMS493, which enhances NCoR1-RARα interaction, serves as a positive control and individual antibodies serve as negative controls. Full field images with insets of the NCoR1/RARα complex in red (left) and quantification of # of PLA particles/nuclei (right). Nuclei are highlighted with DAPI. Ordinary one-way ANOVA with Bonferroni's posthoc analysis was used. ****$p \leq 0.0001$. (**D**) CMA activity in A549 cells expressing KFERQ-PS-Dendra treated with 1 μM CIM7, 1 μM Biotin-CIM7, or equal volume DMSO for 24 hours. Representative images (left) and quantification of puncta per cell (right). Insets show higher magnification. Nuclei are highlighted with DAPI and insets show higher magnification of the red channel. Ordinary one-way ANOVA followed by Bonferroni's multiple comparisons post-hoc test was used. ****$p \leq 0.0001$. E Representative isothermal titration calorimetry curve of Biotin-CIM7 binding to recombinant RARα. This experiment was repeated twice with consistent results.

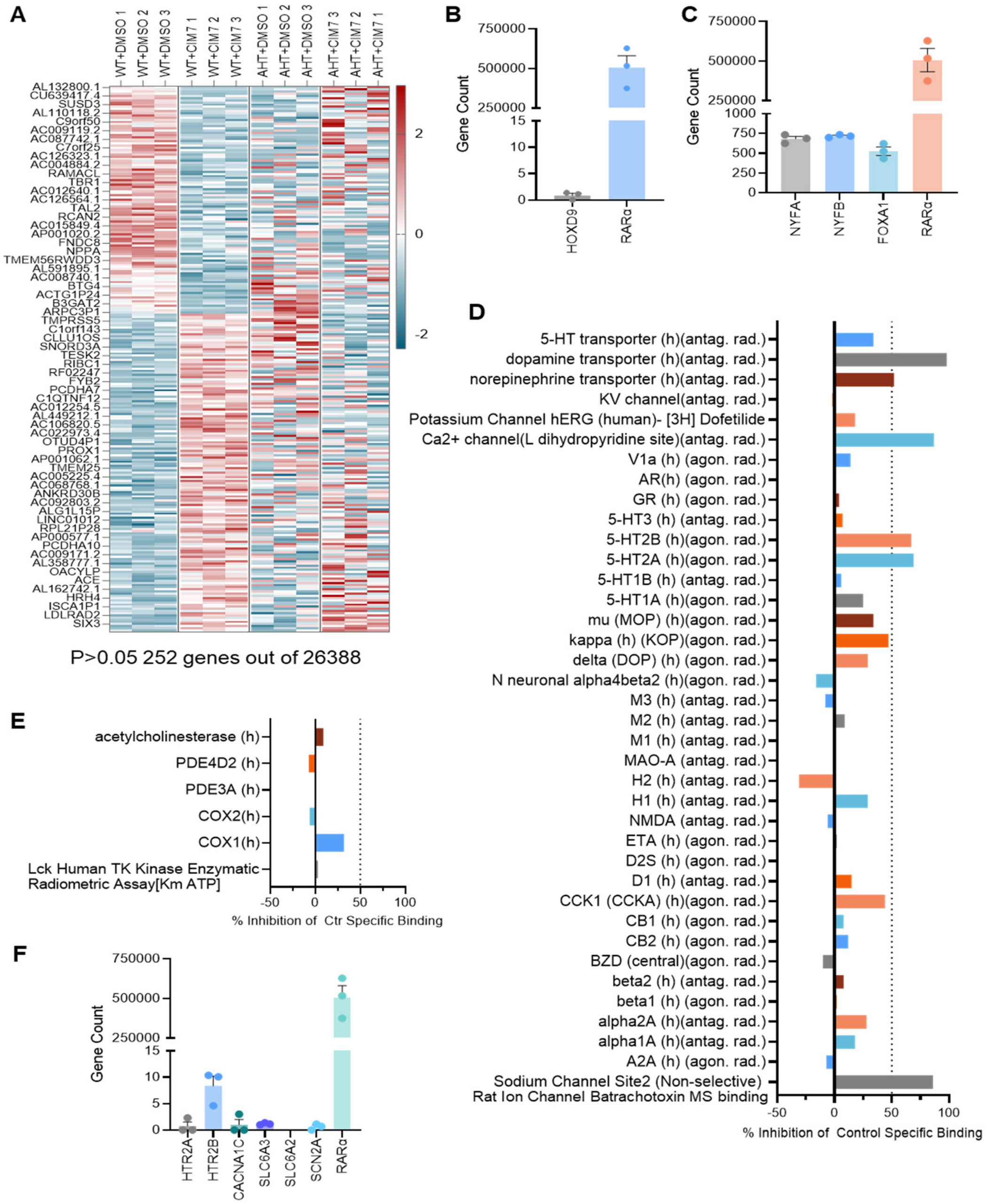

Appendix Figure S6 - Evaluation of potential off-target proteins.

(**A**) Transcriptional changes (significant p<0.05 out of 26,388 genes) detected in A549 cells treated with DMSO or CIM7 or expressing the AHT RARα mutant supplemented or not with CIM7. Three individual experiments contributing to the averages shown in Fig. 4E. (**B, C**) RNA expression of hits from TRANSFAC analysis in A549 cells expressing WT RARα and treated with DMSO as gene count based on RNA-seq analysis. (**D, E**) Results of SafetyScreen44 panel. Proteins with more than 50% inhibition of control specific binding (dashed line) from binding (**D**) or enzymatic (**E**) assays are considered potential off-targets. (**F**) RNA expression of potential off-targets identified in C in A549 cells expressing WT RARα and treated with DMSO as gene count based on RNA-seq analysis.

A

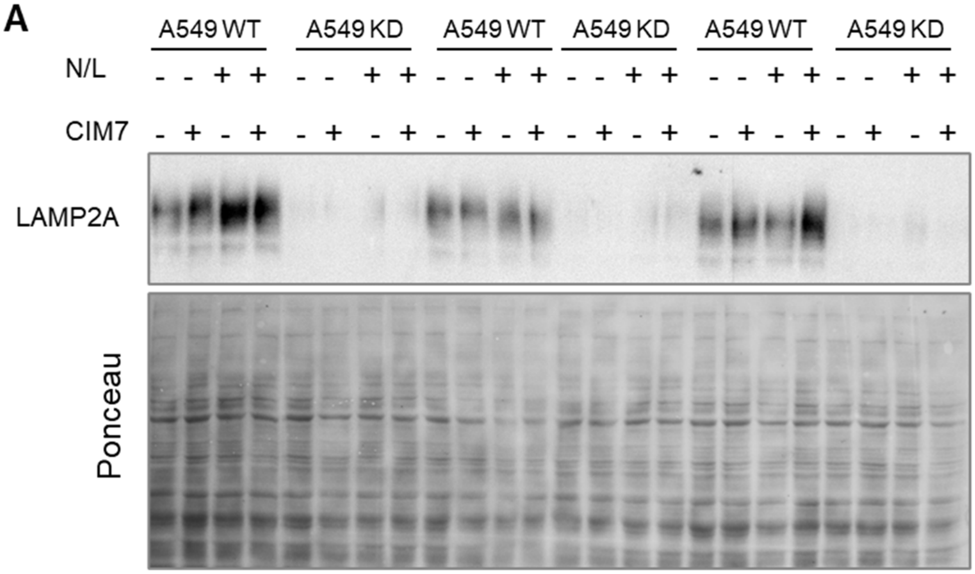

B

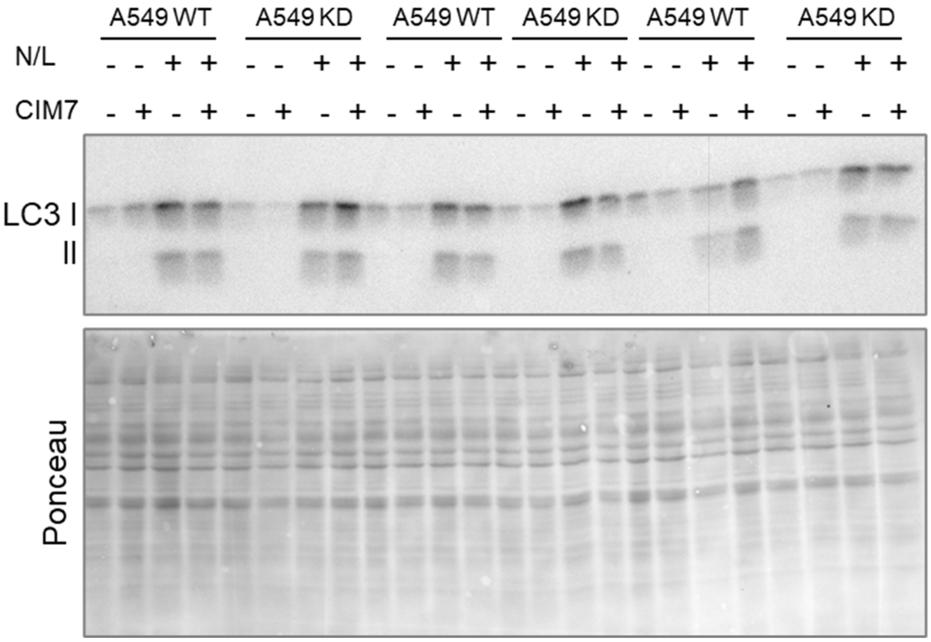

Appendix Figure S7 – Confirmation of LAMP2A knockdown and efficacy of lysosomal proteolysis inhibitor for proteomics.

(**A**) Immunoblot of LAMP2A protein levels in wild-type (WT) or LAMP2A knockdown (KD) A549 cells that were sent for proteomics in triplicate. Cells were treated with or without 5 μM CIM7 in the absence or presence of the lysosomal proteolysis inhibitors ammonium chloride and leupeptin (N/L). Ponceau staining is shown as loading control. (**B**) Immunoblot evaluation of LC3-II degradation in the same cells above to confirm inhibition of lysosomal proteolysis by N/L. Ponceau is shown as loading control.

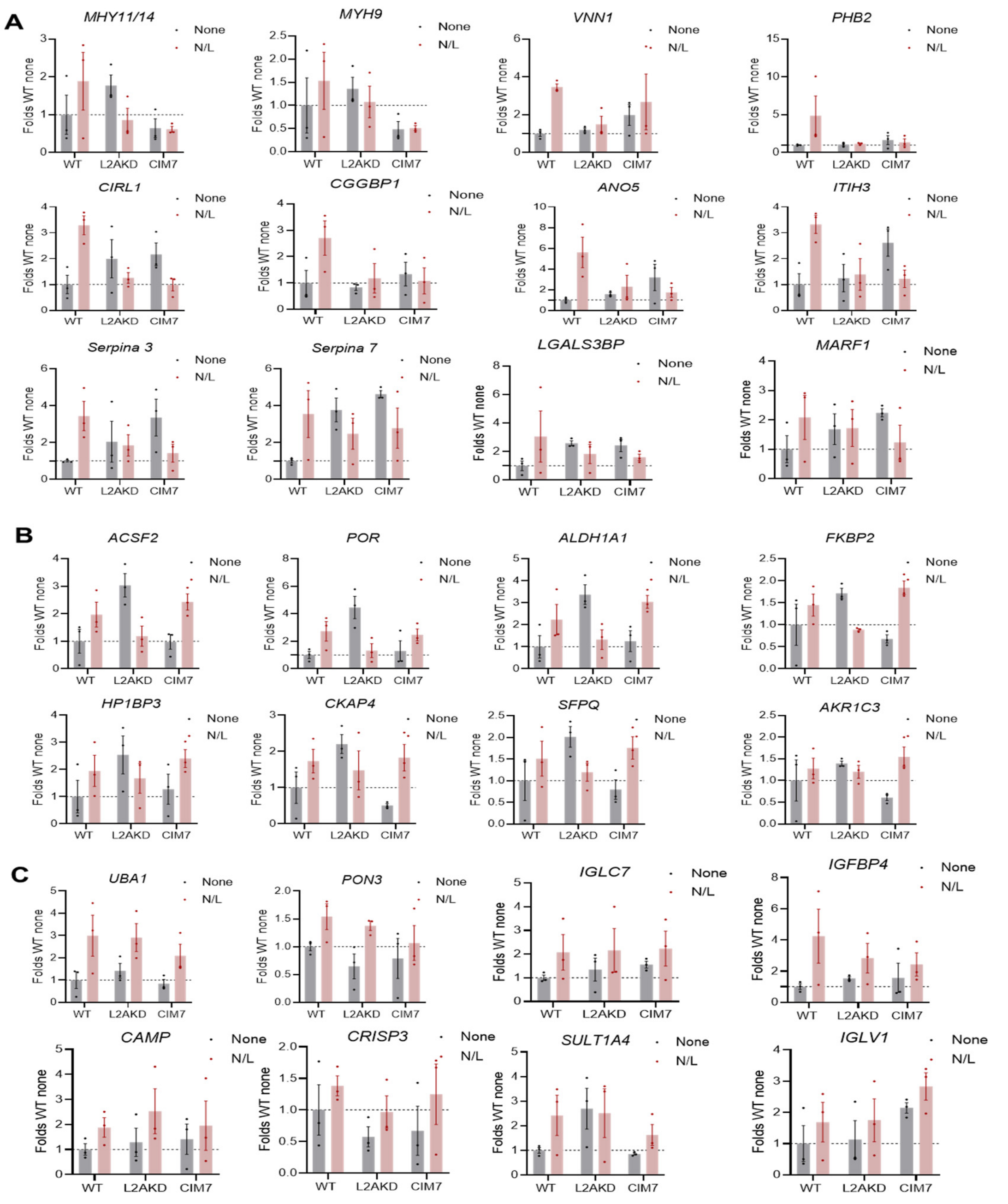

Appendix Figure S8 – Examples of the impact of CIM7 on the lysosomal degradation of different proteins.

(**A,B**) Changes in levels of proteins with pattern of CMA substrates (lysosomal degradation dependent of LAMP2A) whose degradation is inhibited (**A**) or not (**B**) upon treatment of A549 cells with CIM7. Lysosomal degradation was detected as the increase in intracellular levels upon inhibition of lysosomal proteolysis with ammonium chloride and leupeptin (N/L). (**C**) Changes in levels of proteins degraded in lysosomes by mechanisms other than CMA (lysosomal degradation independent of LAMP2A) upon treatment of A549 cells with CIM7.

Data information: Values are expressed relative to those in A549 cells wild-type (WT) in absence of N/L and are mean + SEM. Individual data points represent 3 individual experiments.

A

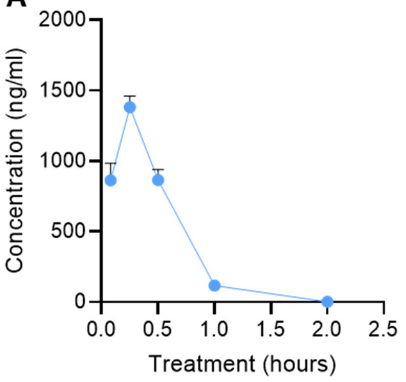

B

Compound ID	Test Concentration	% Protein Bound	% Recovery	Notes
**CIM7**	**10 µM**	**-92.37**	**1.26**	Test compound was below the limit of quantitation
Acebutolol	10 µM	5.64	74.29	
Quinidine	10 µM	75.03	119.050	
Sertraline	10 µM	98.17	109.09	
Warfarin	10 µM	87.78	107.60	

C

```
Human  1    MASNSSSCPTPGGGHLNGYPVPPYAFFFPPMLGGLSPPGALTTLQHQLPVSGYSTPSPAT  60
            MASNSSSCPTPGGGHLNGYPVPPYAFFFPPMLGGLSPPGALT+LQHQLPVSGYSTPSPAT
Mouse  1    MASNSSSCPTPGGGHLNGYPVPPYAFFFPPMLGGLSPPGALTSLQHQLPVSGYSTPSPAT  60

Human  61   IETQSSSSEEIVPSPPSPPPLPRIYKPCFVCQDKSSGYHYGVSACEGCKGFFRRSIQKNM  120
            IETQSSSSEEIVPSPPSPPPLPRIYKPCFVCQDKSSGYHYGVSACEGCKGFFRRSIQKNM
Mouse  61   IETQSSSSEEIVPSPPSPPPLPRIYKPCFVCQDKSSGYHYGVSACEGCKGFFRRSIQKNM  120

Human  121  VYTCHRDKNCIINKVTRNRCQYCRLQKCFEVGMSKESVRNDRNKKKKEVPKPECSESYTL  180
            VYTCHRDKNCIINKVTRNRCQYCRLQKCF+VGMSKESVRNDRNKKKKE PKPECSESYTL
Mouse  121  VYTCHRDKNCIINKVTRNRCQYCRLQKCFDVGMSKESVRNDRNKKKKEAPKPECSESYTL  180

Human  181  TPEVGELIEKVRKAHQETFPALCQLGKYTTNNSSEQRVSLDIDLWDKFSELSTKCIIKTV  240
            TPEVGELIEKVRKAHQETFPALCQLGKYTTNNSSEQRVSLDIDLWDKFSELSTKCIIKTV
Mouse  181  TPEVGELIEKVRKAHQETFPALCQLGKYTTNNSSEQRVSLDIDLWDKFSELSTKCIIKTV  240

Human  241  EFAKQLPGFTTLTIADQITLLKAACLDILILRICTRYTPEQDTMTFSDGLTLNRTQMHNA  300
            EFAKQLPGFTTLTIADQITLLKAACLDILILRICTRYTPEQDTMTFSDGLTLNRTQMHNA
Mouse  241  EFAKQLPGFTTLTIADQITLLKAACLDILILRICTRYTPEQDTMTFSDGLTLNRTQMHNA  300

Human  301  GFGPLTDLVFAFANQLLPLEMDDAETGLLSAICLICGDRQDLEQPDRVDMLQEPLLEALK  360
            GFGPLTDLVFAFANQLLPLEMDDAETGLLSAICLICGDRQDLEQPD+VDMLQEPLLEALK
Mouse  301  GFGPLTDLVFAFANQLLPLEMDDAETGLLSAICLICGDRQDLEQPDKVDMLQEPLLEALK  360

Human  361  VYVRKRRPSRPHMFPKMLMKITDLRSISAKGAERVITLKMEIPGSMPPLIQEMLENSEGL  420
            VYVRKRRPSRPHMFPKMLMKITDLRSISAKGAERVITLKMEIPGSMPPLIQEMLENSEGL
Mouse  361  VYVRKRRPSRPHMFPKMLMKITDLRSISAKGAERVITLKMEIPGSMPPLIQEMLENSEGL  420

Human  421  DTLSGQPGGGGRDGGGLAPPPGSCSPSLSPSSNRSSPATHSP  462
            DTLSGQ GGG RDGGGLAPPPGSCSPSLSPSS+RSSPAT SP
Mouse  421  DTLSGQSGGGTRDGGGLAPPPGSCSPSLSPSSHRSSPATQSP  462
```

D

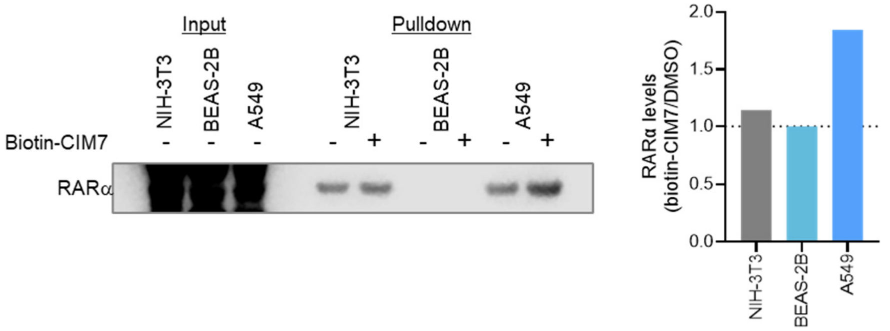

Appendix Figure S9 – CIM7 is distributed in mouse plasma but binds minimally to RARα in mouse fibroblasts (NIH-3T3) or has no interaction to RARα in human lung epithelial BEAS-2B cells. (**A**) Levels of CIM7 at the indicated times in plasma after i.p. (intraperitoneal) administration of 25 mg/kg CIM7. n = 3 mice per time point. (**B**) Plasma binding analysis of CIM7 and four control compounds in mouse plasma. Results are the average of two replicates. (**C**) BLAST alignment of the human (top line) and mouse (bottom line) protein sequence of RARα, with residues predicted to interact with CIM7 highlighted with black boxes. (**D**) Representative immunoblot (left) and quantification (right) for RARα of streptavidin pulldowns of NIH-3T3, BEAS-2B, or A549 cellular lysates incubated without additions or with biotin-CIM7 (50 µM).

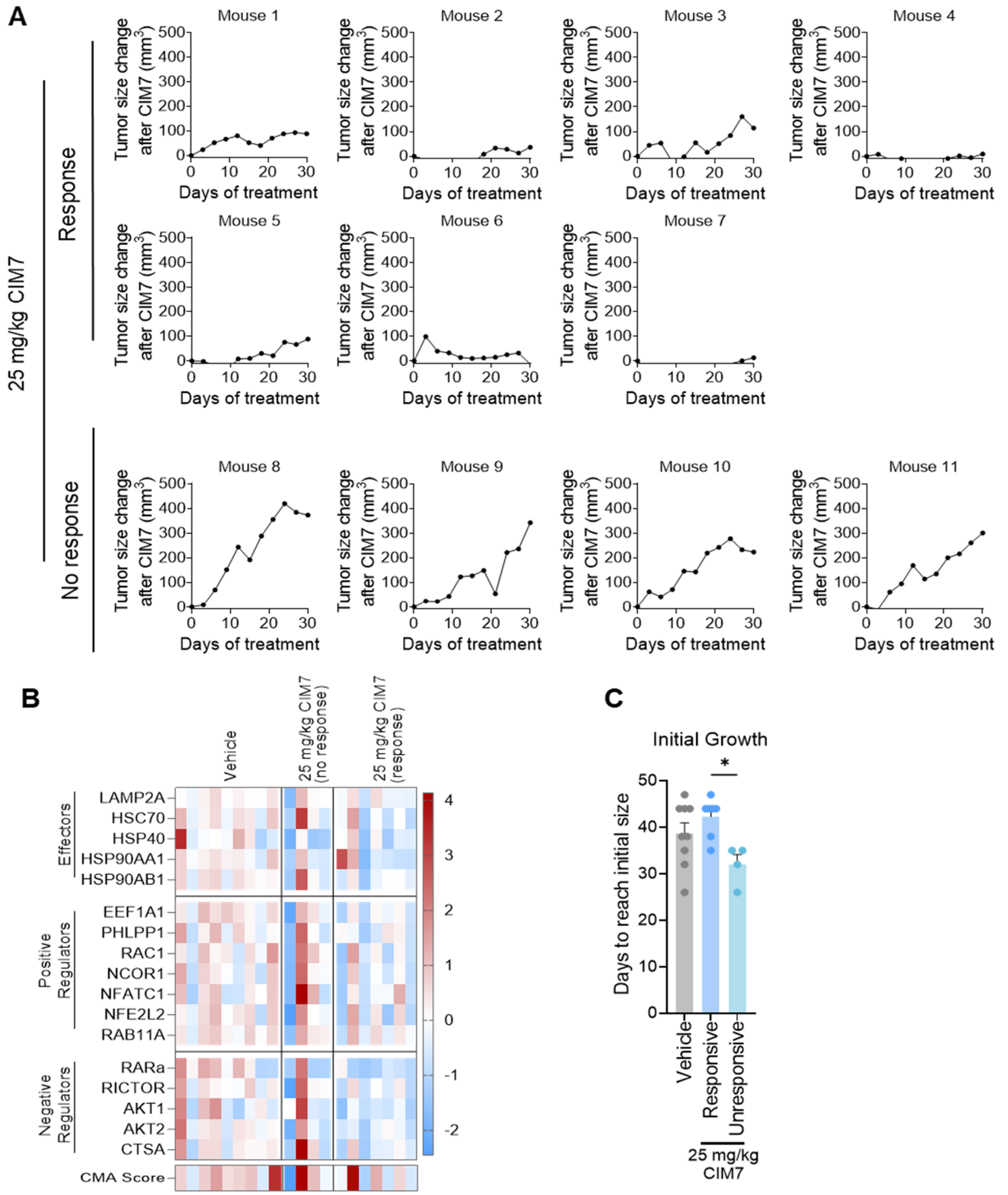

Appendix Figure S10 - Individual mouse response to CIM7 treatment. (**A**) Changes in tumor growth over time, measured every 3 days during the 30-day course of treatment in each of the individual mice that received 25 mg/kg (b.w.) CIM7. (**B**) Heatmap of transcriptional changes in CMA-related genes (top) and calculated CMA scores (bottom) in tumor tissue from each individual vehicle- or 25 mg/kg CIM7-treated mouse. (**C**) Days for tumor to reach target volume for injection (~150 mm^3) for vehicle- or 25 mg/kg CIM7-treated mice, separating unresponsive CIM7-treated mice from mice responsive to treatment.

Data information: All values are mean + SEM with individual data points to represent individual mice in bar graphs. Ordinary one-way ANOVA followed by Bonferroni's multiple comparisons post-hoc test (c) was used. *p≤0.05.

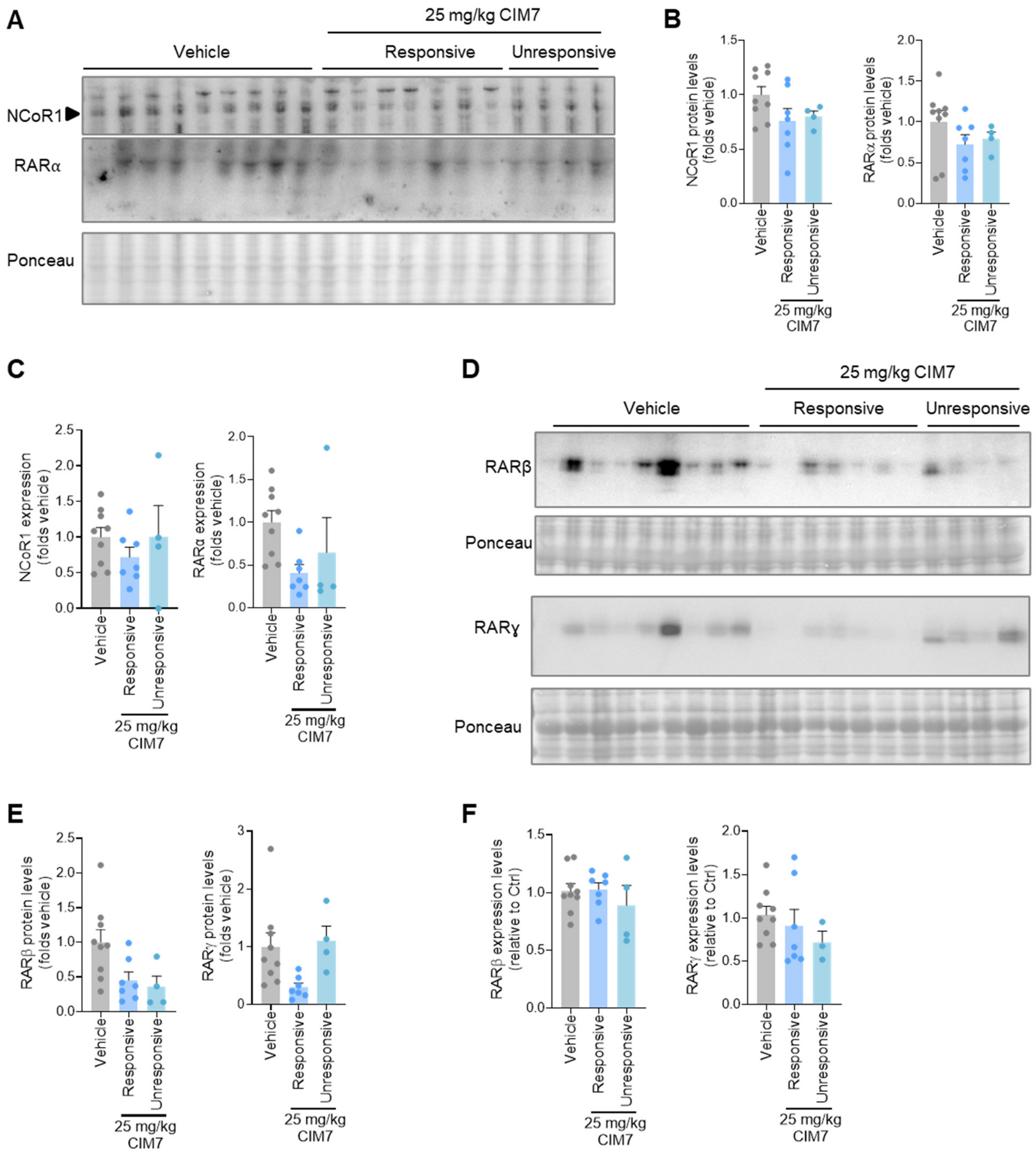

Appendix Figure S11 – Differences in NCoR1 and RAR in individual mice. (**A,B**) Immunoblot (**A**) and quantification (**B**) of NCoR1 and RARα in tumors from individual vehicle- or 25 mg/kg CIM7-treated mice. Ponceau is used as a loading control for immunoblot. (**C**) RNA expression by qPCR of NCoR1 and RARα in the same tumors as in A. (**D,E**) Immunoblot (**D**) and quantification (**E**) of RARβ and RARγ in tumors from A. Ponceau is used as a loading control for immunoblot. (**F**) RNA expression by qPCR of RARβ and RARγ in the same tumors as in A.

Data information: Values are mean + SEM. Individual data points represent individual mice. Ordinary one-way ANOVA followed by Bonferroni’s multiple comparisons post-hoc test was used.

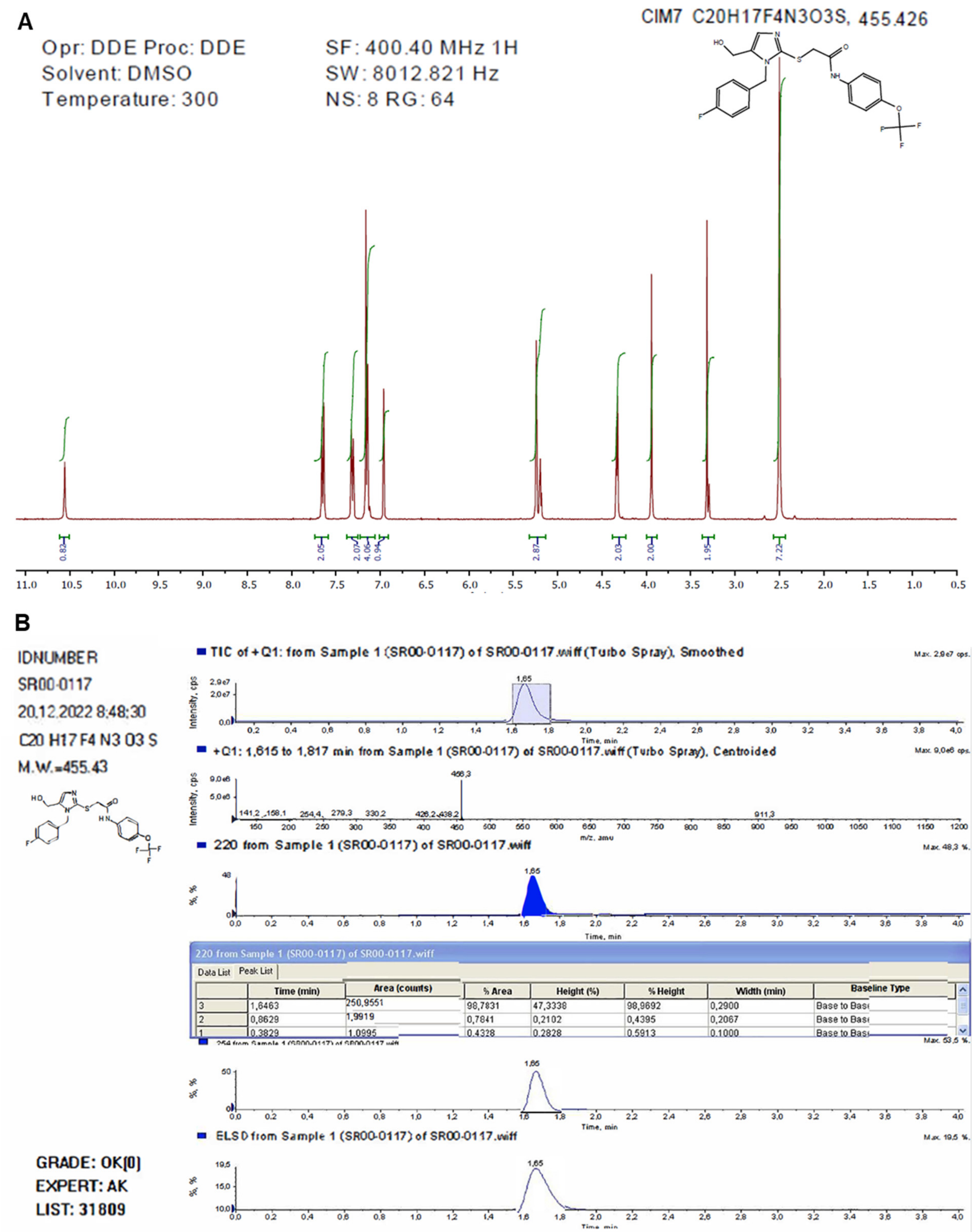

Appendix Figure S12 - Quality Control tests for CIM7 synthesis.
A,B NMR (**A**) and LC/MS (**B**) of CIM7 following synthesis.

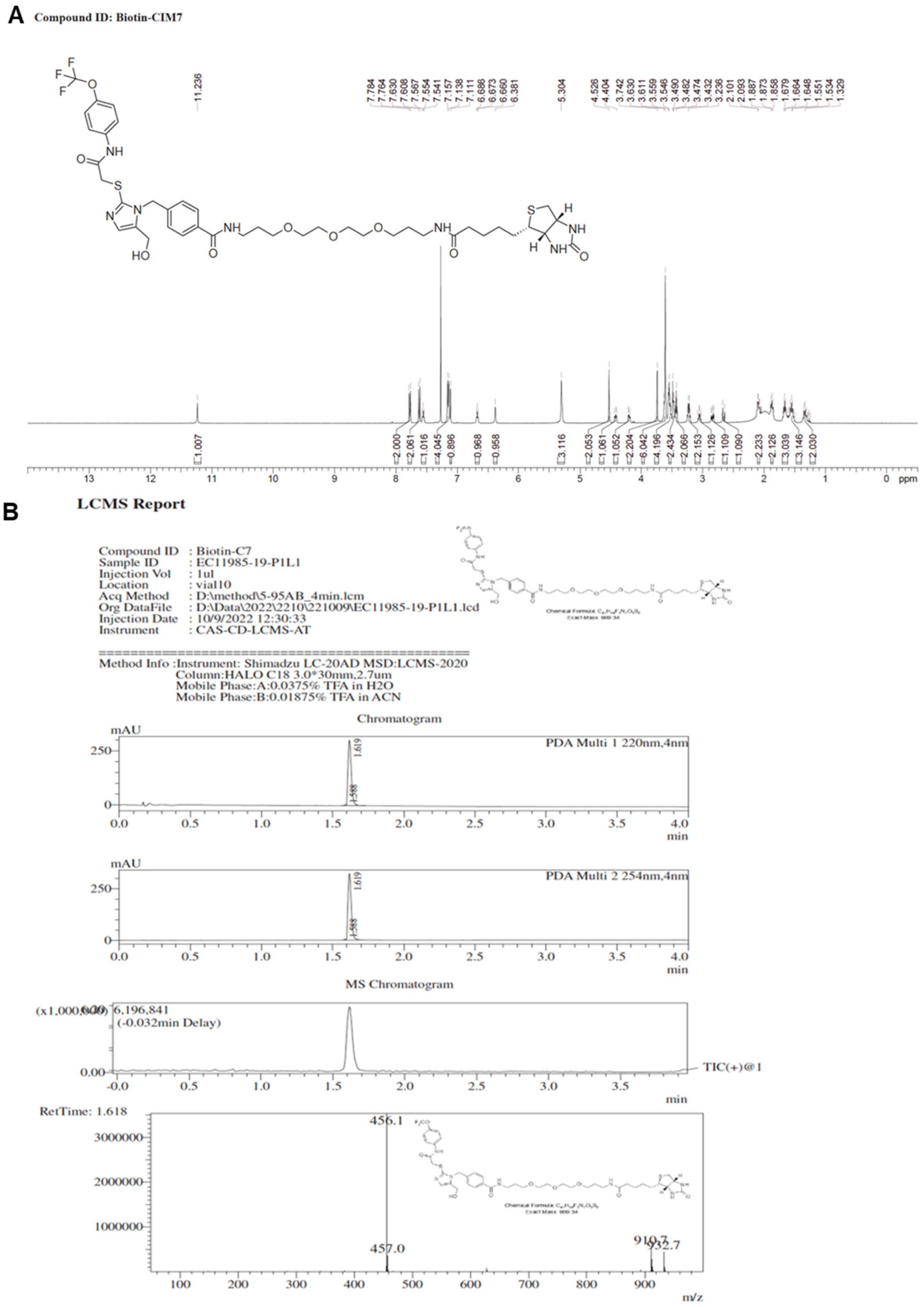

Appendix Figure S13 - Quality Control tests for biotin-CIM7 synthesis.
A,B NMR (**A**) and LC/MS (**B**) of biotin-CIM7 following synthesis.

Appendix Note S1

PATHOLOGY REPORT

HISTOPATHOLOGY AND COMPARATIVE PATHOLOGY FACILITY

ALBERT EINSTEIN COLLEGE OF MEDICINE

Comparative Pathologist: Dr. Elizabeth Neyens, Elizabeth.neyens@einsteinmed.org

Laboratory Manager: Laura Nanette Ramkissoon, laura.ramkissoon@einsteinmed.org

Lab: (718) 678-1043

Accession #: HP23-11273

Date: 5-03-23

Investigator: Mericka McCabe

Histology protocol:

The following organs are examined histologically: heart, lungs, kidneys, spleen and liver.

Organ Weights:

The mean weight of the spleens in the controls (n=12) was approximately 134 mg and the mean weight of the spleens in the treated animals (n=8) was approximately 199 mg; Therefore, there was a +/- 200% of increase in mean spleen weights. In order to gain more exactitude, these values should be compared to the body weight of the respective animals and mean values should be calculated per mean body weight.

Histopathology:

The spleens of all treated animals showed a slight (Grade 2) increased amount of extra-medullary hematopoiesis compared to a minimal increase (Grade 1) observed in the control animals. This change in the treated animals was always associated with the presence of brown pigment. The nature of the brown pigment was most likely associated with hemosiderosis in histiocytes or breakdown of premature/mature erythrocytes. Both splenic changes are most likely related to the treatment of the animals if no extra blood sampling was applied to these treated animals. Histopathologic changes correlated with the increases in spleen organ weights. The sometimes increased white pulp was seen as well in control animals as in treated animals and was therefore of no further toxicologic significance.

Remaining organs such as heart, lungs, kidneys and spleen did not show significant abnormalities and were therefore, interpreted as normal.

Conclusion: All treated animals showed treatment-related changes in spleen which were consistent with a slight increased extra-medullary hematopoiesis and the presence of brown pigment.

Appendix Table S1. Cell lines utilized for CMA score calculation

Cell Line	Classification
BEAS2B	Healthy Lung
CCD-11Lu	Healthy Lung
CCD-13Lu	Healthy Lung
CCD-16Lu	Healthy Lung
CCD-19Lu	Healthy Lung
CCD-25Lu	Healthy Lung
CCD-29Lu	Healthy Lung
CCD-33Lu	Healthy Lung
CCD-8Lu	Healthy Lung
LL24	Healthy Lung
LL47	Healthy Lung
LL86	Healthy Lung
MeT5A	Healthy Lung
MRC5	Healthy Lung
NuLi-1	Healthy Lung
WI-38	Healthy Lung
IMR90	Healthy Lung
A549	Non-small cell lung cancer
H1703	Non-small cell lung cancer
H23	Non-small cell lung cancer
H460	Non-small cell lung cancer
H520	Non-small cell lung cancer
H1299	Non-small cell lung cancer
H1648	Non-small cell lung cancer
H1792	Non-small cell lung cancer
H1944	Non-small cell lung cancer
H1975	Non-small cell lung cancer
H226	Non-small cell lung cancer
H292	Non-small cell lung cancer
H522	Non-small cell lung cancer
H661	Non-small cell lung cancer
SK-MES-1	Non-small cell lung cancer

Appendix Table S2. Small molecules utilized for CMA inhibitor screening.

Compound ID	ChemDiv IDNUMBER	Molecular Formula	MOLWEIGHT
A1	4466-0640	C12H11N5O	241.25
B1	8005-2486	C14H15NO3	245.28
C1	8017-0975	C21H22ClN5O2S	443.96
D1	8019-2477	C26H31N3O2	417.56
E1	8019-9092	C11H17N3O4	255.28
F1	8020-3060	C16H14ClFN4O	332.77
G1	8640-0681	C18H22N4O2S2	390.53
H1	C073-4421	C32H37N3O6	559.67
A2	C102-0342	C11H12N4O	216.24
B2	C200-7051	C12H9N3OS	243.29
C2	C270-0110	C12H10ClN3O	247.69
D2	C270-0491	C20H24ClN3O	357.89
E2	C324-0503	C12H14ClN3O2S	299.78
F2	C636-2450	C17H11Cl2FN2O2S	397.26
G2	CIM795-0058	C16H21N5O2S	347.44
H2	C889-0386	C20H24N2O2	324.43
A3	D012-0401	C17H17N3O	279.34
B3	D144-0924	C22H23NO5	381.43
C3	D269-0446	C14H23N3OS	281.42
D3	D305-2154	C18H23N5O	325.42
E3	D340-1050	C14H22N4O3	294.36
F3	D345-0362	C24H22N2OS	386.52
G3	D345-0629	C15H24N2OS	280.44
H3	D420-6417	C24H24N4O3	416.48
A4	D684-0028	C9H14N8S	266.33
B4	D718-0912	C23H24N4O2	388.47
C4	D724-0801	C15H16N4	252.32
D4	E013-1940	C13H15BrN2O2S	343.24
E4	E016-2712	C24H25N5O2	415.50
F4	E213-0535	C16H16N4O2S2	360.46
G4	E228-2374	C19H20FN3O2	341.39
H4	E230-0461	C21H30N2O	326.49
A5	E525-0016	C26H29ClN4O2	465.00
B5	E646-2834	C25H27N3O6S	497.57
C5	E787-0934	C24H30N2O6	442.52
D5	E853-1101	C24H29ClN4O4	472.98
E5	F145-0249	C19H17F2N3O2S	389.43
F5	F222-1077	C23H27N3O4S	441.55
G5	F532-3822	C20H22N4O4	382.42
H5	F711-0408	C19H23ClN4O3S	422.94
A6	F711-0557	C22H25N3O2S	395.53
B6	F807-0272	C15H17N3O2S	303.39
C6	G282-2068	C27H35N5O6	525.61
D6	G290-1050	C21H19N3O3S	393.47
E6	G331-0068	C19H17F2N3O	341.36
F6	G336-0433	C24H27FN6O2S	482.58

G6	G365-0268	C16H19N3O3	301.35
H6	G434-1107	C18H18ClN3O4	375.81
A7	G533-0431	C23H28N2O4	396.49
B7	G621-0052	C20H27N3O2S	373.52
D7	G741-1687	C25H26N4O4S	478.57
E7	G765-0451	C16H18N2O4	302.33
F7	G786-1798	C23H25N5O5S	483.55
G7	G826-0370	C24H28N4O4	436.52
H7	G856-4211	C23H27N3O3	393.49
A8	K279-0628	C29H29N5O4S	543.65
B8	K279-1447	C30H35N5O4S	561.71
C8	K284-3371	C28H27ClN4O3S	535.07
D8	K284-5572	C32H34N6O5S	614.73
E8	K405-3684	C15H16ClN5O	317.78
F8	K784-5964	C29H38N2O4	478.64
G8	L491-0631	C21H23N3O3	365.44
H8	L644-0084	C17H19N3O3	313.36
A9	L673-0090	C16H19N3O4S	349.41
B9	L846-0043	C16H18N4O2	298.35
C9	L879-0155	C20H28N4O2	356.47
D9	M460-1495	C19H21FN6O2	384.42
E9	P091-0504	C18H16ClFN2O	330.79
F9	P130-0253	C21H27N5O	365.48
G9	P165-2864	C27H28N4O3	456.55
H9	P759-1299	C17H20N4O2	312.37
A10	S032-1506	C21H23N3O2	349.44
B10	T482-1600	C14H13N5O	267.29
C10	T499-0236	C21H24N4O	348.45
D10	T636-1102	C17H20N4OS	328.44
E10	T842-1499	C20H22FN5O	367.43
F10	V001-5057	C23H22ClF3N2O2	450.89
G10	V004-9837	C26H26ClN3O2	447.97
H10	V008-3442	C27H34FN3O4	483.59
A11	V009-2010	C23H23F4N3O	433.45
B11	V009-2046	C24H27F4N3O2	465.50
C11	V012-8378	C32H39FN2O4	534.68
D11	V014-2162	C28H33Cl2N3O2	514.50
E11	V014-6583	C17H18F6N2O2	396.34
F11	V016-2451	C15H16FN3O2	289.31
G11	V016-8395	C26H26FN3O4	463.51
H11	V018-9947	C26H30N4O	414.56
A12	V019-1297	C28H28ClFN2O4S	543.06
B12	V022-1658	C24H35N3O4	429.56
C12	Y010-0170	C18H22N2O4	330.39
D12	Y020-1267	C21H26N4O3S	414.53
E12	Y031-2038	C16H24N4O3S	352.46
F12	Y300-1378	C16H18FN3O3S	351.40
CIM7	G621-0375	C20H17F4N3O3S	455.43
CIM7.1	G621-0055	C19H21N3O3S	371.46

CIM7.2	G621-0319	C21H22FN3O2S	399.49
CIM7.3	G621-0413	C22H23N3O3S	409.51
CIM7.4	G786-1005	C19H15F4N3OS	409.41

Appendix Table S3. Sequence of forward (F) and reverse (R) primers used for qPCR

Lamp2a	F-5'-TGACGACAACTTCCTTGTGC-3'<br>R-5'-AGCATGATGGTGCTTGAGAC-3'
HSC70	F-5'-ACCTACTCTTGTGTGGGTGTT-3'<br>R-5'-GACATAGCTTGGAGTGGTTCG-3'
HSP40	F-5'-CCGTTGAAGTCAAATGAGCCG-3'<br>R-5'-CCAAACTTGTCGTAGATGCCTC-3'
HSP90AA1	F-5'-CATAACGATGATGAGCAGTACGC-3'<br>R-5'-GACCCATAGGTTCACCTGTGT-3'
HSP90AB1	F-5'-AGAAATTGCCCAACTCATGTCC-3'<br>R-5'-ATCAACTCCCGAAGGAAAATCTC-3'
PHLPP1	F-5'-CCTCATCCGCTTCTATGCAGG-3'<br>R-5'-GCATCTTGCCTTTACGGACAT-3'
GFAP	F-5'-CTGCGGCTCGATCAACTCA-3'<br>R-5'-TCCAGCGACTCAATCTTCCTC-3'
RICTOR	F-5'-GCTAGGTGCATTGACATACAACA-3'<br>R-5'-AGTGCTAGTTCACAGATAATGGC-3'
RAC1	F-5'-ATGTCCGTGCAAAGTGGTATC-3'<br>R-5'-CTCGGATCGCTTCGTCAAACA-3'
RARa	F-5'-CACACACCTGAGCAGCATCAC-3'<br>R-5'-CGGTCCTTTGGTCAAGCAGT-3'
AKT1	F-5'-CTACCCACACAGCAGTACGC-3'<br>R-5'-AAGTCGCTGGTGTTAAGCCG-3'
AKT2	F-5'-AGGCACGGGCTAAAGTGAC-3'<br>R-5'-CTGTGTGAGCGACTTCATCCT-3'
EEF1A1	F-5'-TGTCGTCATTGGACACGTAGA-3'<br>R-5'-ACGCTCAGCTTTCAGTTTATCC-3'
CTSA	F-5'-TCCCAGCATGAACCTTCAGG-3'<br>R-5'-AGTAGGCAAAGTAGACCAGGG-3'
NFATc1	F-5'-TGTGCCGGAATCCTGAAACTC-3'<br>R-5'-GAGCATTCGATGGGGTTGGAG-3'
NFe2L2	F-5'-TCAGCGACGGAAAGAGTATGA-3'<br>R-5'-CCACTGGTTTCTGACTGGATGT-3'
Rab11a	F-5'-CAACAAGAAGCATCCAGGTTGA-3'<br>R-5'-GCACCTACAGCTCCACGATAAT-3'
NCOR1	F-5'-ACACCGCAGTATTGTCCAAAT-3'<br>R-5'-CACCTGGTTTGTCTTGATGTTCT-3'
B2M	F-5'-CCCAAGATAGTTAAGTGGGATCGA-3'<br>R-5'-CCAAATGCGGCATCTTCAA-3'
b-actin	*F-5'*-CATGTACGTTGCTATCCAGGC-*3'*<br>*R-5'*-CTCCTTAATGTCACGCACGAT-*3'*
TBP	F-5'-TGTATCCACAGTGAATCTTGGTTG-3'<br>R-5'-GGTTCGTGGCTCTCTTATCCTC-3'

Appendix Methods

Chemical Synthesis of CIM7

All chemical reagents and solvents were obtained from commercial sources (Aldrich, Acros, Fisher) and used without further purification unless otherwise noted. Analytical thin layer chromatography (TLC) was performed on aluminum-backed Silicycle silica gel plates (250 mm film thickness, indicator F254). Compounds were visualized using a dual wavelength (254 and 365 nm) UV lamp, and/or staining with CAM (cerium ammonium molybdate) or KMnO4 stains. NMR spectra were recorded on Bruker DRX400. ^{1}H chemical shifts (d) are reported relative to tetramethyl silane (TMS, 0.00/0.00 ppm) as internal standard or to residual solvent (CDCl3: 7.26/77.16 ppm; dmso-d6: 2.50/39.52 ppm). Purity and identity of all synthesized compounds were confirmed by LC-MS analysis performed on Shimadzu Analytical 10Avp equipped with PE SCIEX API 165 mass-, Sedex 75 ELSD-, and Shimadzu UV- (254 and 215) detectors. Separation was achieved with C18 column 100 x 4.6 mm, 5.0 mm, pore size 100 Å, wateracetonitrile+0.1 TFA, gradient 5 to 87 for 10 minutes. Preparative HPLC purification was carried out on Shimadzu instrument equipped with SPD-10Avp detector and FRC-10A fraction collector. Separation was achieved with a column YMC-Pack ODS-AQ 250×20 mml, S-10 mm, 12 nm, gradient solution A – solution B (A: 1000 ml H_2O-226 ml TFA; B: 1000 ml CH_3CN-226 ml TFA).

Scheme of synthesis

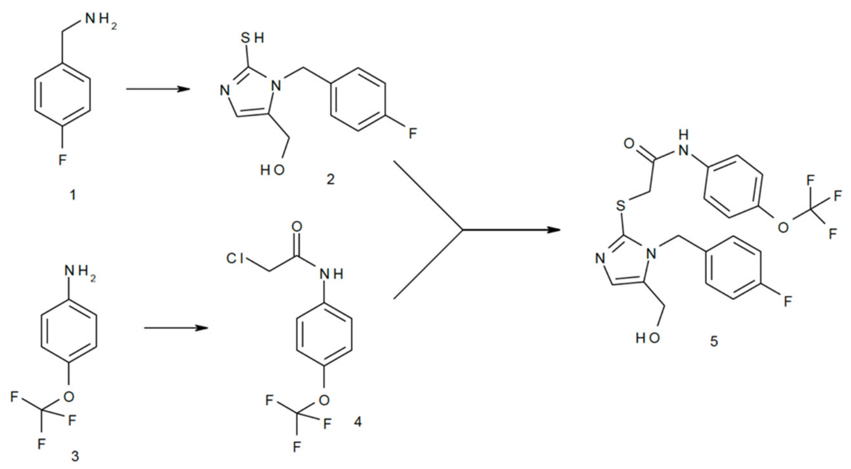

Step 1. [1-(4-fluorobenzyl)-2-mercapto-1H-imidazol-5-yl]methanol (2)

To an ice cold solution of the (4-fluorophenyl)methaneamine (1) (4.33 g, 34.6 mmol) in acetonitrile (50 ml) was added conc. HCl (2.5 ml, 34.6 mmol, 1 equiv) over 3 minutes with vigorous stirring, white solid was formed. Then propionic acid (2.5 ml), 1,3-dihydroxypropan-2-one (5.3 g, 59 mmol, 1.7 equiv) and KSCN (3.4 g, 34.6 mmol, 1 equiv) were added and the mixture was stirred at 70°C for 2 hours. After cooling, the mixture was diluted with water (100 ml) and stirred for 20 minutes. The precipitate obtained was collected by filtration, washed with water and cooled ethanol, and then dried to obtain the title compound (2) (7 g, 85%).

1H-NMR (d6-DMSO, 300 MHz) 12.17 (s, 1H), 7.35-7.25 (m, 2H), 7.20-7.10 (m, 2H), 6.85 (s, 1H), 5.32 (s, 2H), 5.26 (s, 1H, -OH), 4.13 (s, 2H).

Step 2. 2-chloro-N1-[4-(trifluoromethoxy)phenyl]acetamide (4)

To a solution of 4-(trifluoromethoxy)aniline (3) (3 g, 16.9 mmol, 1 equiv) in DCM (30 ml) was added TEA (2.59 ml, 18.6 mmol, 1.1 equiv), followed by chloroacetyl chloride (2.1 g, 18.6 mmol, 1.1 equiv) at 0°C, and the reaction mixture was stirred at room temperature for 4 hours. The extra solvent was removed in vacuo, the residue obtained was purified by column chromatography on silica gel (eluent: DCM / EtOAc (4:1) to provide 3 g (70%) of the title compound (4).

Step 3. 2-[1-(4-fluorobenzyl)-5-(hydroxymethyl)-1H-imidazol-2-yl]sulfanyl-N1-[4-(trifluoromethoxy)phenyl]acetamide (5)

To a solution of compound (2) (1 g, 4.2 mmol, 1 equiv) in MeOH (10mL) was added NaOH (0.33 g, 8.4 mmol, 2 equiv), followed by portion wise addition of compound (4) (1.17 g, 4.62 mmol, 1.1 equiv) at 0°C. The reaction mixture was stirred at room temperature overnight. The solvent was removed in vacuo, the residue obtained was diluted with water (20 ml) and EtOAc (20 ml), layers were separated, organic layer was washed with water, brine, dried over Na_2SO_4, and concentrated under reduced pressure. The residue was purified by column chromatography on silica gel (eluent: DCM) to provide 1 g (52%) of the title compound (5).

^{1}H NMR (400 MHz, DMSO-d6) δ 10.56 (s, 1H), 7.69 – 7.60 (m, 2H), 7.31 (d, J = 8.6 Hz, 2H), 7.19 – 7.11 (m, 4H), 6.96 (s, 1H), 5.24 (s, 2H), 5.19 (t, J = 5.2 Hz, 1H), 4.33 (d, J = 5.2 Hz, 2H), 3.94 (s, 2H). LCMS (m/z) (for $C_{20}H_{17}F_4N_3O_3S$, M+H): calculated: 455.4, found: 455.4.

Chemical Synthesis of Biotin-CIM7

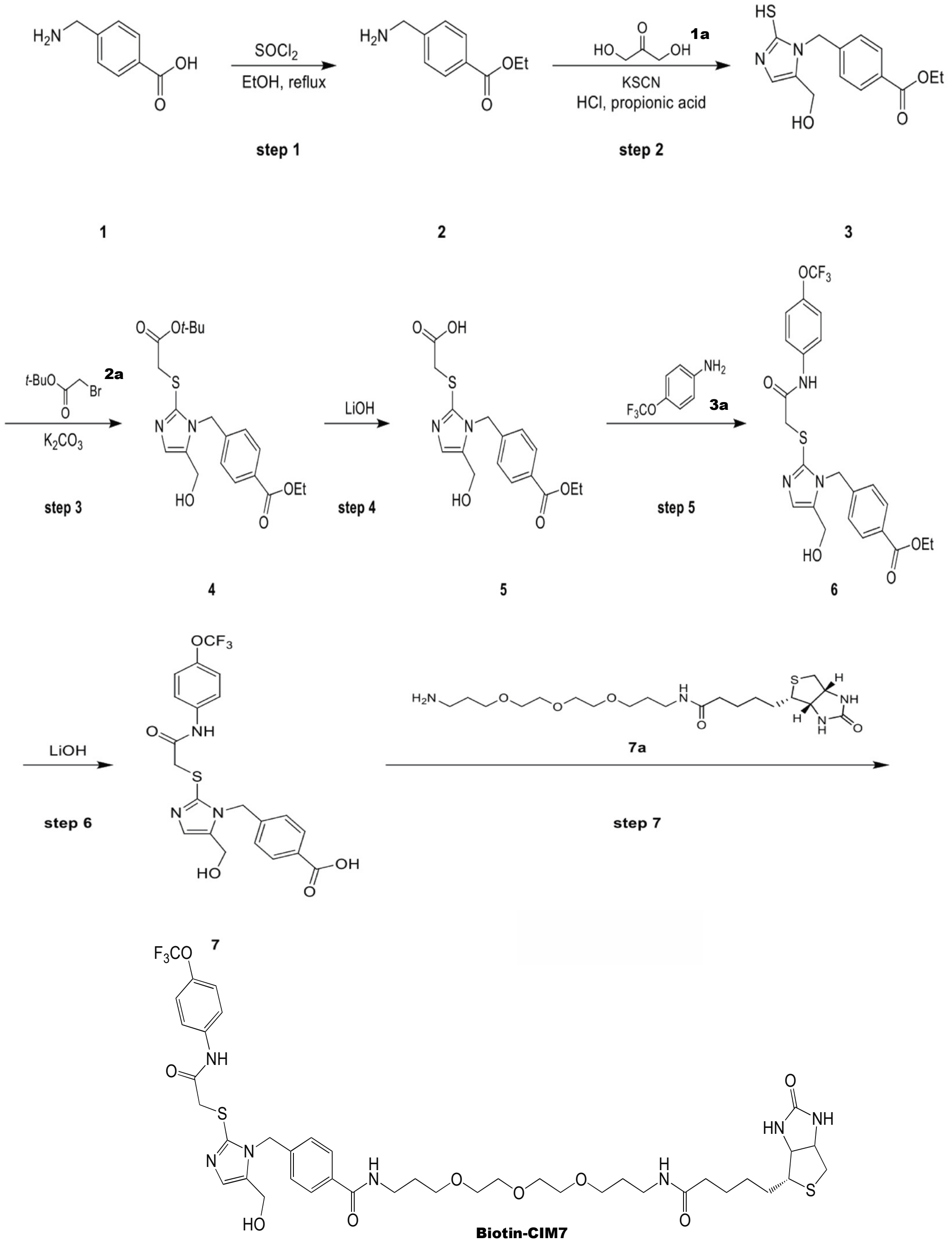

Step 1: To a solution of compound 1 (10.0 g, 66.2 mmol, 1.00 eq) in EtOH (100 ml) was added dropwise H_2SO_4 (648 mg, 6.62 mmol, 352 ml, 0.100 eq) at 0°C. The resulting mixture was stirred at 80°C for 12 hours. LCMS (product: RT = 0.189 min, m/z = 163.5 $[M+H]^+$) showed compound 1 was consumed completely and 99.6% of compound 2 was detected. The reaction mixture was concentrated under reduced pressure to remove EtOH. The residue was diluted with ethyl acetate 150 ml and was slowly added 35% NaOH aqueous solution to adjust pH = 7. The residue was extracted with ethyl acetate 200 ml (100 ml * 2), dried over Na_2SO_4, filtered and concentrated under reduced pressure to give compound 2 (11.0 g, 60.7 mmol, 91.8% yield) as a yellow oil.

Step 2: To a solution of compound 2 (4.00 g, 22.3 mmol, 1.00 eq) in ACN (100 ml) was added dropwise HCl (2.20 g, 22.3 mmol, 2.16 mL, 37.0% purity, 1.00 eq) at 0°C. And then compound 1a (3.42 g, 37.9 mmol, 1.70 eq), KSCN (2.17 g, 22.3 mmol, 2.17 ml, 1.00 eq) and propionic acid (1.65 g, 22.3 mmol, 1.67 ml, 1.00 eq) was added dropwise at 0°C. The resulting mixture was stirred at 70°C for 3 hours. LCMS (product: RT = 0.319 min, m/z = 293.3 $[M+H]^+$) showed compound 2 was consumed completely and 95.2% of compound 3 was detected. The reaction mixture was filtered and the filter cake was washed with ethanol (100 mL). The filter cake was concentrated under reduced pressure to give compound 3 (4.76 g, 16.3 mmol, 73.56% yield) as a white solid.

Step 3: A mixture of compound 3 (5.00 g, 17.1 mmol, 1.00 eq), compound 2a (4.00 g, 20.5 mmol, 3.03 ml, 1.20 eq), K_2CO_3 (4.73 g, 34.1 mmol, 2.00 eq) in MeOH (50.0 ml) was degassed and purged with N_2 three times, and then the mixture was stirred at 20°C for 3 hours under N_2 atmosphere. TLC (Petroleum ether : Ethyl acetate = 0 : 1, compound 3 R_f = 0.30, compound 4 R_f = 0.10) indicated compound 3 was consumed completely, and one major new spot with larger polarity was detected. The reaction mixture was filtered and concentrated under reduced pressure to get compound 4 (6.90 g, 16.9 mmol, Crude) as a white solid.

Step 4: A mixture of compound 4 (5.00 g, 12.3 mmol, 1.00 eq), TFA (2.81 g, 24.6 mmol, 1.83 ml, 2.00 eq) in MeOH (50.0 ml) was degassed and purged with N_2 three times, and then the mixture

was stirred at 20°C for 12 hours under N_2 atmosphere. LCMS (product: RT = 0.276 min, m/z = 351.0 $[M+H]^+$) showed compound 4 was consumed completely and 77.0% of compound 5 was detected. The reaction mixture was adjusted pH = 3 with HCl (1.00 M) and concentrated under reduced pressure to remove MeOH. The residue was diluted with H_2O 100 ml and extracted with Ethyl acetate 200 ml (100 ml * 2). The combined organic layers were washed with brine 200 ml (100 ml * 2), dried over Na_2SO_4, filtered and concentrated under reduced pressure to give compound 5 (2.88 g, 8.22 mmol, 66.8% yield) as a white solid.

Step 5: A mixture of compound 5 (3.00 g, 8.56 mmol, 1.00 eq), compound 3a (1.52 g, 8.56 mmol, 1.16 ml, 1.00 eq), EDCI (1.64 g, 8.56 mmol, 1.00 eq), in pyridine (50.0 ml) was degassed and purged with N_2 three times, and then the mixture was stirred at 20°C for 3 hours under N_2 atmosphere. LCMS (product: RT = 0.391 min, m/z = 510.2 $[M+H]^+$) showed compound 5 was consumed completely and 54.6% of compound 6 was detected. The reaction mixture was filtered and concentrated under reduced pressure to give a residue. The crude product was purified by column chromatography (Petroleum ether : Ethyl acetate = 5 : 1 to 1 : 2) to obtain compound 6 (3.00 g, 5.89 mmol, 68.8% yield) as a yellow oil.

Step 6: A mixture of compound 6 (2.00 g, 3.93 mmol, 1.00 eq), NaOH (314 mg, 7.85 mmol, 2.00 eq), in MeOH (20.0 ml) was degassed and purged with N_2 three times, and then the mixture was stirred at 20°C for 1 hour under N_2 atmosphere. LCMS (product: RT = 0.293 min, m/z = 482.2 $[M+H]^+$) showed compound 6 was consumed completely and 88.6% of compound 7 was detected. The reaction mixture was adjusted pH = 3 with HCl (1.00 M) and concentrated under reduced pressure to remove MeOH. The residue was diluted with H_2O 50.0 ml and extracted with Ethyl acetate 100 ml (50.0 ml * 2). The combined organic layers were washed with brine 100 ml (50.0 ml * 2), dried over Na_2SO_4, filtered, and concentrated under reduced pressure to give compound 7 (1.00 g, 2.08 mmol, 52.9% yield) as a white solid.

Step 7: A mixture of compound 7 (103 mg, 214 mmol, 1.20 eq), compound 7a (80.0 mg, 179 mmol, 1.00 eq), EDCL (41.2 mg, 215 mmol, 1.20 eq), in pyridine (10.0 ml) was degassed and

purged with N_2 three times, and then the mixture was stirred at 20°C for 4 hours under N_2 atmosphere. LCMS (product: RT = 0.370 min, m/z = 910.7 $[M+H]^+$, SM: RT = 0.343) showed 36.0% of compound 7 was remained and 59.1% of Biotin-CIM7 was detected. The reaction mixture was filtered and concentrated under reduced pressure to give a residue. The crude product was purified by reversed-phase HPLC (16 - 46% 45 min; 0.1% FA condition) to obtain Biotin-CIM7 (50.0 mg, 54.9 mmol, 30.6% yield) as a white solid.

LCMS: Biotin-CIM7 product: RT = 1.619 mins, m/z = 910.7 $[M+H]^+$ ^1H NMR: Biotin-CIM7, (400 MHz, Chloroform-*d*): δ 11.24 (s, 1H), 7.77 (d, *J* = 8.3 Hz, 2H), 7.62 (d, *J* = 9.0 Hz, 2H), 7.55 (t, *J* = 5.2 Hz, 1H), 7.15 (d, *J* = 7.4 Hz, 4H), 7.11 (s, 1H), 6.67 (t, *J* = 5.4 Hz, 1H), 6.38 (s, 1H), 5.30 (s, 3H), 4.53 (s, 2H), 4.45 - 4.39 (m, 1H), 4.23 - 4.17 (m, 1H), 3.74 (s, 2H), 3.64 - 3.60 (m, 6H), 3.57 - 3.51 (m, 4H), 3.50 - 3.46 (m, 2H), 3.43 (t, *J* = 5.8 Hz, 2H), 3.23 (q, *J* = 6.1 Hz, 2H), 3.09 - 3.01 (m, 1H), 2.84 (dd, *J* = 4.9, 12.9 Hz, 1H), 2.66 (d, *J* = 12.9 Hz, 1H), 2.11 - 2.07 (m, 2H), 1.89 - 1.86 (m, 2H), 1.70 - 1.62 (m, 3H), 1.59 - 1.51 (m, 3H), 1.38 - 1.29 (m, 2H).

PolyA mRNA Sequencing

1. RNA integrity was assessed using the Bioanalyzer 2100 system (Agilent Technologies, CA, USA).

2. Messenger RNA was purified from total RNA using poly-T oligo-attached magnetic beads. After fragmentation, the first strand cDNA was synthesized using random hexamer primers. Then the second strand cDNA was synthesized using dUTP, instead of dTTP. The directional library was ready after end repair, A-tailing, adapter ligation, size selection, amplification, and purification. The library was checked with Qubit and real-time PCR for quantification and bioanalyzer for size distribution detection.

3. After library quality control, different libraries were pooled based on the effective concentration and targeted data amount, then subjected to Illumina sequencing. The basic principle of sequencing is "Sequencing by Synthesis", where fluorescently labeled dNTPs, DNA polymerase,

and adapter primers are added to the sequencing flow cell for amplification. As each sequencing cluster extends its complementary strand, the addition of each fluorescently labeled dNTP releases a corresponding fluorescence signal. The sequencer captures these fluorescence signals and converts them into sequencing peaks through computer software, thereby obtaining the sequence information of the target fragment.

4. Raw data (raw reads) of fastq format were firstly processed through fastp software. In this step, clean data (clean reads) were obtained by removing reads containing adapter, reads containing ploy-N and low quality reads from raw data. At the same time, Q20, Q30 and GC content the clean data were calculated. All the downstream analyses were based on the clean data with high quality.

5. Reference genome and gene model annotation files were downloaded from genome website directly. Index of the reference genome was built 3 using Hisat2 v2.0.5 and paired-end clean 1 reads were aligned to the reference genome using Hisat2 v2.0.5.
