# Supplementary figures and images for: Small molecule disruption of RARα/NCoR1 interaction inhibits chaperone-mediated autophagy in cancer (part 1 of 2)
Source: EMBO Mol Med. 2025 Jun 9;17(7):1716–55. doi: 10.1038/s44321-025-00254-y (PMC12254369; doi:10.1038/s44321-025-00254-y)

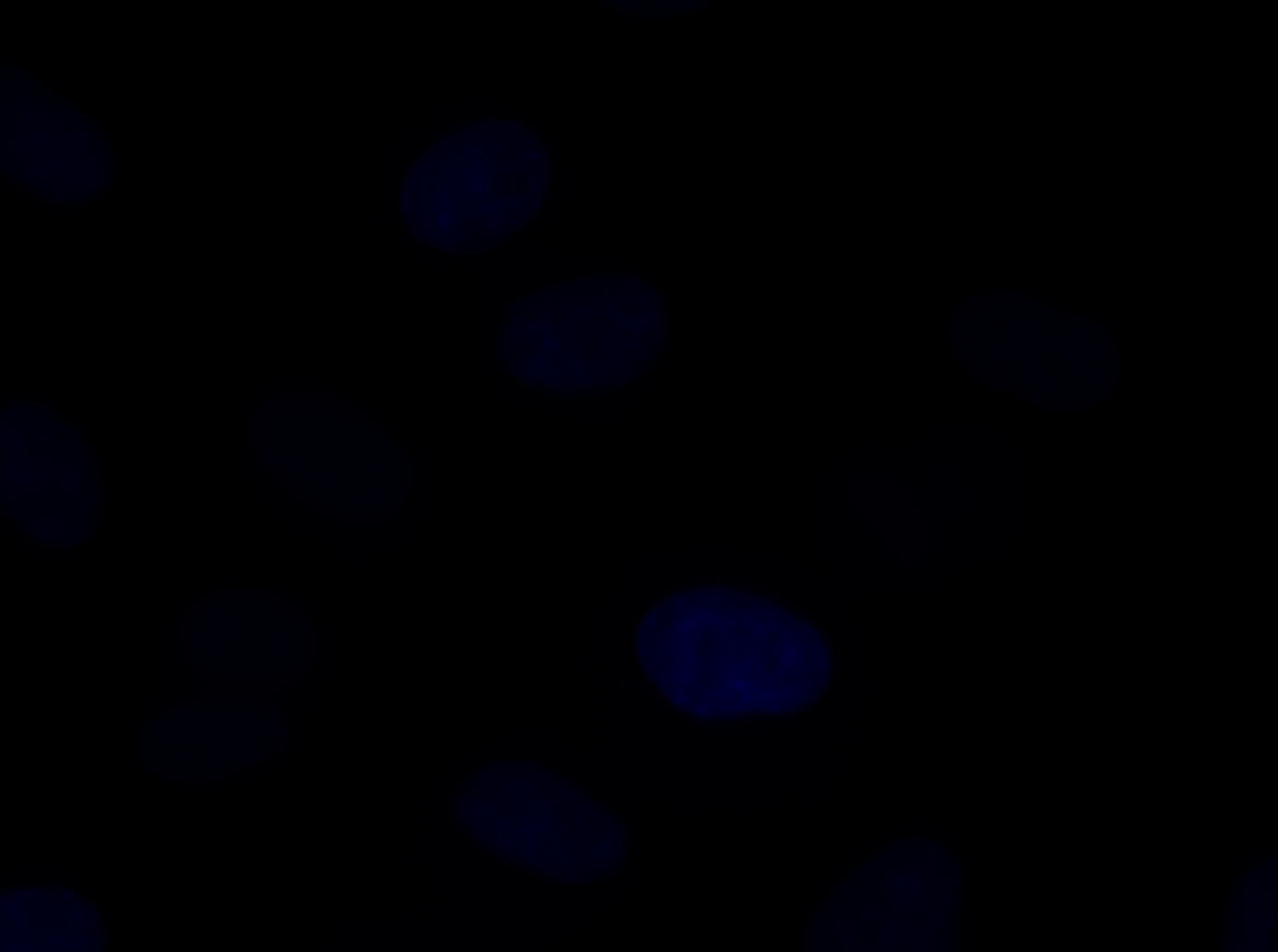

Supplement: Supplementary file 3 — Source data Fig. 1 [file 44321_2025_254_MOESM3_ESM.zip › Figure 1/Fig1A/A549_DAPI.TIF]

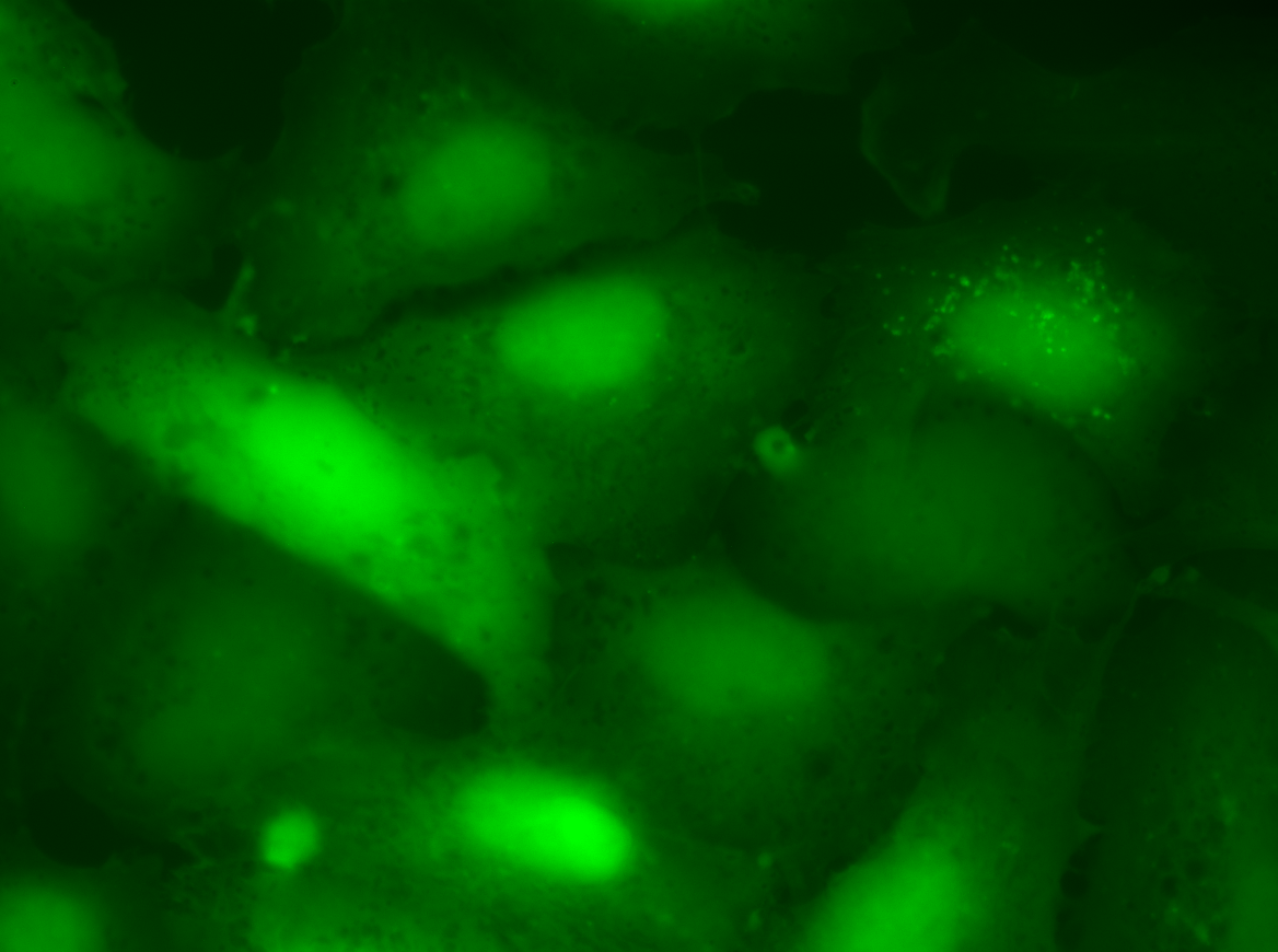

Supplement: Supplementary file 3 — Source data Fig. 1 [file 44321_2025_254_MOESM3_ESM.zip › Figure 1/Fig1A/A549_FITC.TIF]

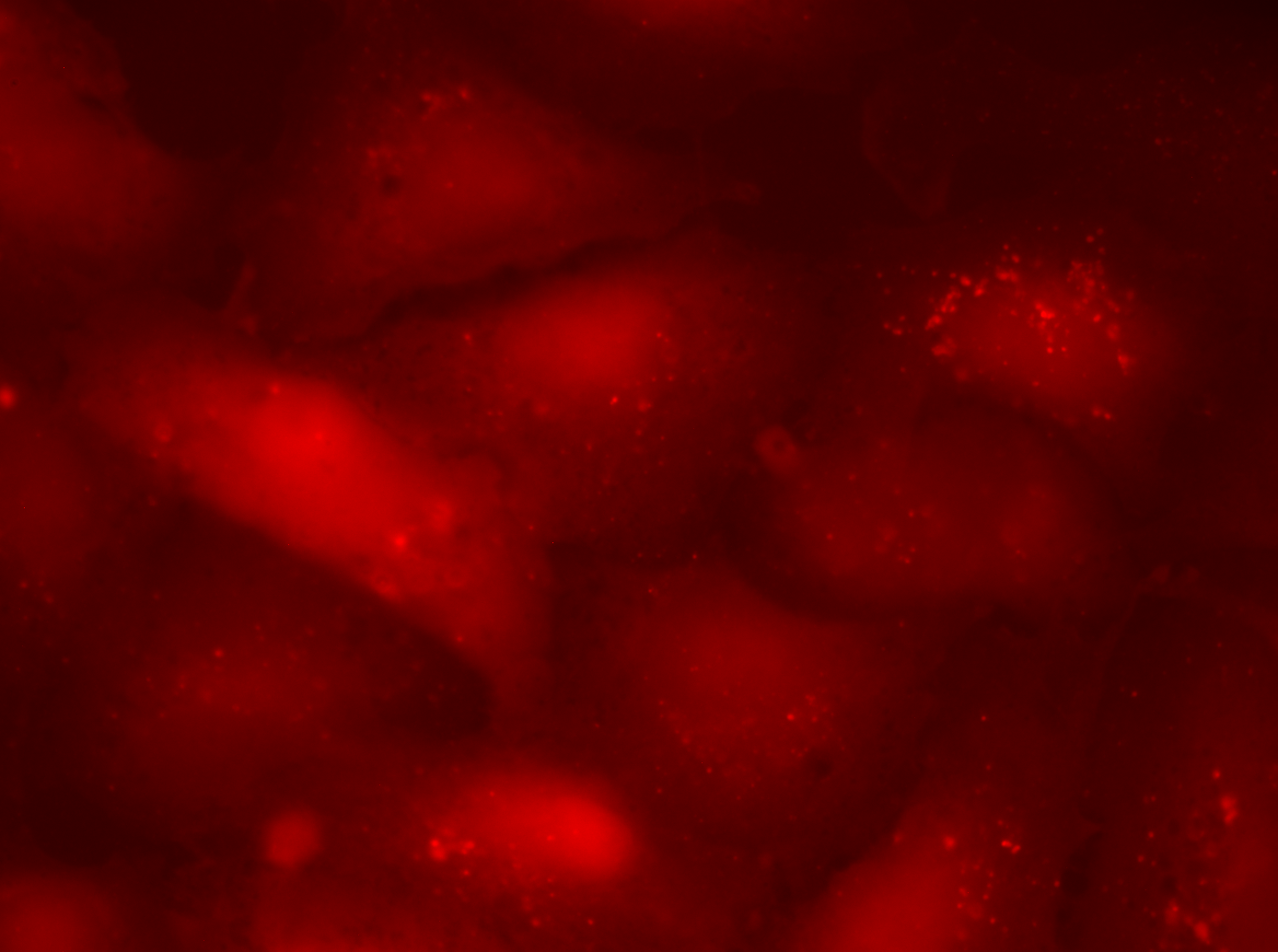

Supplement: Supplementary file 3 — Source data Fig. 1 [file 44321_2025_254_MOESM3_ESM.zip › Figure 1/Fig1A/A549_Rhodamine.TIF]

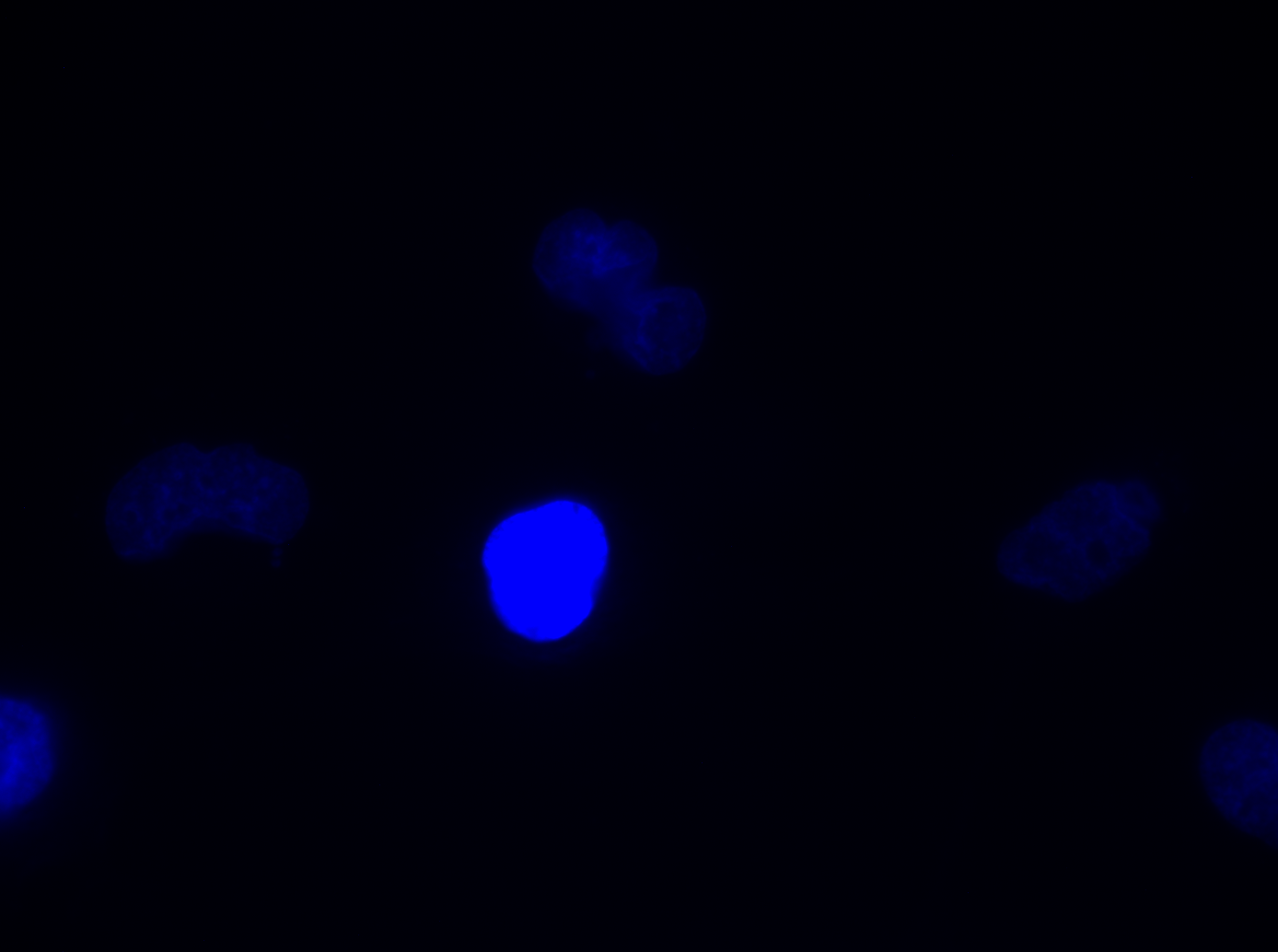

Supplement: Supplementary file 3 — Source data Fig. 1 [file 44321_2025_254_MOESM3_ESM.zip › Figure 1/Fig1A/BEAS2B_DAPI.TIF]

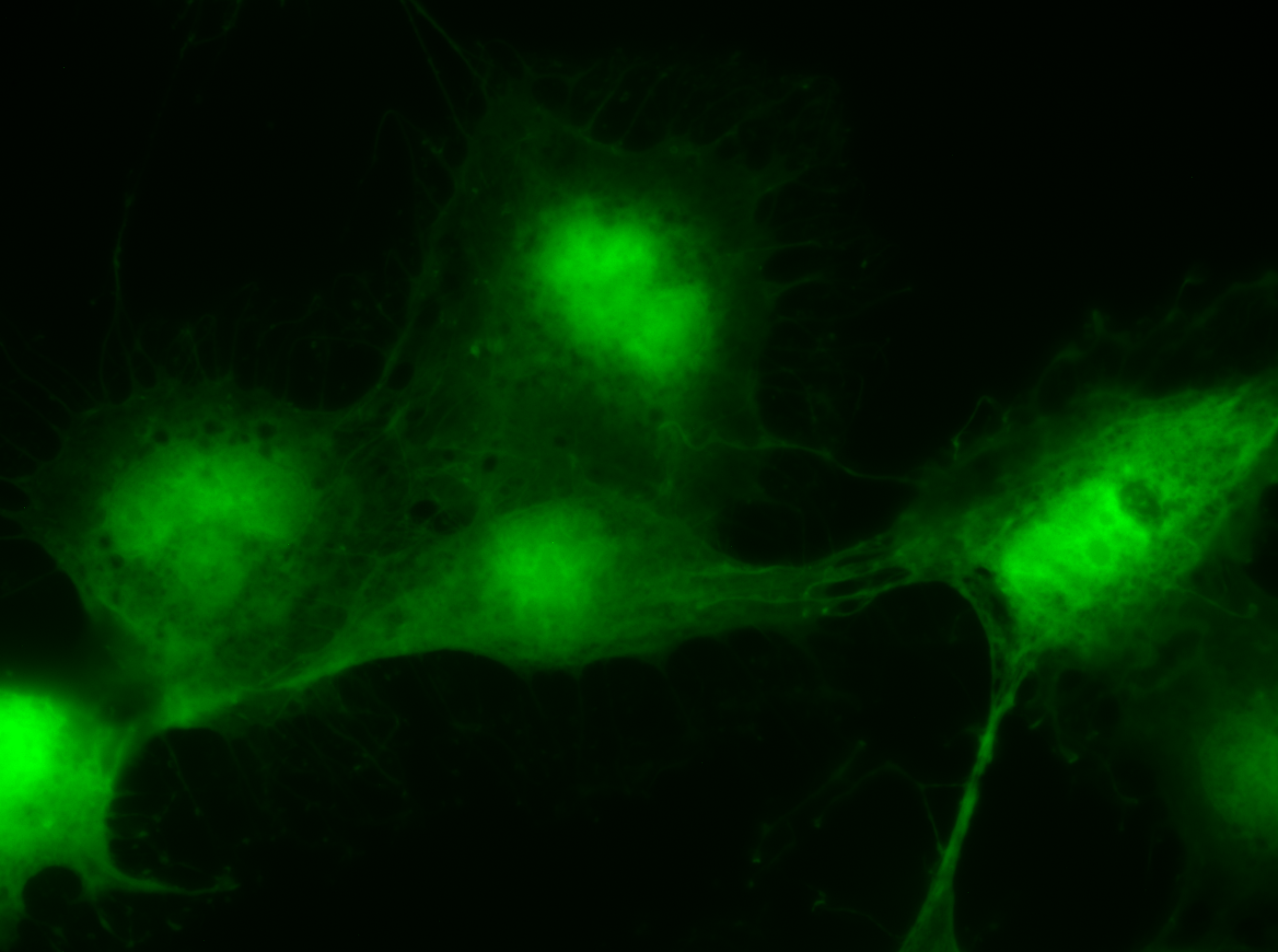

Supplement: Supplementary file 3 — Source data Fig. 1 [file 44321_2025_254_MOESM3_ESM.zip › Figure 1/Fig1A/BEAS2B_FITC.TIF]

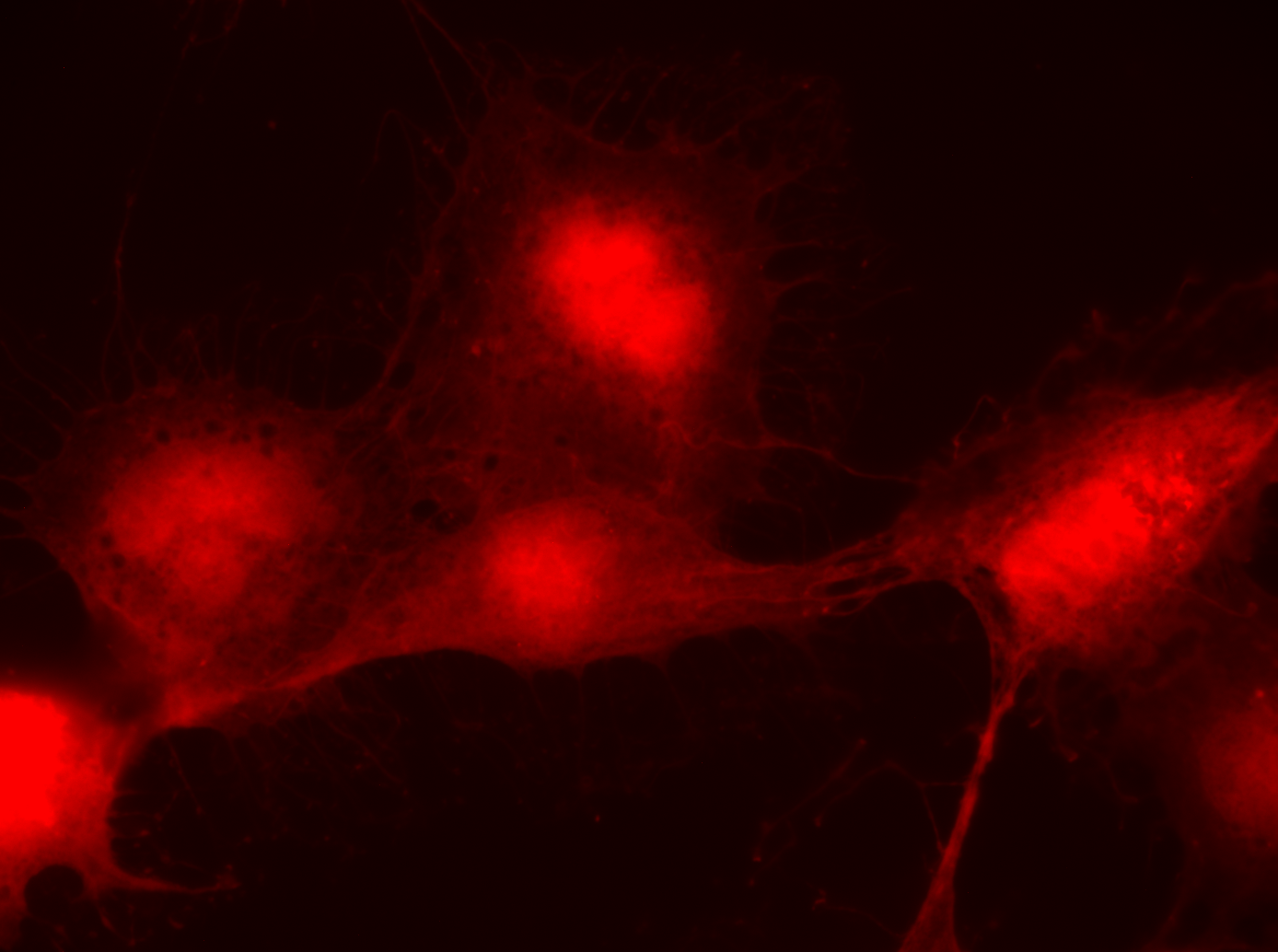

Supplement: Supplementary file 3 — Source data Fig. 1 [file 44321_2025_254_MOESM3_ESM.zip › Figure 1/Fig1A/BEAS2B_Rhodamine.TIF]

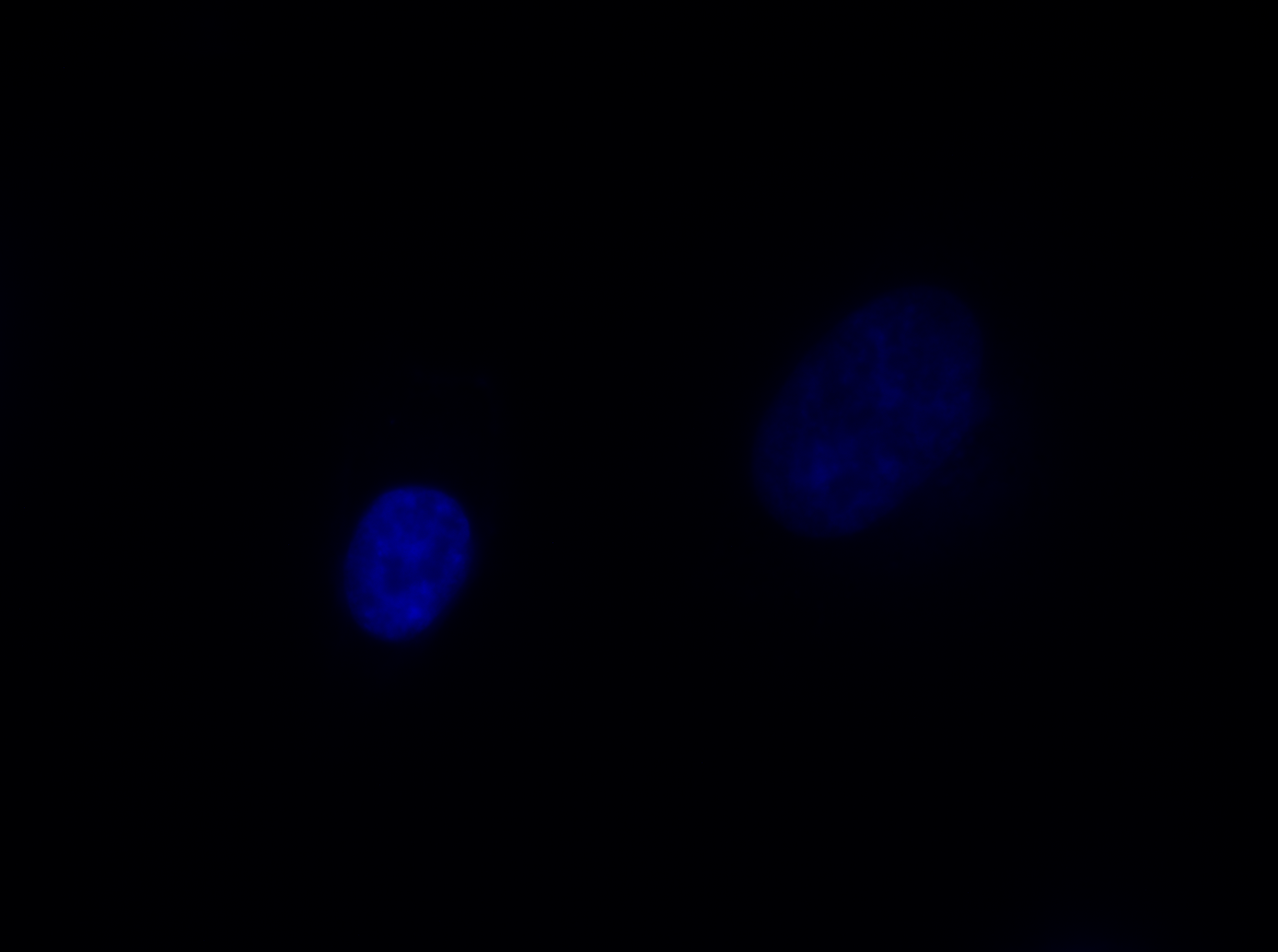

Supplement: Supplementary file 3 — Source data Fig. 1 [file 44321_2025_254_MOESM3_ESM.zip › Figure 1/Fig1A/H1703_DAPI.TIF]

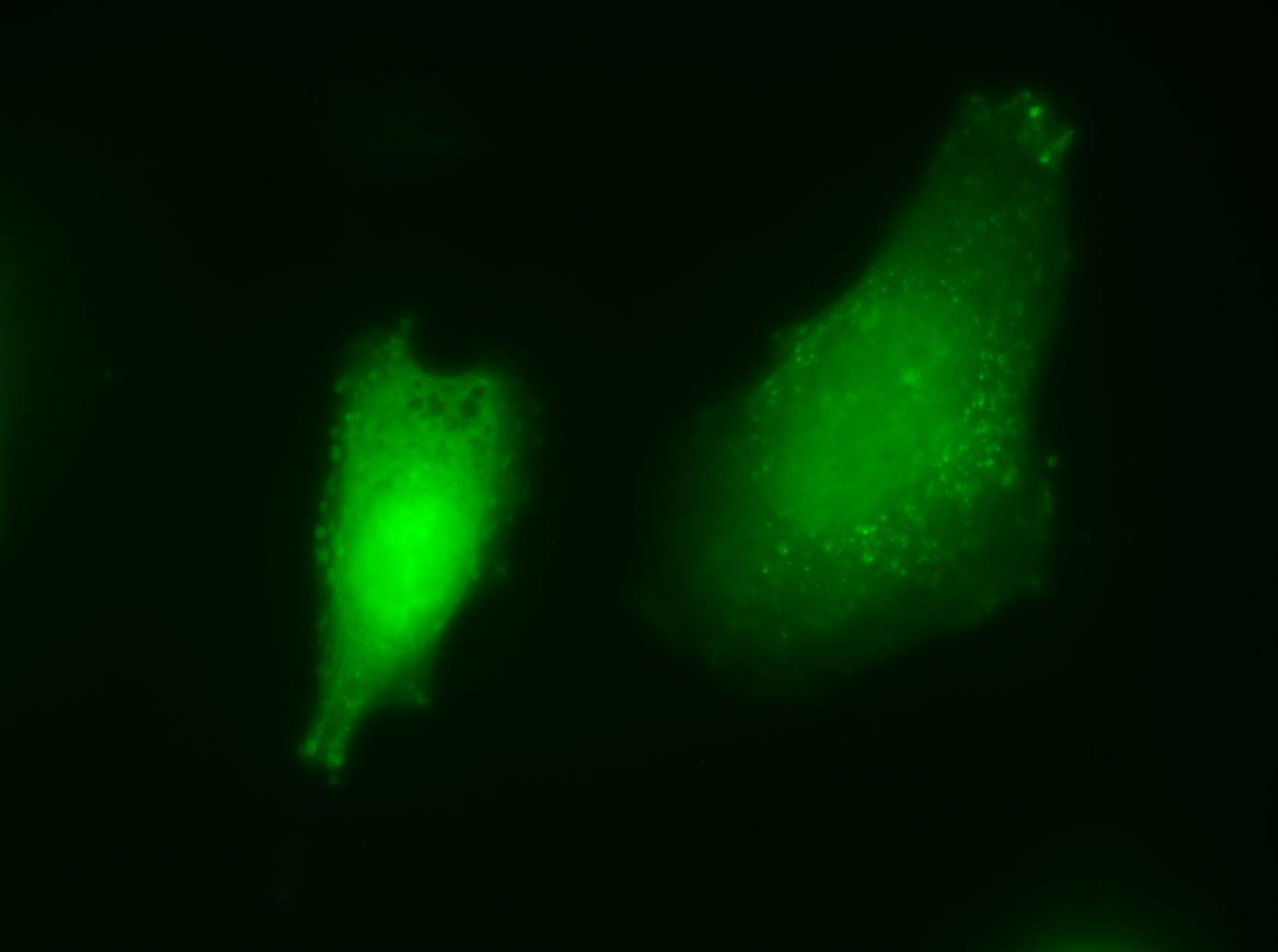

Supplement: Supplementary file 3 — Source data Fig. 1 [file 44321_2025_254_MOESM3_ESM.zip › Figure 1/Fig1A/H1703_FITC.TIF]

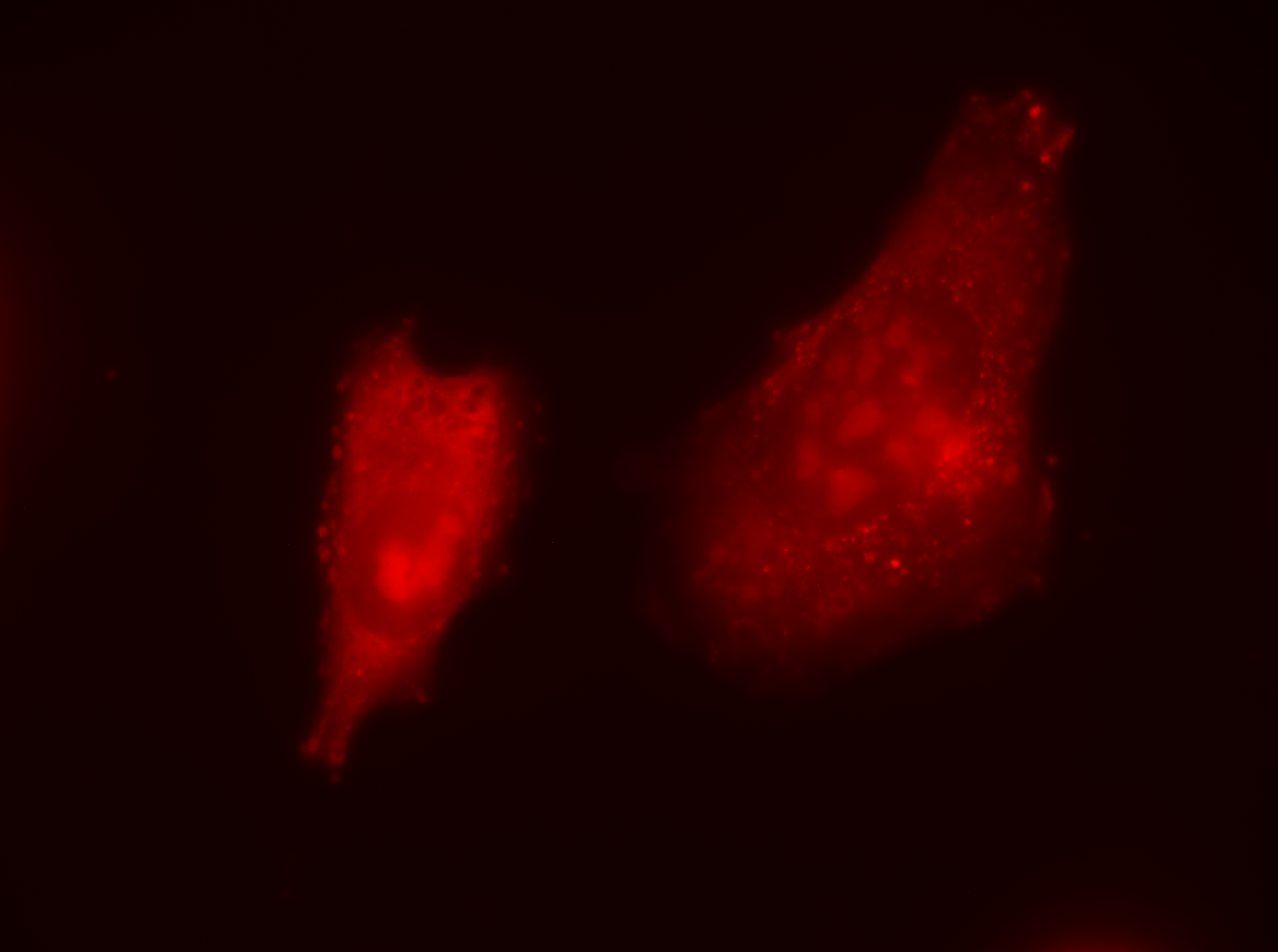

Supplement: Supplementary file 3 — Source data Fig. 1 [file 44321_2025_254_MOESM3_ESM.zip › Figure 1/Fig1A/H1703_Rhodamine.TIF]

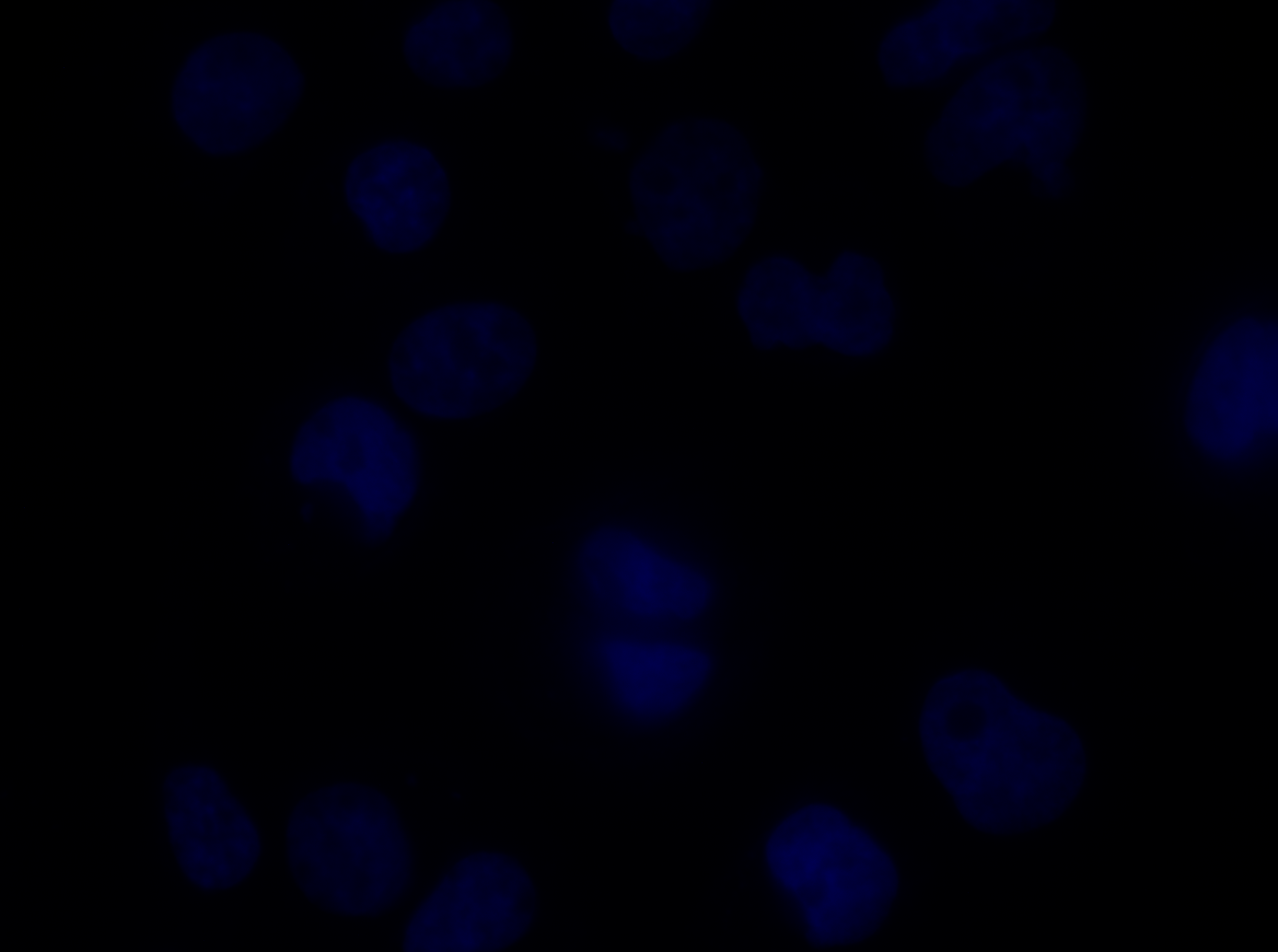

Supplement: Supplementary file 3 — Source data Fig. 1 [file 44321_2025_254_MOESM3_ESM.zip › Figure 1/Fig1A/H23_DAPI.TIF]

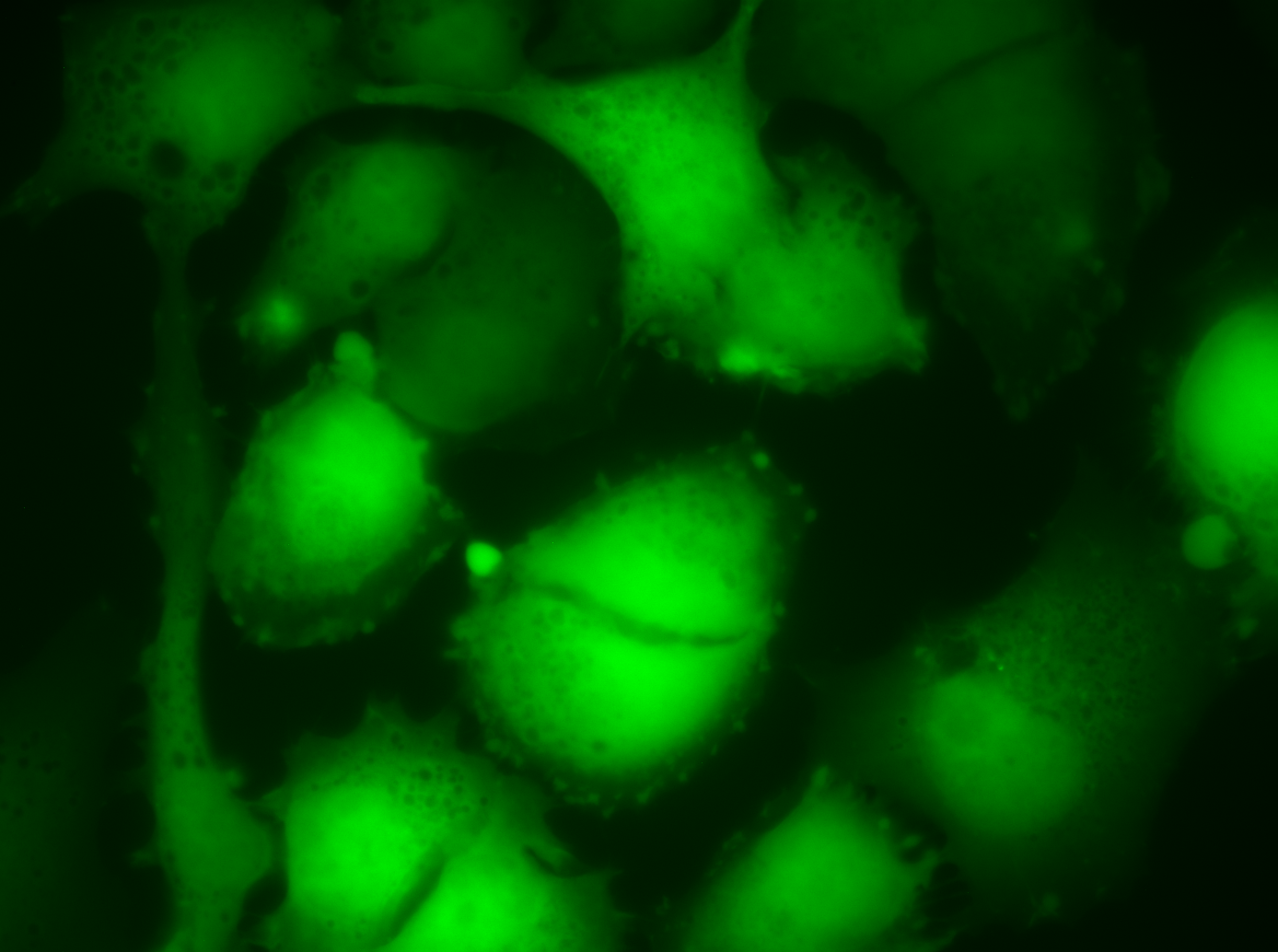

Supplement: Supplementary file 3 — Source data Fig. 1 [file 44321_2025_254_MOESM3_ESM.zip › Figure 1/Fig1A/H23_FITC.TIF]

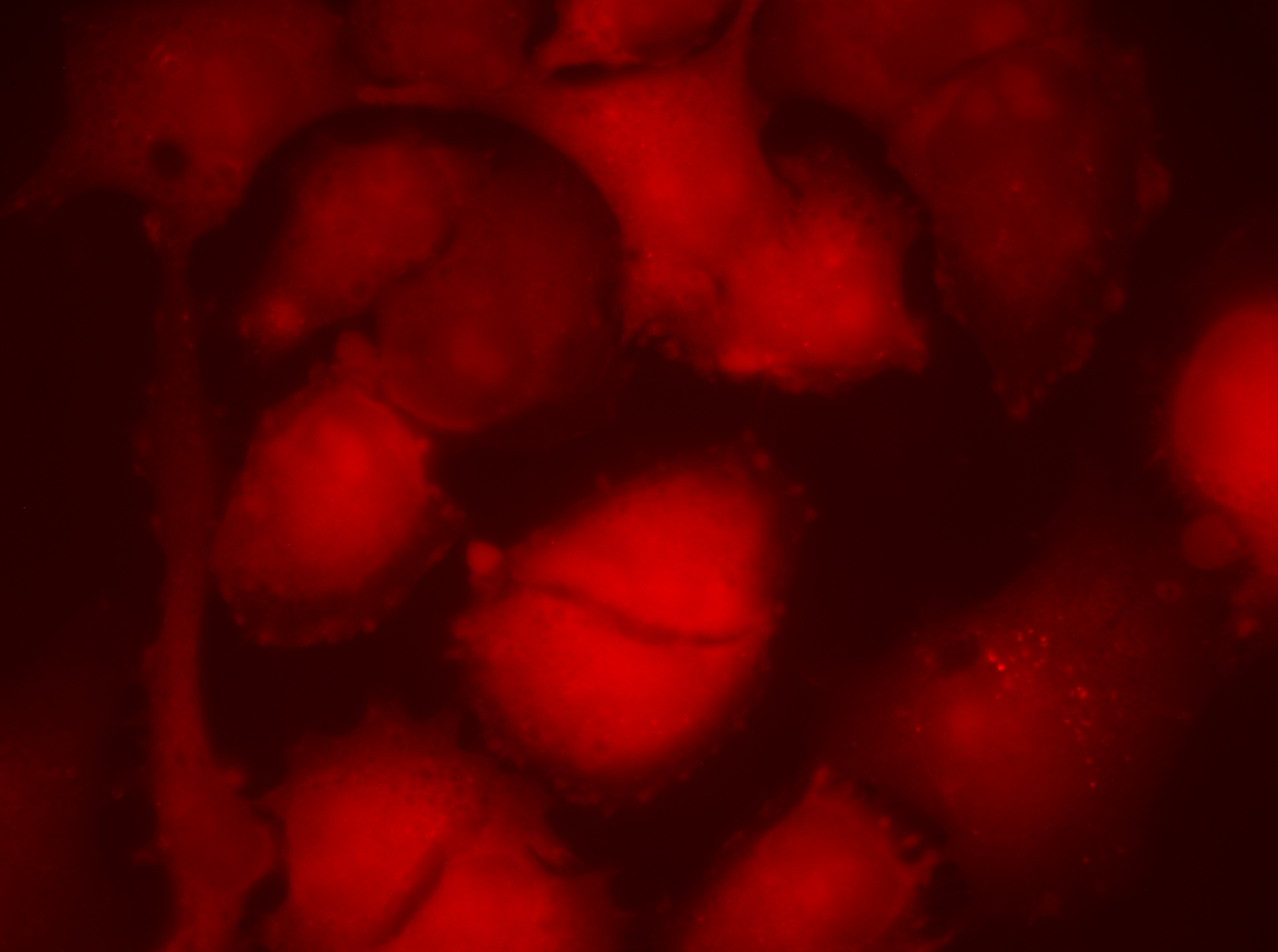

Supplement: Supplementary file 3 — Source data Fig. 1 [file 44321_2025_254_MOESM3_ESM.zip › Figure 1/Fig1A/H23_Rhodamine.TIF]

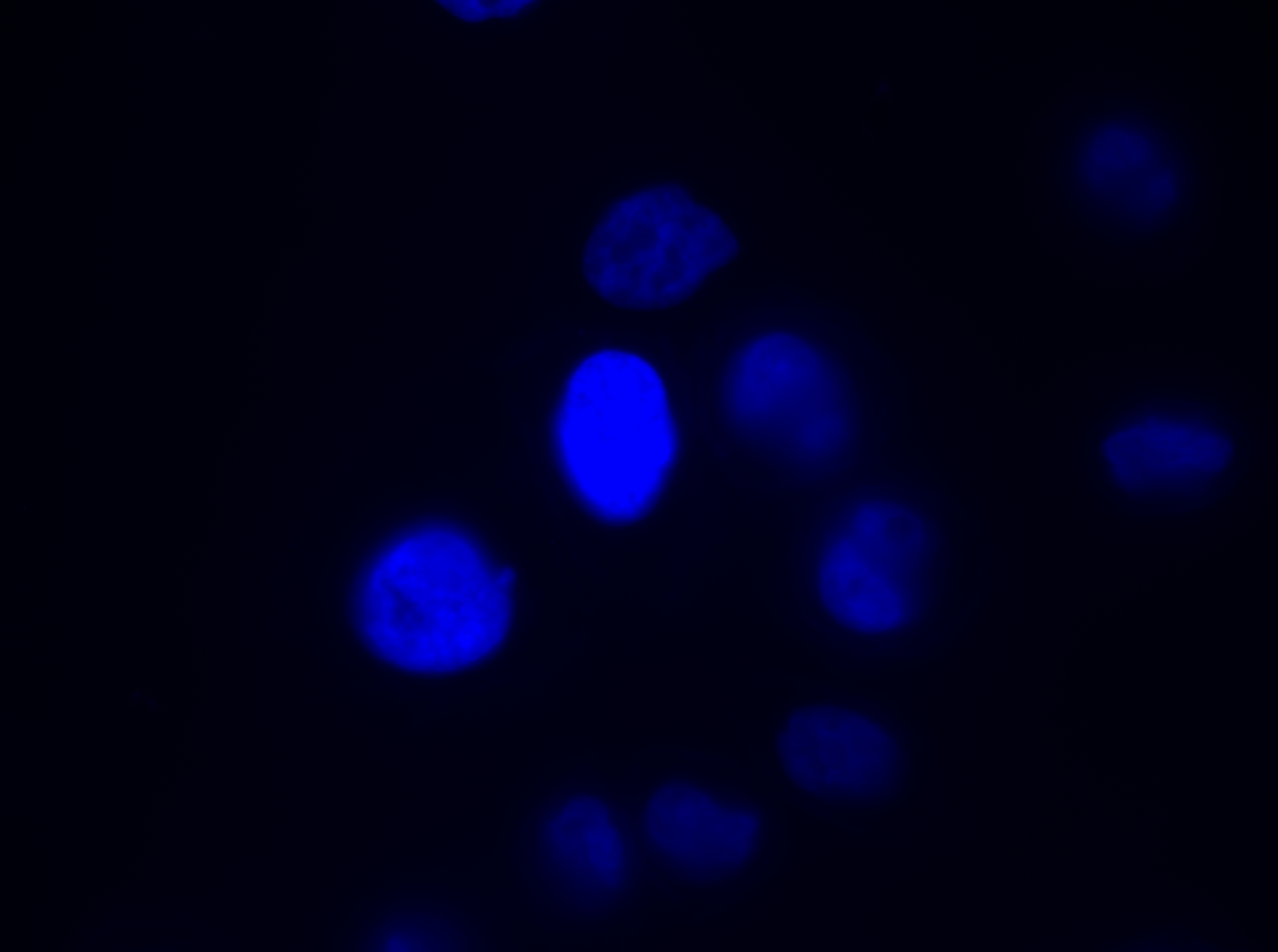

Supplement: Supplementary file 3 — Source data Fig. 1 [file 44321_2025_254_MOESM3_ESM.zip › Figure 1/Fig1A/H460_DAPI.TIF]

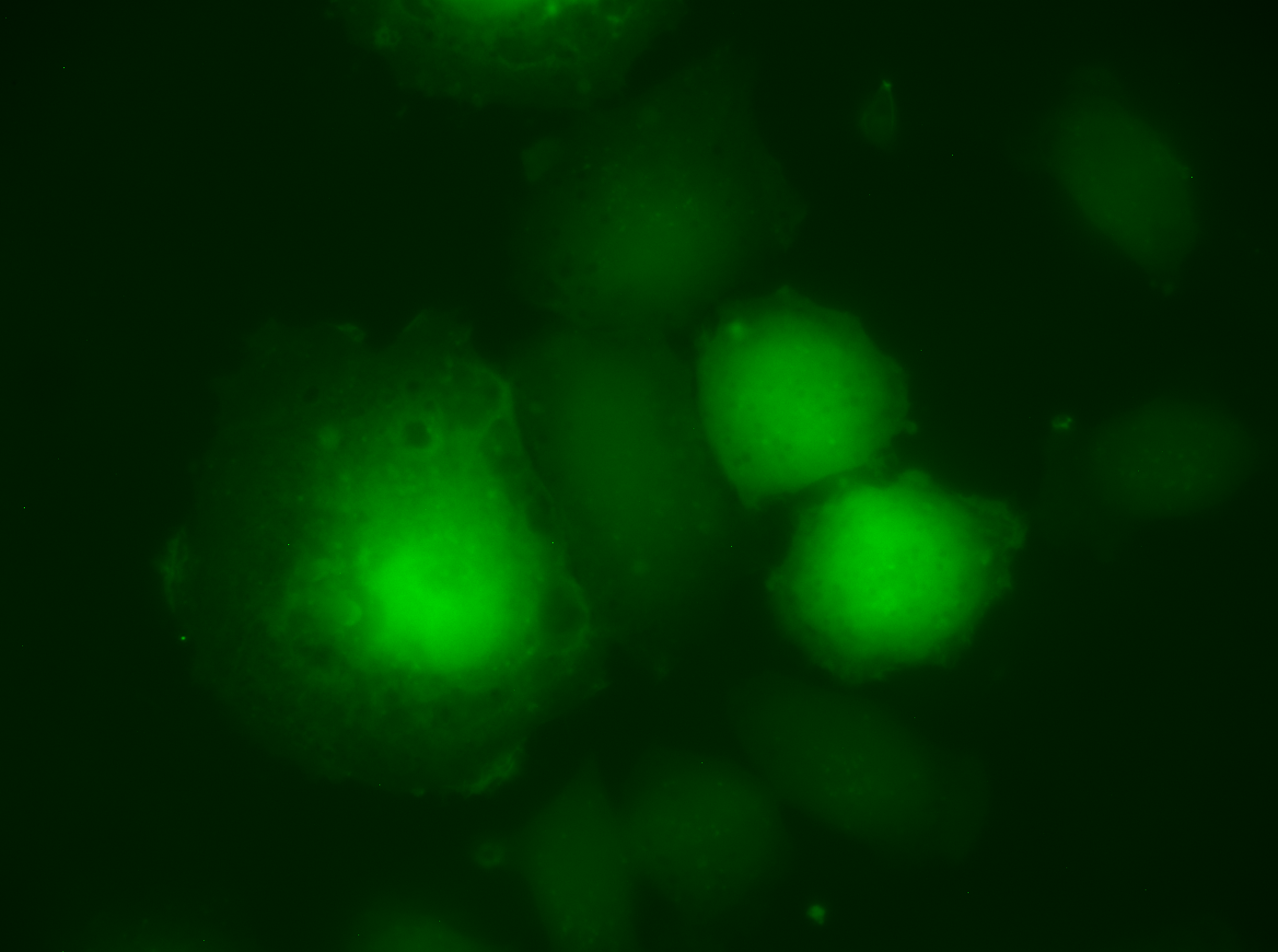

Supplement: Supplementary file 3 — Source data Fig. 1 [file 44321_2025_254_MOESM3_ESM.zip › Figure 1/Fig1A/H460_FITC.TIF]

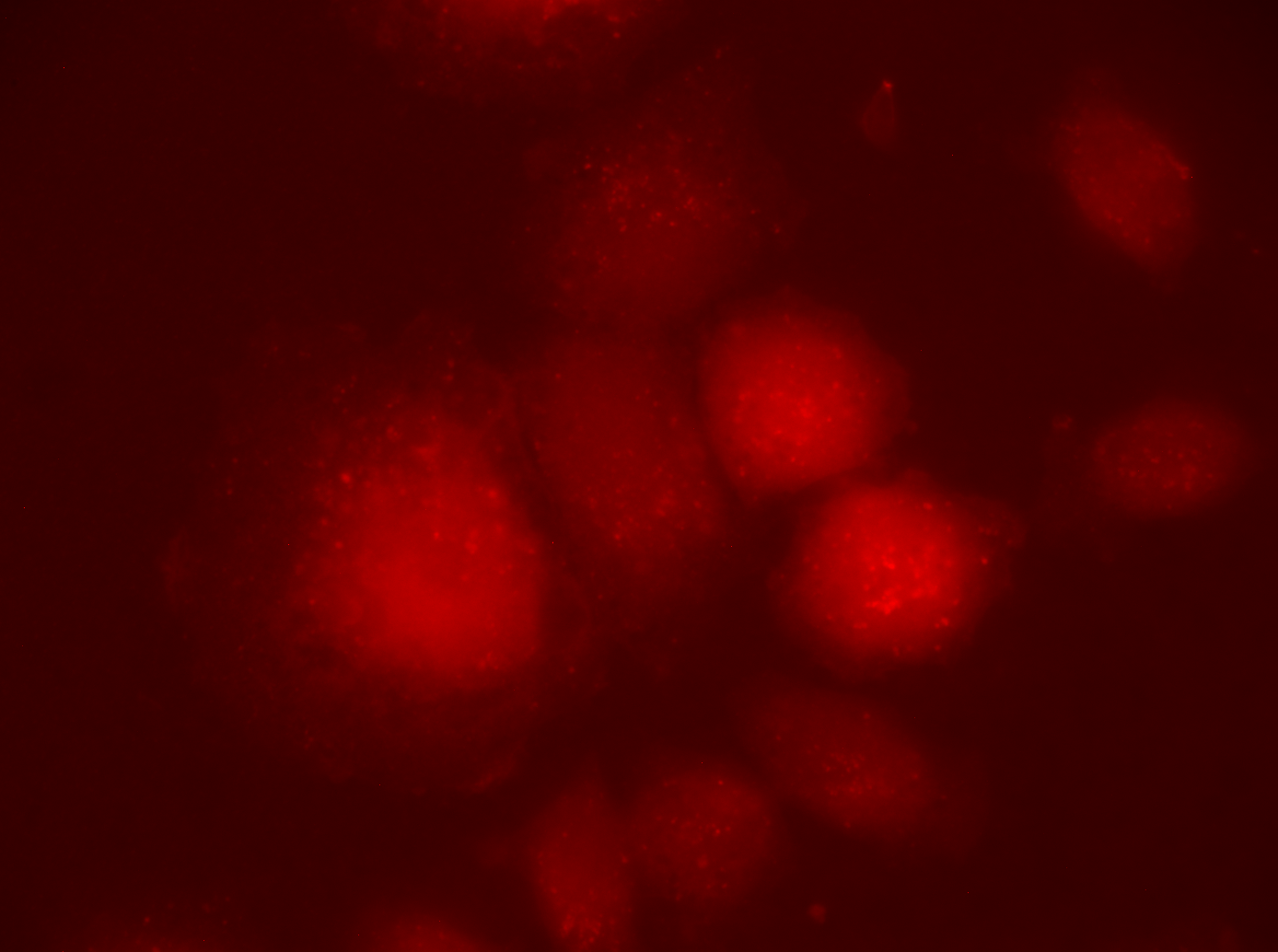

Supplement: Supplementary file 3 — Source data Fig. 1 [file 44321_2025_254_MOESM3_ESM.zip › Figure 1/Fig1A/H460_Rhodamine.TIF]

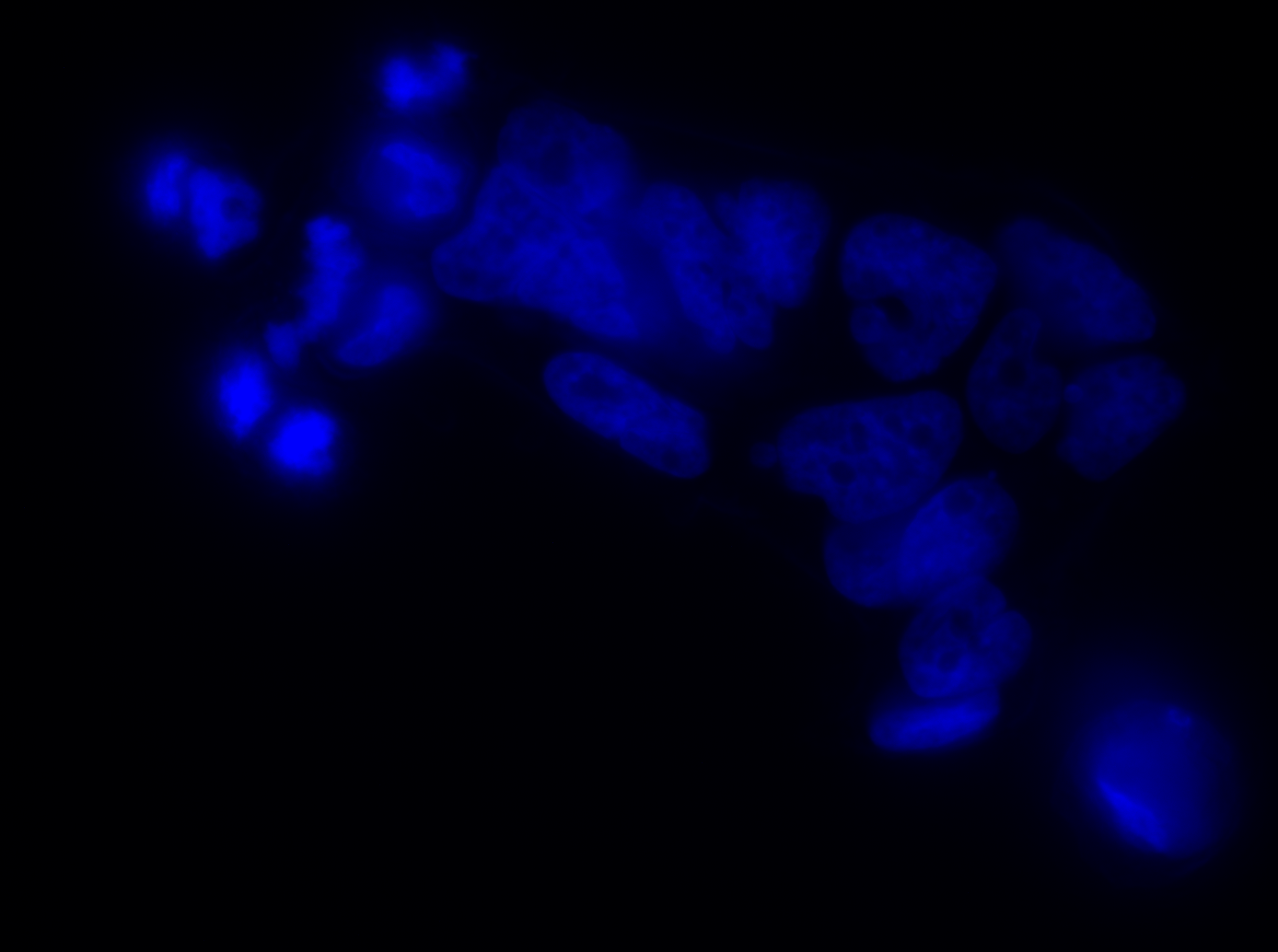

Supplement: Supplementary file 3 — Source data Fig. 1 [file 44321_2025_254_MOESM3_ESM.zip › Figure 1/Fig1A/H520_DAPI.TIF]

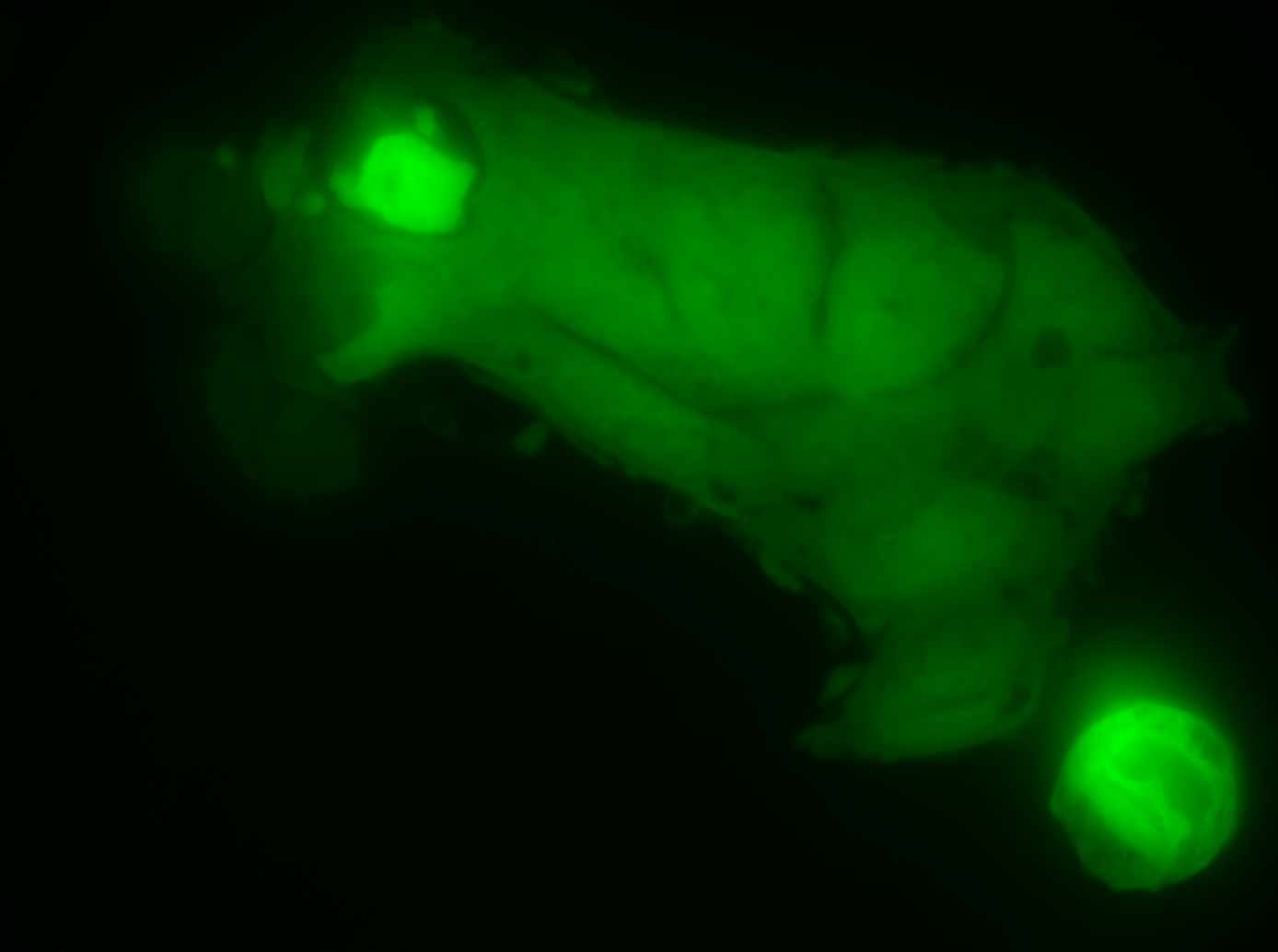

Supplement: Supplementary file 3 — Source data Fig. 1 [file 44321_2025_254_MOESM3_ESM.zip › Figure 1/Fig1A/H520_FITC.TIF]

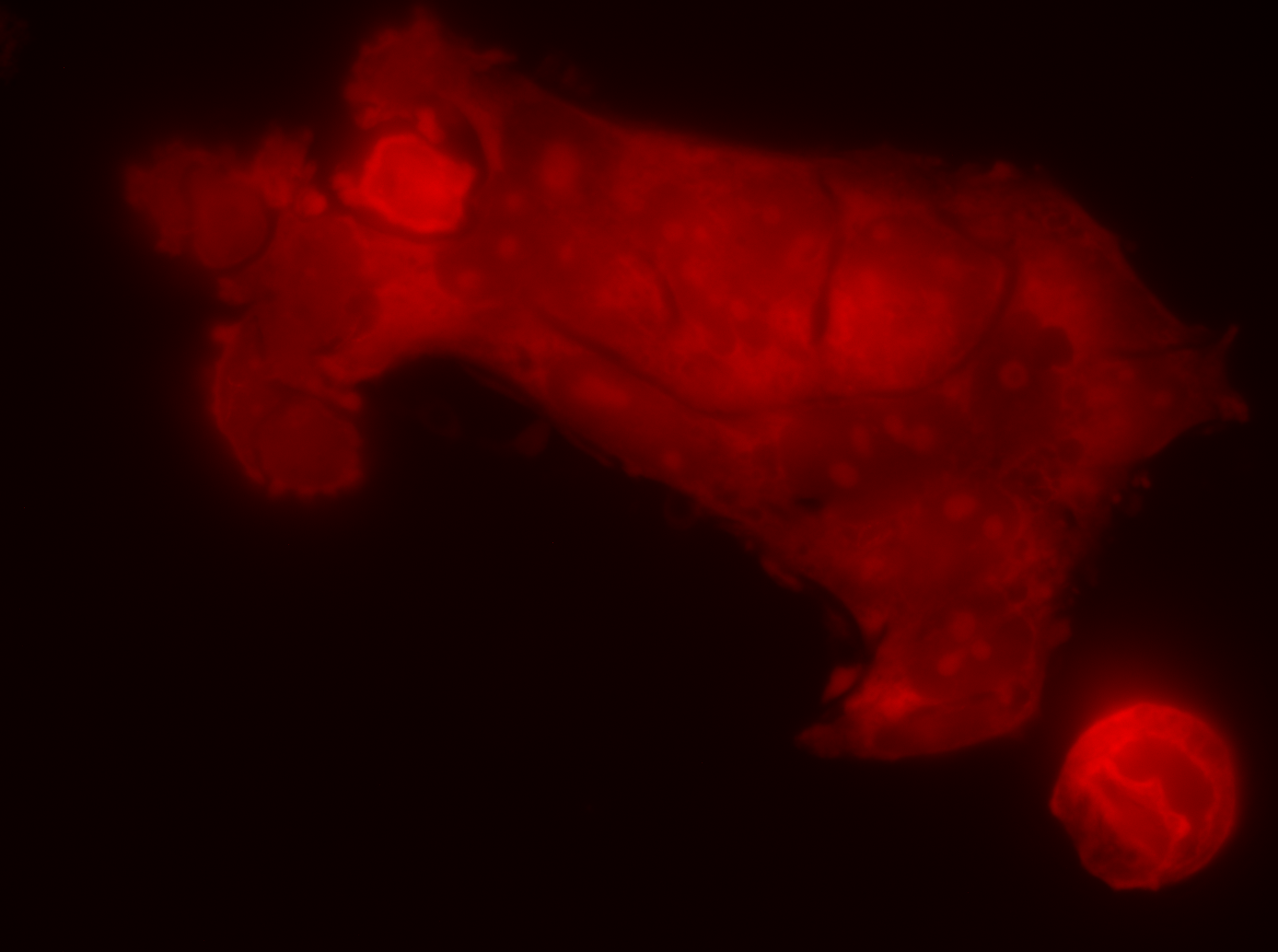

Supplement: Supplementary file 3 — Source data Fig. 1 [file 44321_2025_254_MOESM3_ESM.zip › Figure 1/Fig1A/H520_Rhodamine.TIF]

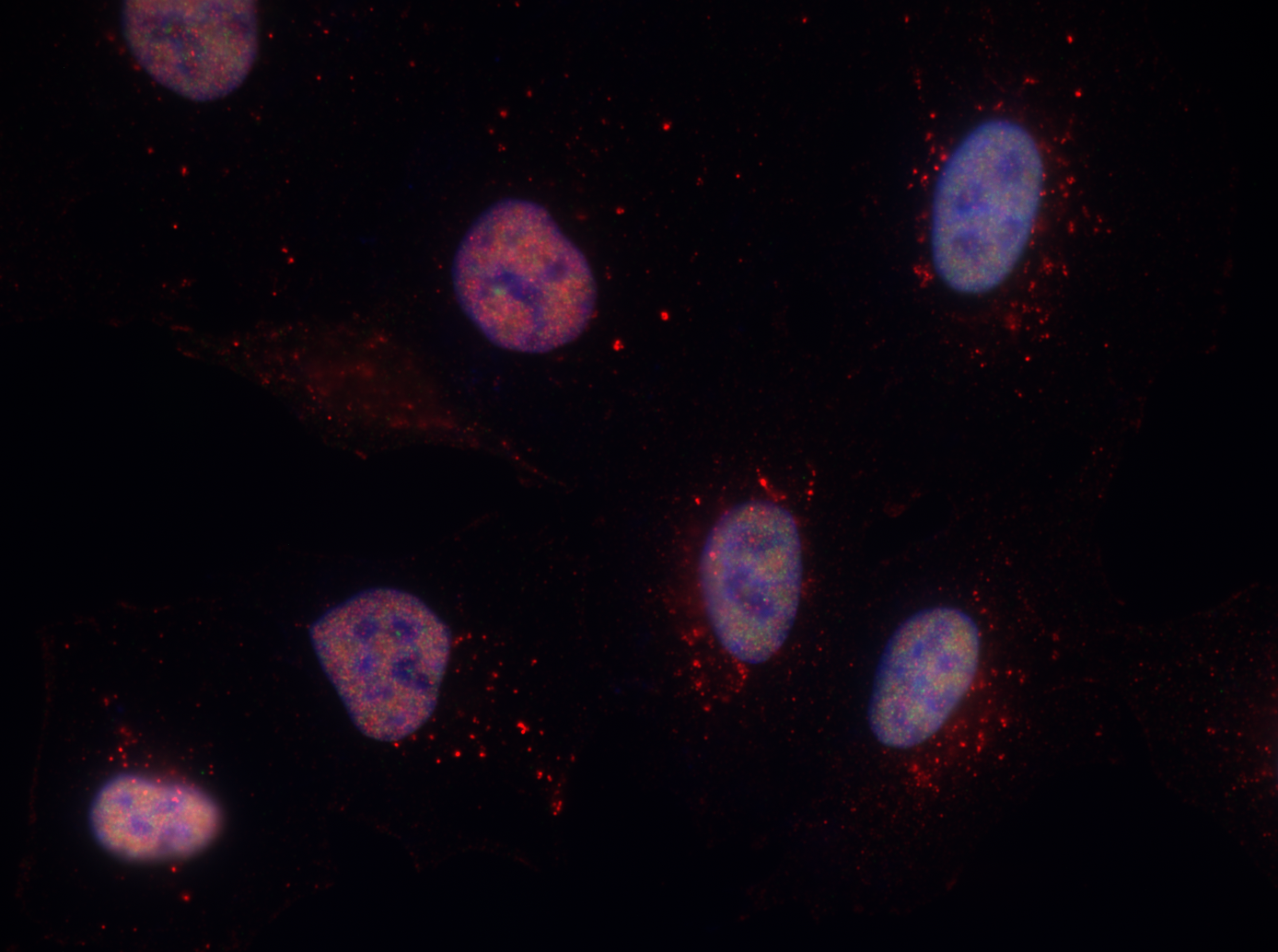

Supplement: Supplementary file 3 — Source data Fig. 1 [file 44321_2025_254_MOESM3_ESM.zip › Figure 1/Fig1C/A549_(DAPI+FITC+Rhodamine).TIF]

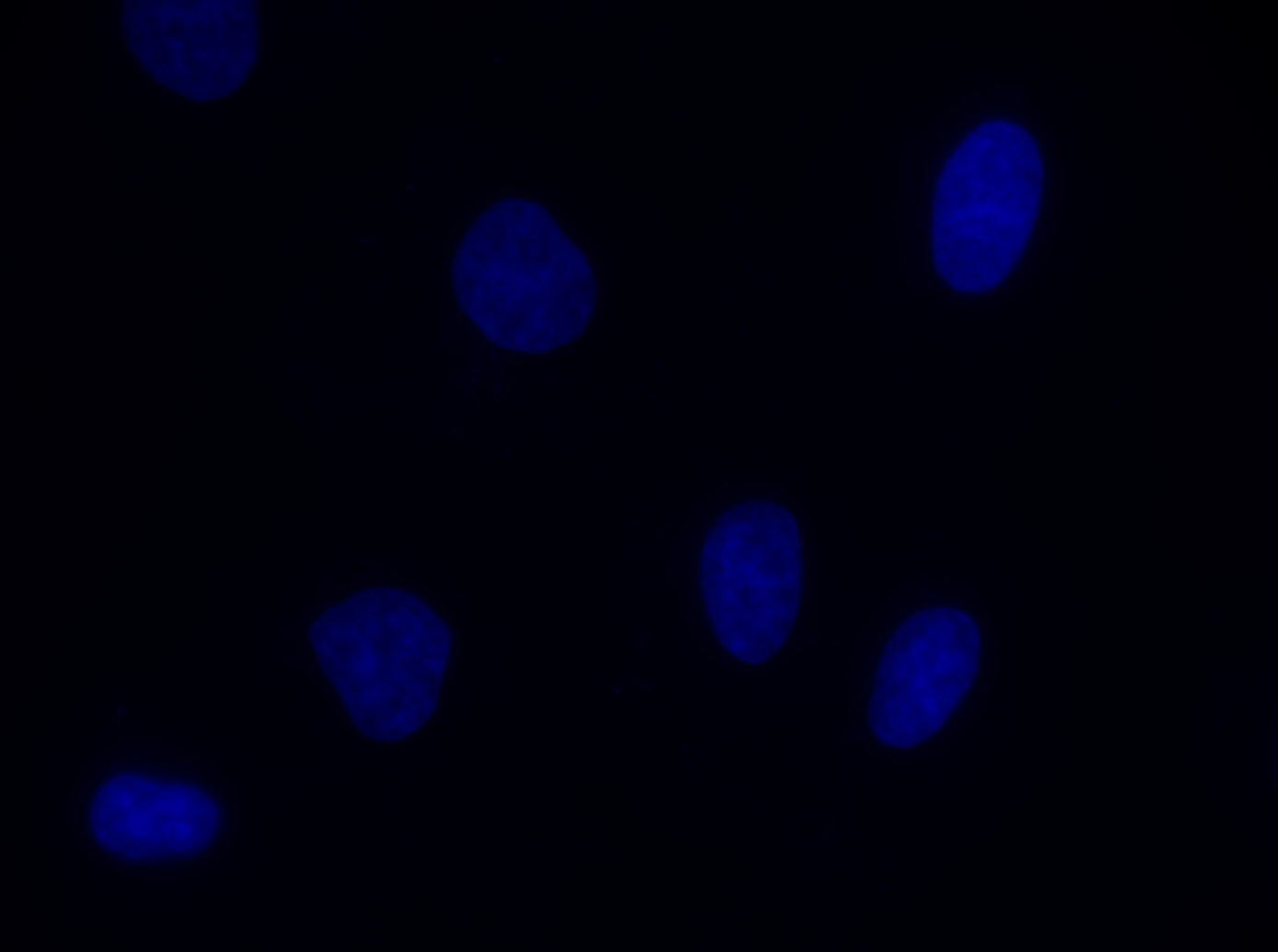

Supplement: Supplementary file 3 — Source data Fig. 1 [file 44321_2025_254_MOESM3_ESM.zip › Figure 1/Fig1C/A549_DAPI.TIF]

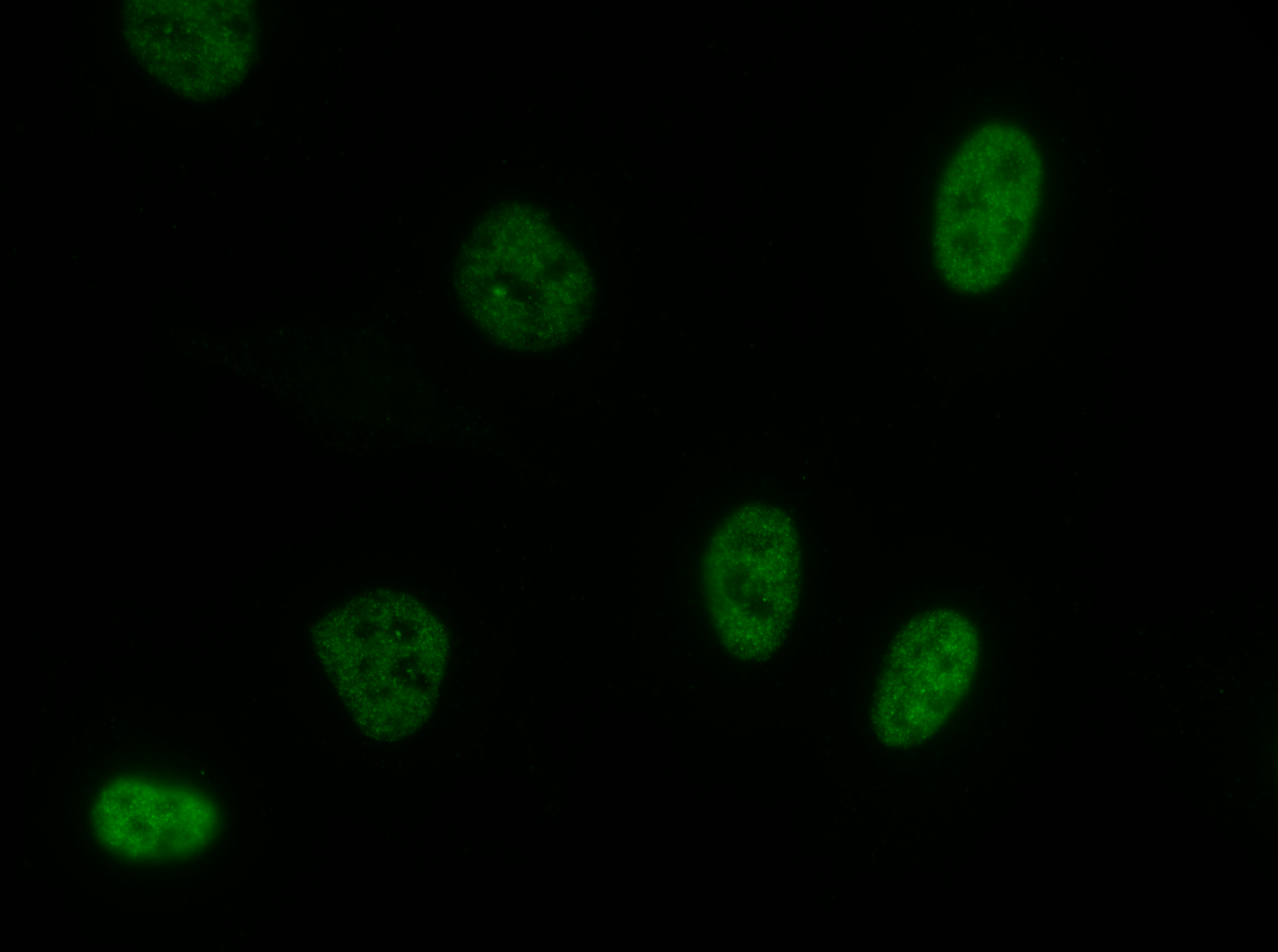

Supplement: Supplementary file 3 — Source data Fig. 1 [file 44321_2025_254_MOESM3_ESM.zip › Figure 1/Fig1C/A549_FITC.TIF]

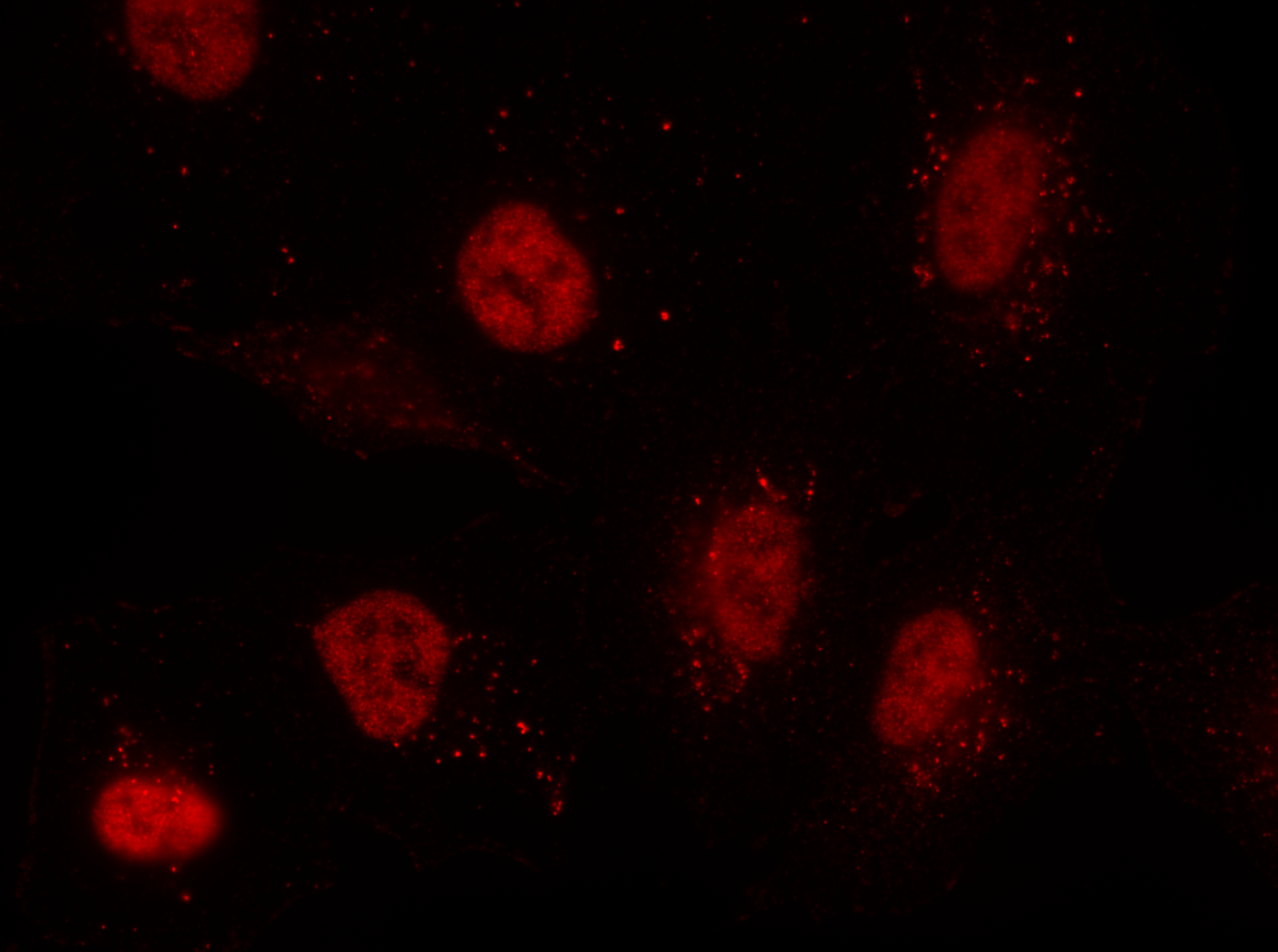

Supplement: Supplementary file 3 — Source data Fig. 1 [file 44321_2025_254_MOESM3_ESM.zip › Figure 1/Fig1C/A549_Rhodamine.TIF]

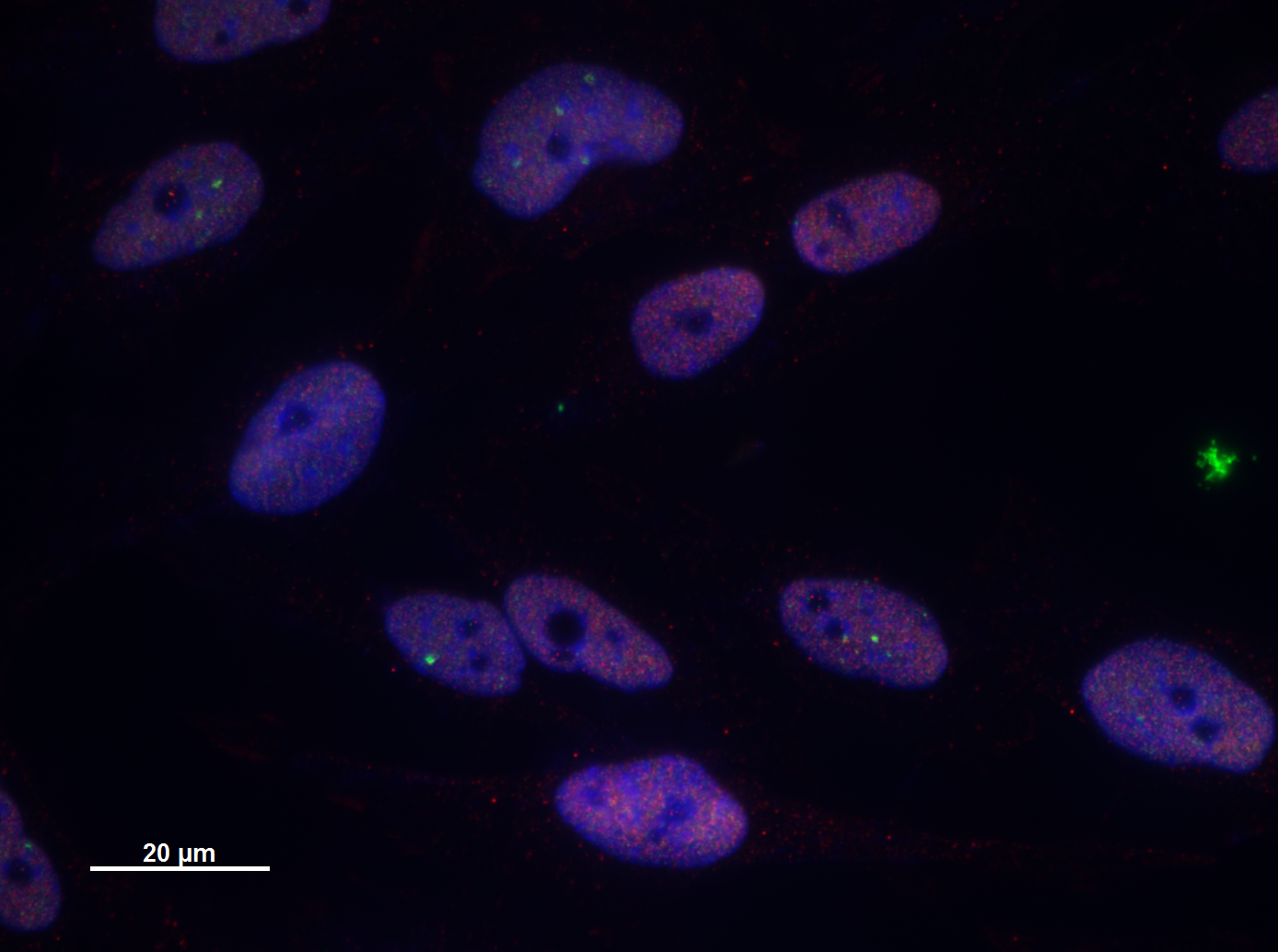

Supplement: Supplementary file 3 — Source data Fig. 1 [file 44321_2025_254_MOESM3_ESM.zip › Figure 1/Fig1C/BEAS2B_(DAPI+FITC+Rhodamine).TIF]

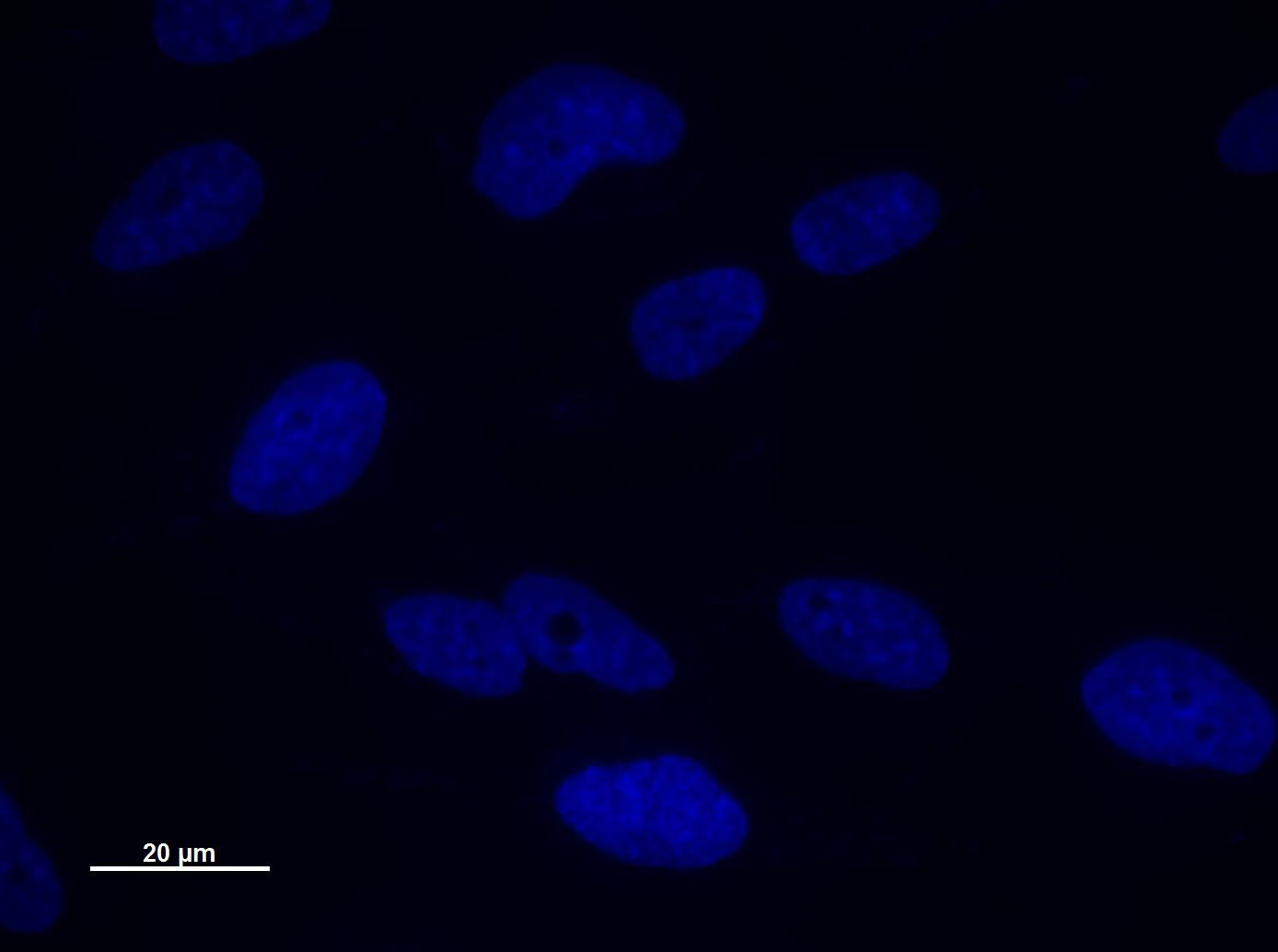

Supplement: Supplementary file 3 — Source data Fig. 1 [file 44321_2025_254_MOESM3_ESM.zip › Figure 1/Fig1C/BEAS2B_DAPI.TIF]

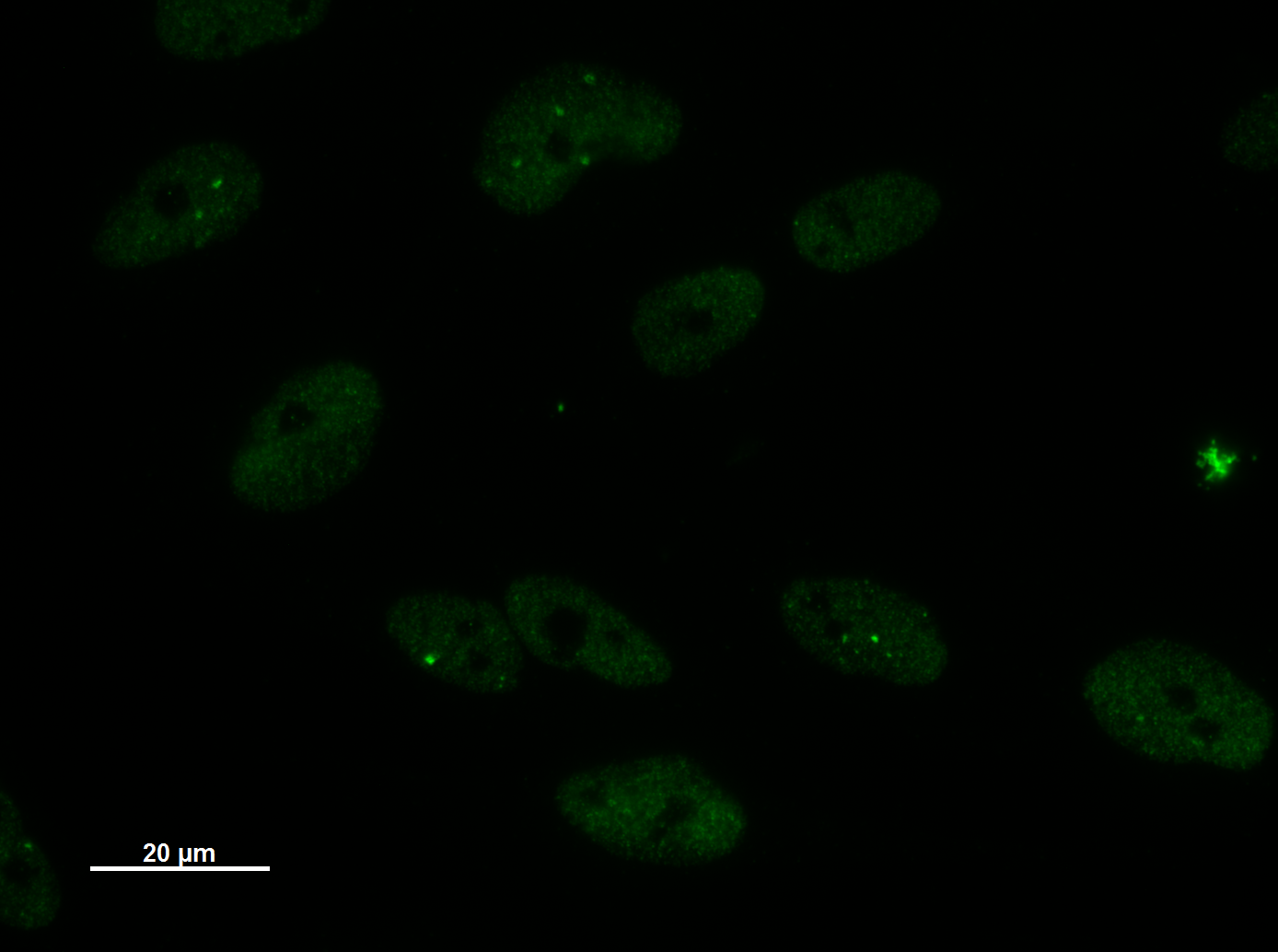

Supplement: Supplementary file 3 — Source data Fig. 1 [file 44321_2025_254_MOESM3_ESM.zip › Figure 1/Fig1C/BEAS2B_FITC.TIF]

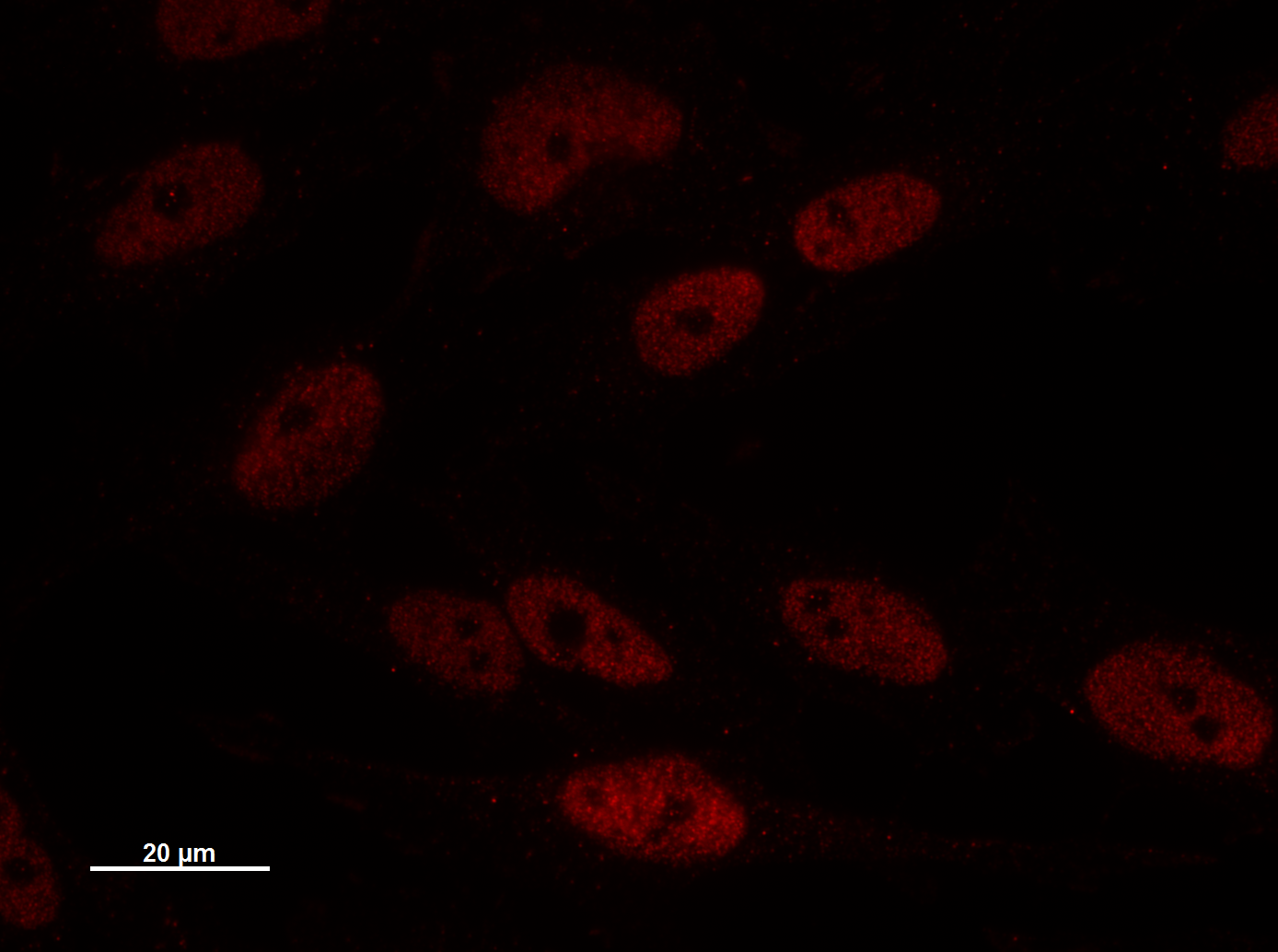

Supplement: Supplementary file 3 — Source data Fig. 1 [file 44321_2025_254_MOESM3_ESM.zip › Figure 1/Fig1C/BEAS2B_Rhodamine.TIF]

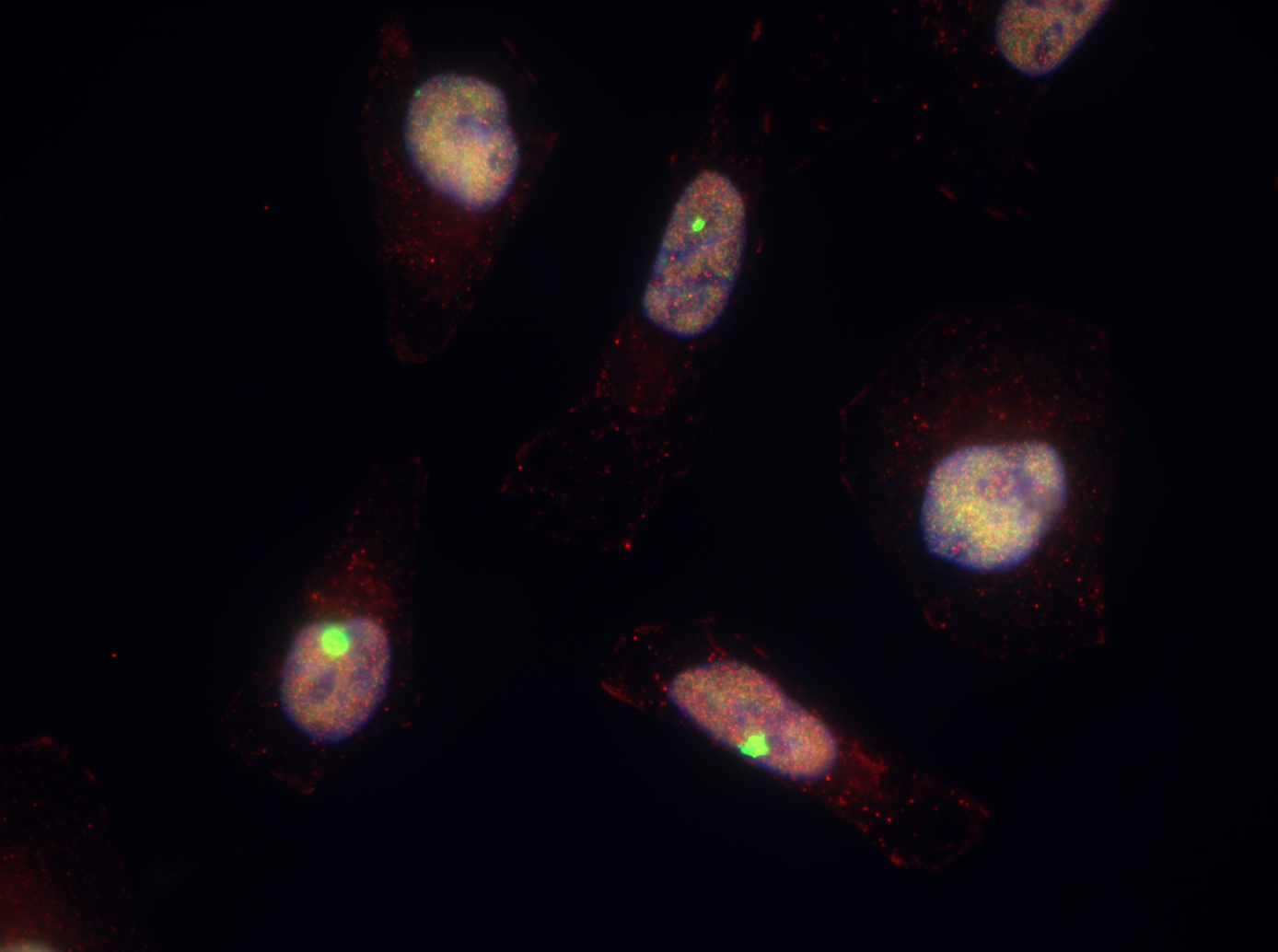

Supplement: Supplementary file 3 — Source data Fig. 1 [file 44321_2025_254_MOESM3_ESM.zip › Figure 1/Fig1C/H1703_(DAPI+FITC+Rhodamine).TIF]

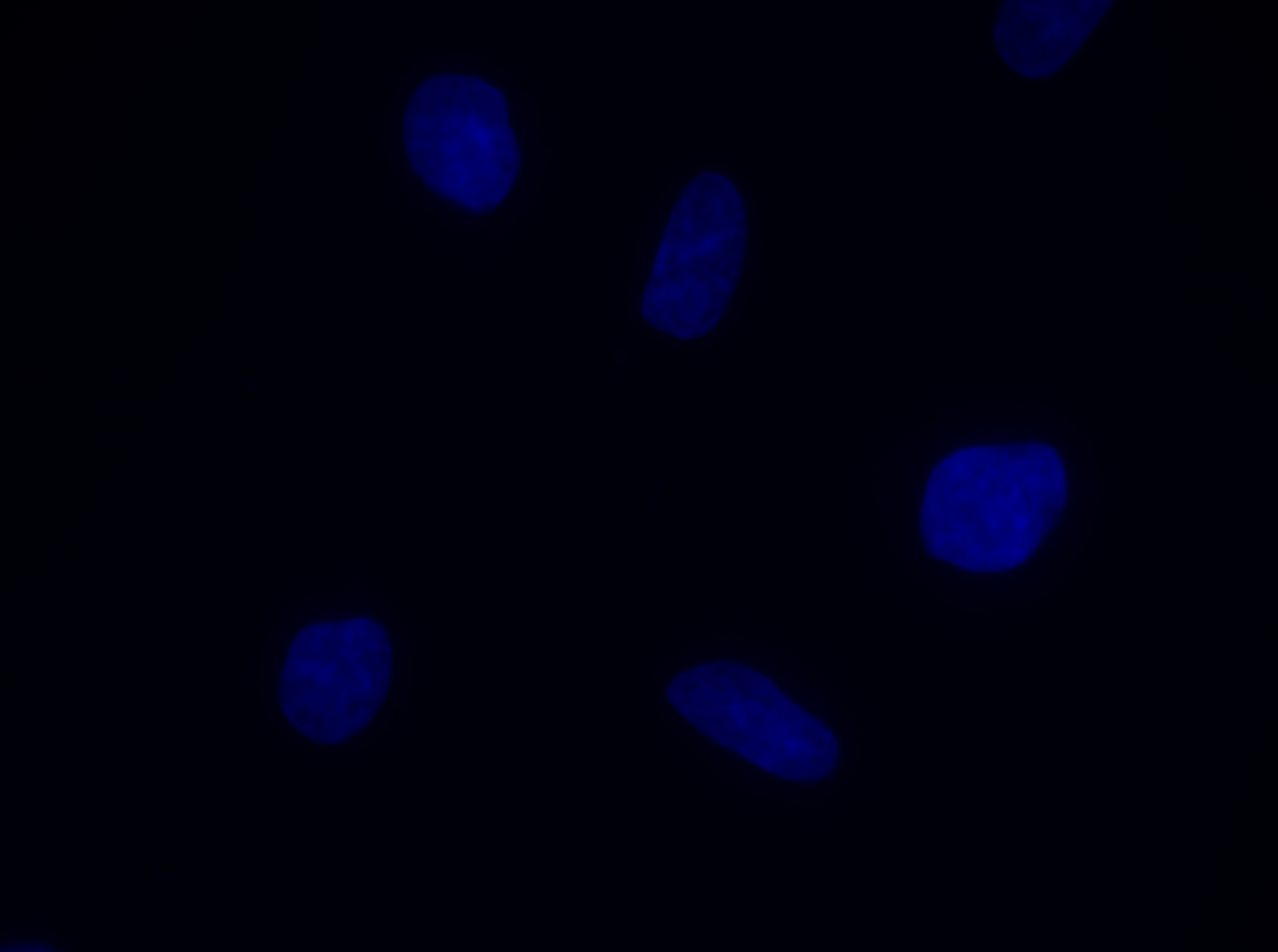

Supplement: Supplementary file 3 — Source data Fig. 1 [file 44321_2025_254_MOESM3_ESM.zip › Figure 1/Fig1C/H1703_DAPI.TIF]

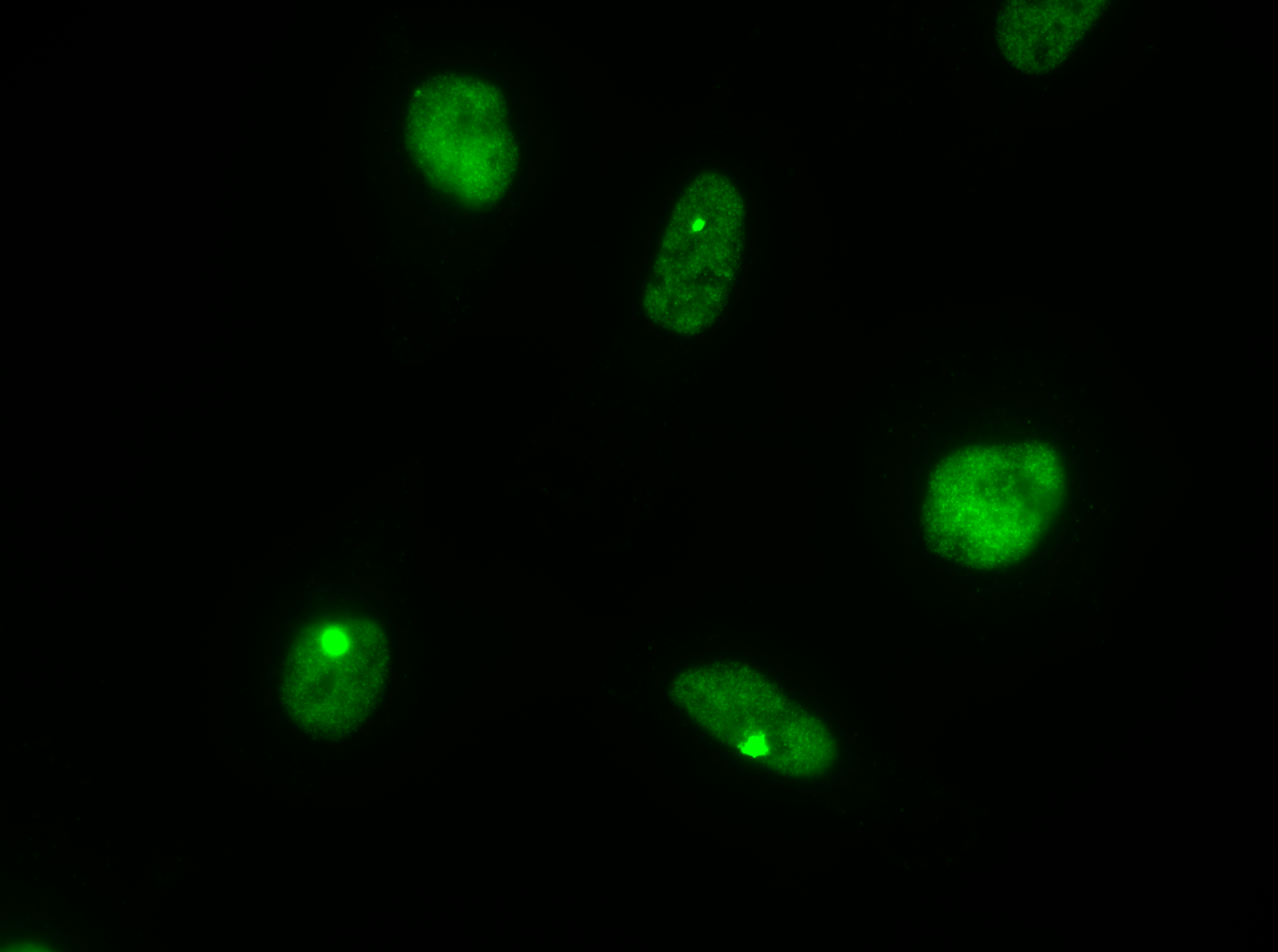

Supplement: Supplementary file 3 — Source data Fig. 1 [file 44321_2025_254_MOESM3_ESM.zip › Figure 1/Fig1C/H1703_FITC.TIF]

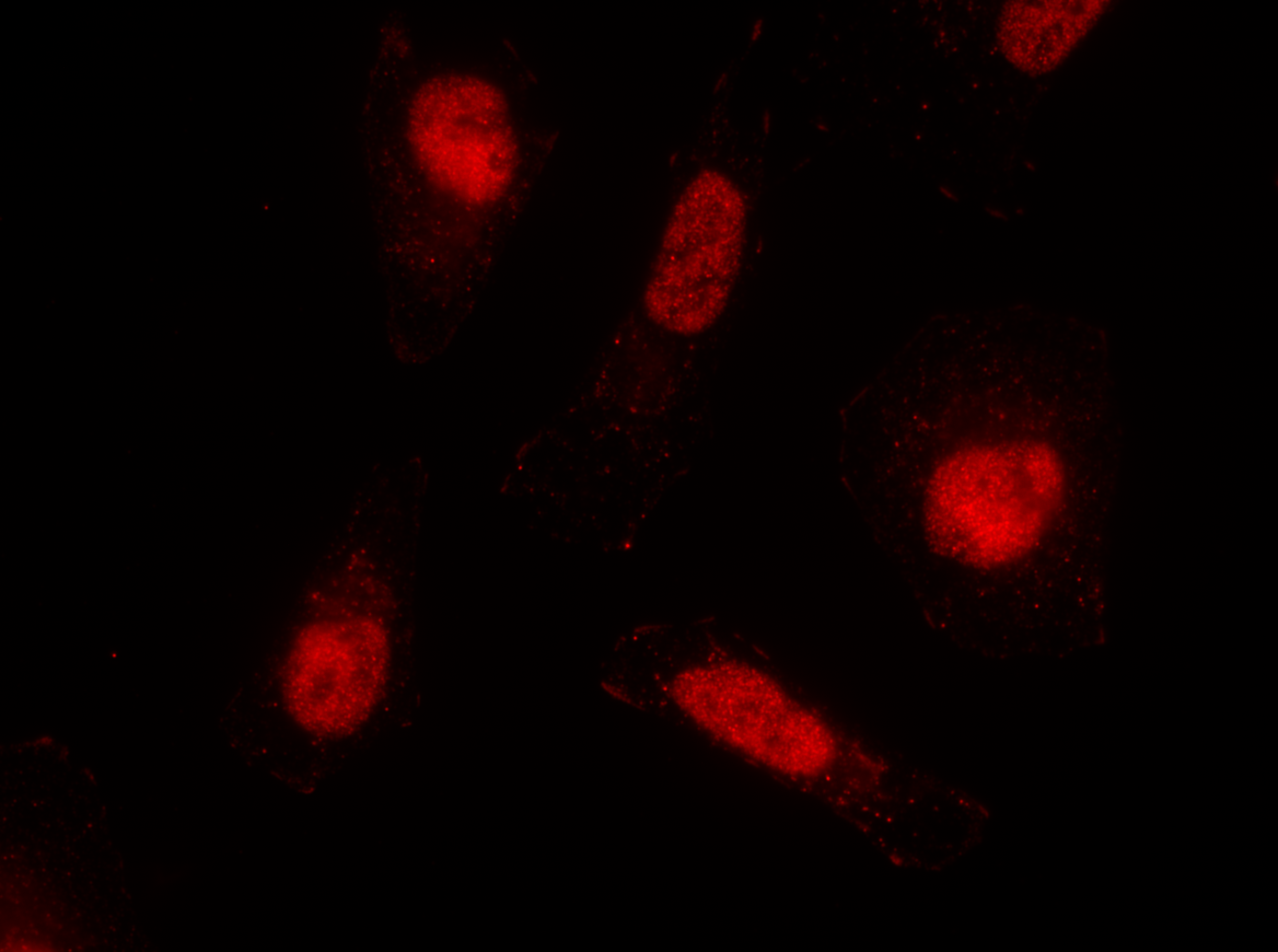

Supplement: Supplementary file 3 — Source data Fig. 1 [file 44321_2025_254_MOESM3_ESM.zip › Figure 1/Fig1C/H1703_Rhodamine.TIF]

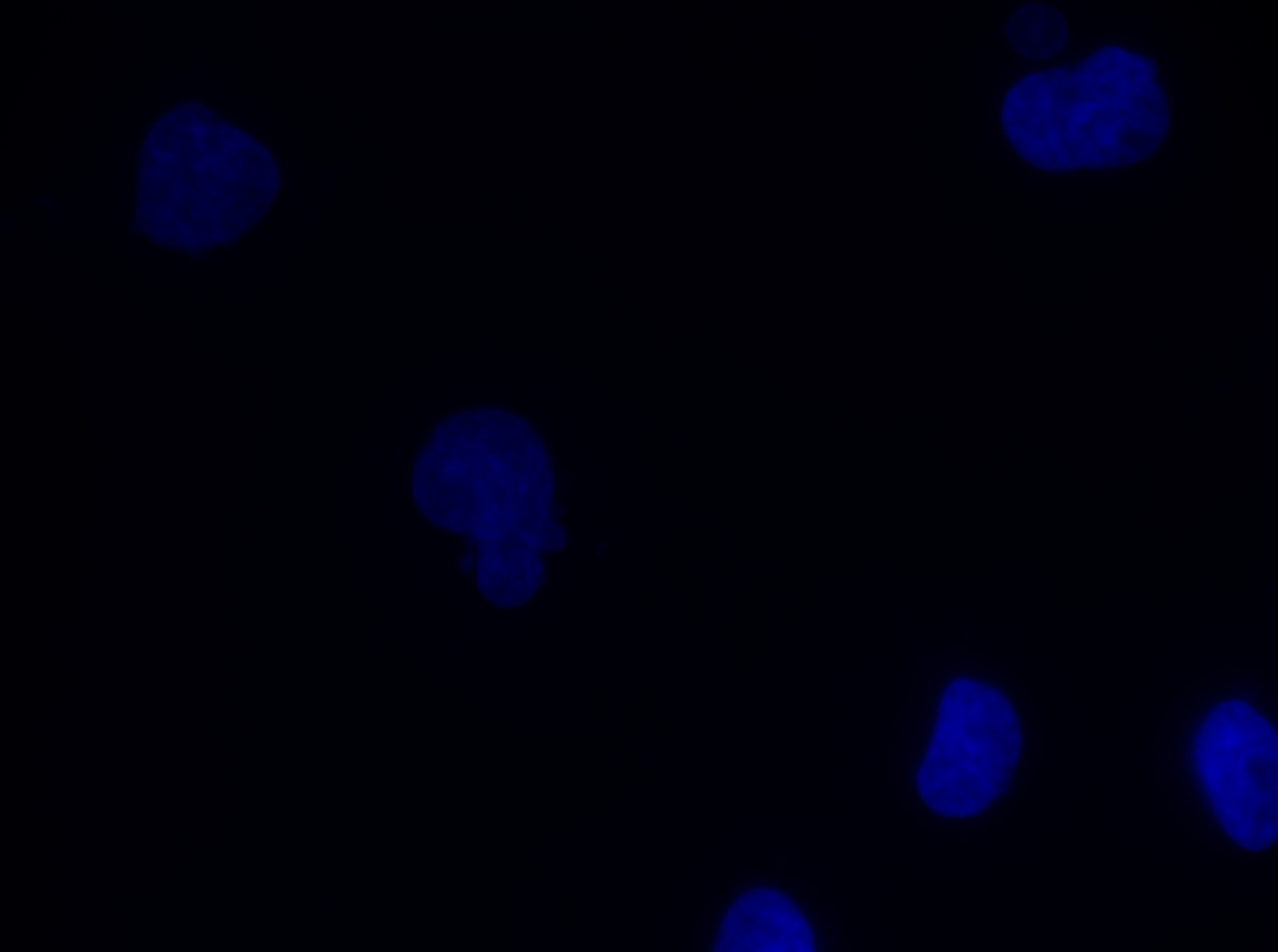

Supplement: Supplementary file 3 — Source data Fig. 1 [file 44321_2025_254_MOESM3_ESM.zip › Figure 1/Fig1C/H23_DAPI.TIF]

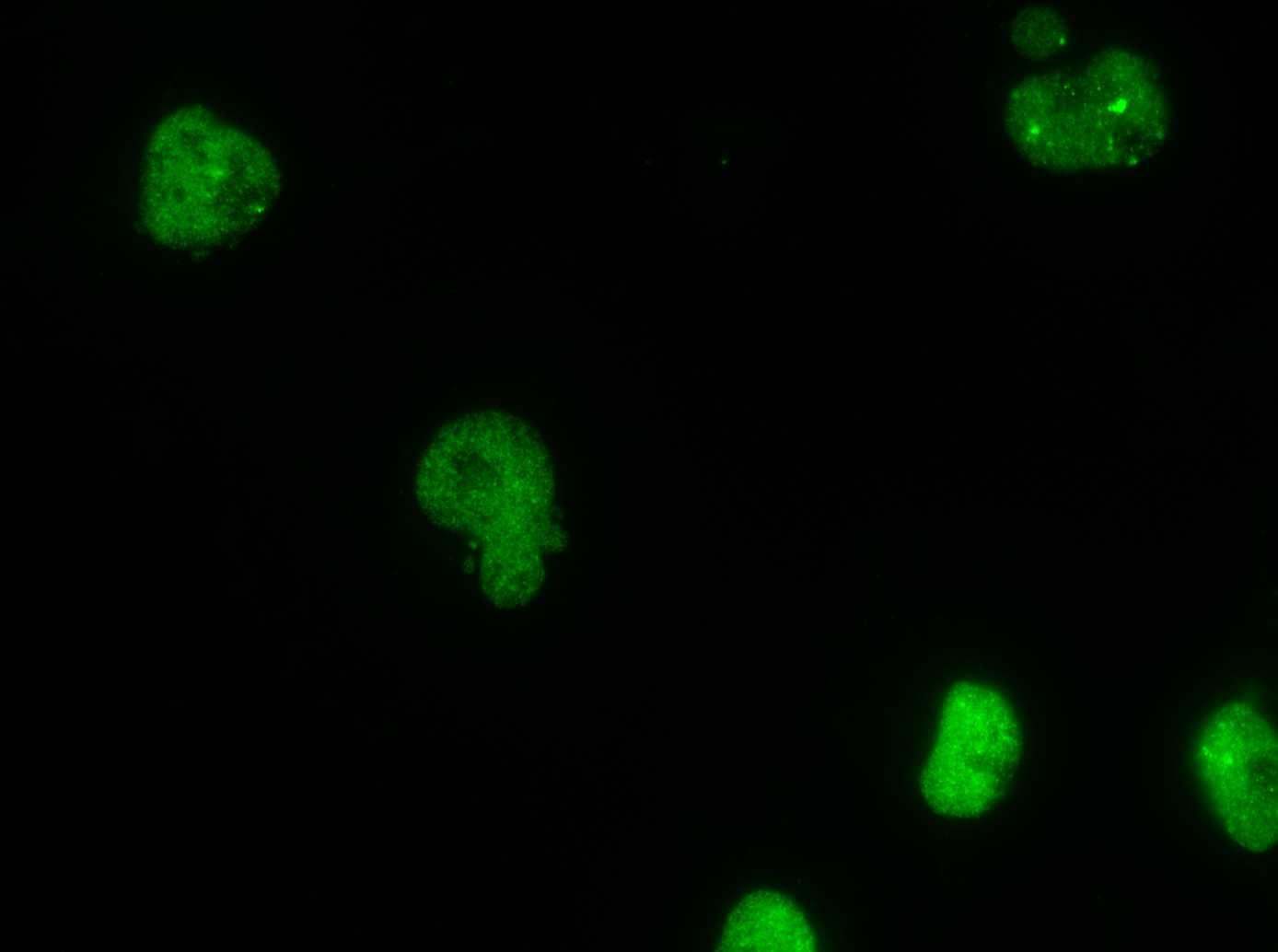

Supplement: Supplementary file 3 — Source data Fig. 1 [file 44321_2025_254_MOESM3_ESM.zip › Figure 1/Fig1C/H23_FITC.TIF]

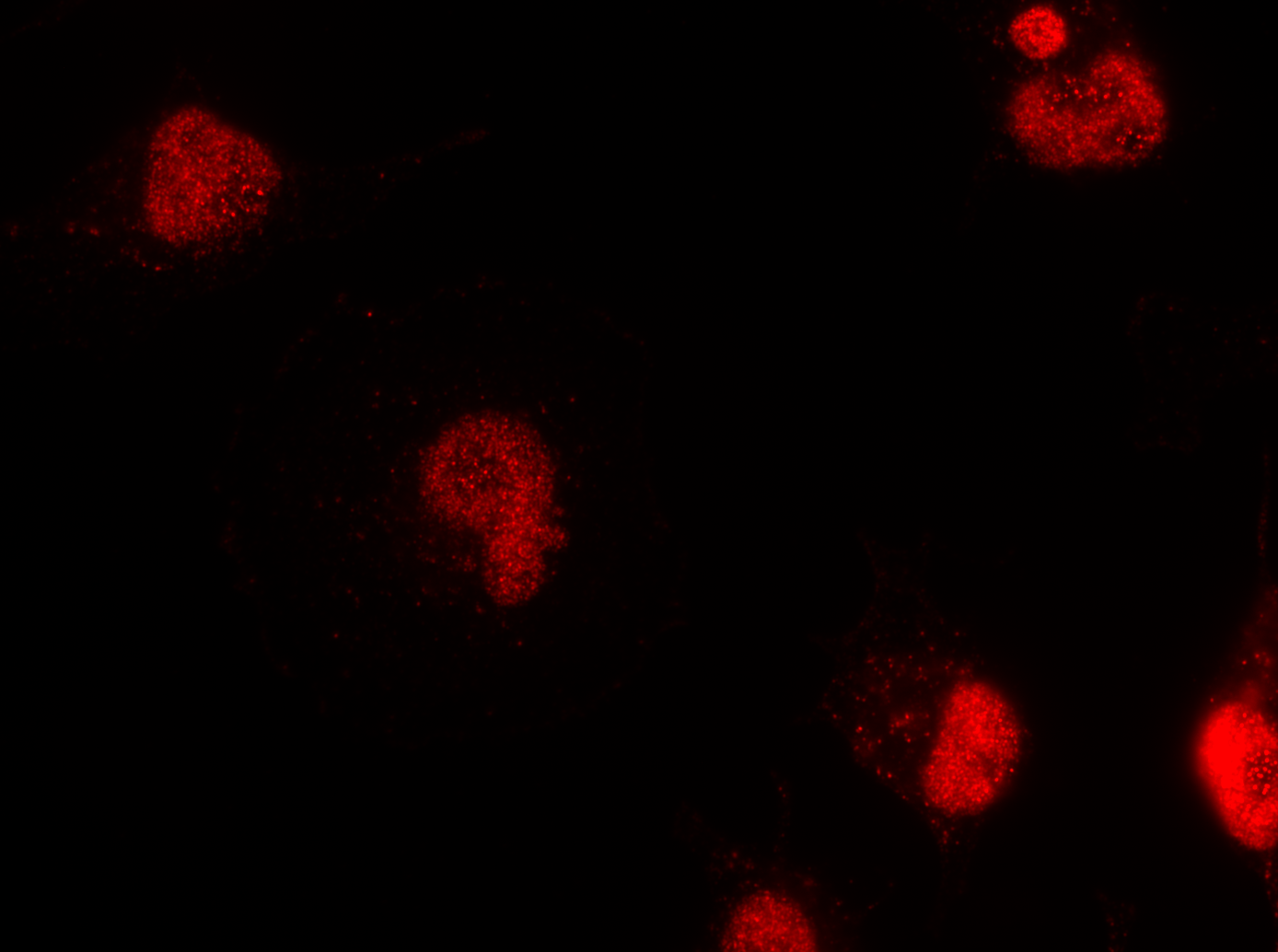

Supplement: Supplementary file 3 — Source data Fig. 1 [file 44321_2025_254_MOESM3_ESM.zip › Figure 1/Fig1C/H23_Rhodamine.TIF]

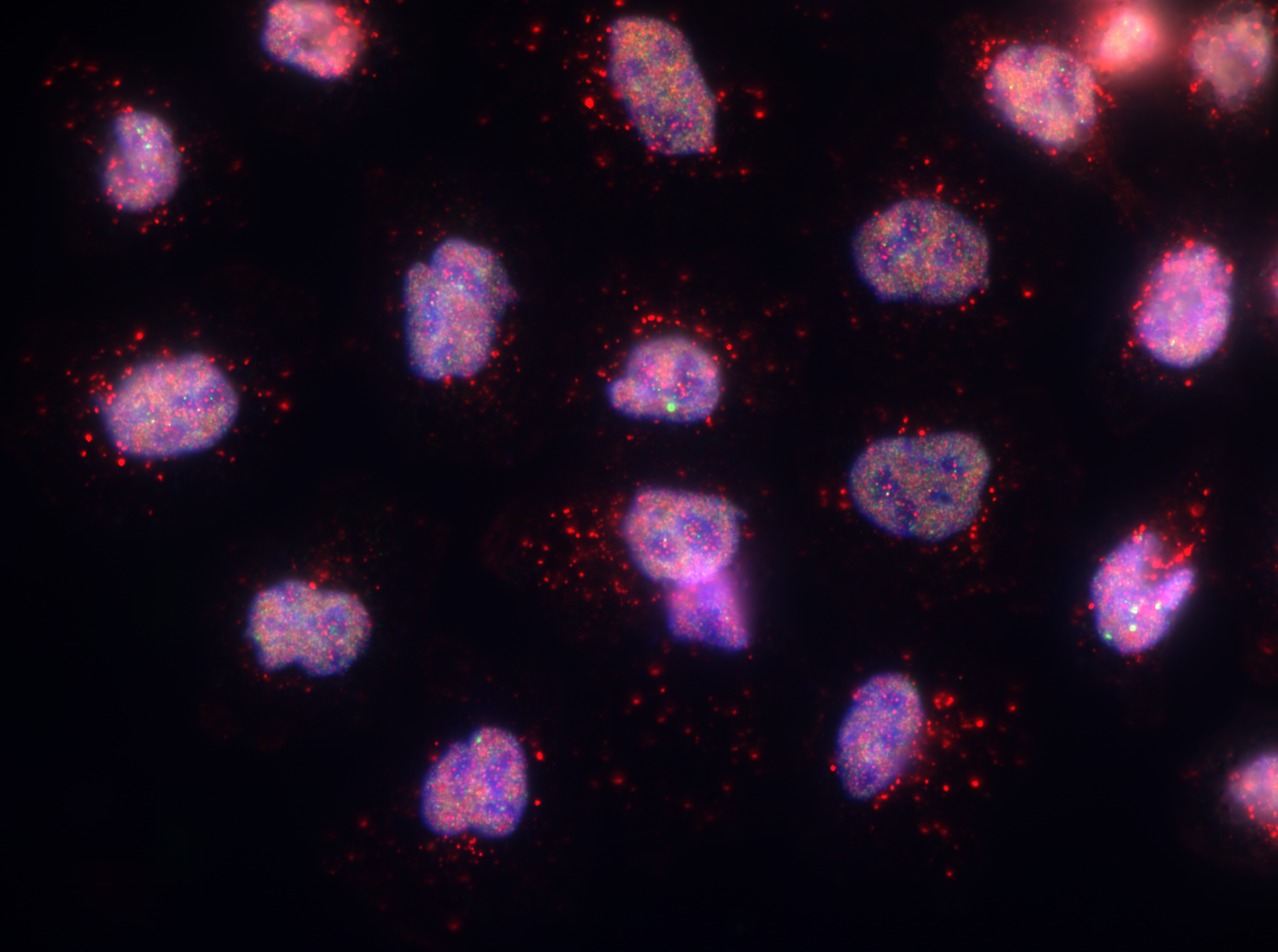

Supplement: Supplementary file 3 — Source data Fig. 1 [file 44321_2025_254_MOESM3_ESM.zip › Figure 1/Fig1C/H460_(DAPI+FITC+Rhodamine).TIF]

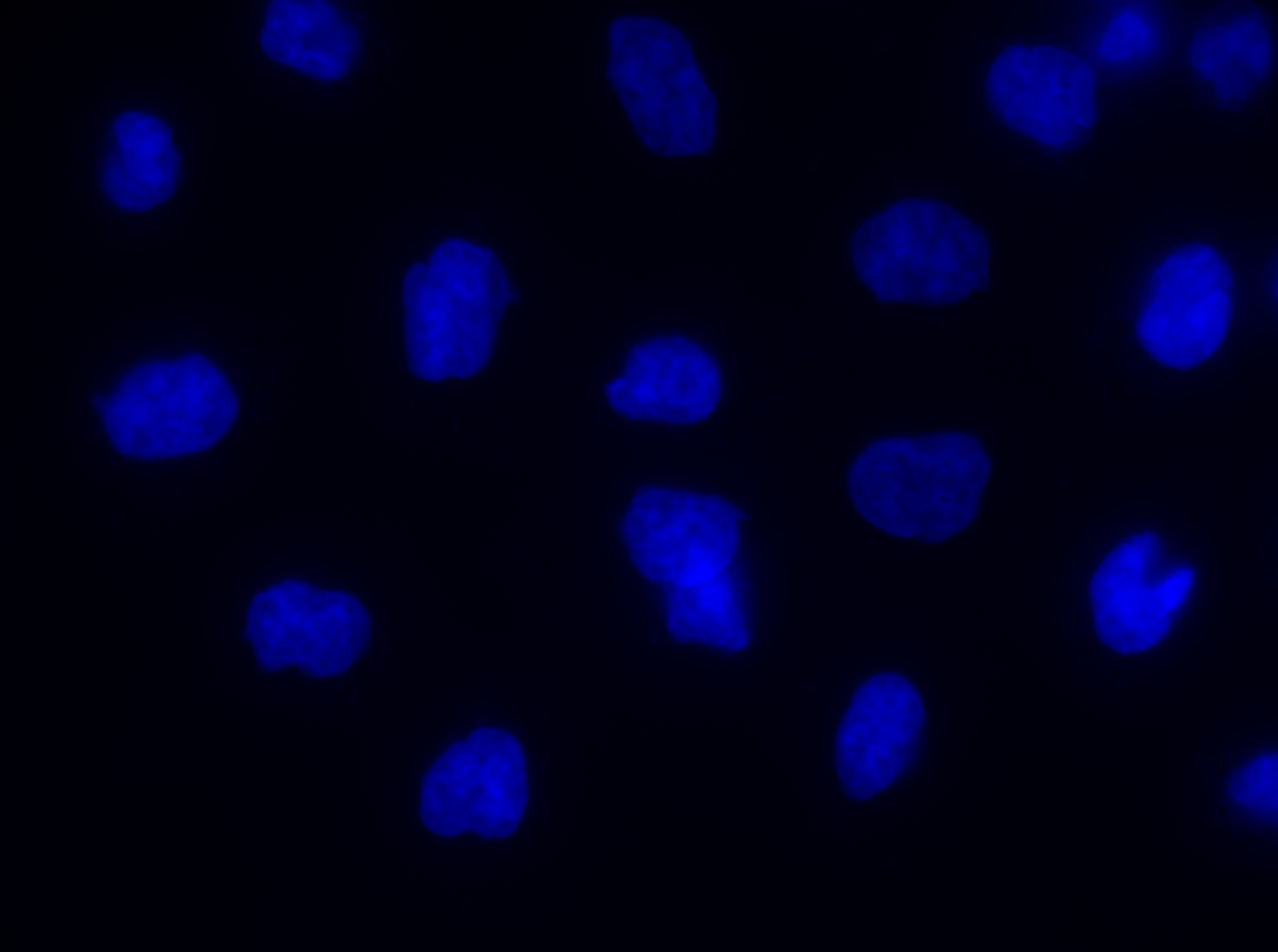

Supplement: Supplementary file 3 — Source data Fig. 1 [file 44321_2025_254_MOESM3_ESM.zip › Figure 1/Fig1C/H460_DAPI.TIF]

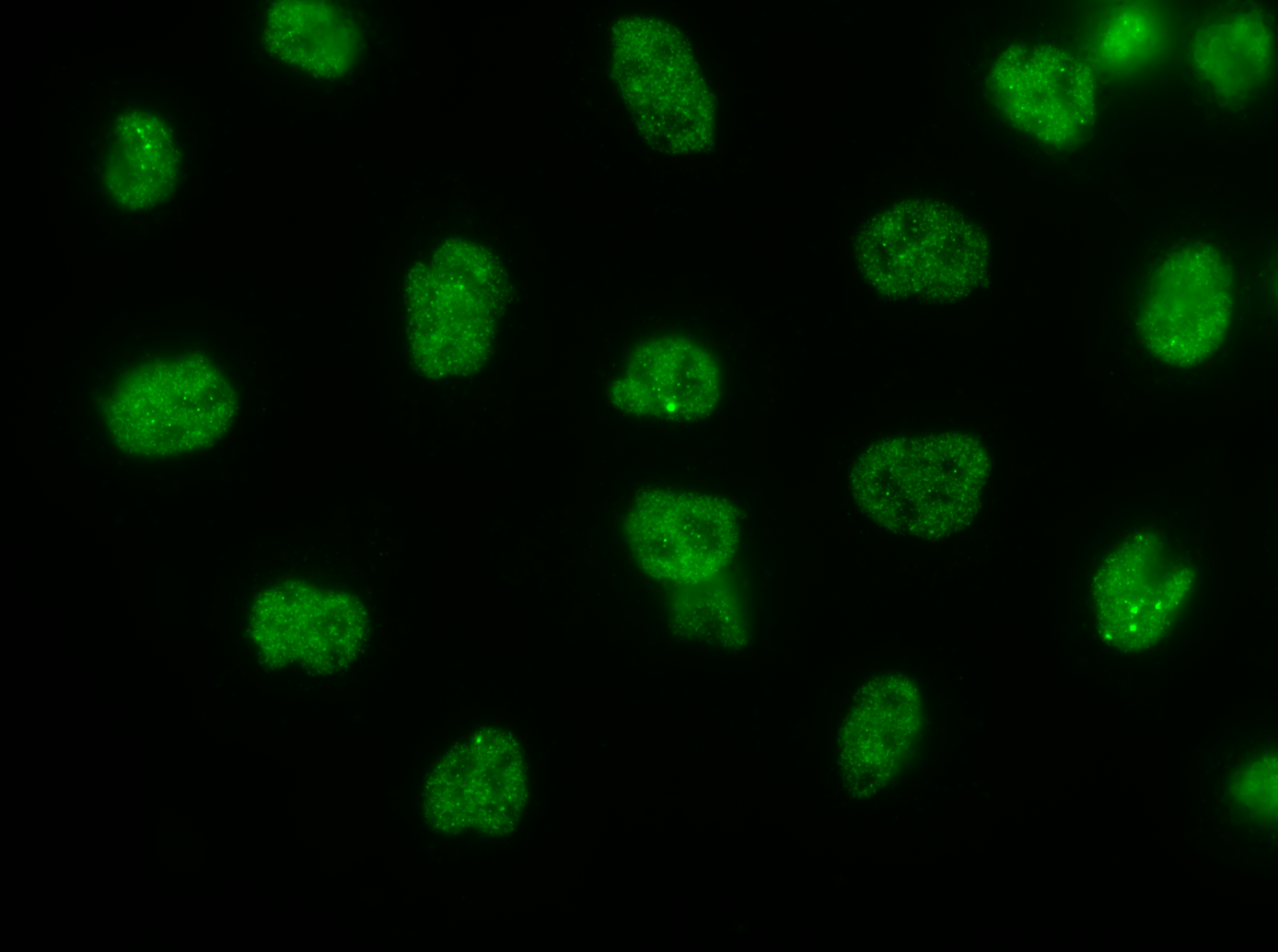

Supplement: Supplementary file 3 — Source data Fig. 1 [file 44321_2025_254_MOESM3_ESM.zip › Figure 1/Fig1C/H460_FITC.TIF]

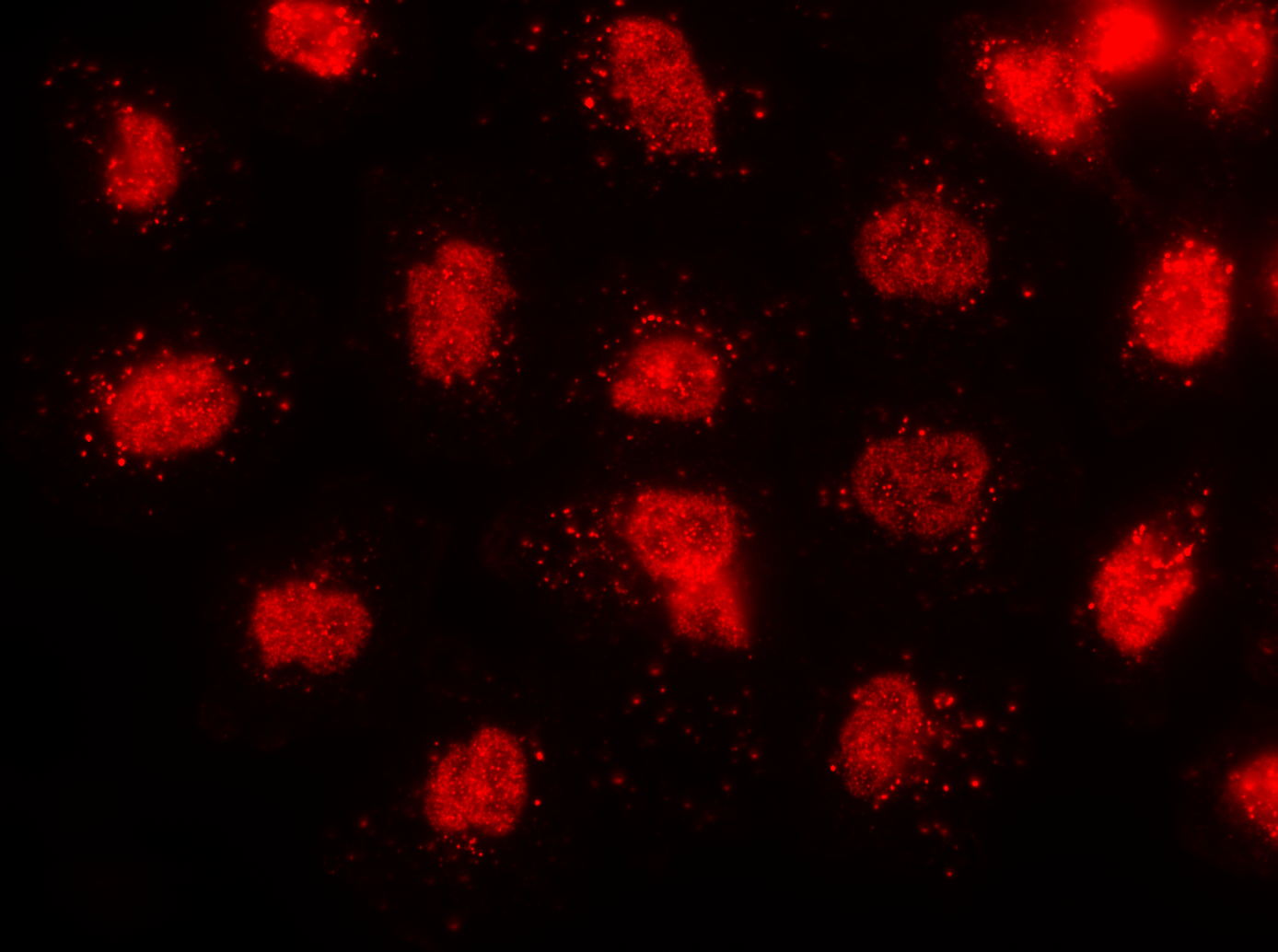

Supplement: Supplementary file 3 — Source data Fig. 1 [file 44321_2025_254_MOESM3_ESM.zip › Figure 1/Fig1C/H460_Rhodamine.TIF]

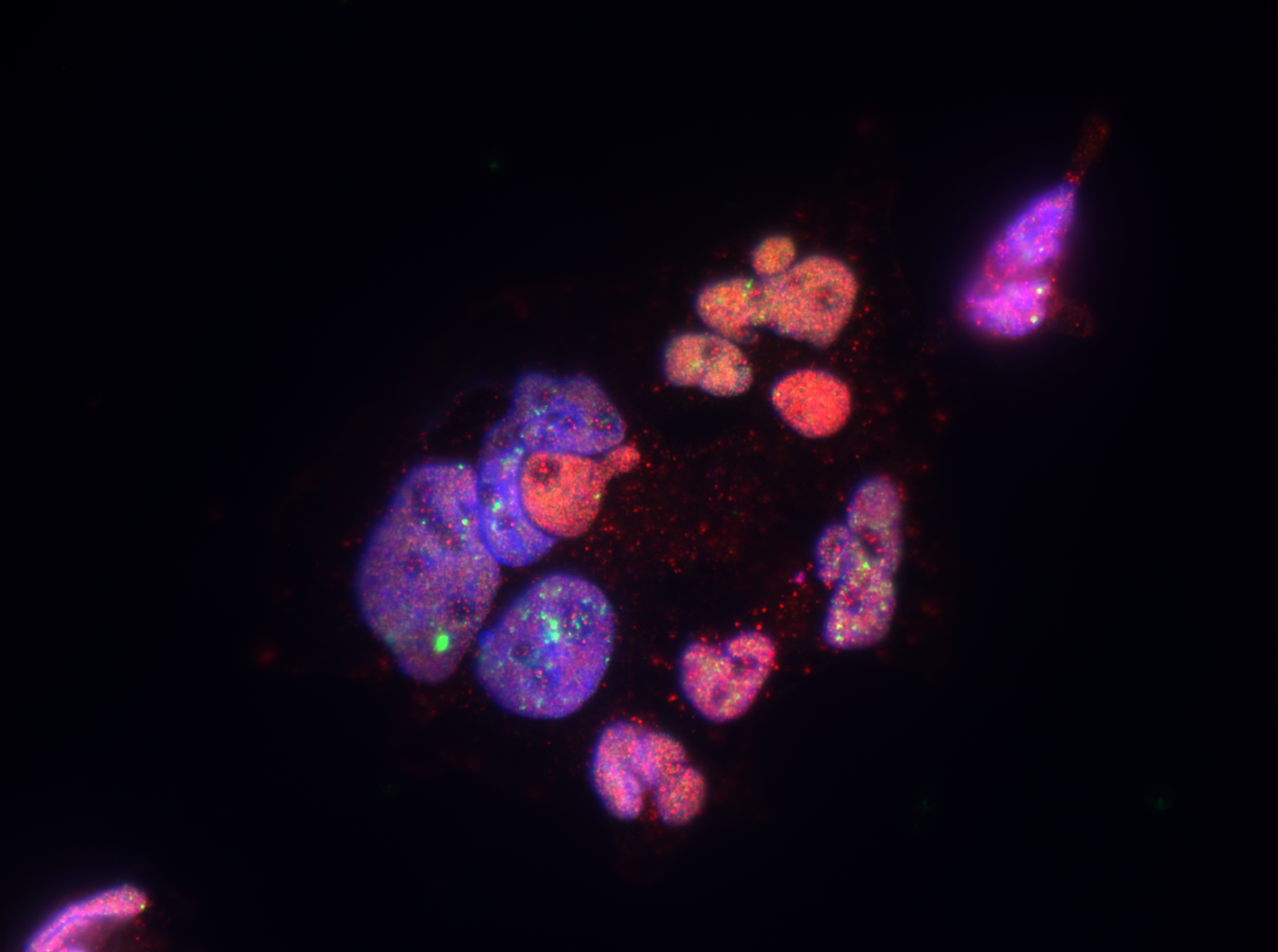

Supplement: Supplementary file 3 — Source data Fig. 1 [file 44321_2025_254_MOESM3_ESM.zip › Figure 1/Fig1C/H520_(DAPI+FITC+Rhodamine).TIF]

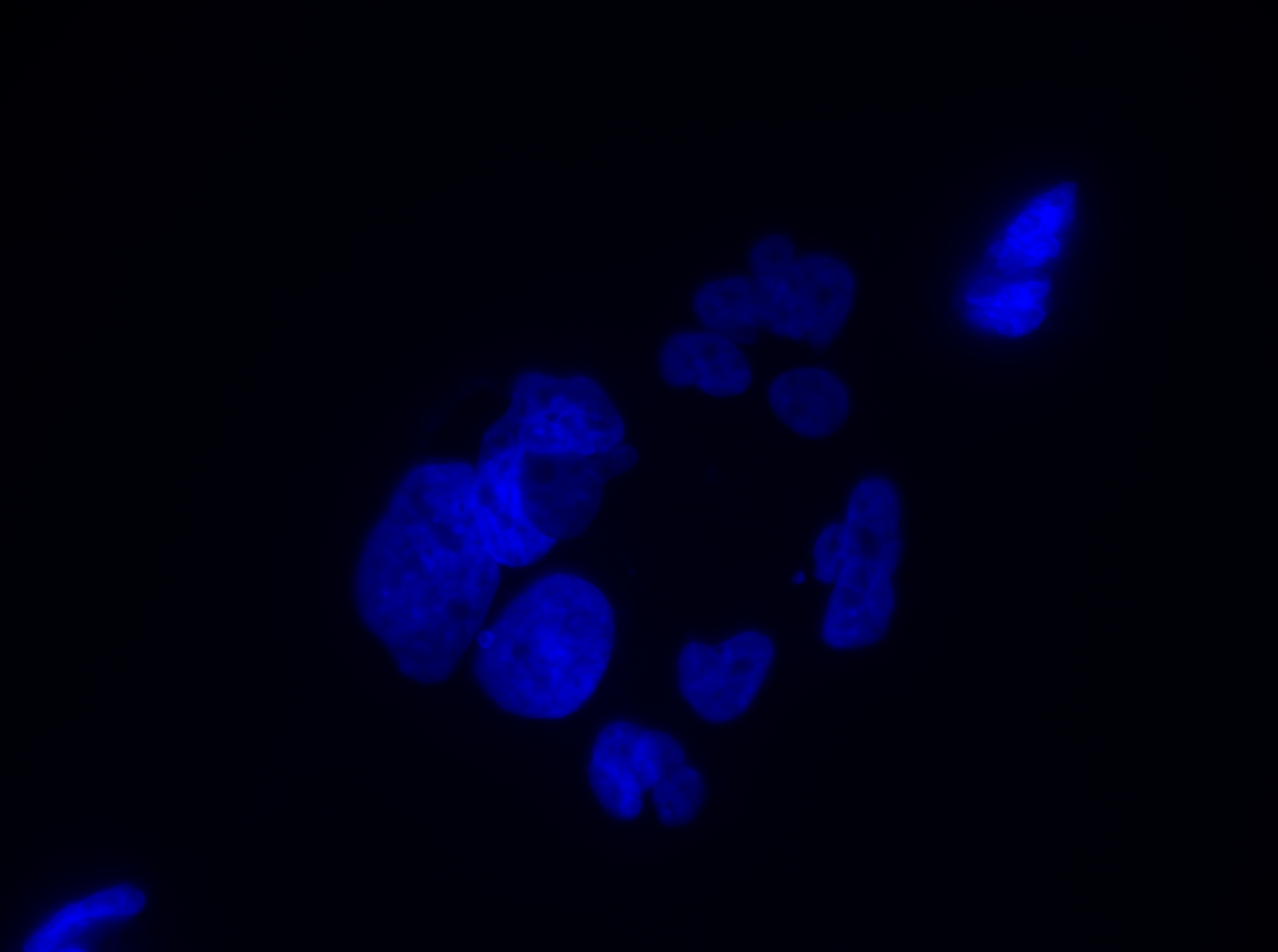

Supplement: Supplementary file 3 — Source data Fig. 1 [file 44321_2025_254_MOESM3_ESM.zip › Figure 1/Fig1C/H520_DAPI.TIF]

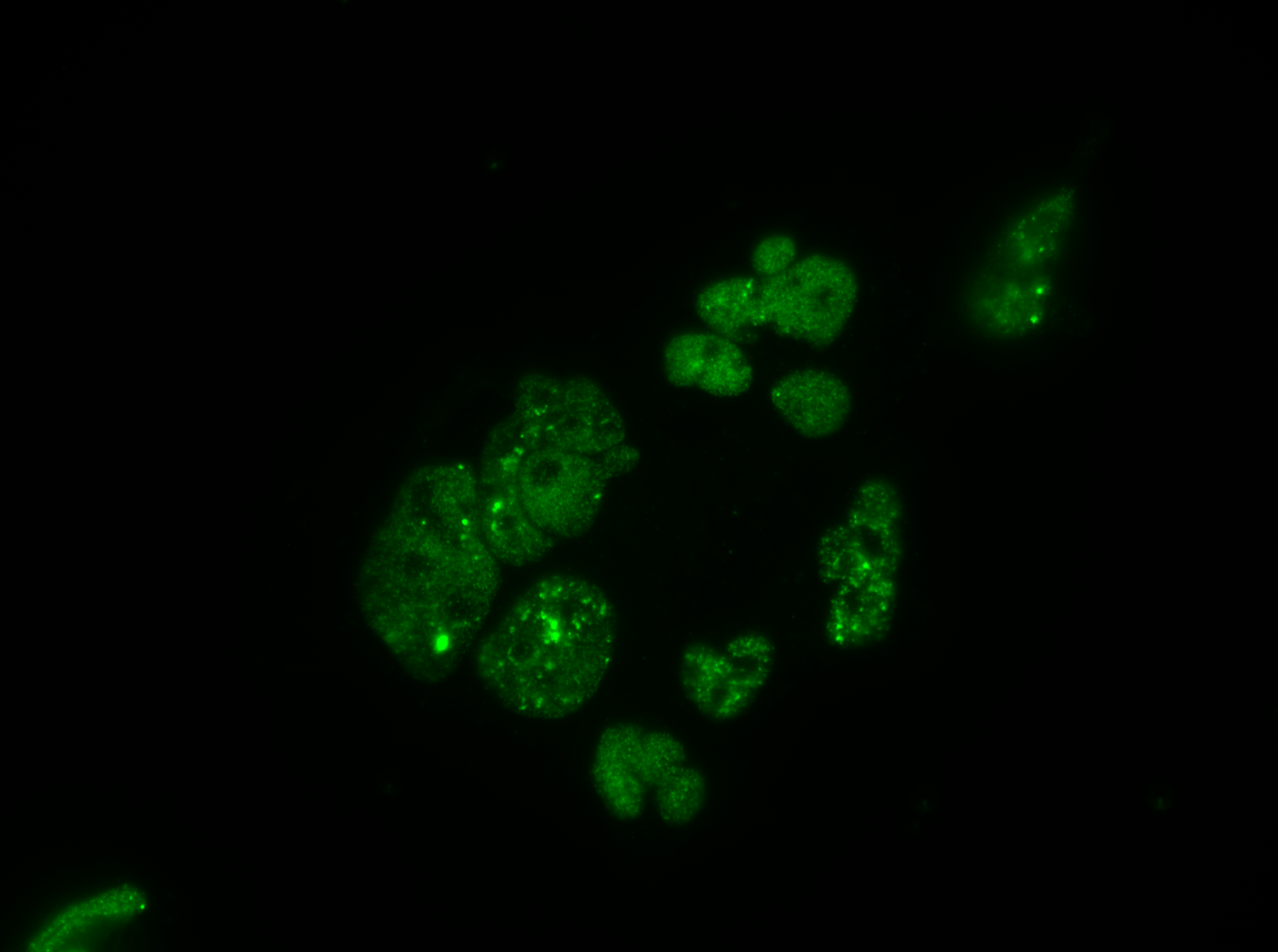

Supplement: Supplementary file 3 — Source data Fig. 1 [file 44321_2025_254_MOESM3_ESM.zip › Figure 1/Fig1C/H520_FITC.TIF]

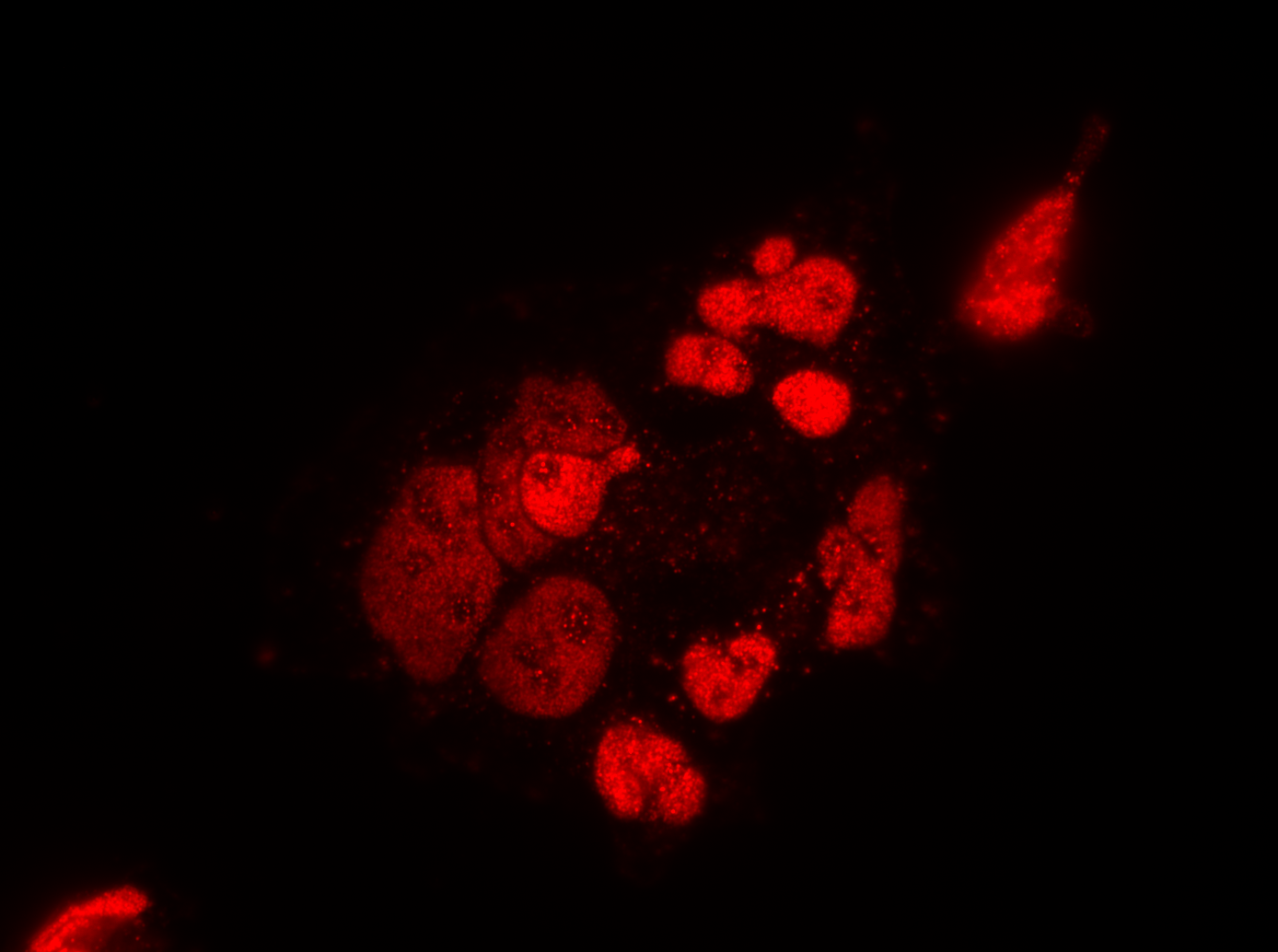

Supplement: Supplementary file 3 — Source data Fig. 1 [file 44321_2025_254_MOESM3_ESM.zip › Figure 1/Fig1C/H520_Rhodamine.TIF]

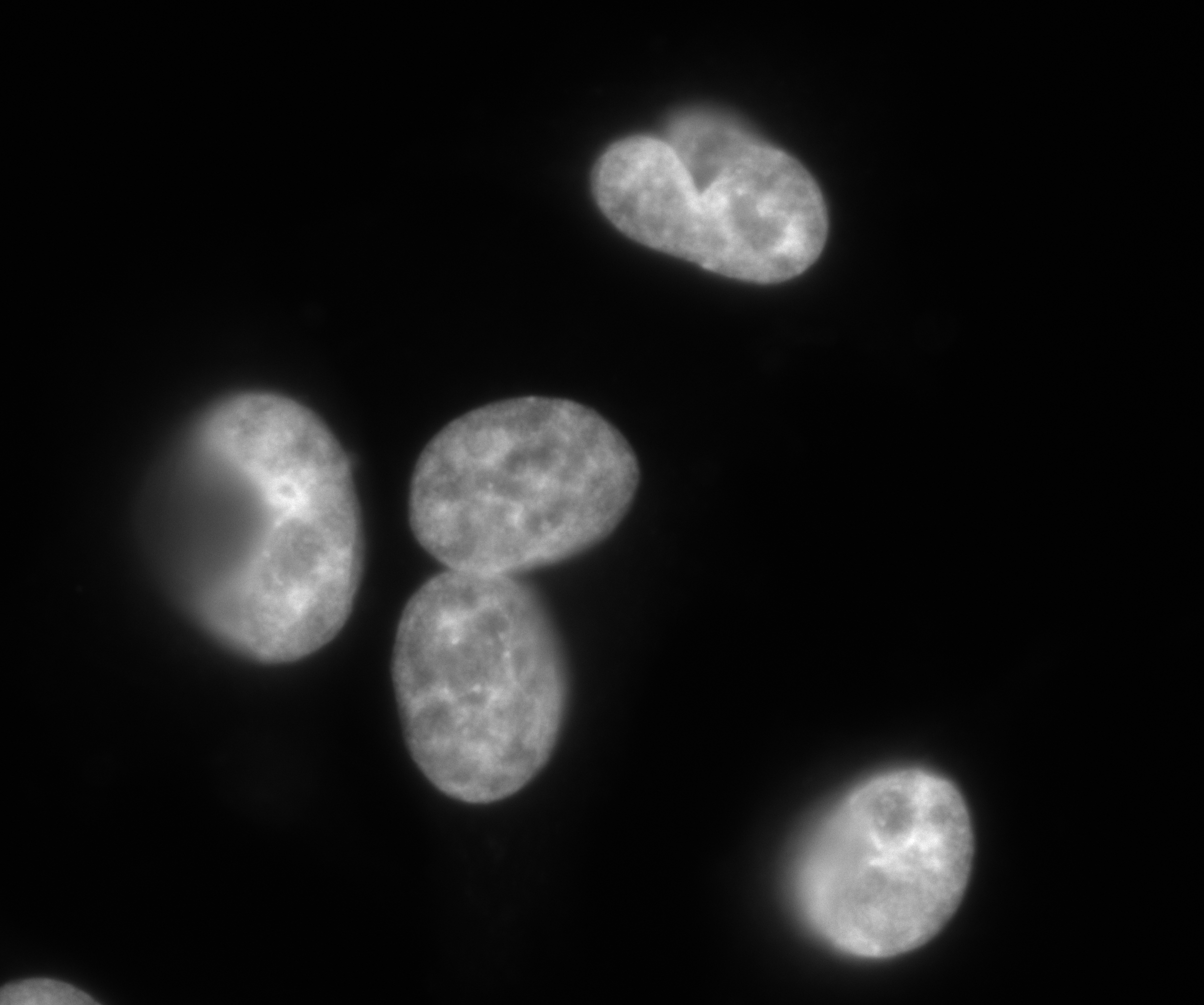

Supplement: Supplementary file 3 — Source data Fig. 1 [file 44321_2025_254_MOESM3_ESM.zip › Figure 1/Fig1D/A549-CTsi_DAPI.tif]

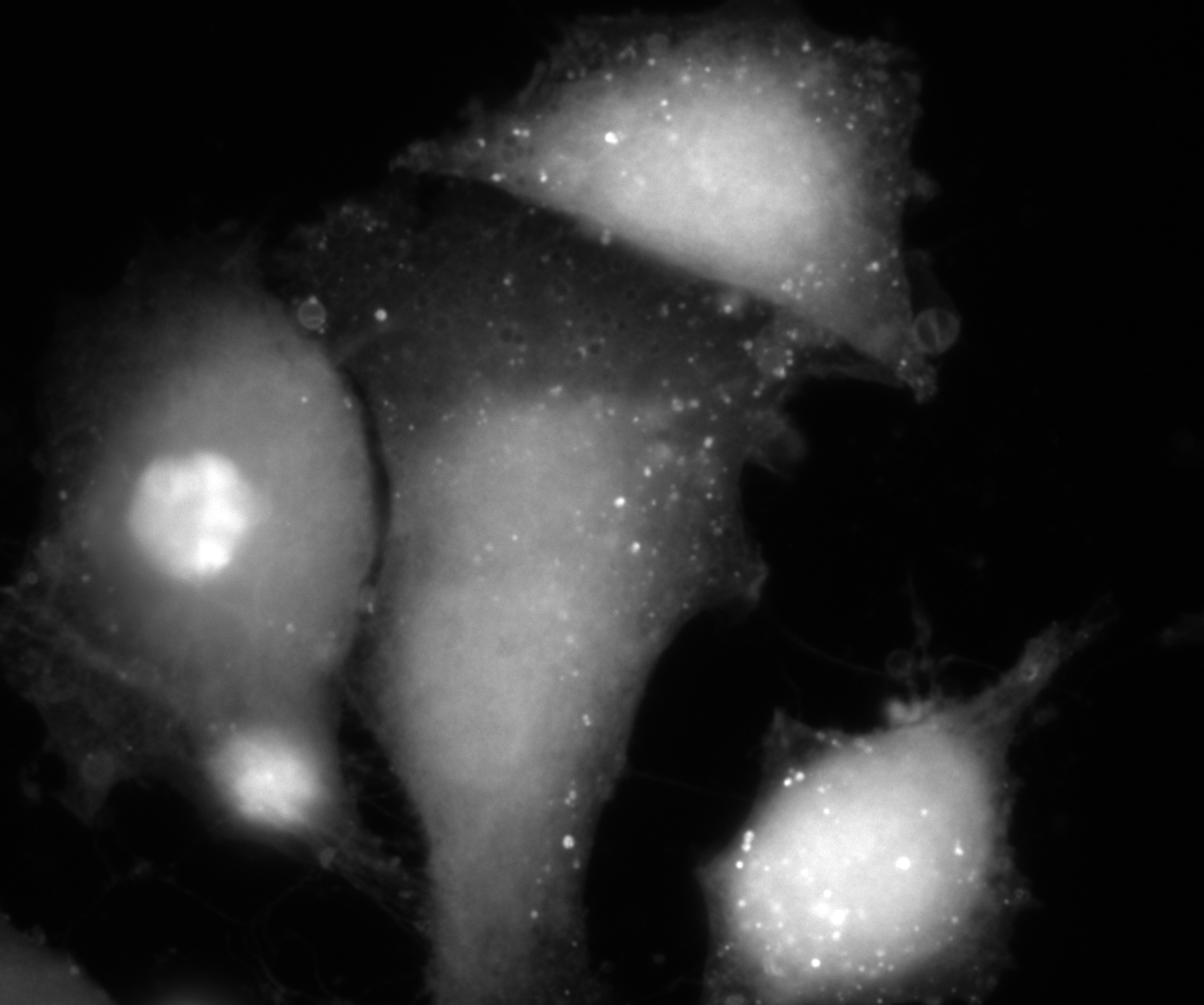

Supplement: Supplementary file 3 — Source data Fig. 1 [file 44321_2025_254_MOESM3_ESM.zip › Figure 1/Fig1D/A549-CTsi_Rhodamine.tif]

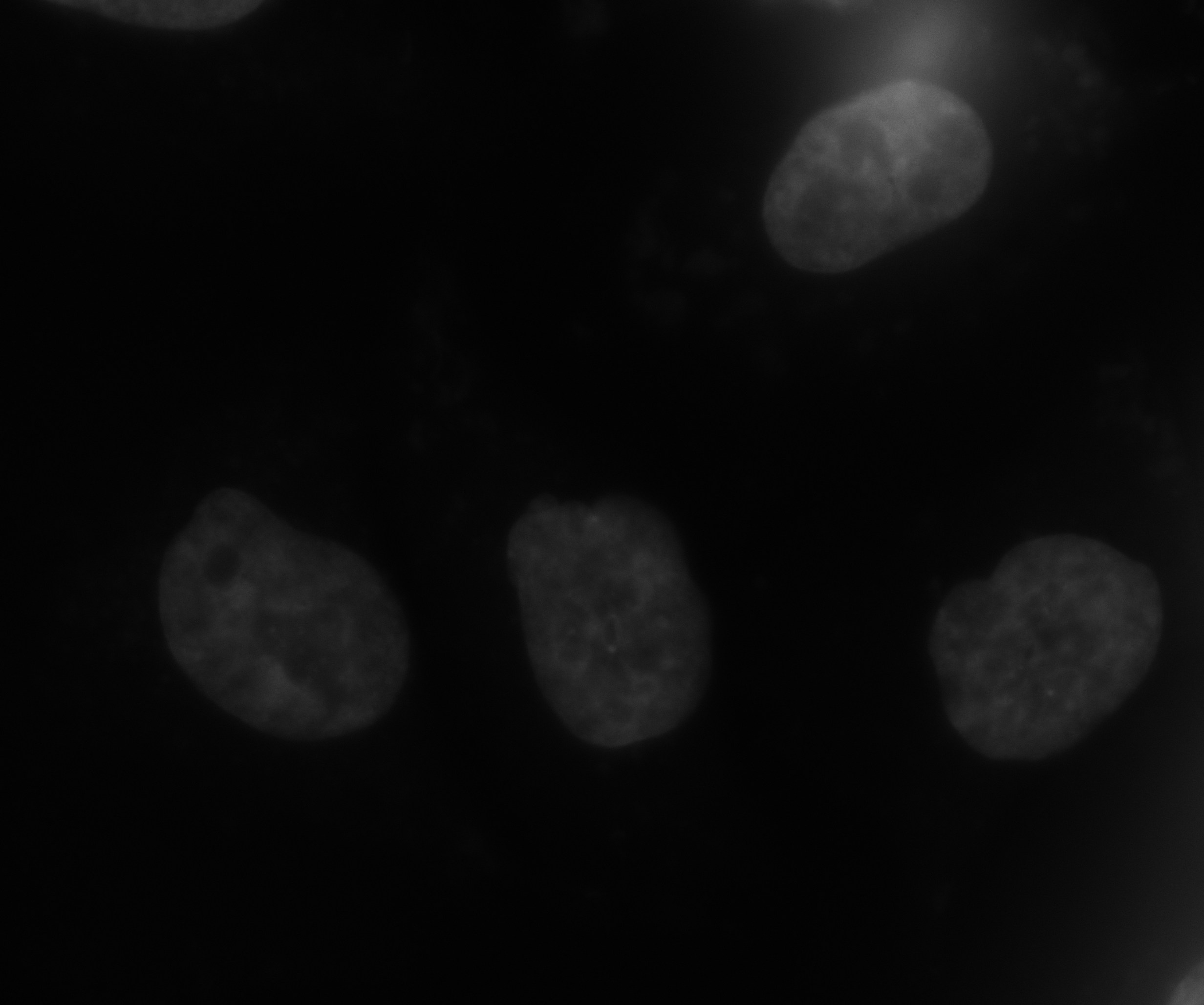

Supplement: Supplementary file 3 — Source data Fig. 1 [file 44321_2025_254_MOESM3_ESM.zip › Figure 1/Fig1D/A549-RARsi_DAPI.tif]

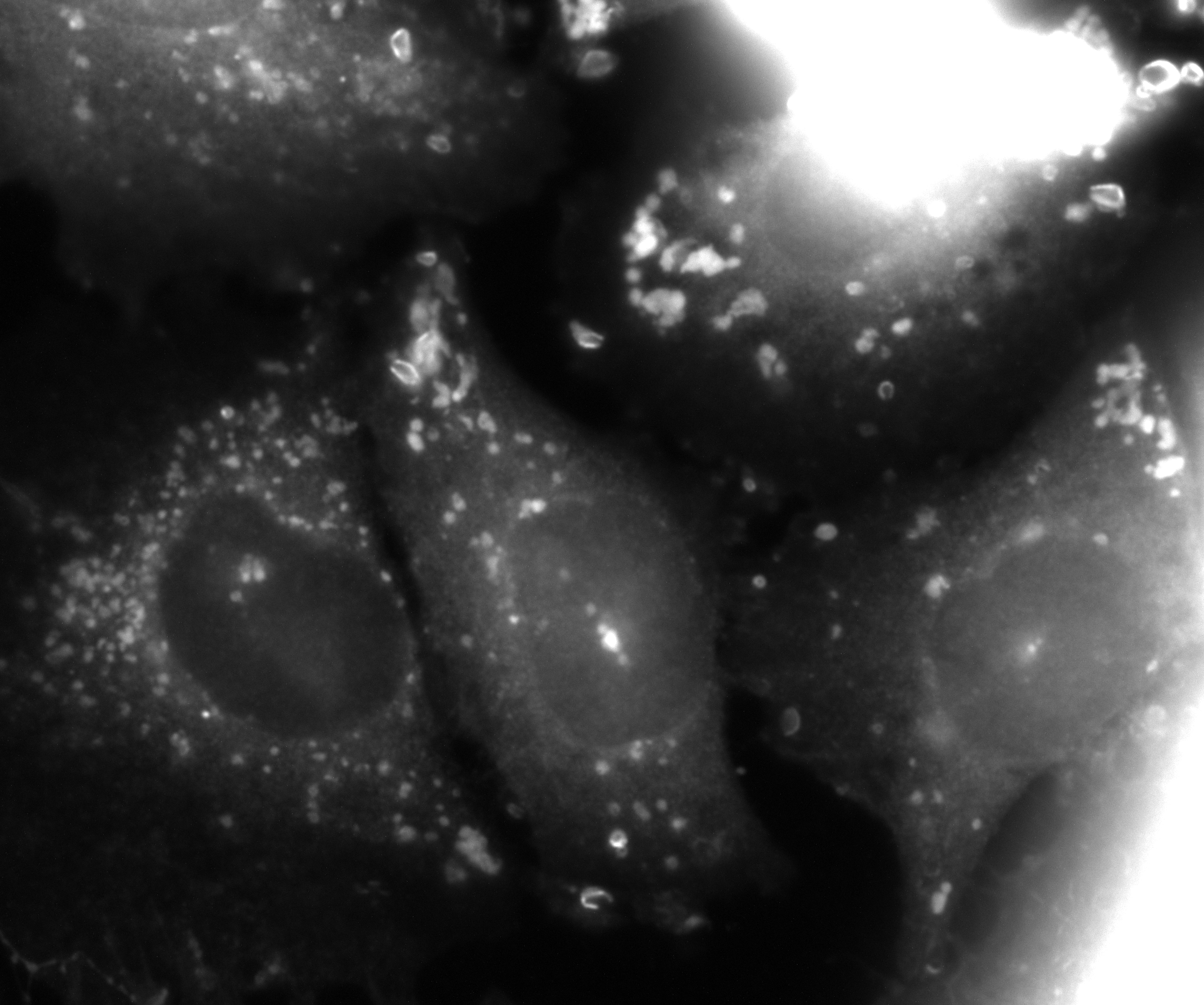

Supplement: Supplementary file 3 — Source data Fig. 1 [file 44321_2025_254_MOESM3_ESM.zip › Figure 1/Fig1D/A549-RARsi_Rhodamine.tif]

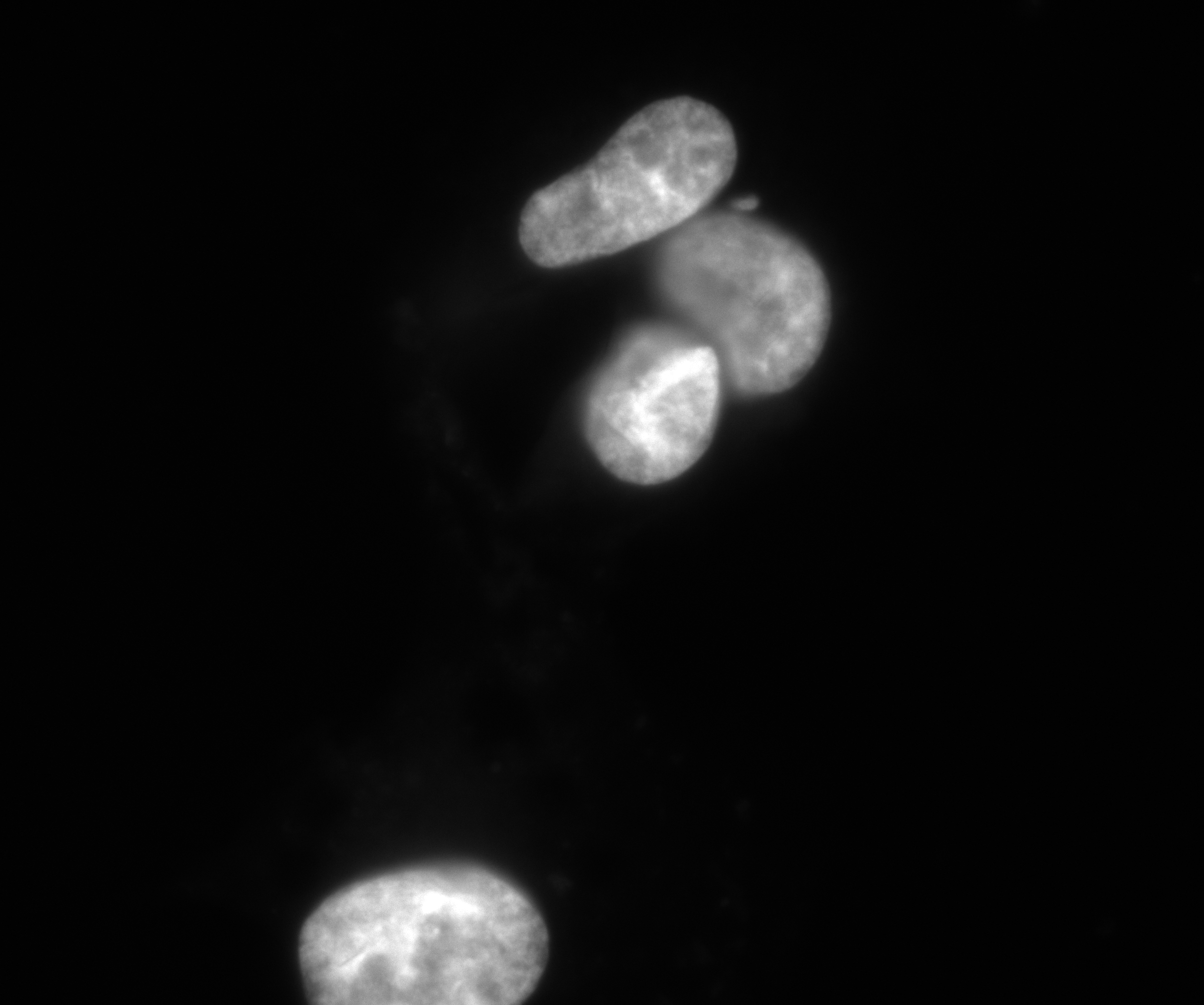

Supplement: Supplementary file 3 — Source data Fig. 1 [file 44321_2025_254_MOESM3_ESM.zip › Figure 1/Fig1G/A549-CTsi_DAPI.tif]

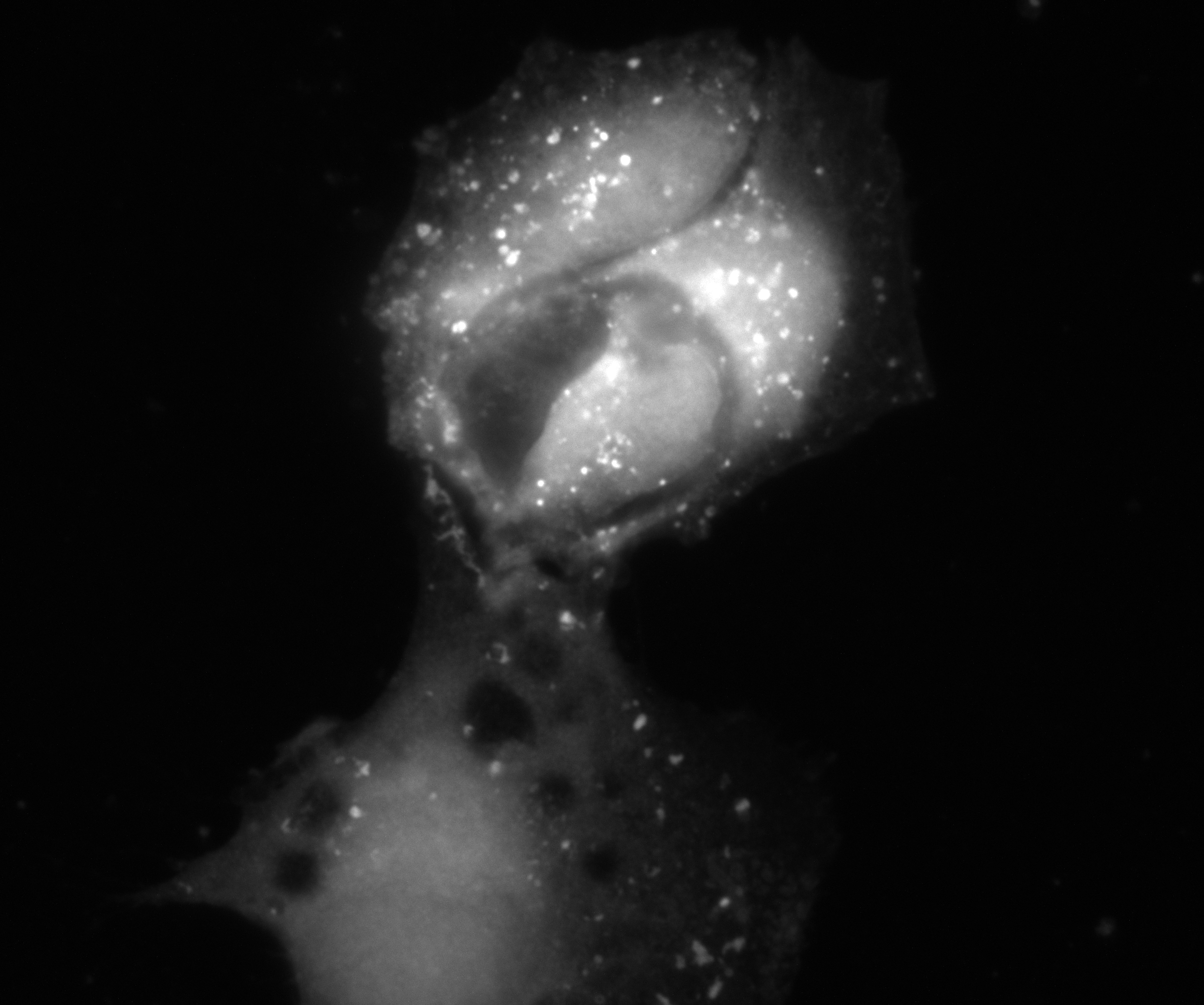

Supplement: Supplementary file 3 — Source data Fig. 1 [file 44321_2025_254_MOESM3_ESM.zip › Figure 1/Fig1G/A549-CTsi_Rhodamine.tif]

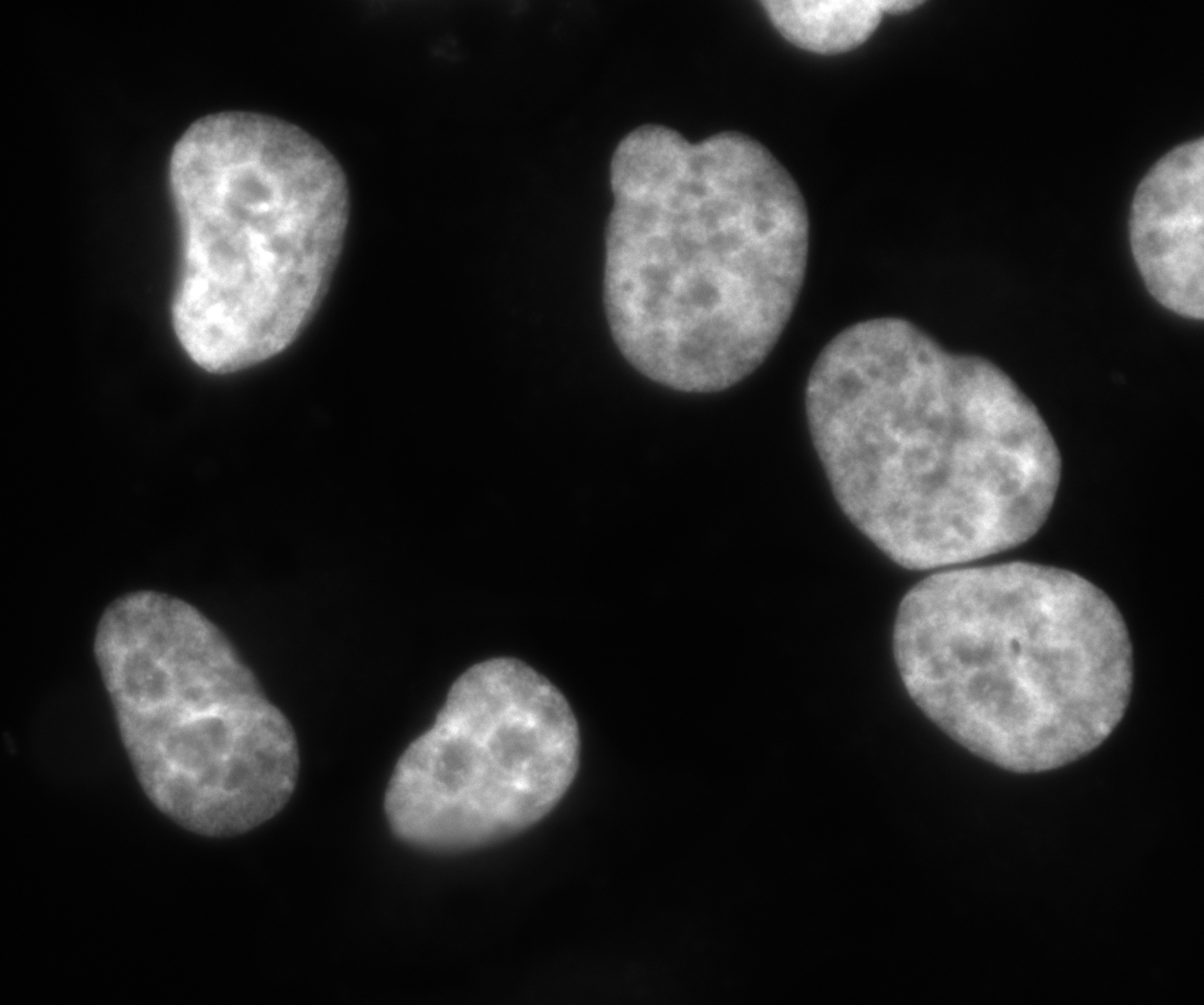

Supplement: Supplementary file 3 — Source data Fig. 1 [file 44321_2025_254_MOESM3_ESM.zip › Figure 1/Fig1G/A549-NCoRsi_DAPI.tif]

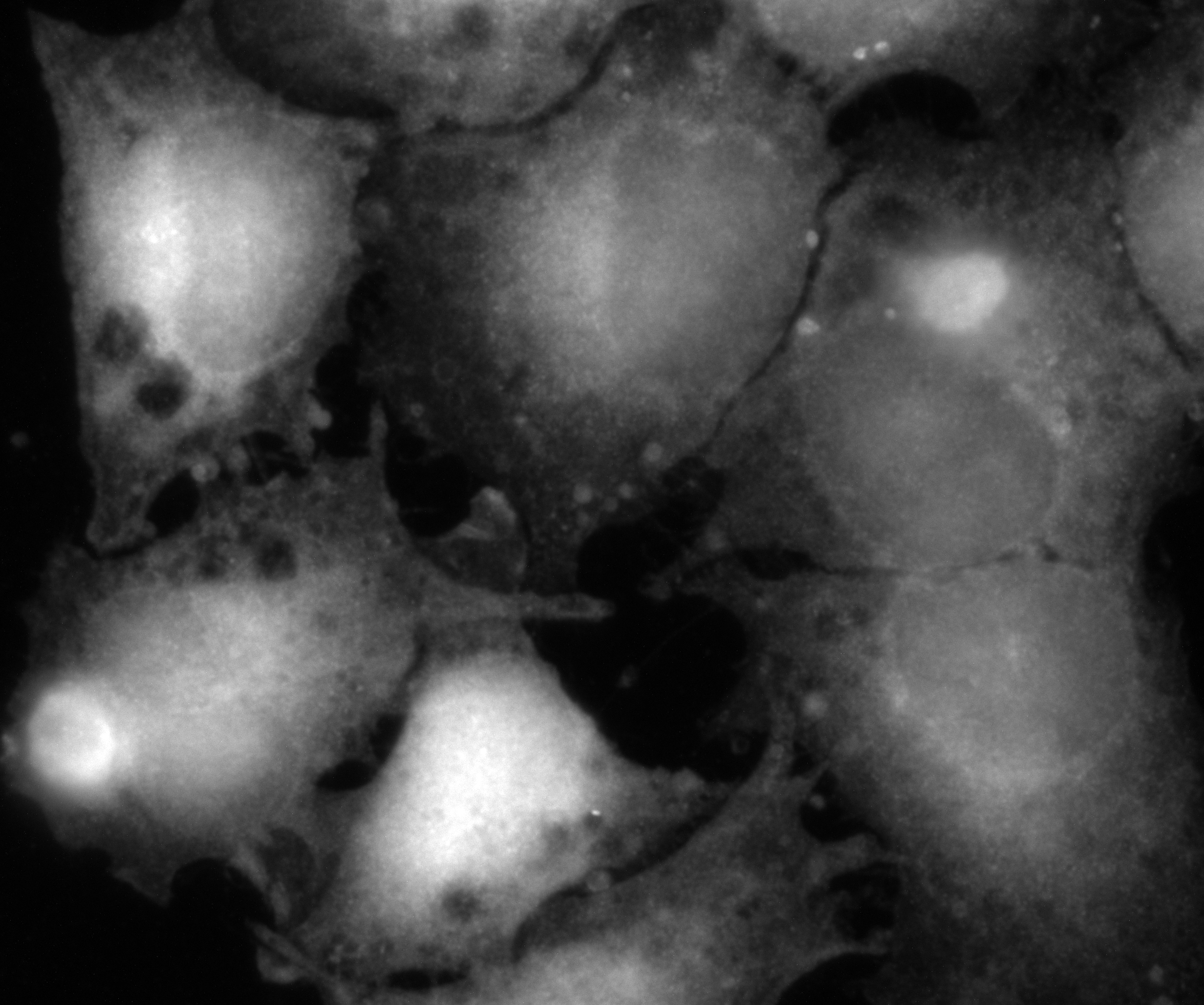

Supplement: Supplementary file 3 — Source data Fig. 1 [file 44321_2025_254_MOESM3_ESM.zip › Figure 1/Fig1G/A549-NCoRsi_Rhodamine.tif]

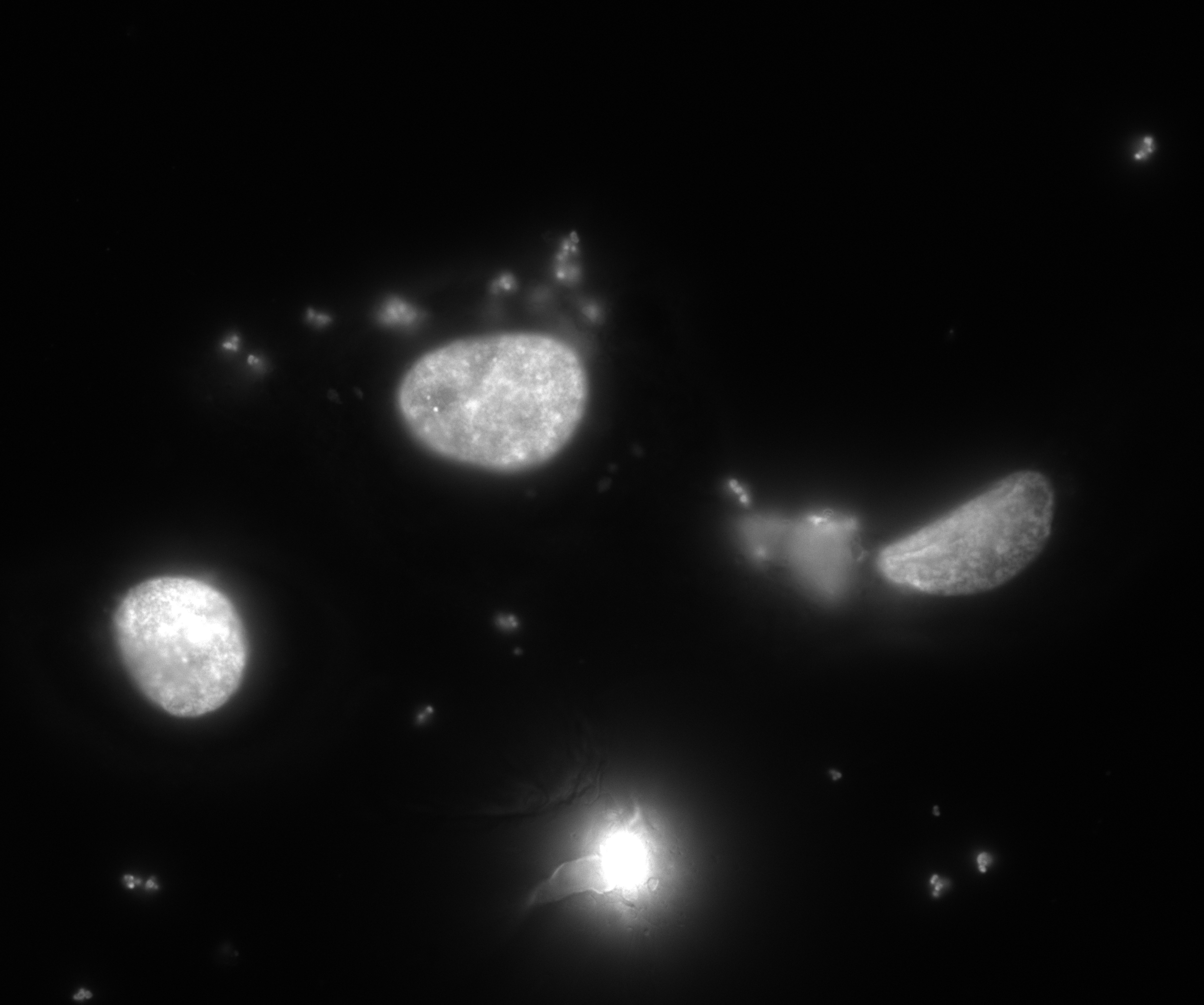

Supplement: Supplementary file 3 — Source data Fig. 1 [file 44321_2025_254_MOESM3_ESM.zip › Figure 1/Fig1H/RARAHT_DAPI.tif]

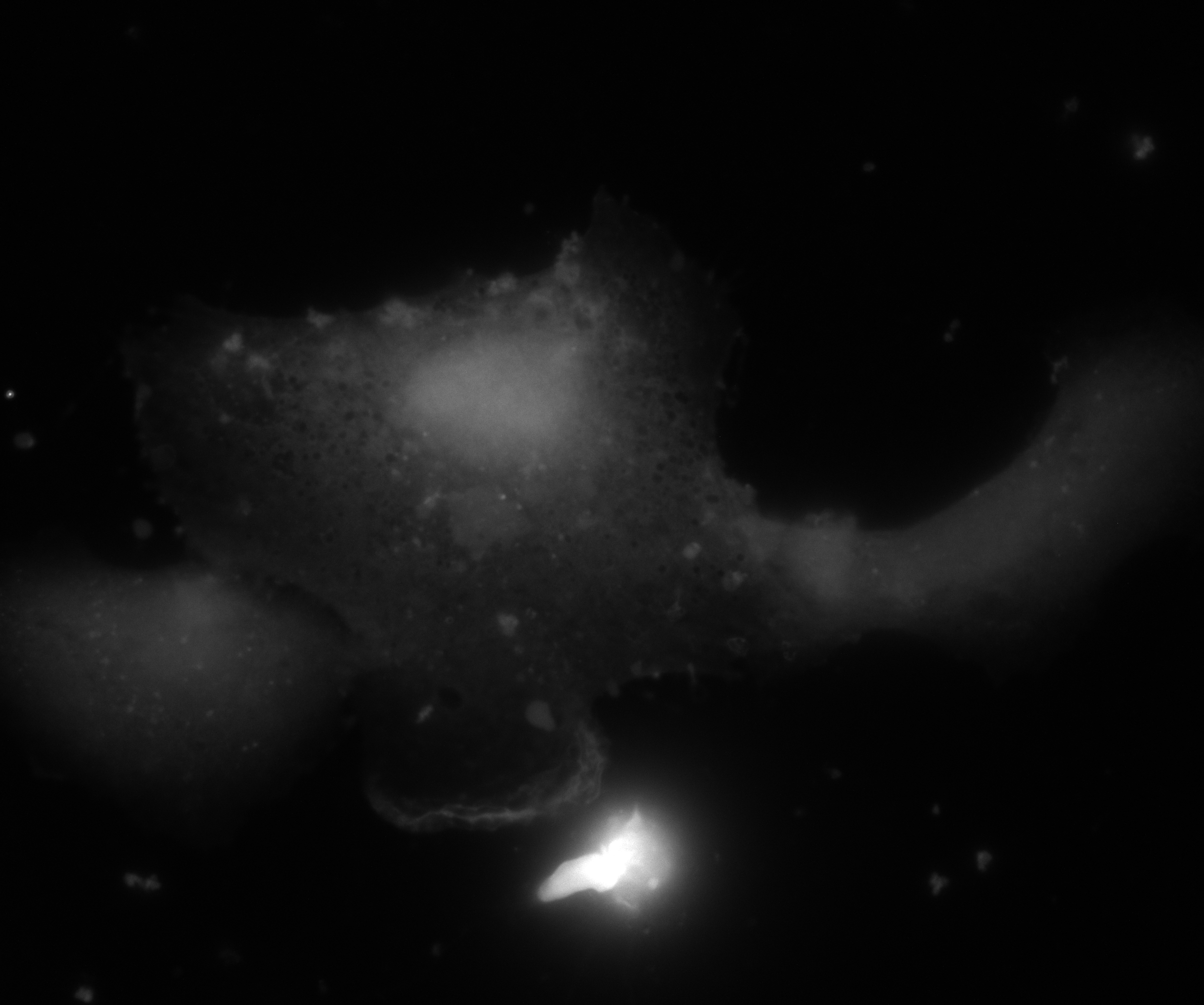

Supplement: Supplementary file 3 — Source data Fig. 1 [file 44321_2025_254_MOESM3_ESM.zip › Figure 1/Fig1H/RARAHT_Rhodamine.tif]

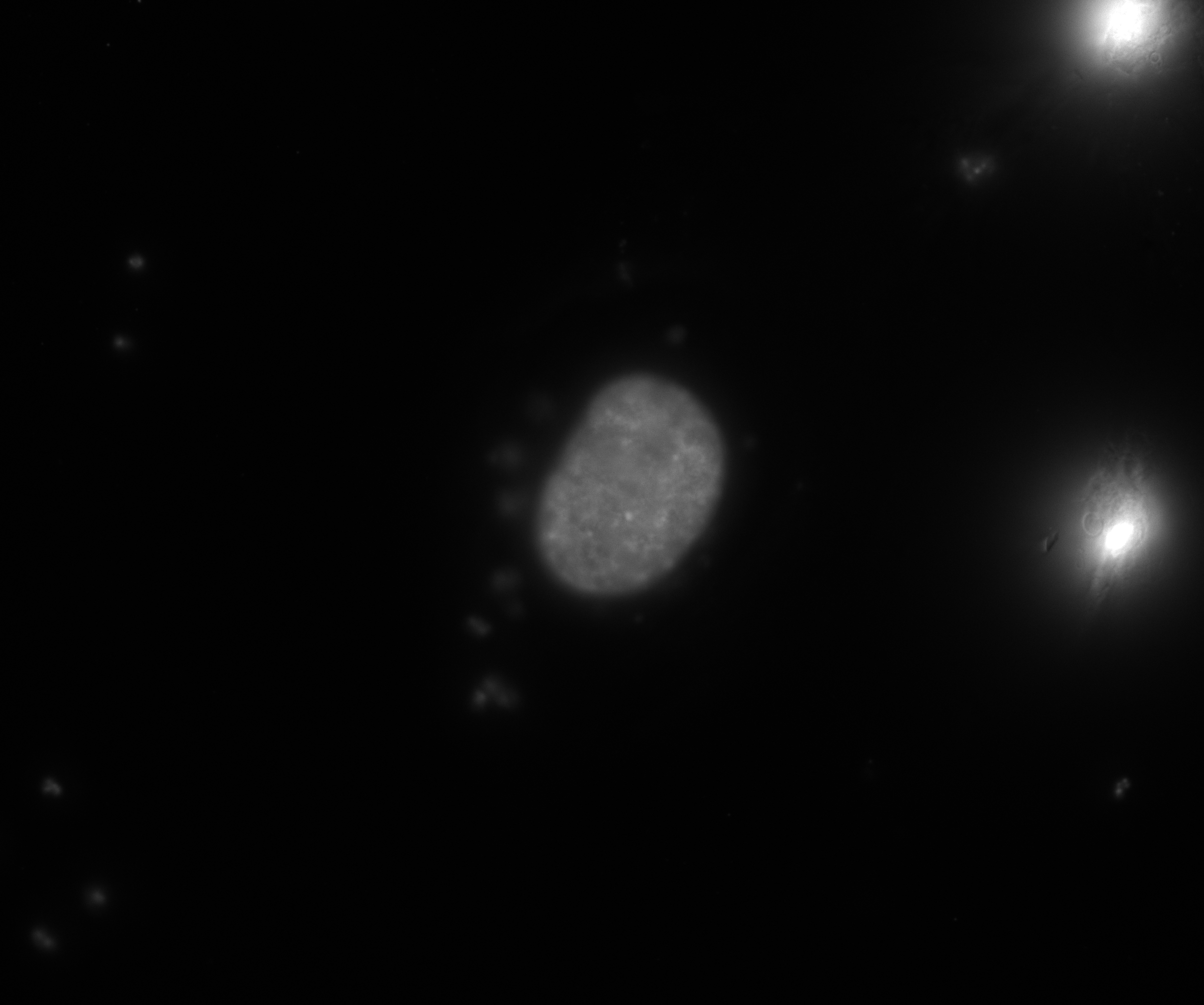

Supplement: Supplementary file 3 — Source data Fig. 1 [file 44321_2025_254_MOESM3_ESM.zip › Figure 1/Fig1H/WTRAR_DAPI.tif]

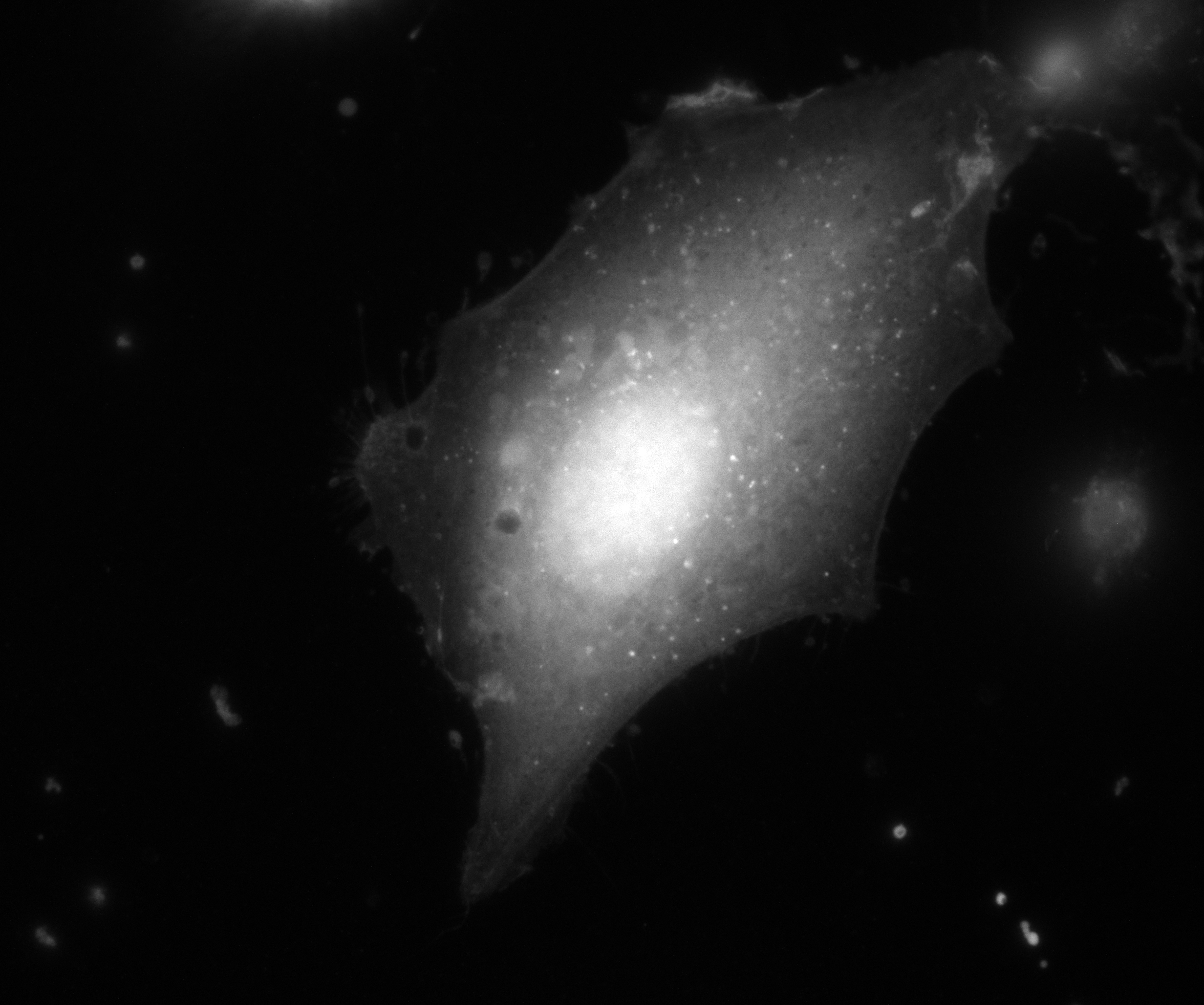

Supplement: Supplementary file 3 — Source data Fig. 1 [file 44321_2025_254_MOESM3_ESM.zip › Figure 1/Fig1H/WTRAR_Rhodamine.tif]

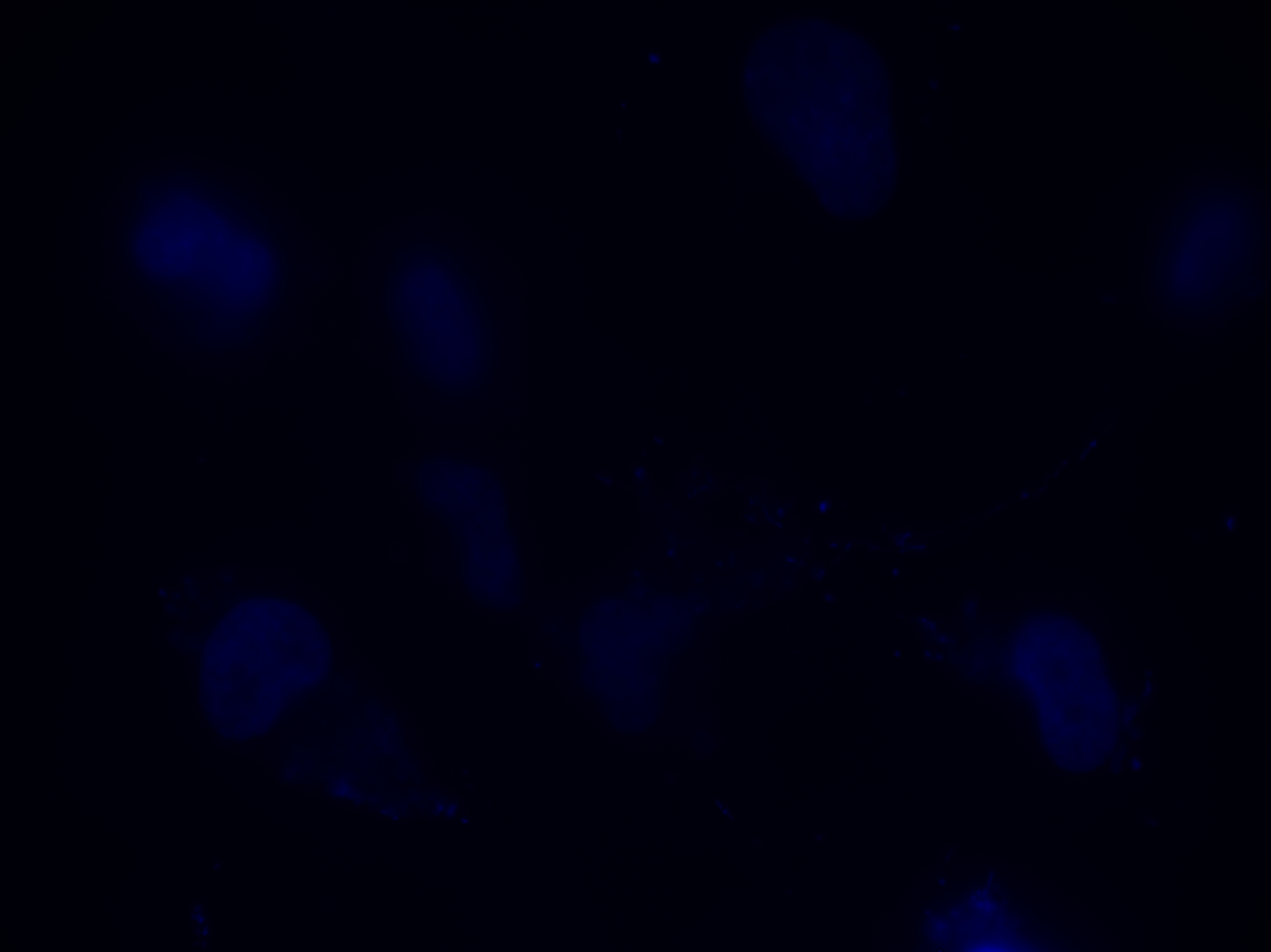

Supplement: Supplementary file 4 — Source data Fig. 2 [file 44321_2025_254_MOESM4_ESM.zip › Figure 2/2C/1 uM C11_DAPI.TIF]

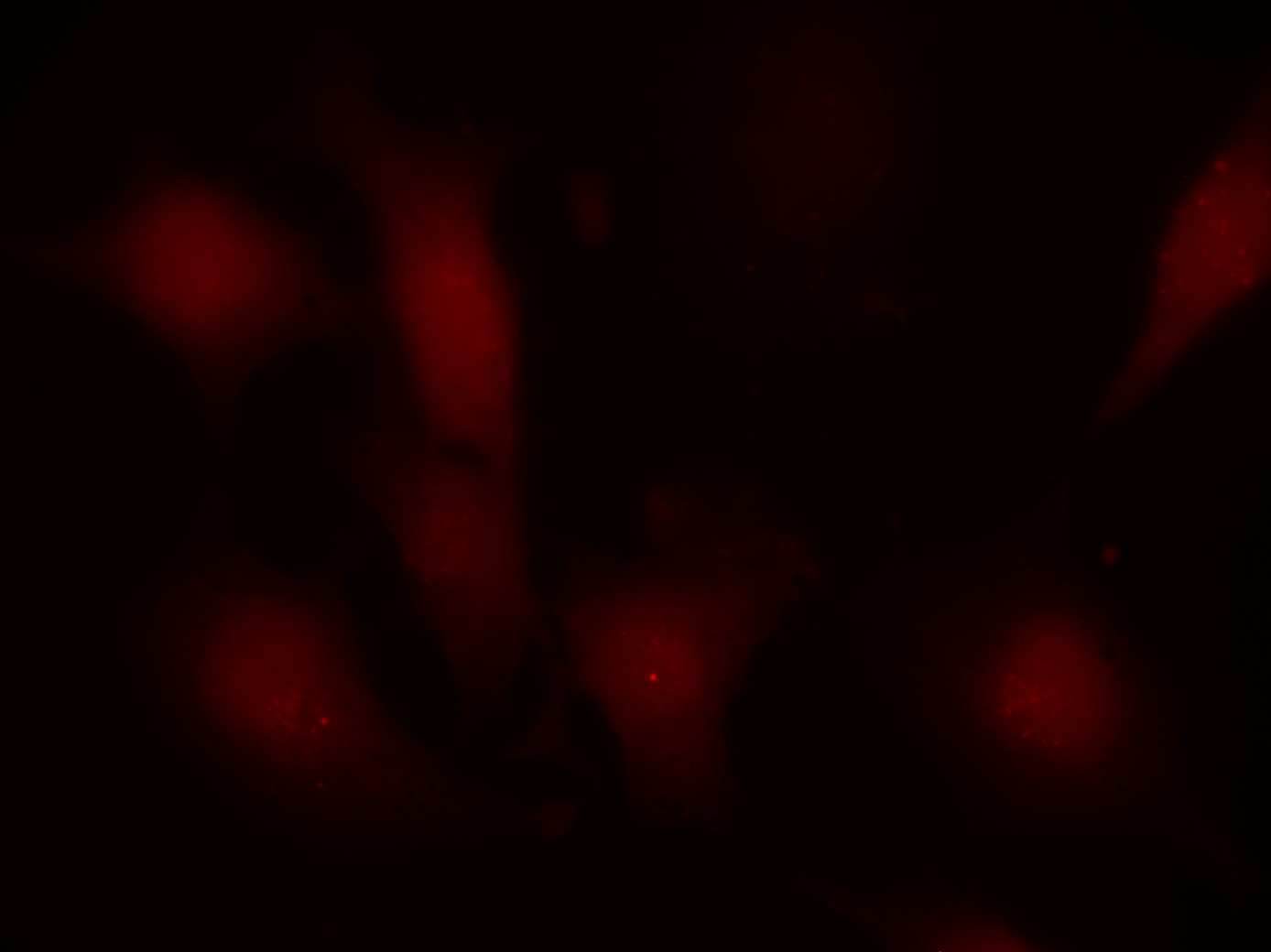

Supplement: Supplementary file 4 — Source data Fig. 2 [file 44321_2025_254_MOESM4_ESM.zip › Figure 2/2C/1 uM C11_Rhodamine.TIF]

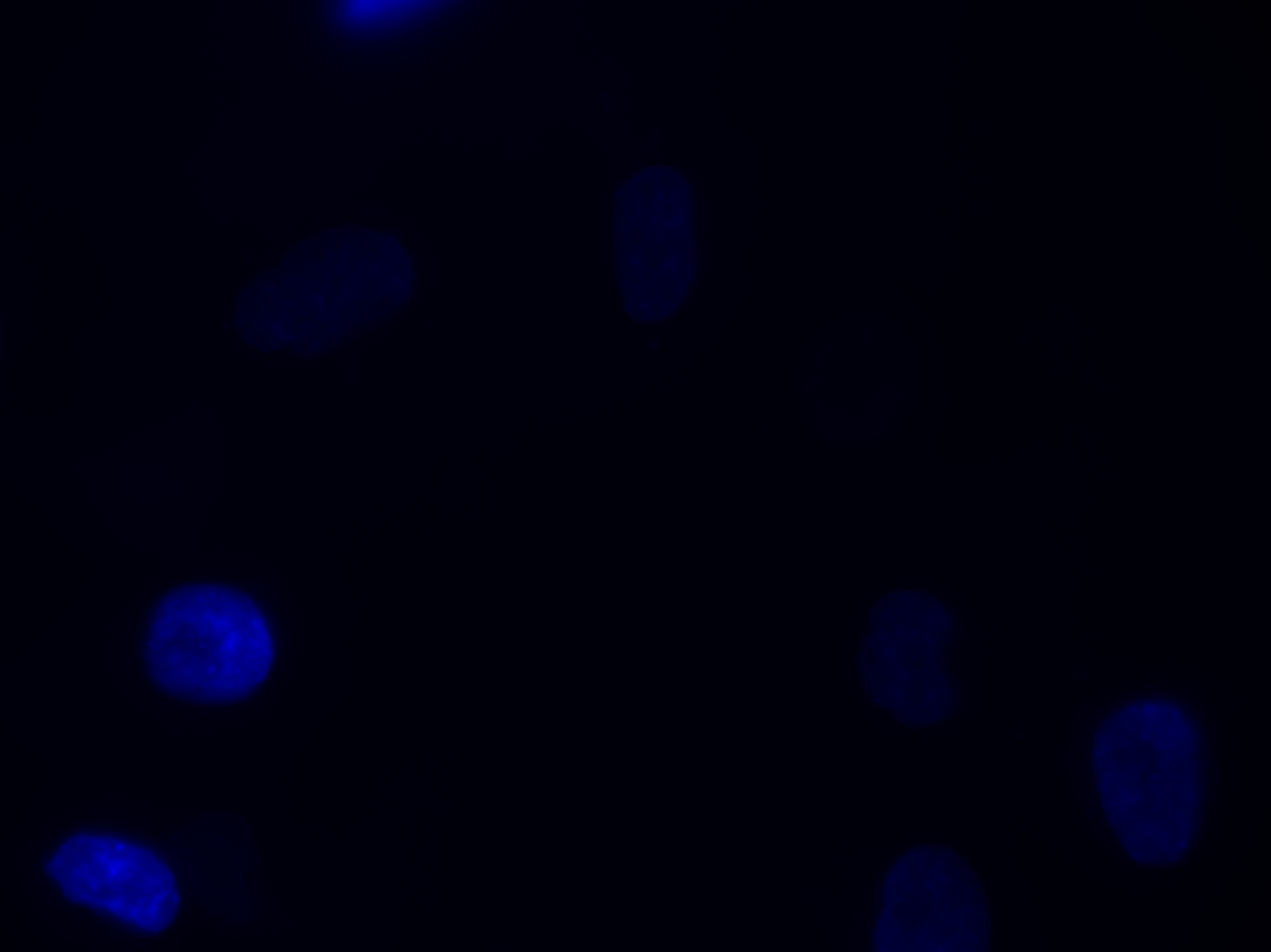

Supplement: Supplementary file 4 — Source data Fig. 2 [file 44321_2025_254_MOESM4_ESM.zip › Figure 2/2C/1 uM C8_DAPI.TIF]

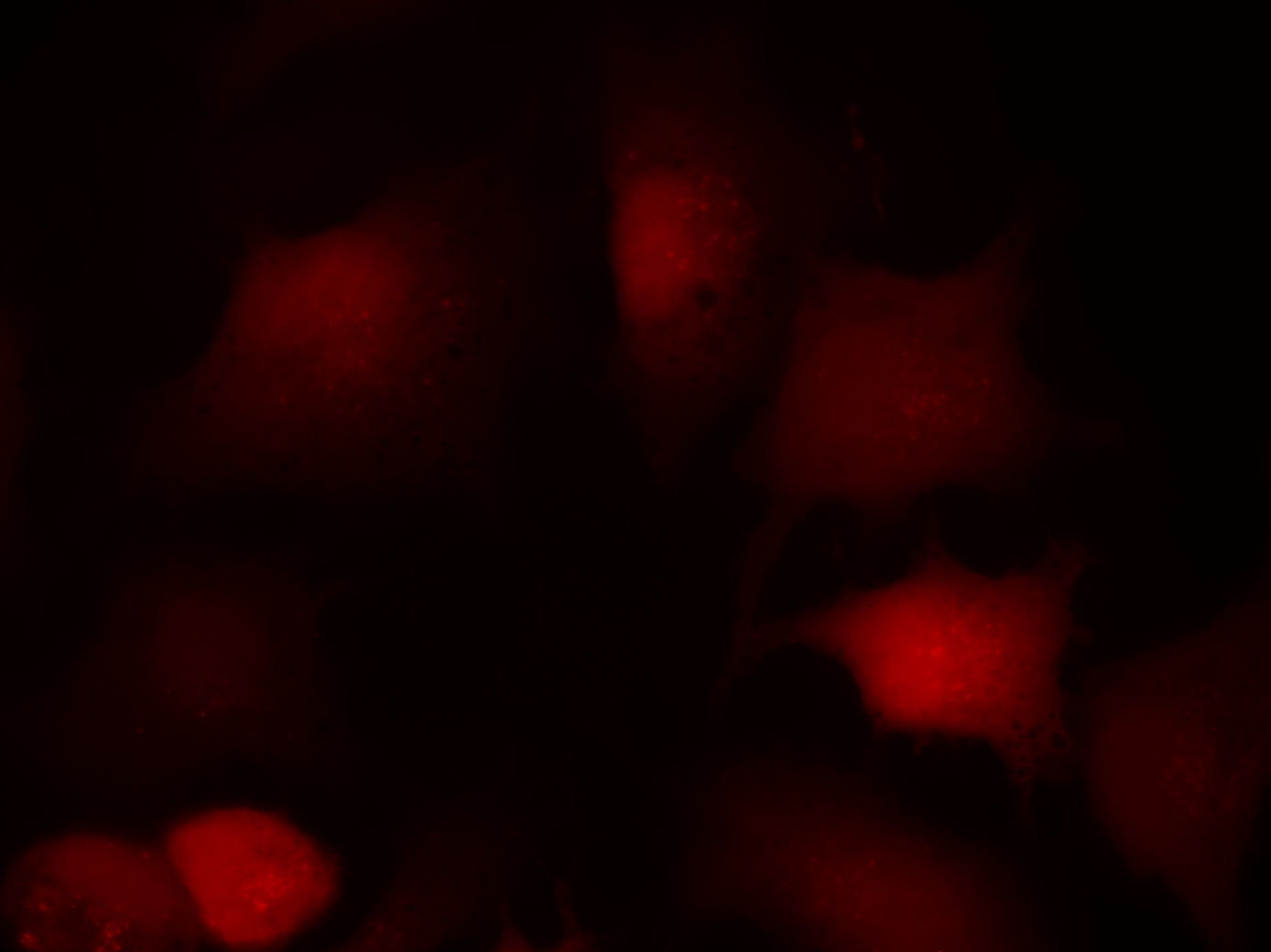

Supplement: Supplementary file 4 — Source data Fig. 2 [file 44321_2025_254_MOESM4_ESM.zip › Figure 2/2C/1 uM C8_Rhodamine.TIF]

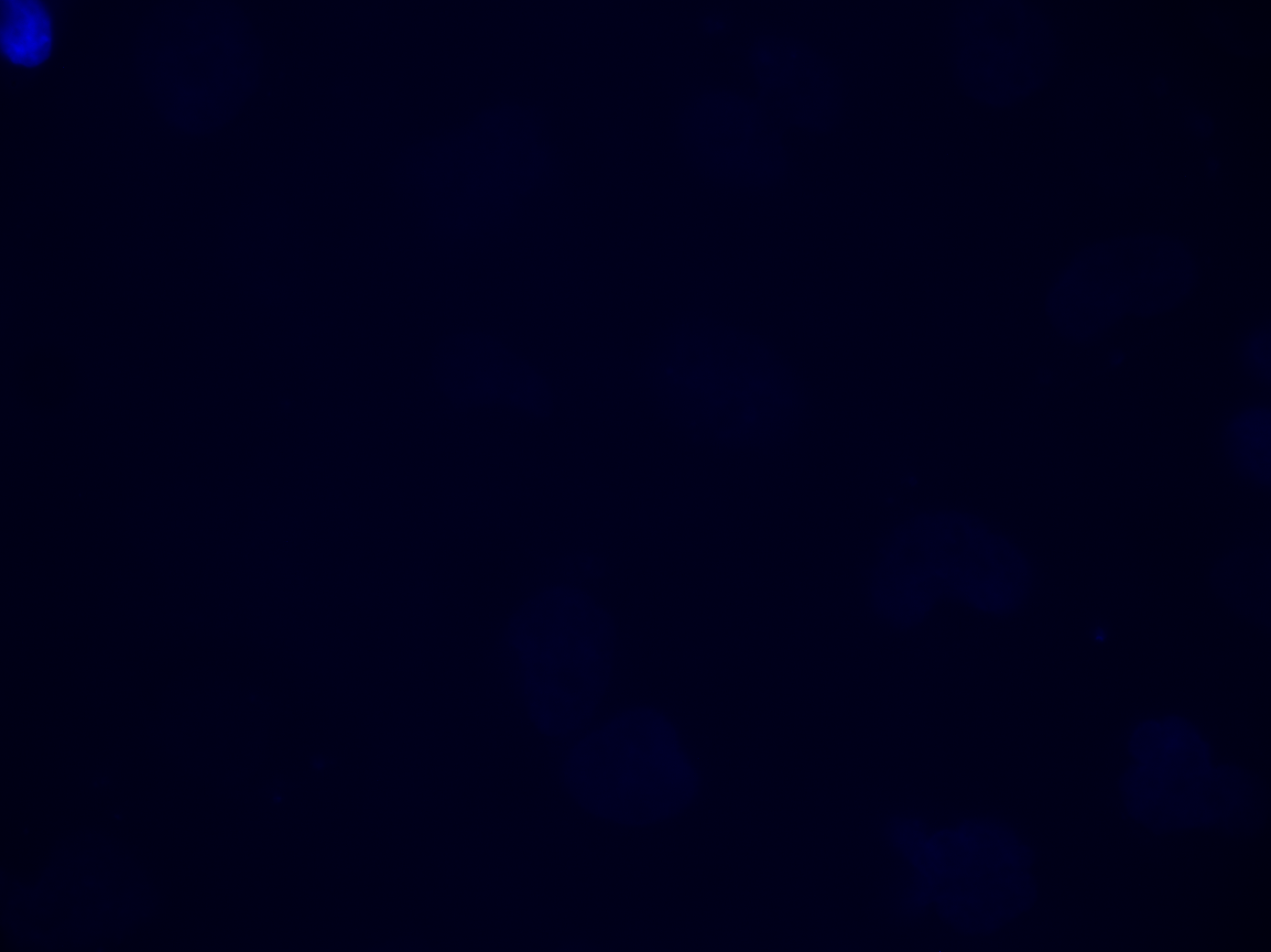

Supplement: Supplementary file 4 — Source data Fig. 2 [file 44321_2025_254_MOESM4_ESM.zip › Figure 2/2C/1 uM CIM7_DAPI.TIF]

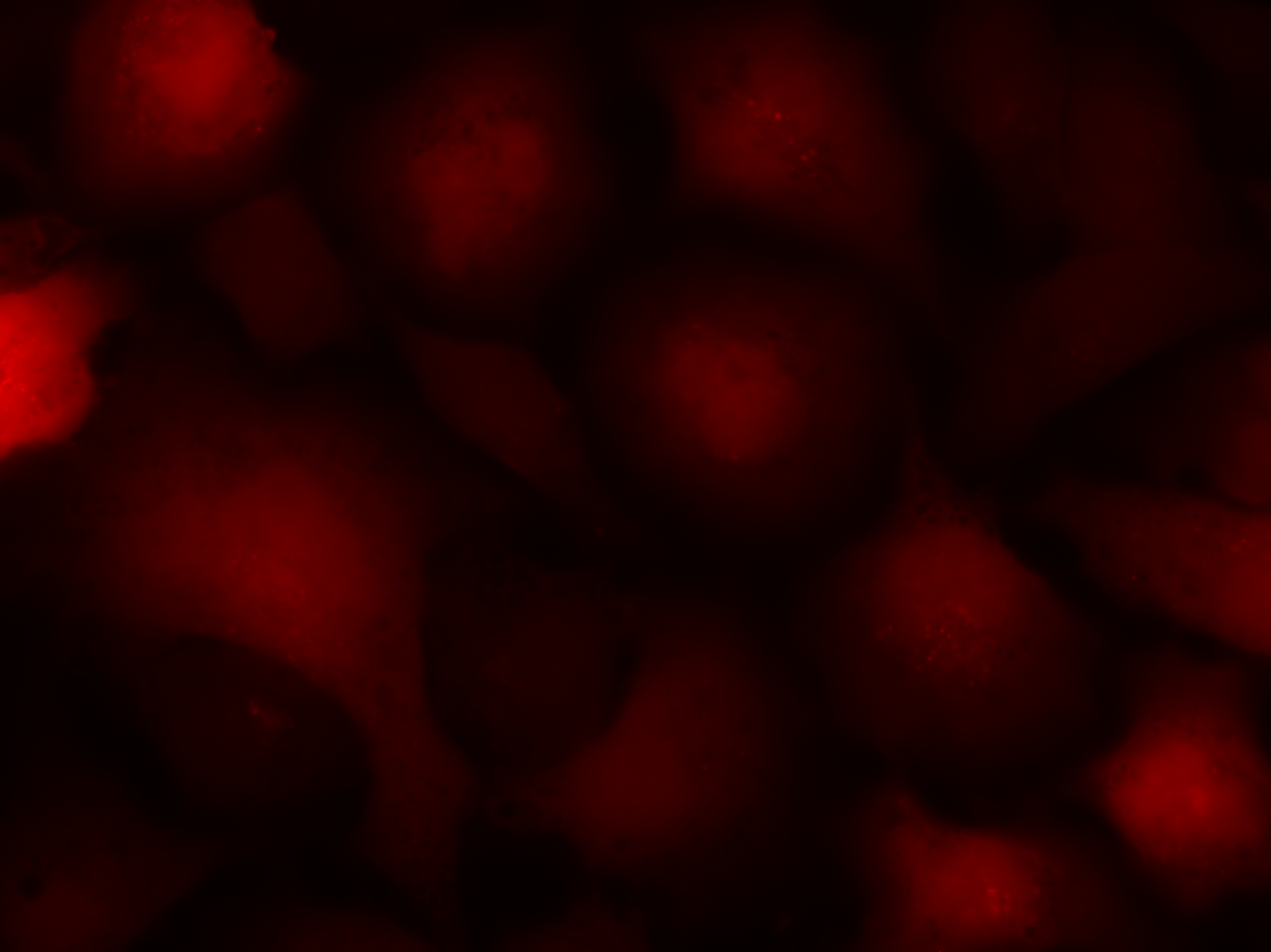

Supplement: Supplementary file 4 — Source data Fig. 2 [file 44321_2025_254_MOESM4_ESM.zip › Figure 2/2C/1 uM CIM7_Rhodamine.TIF]

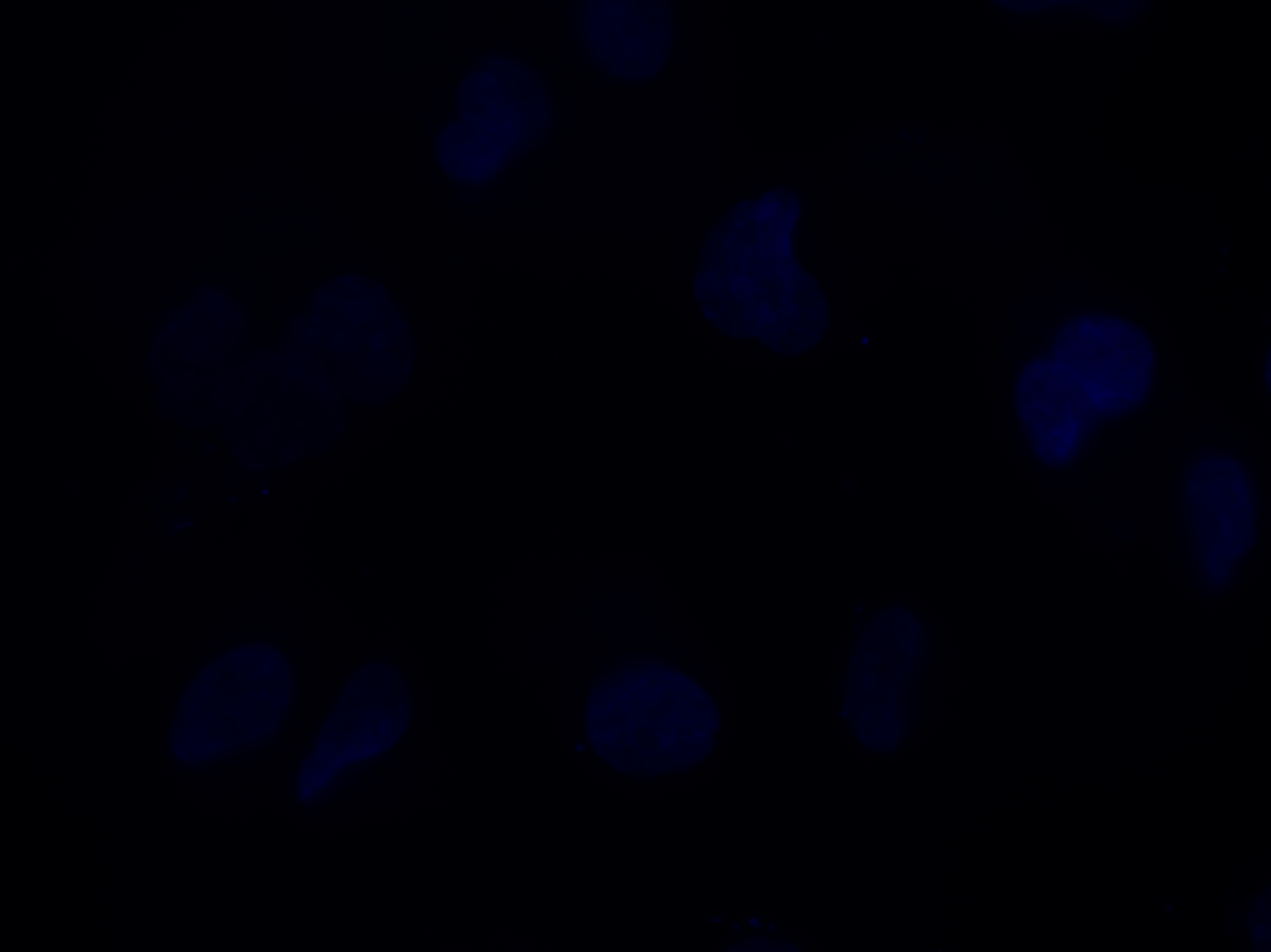

Supplement: Supplementary file 4 — Source data Fig. 2 [file 44321_2025_254_MOESM4_ESM.zip › Figure 2/2C/1 uM D11_DAPI.TIF]

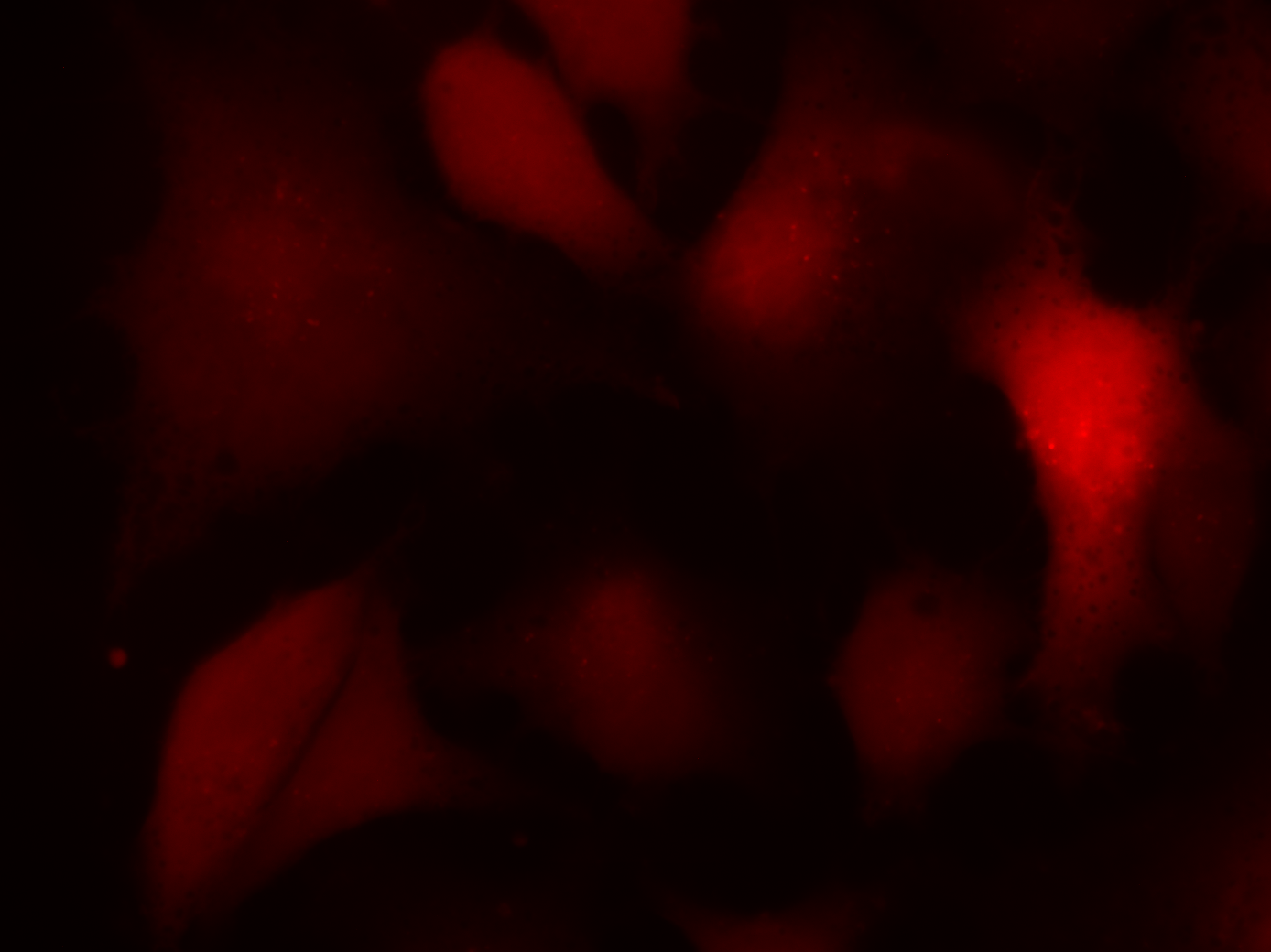

Supplement: Supplementary file 4 — Source data Fig. 2 [file 44321_2025_254_MOESM4_ESM.zip › Figure 2/2C/1 uM D11_Rhodamine.TIF]

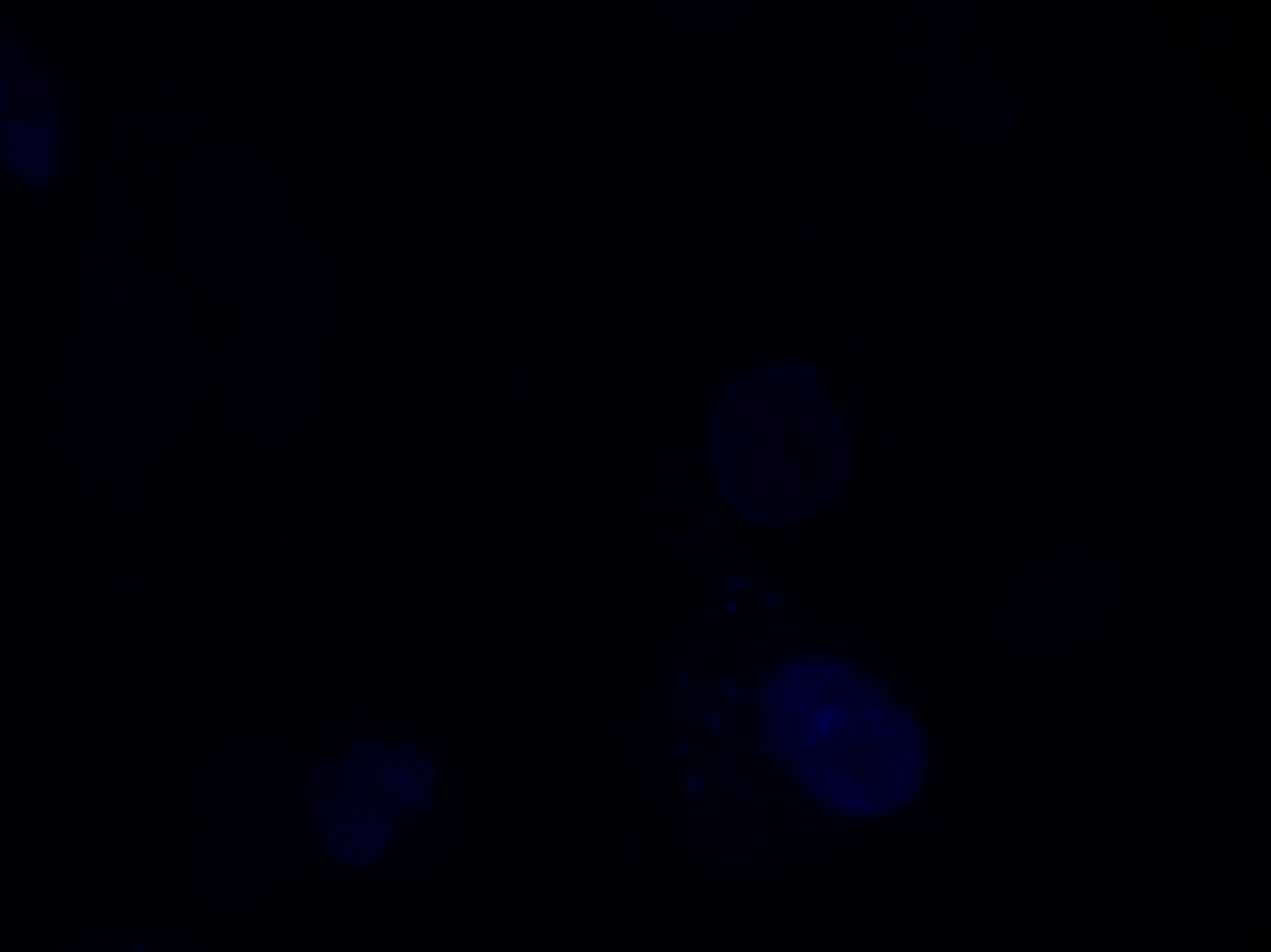

Supplement: Supplementary file 4 — Source data Fig. 2 [file 44321_2025_254_MOESM4_ESM.zip › Figure 2/2C/1 uM D12_DAPI.TIF]

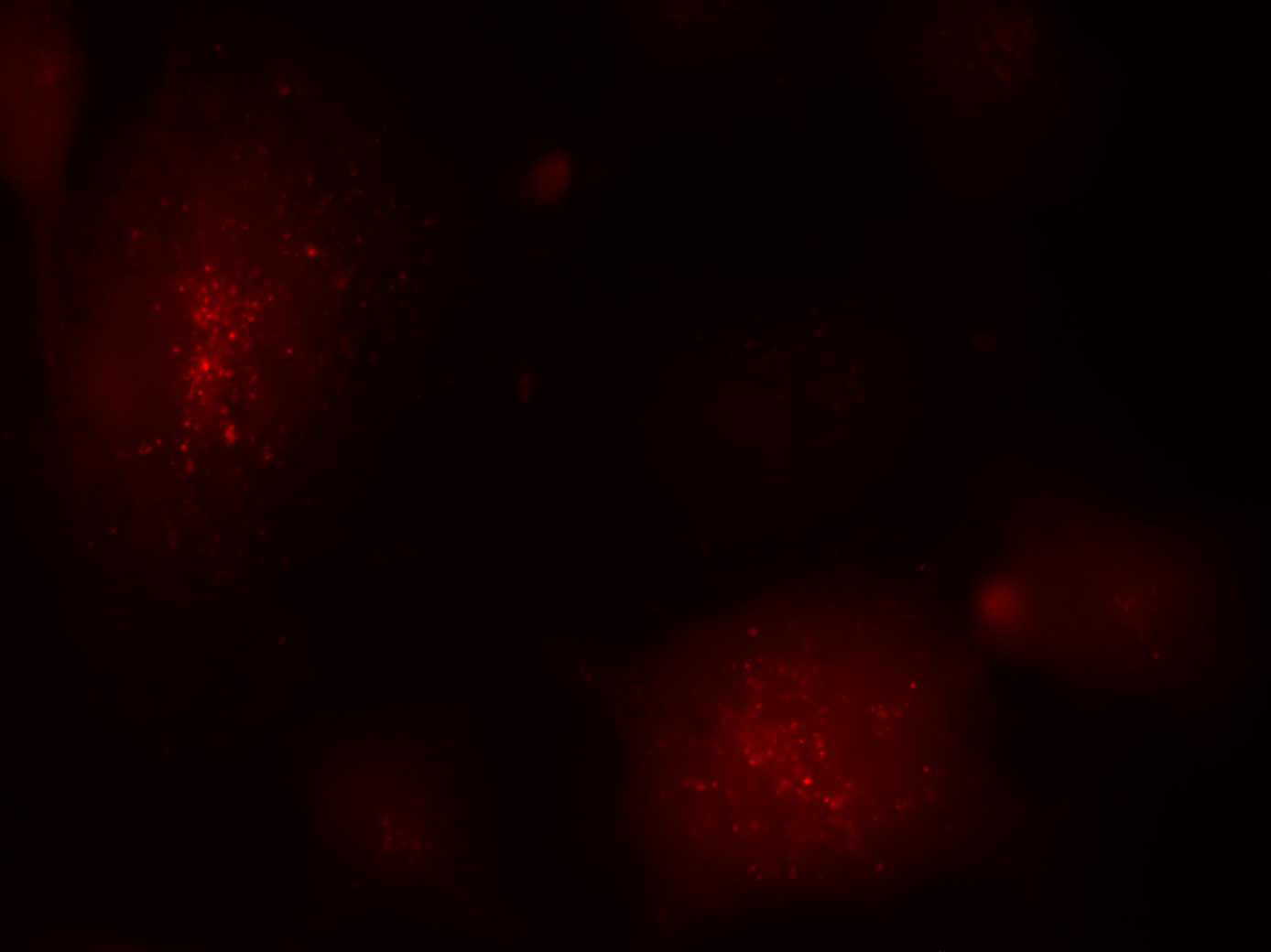

Supplement: Supplementary file 4 — Source data Fig. 2 [file 44321_2025_254_MOESM4_ESM.zip › Figure 2/2C/1 uM D12_Rhodamine.TIF]

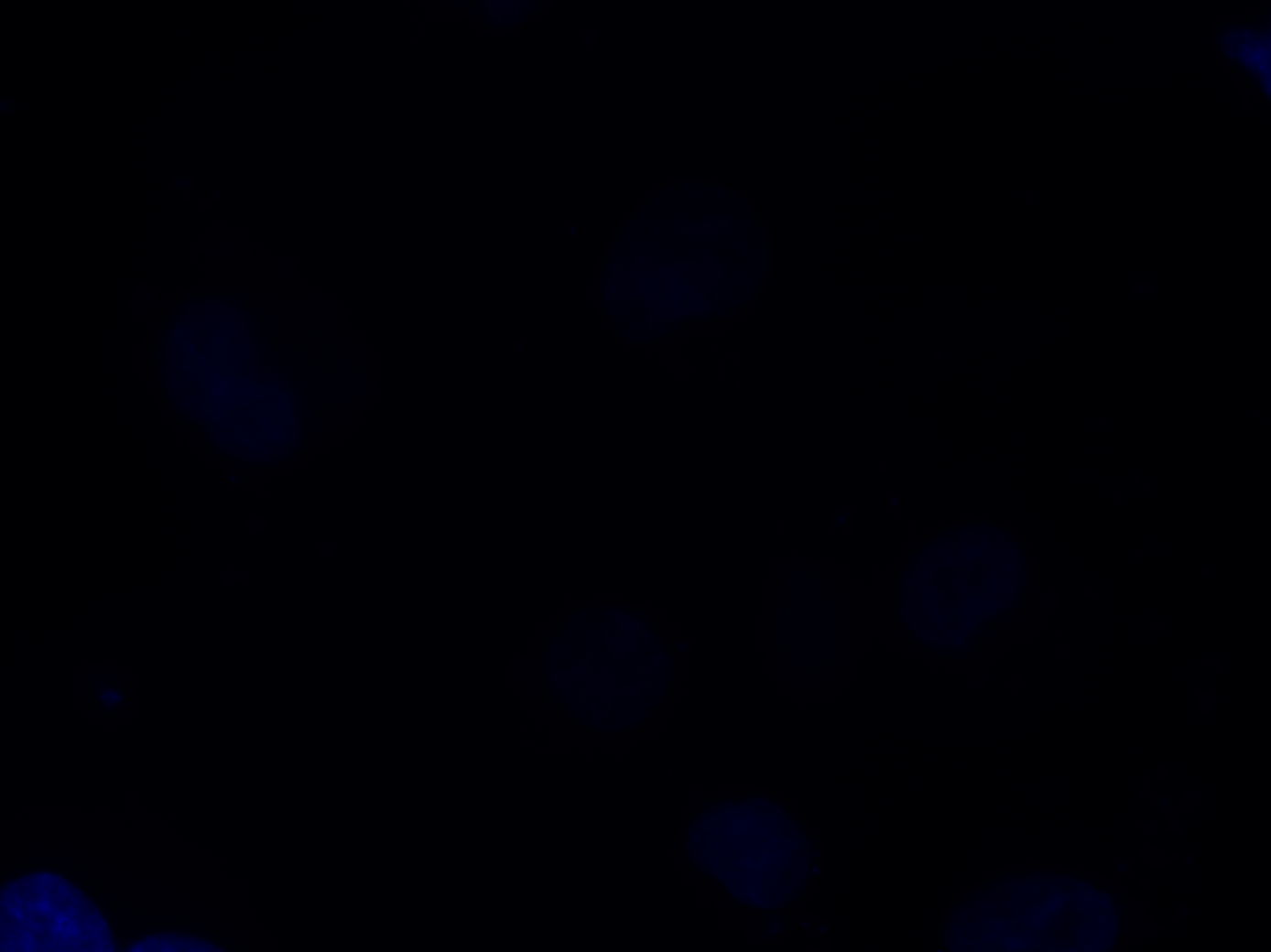

Supplement: Supplementary file 4 — Source data Fig. 2 [file 44321_2025_254_MOESM4_ESM.zip › Figure 2/2C/1 uM G11_DAPI.TIF]

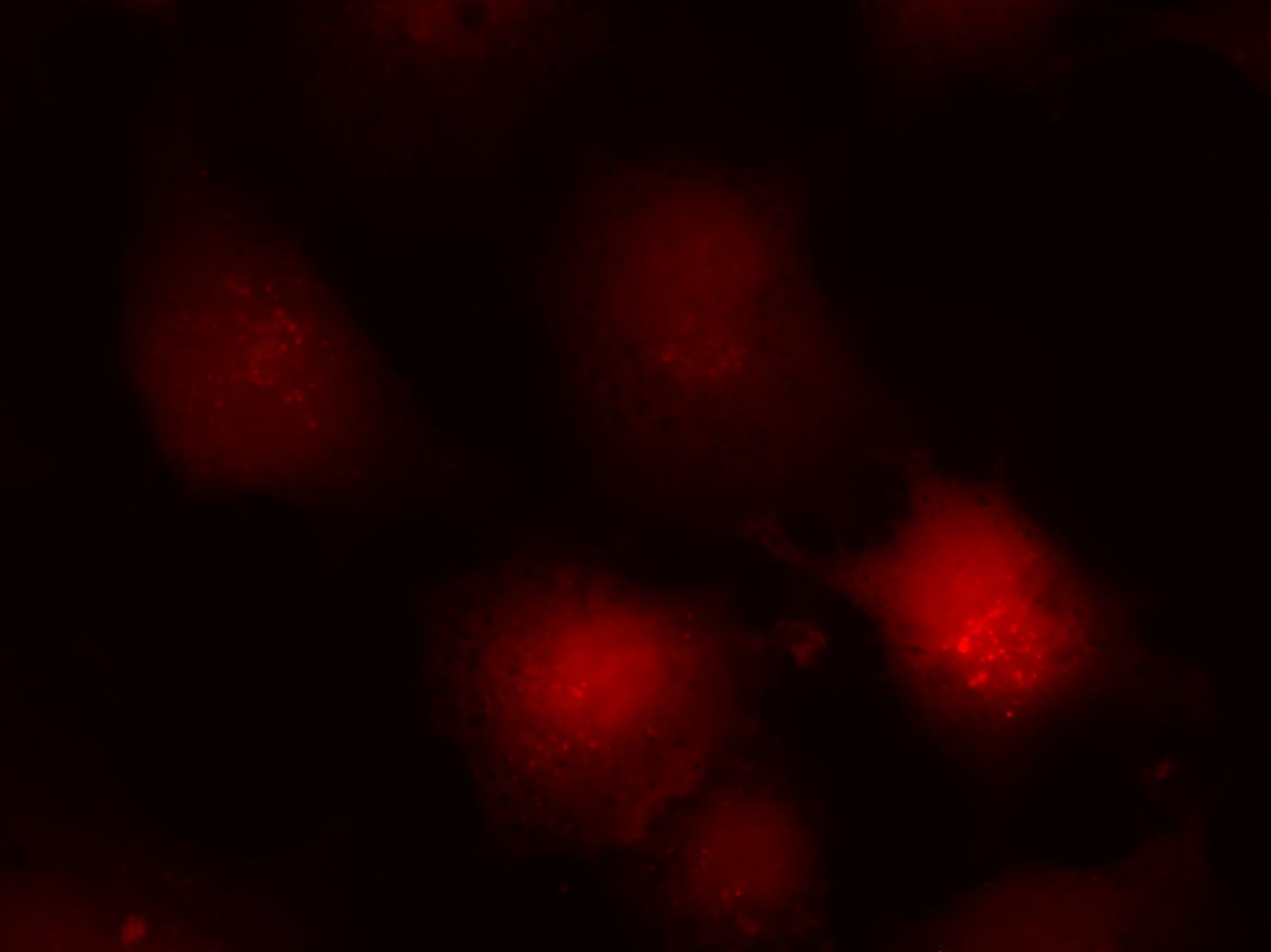

Supplement: Supplementary file 4 — Source data Fig. 2 [file 44321_2025_254_MOESM4_ESM.zip › Figure 2/2C/1 uM G11_Rhodamine.TIF]

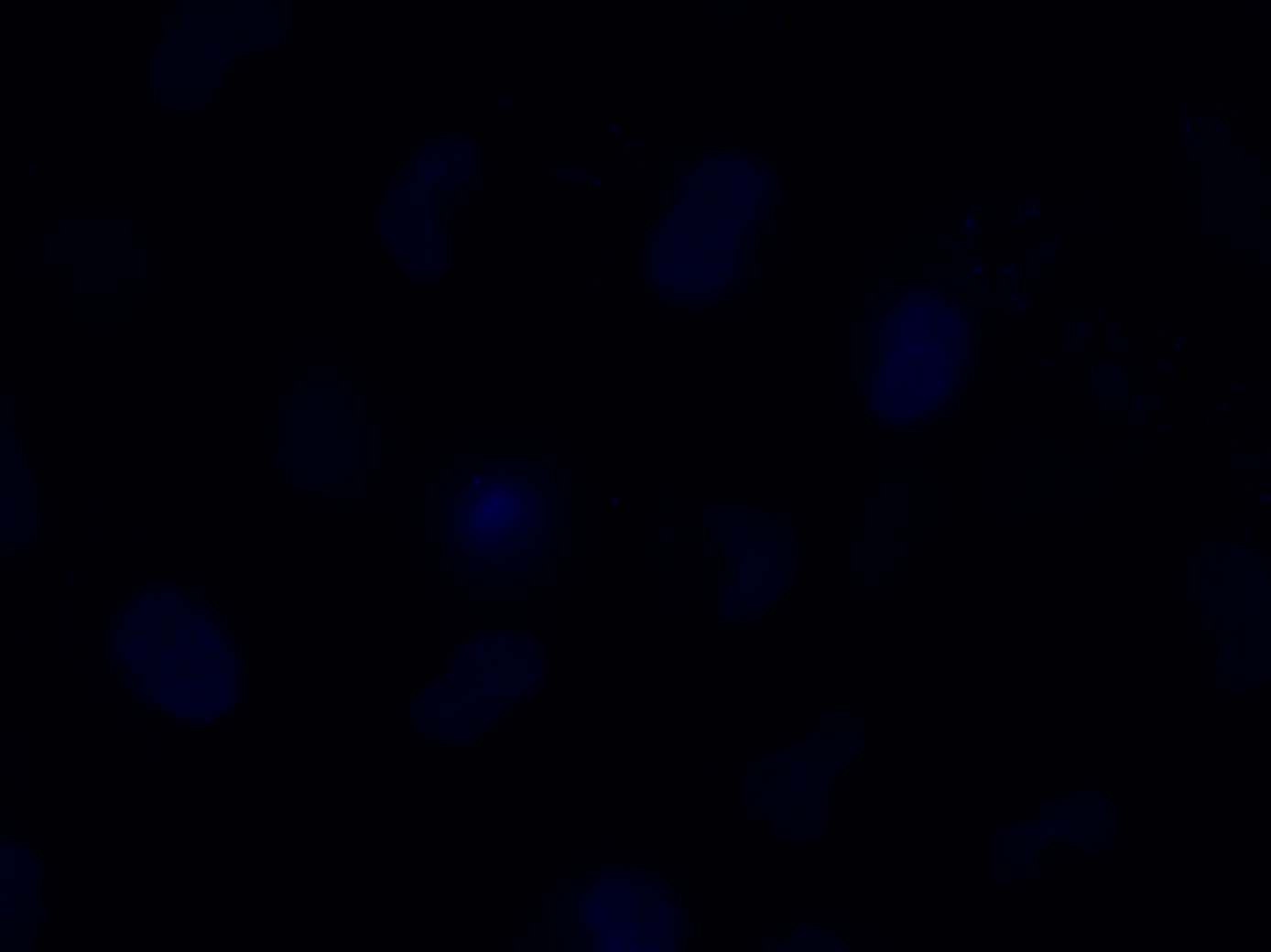

Supplement: Supplementary file 4 — Source data Fig. 2 [file 44321_2025_254_MOESM4_ESM.zip › Figure 2/2C/DMSO_DAPI.TIF]

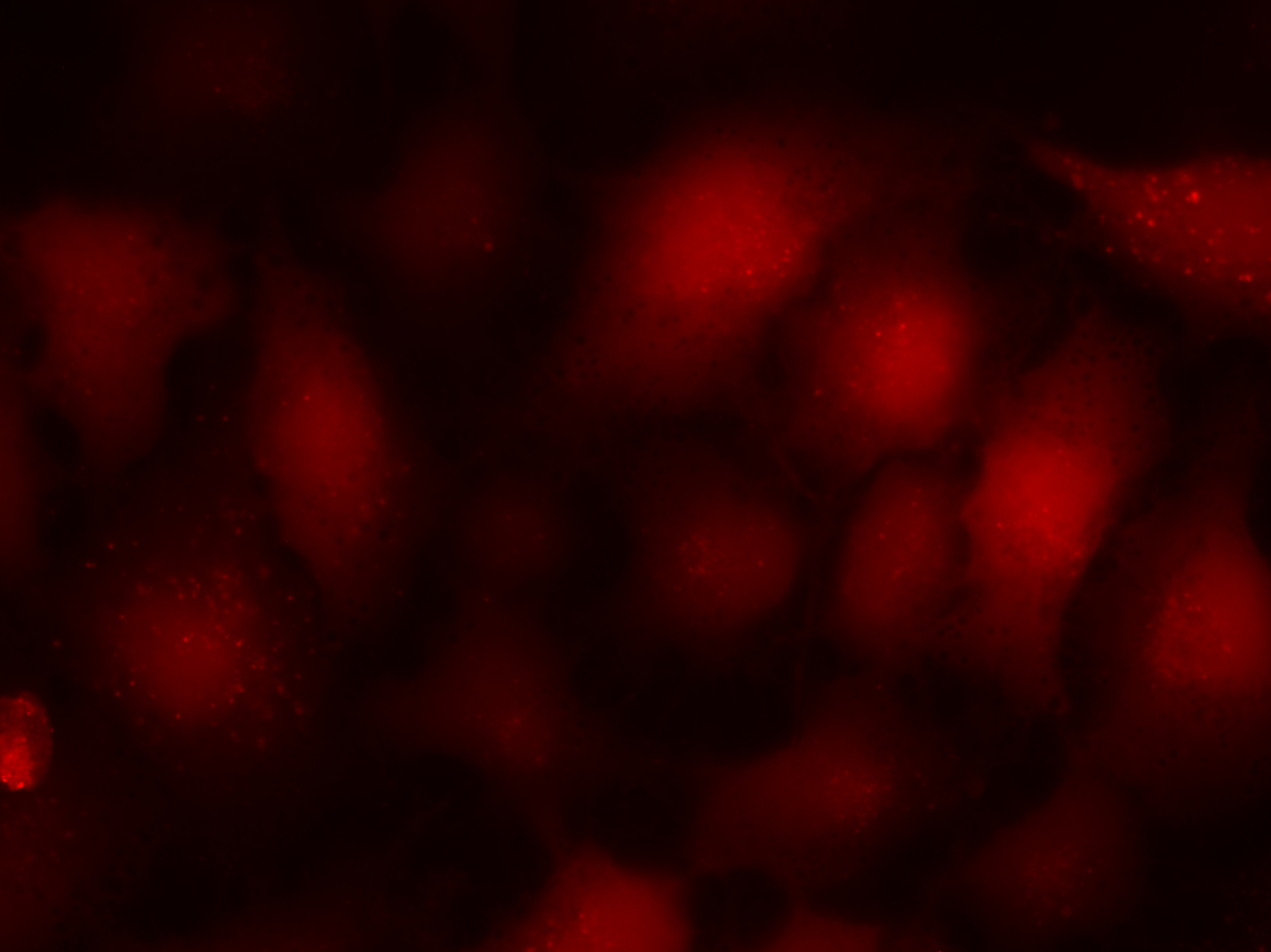

Supplement: Supplementary file 4 — Source data Fig. 2 [file 44321_2025_254_MOESM4_ESM.zip › Figure 2/2C/DMSO_Rhodamine.TIF]

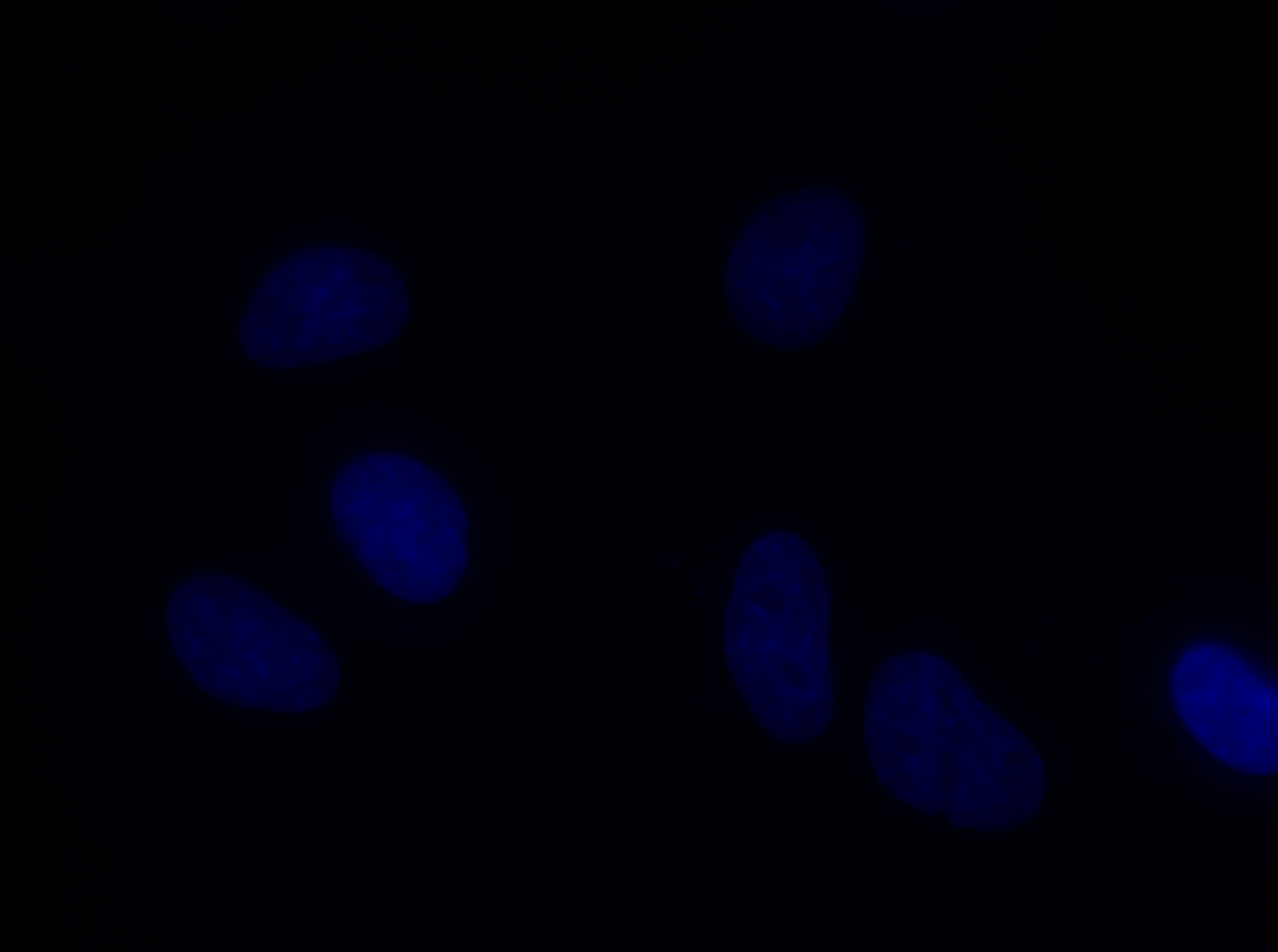

Supplement: Supplementary file 4 — Source data Fig. 2 [file 44321_2025_254_MOESM4_ESM.zip › Figure 2/2E/A549-1000nMCIM7_DAPI.TIF]

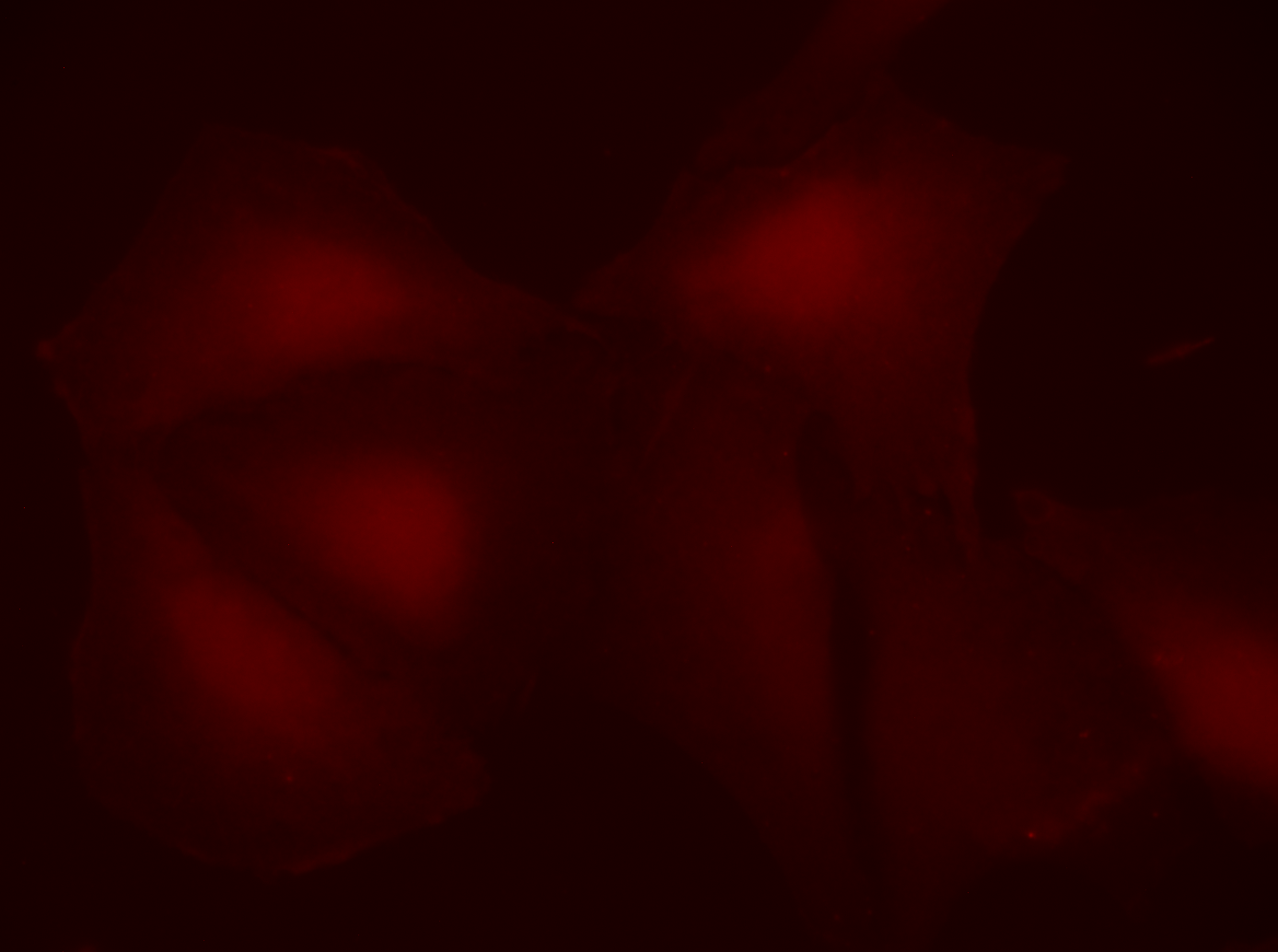

Supplement: Supplementary file 4 — Source data Fig. 2 [file 44321_2025_254_MOESM4_ESM.zip › Figure 2/2E/A549-1000nMCIM7_Rhodamine.TIF]

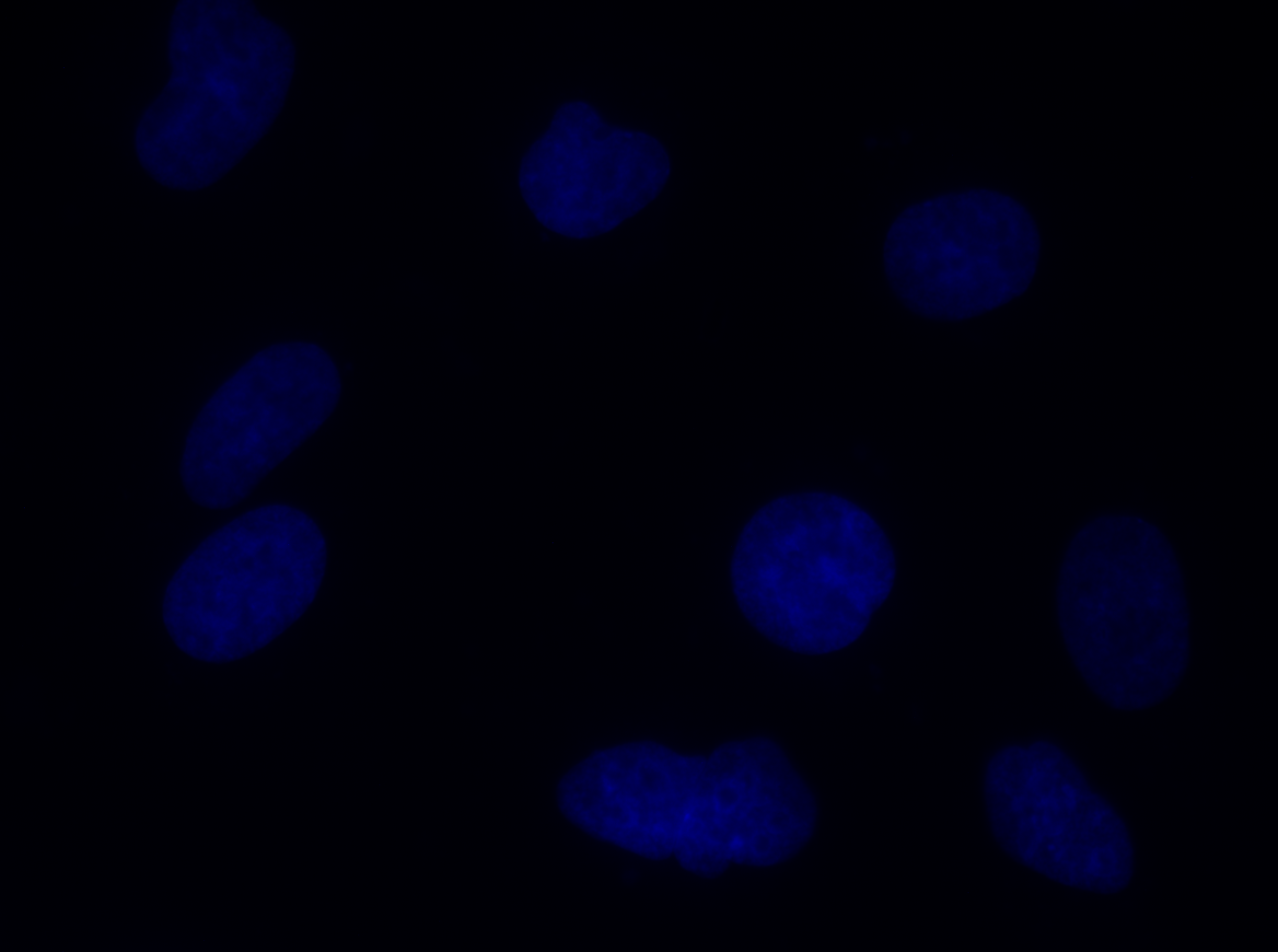

Supplement: Supplementary file 4 — Source data Fig. 2 [file 44321_2025_254_MOESM4_ESM.zip › Figure 2/2E/A549-250nMCIM7_DAPI.TIF]

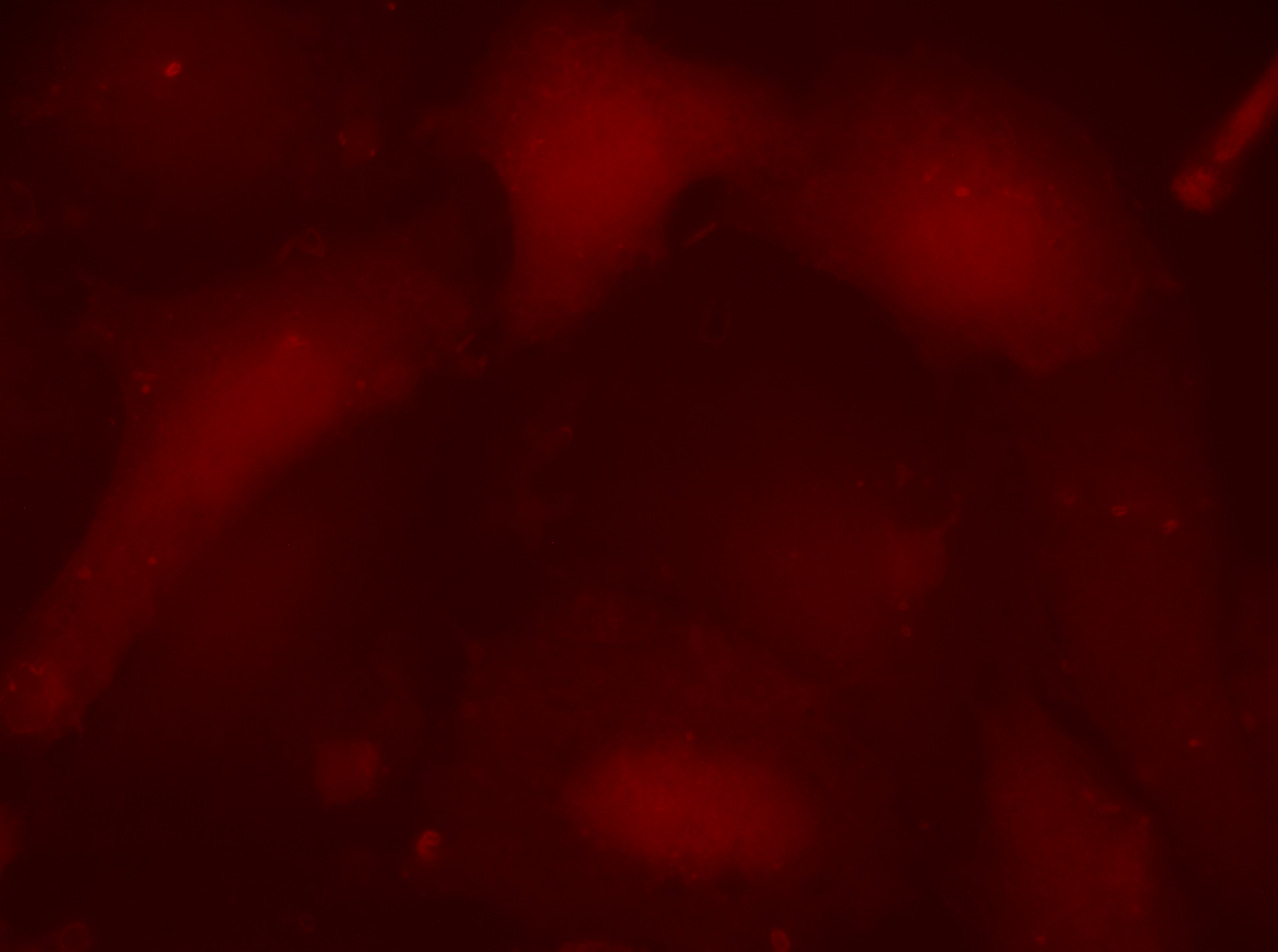

Supplement: Supplementary file 4 — Source data Fig. 2 [file 44321_2025_254_MOESM4_ESM.zip › Figure 2/2E/A549-250nMCIM7_Rhodamine.TIF]

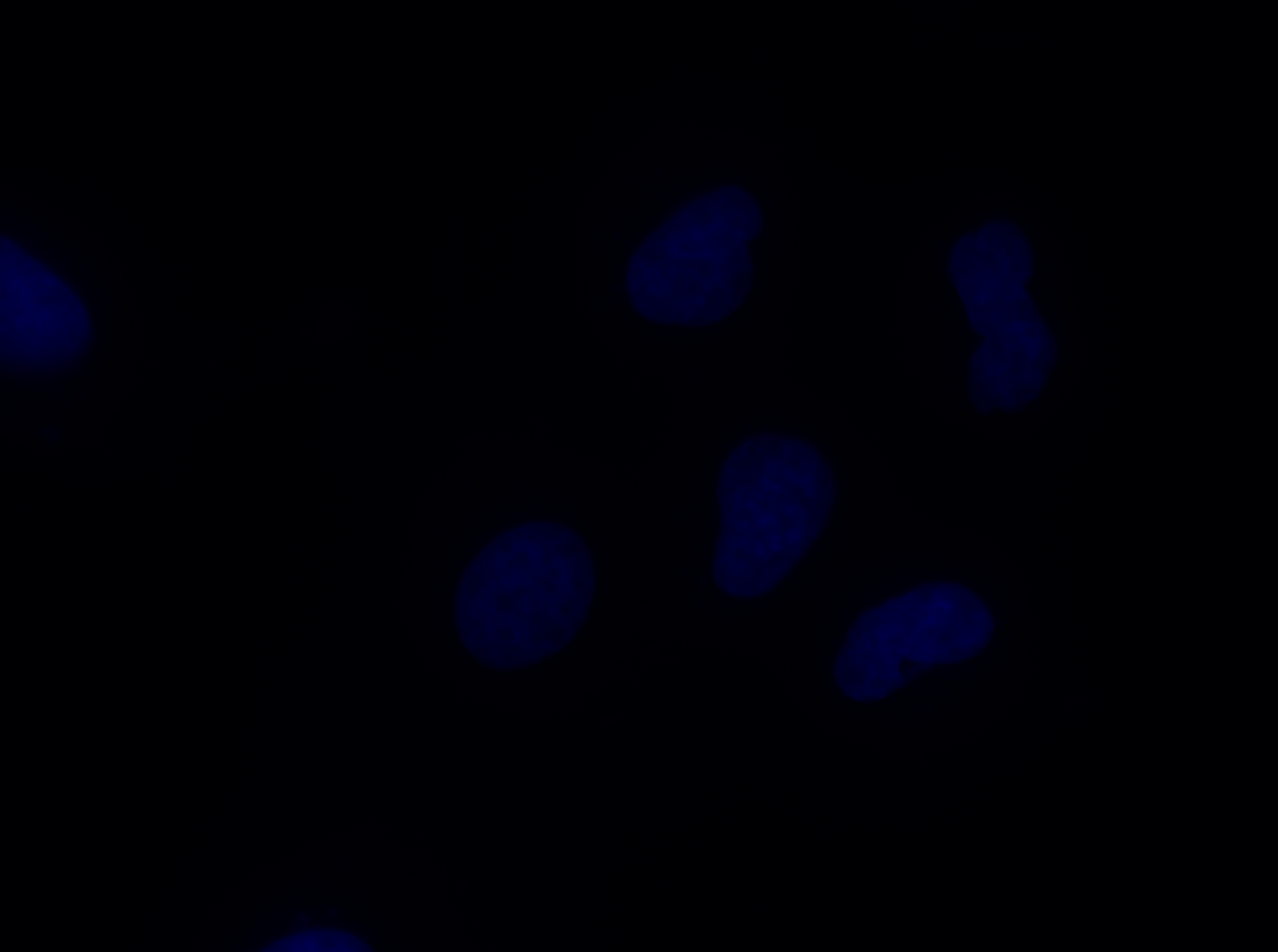

Supplement: Supplementary file 4 — Source data Fig. 2 [file 44321_2025_254_MOESM4_ESM.zip › Figure 2/2E/A549-500nMCIM7_DAPI.TIF]

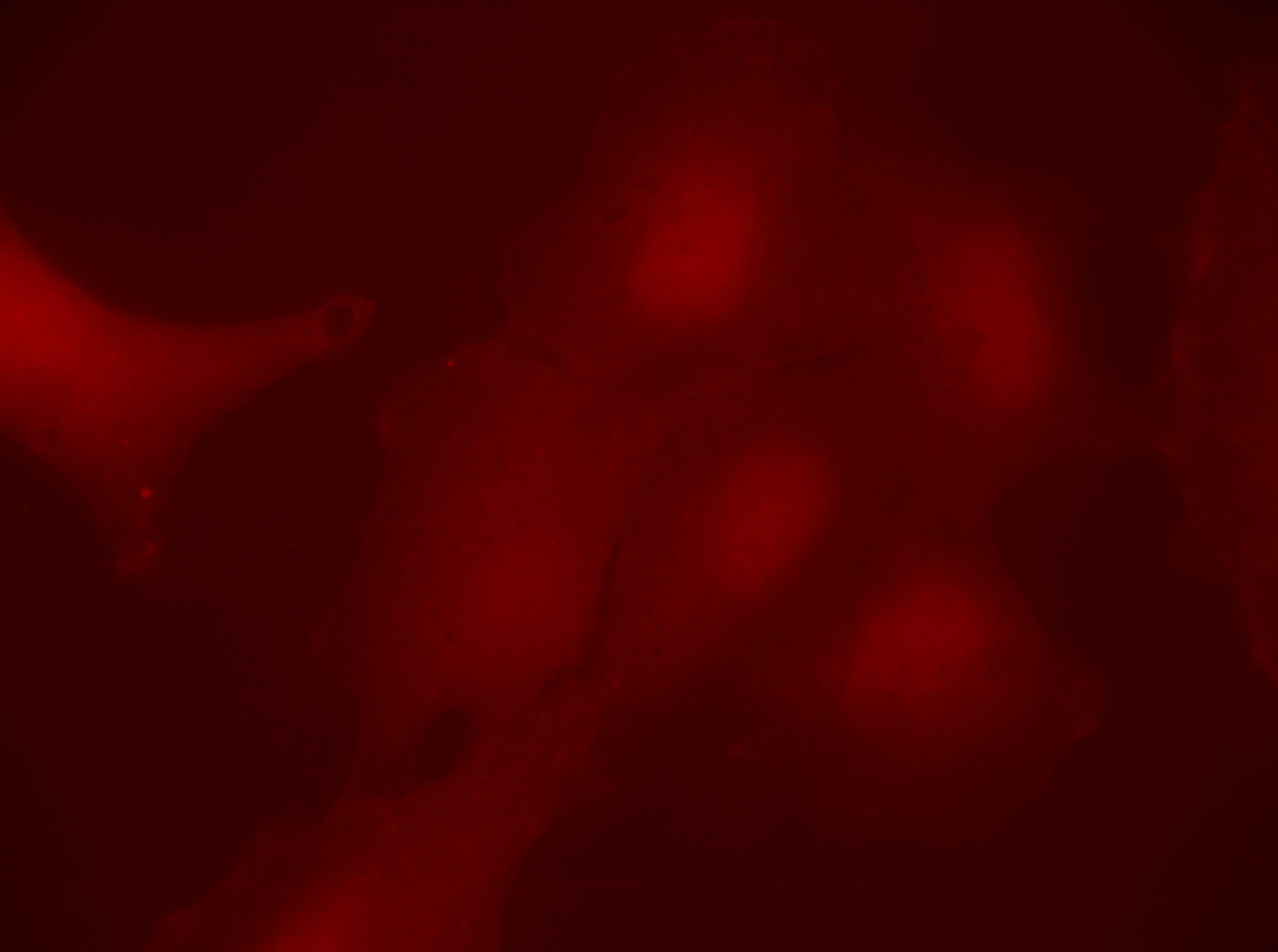

Supplement: Supplementary file 4 — Source data Fig. 2 [file 44321_2025_254_MOESM4_ESM.zip › Figure 2/2E/A549-500nMCIM7_Rhodamine.TIF]

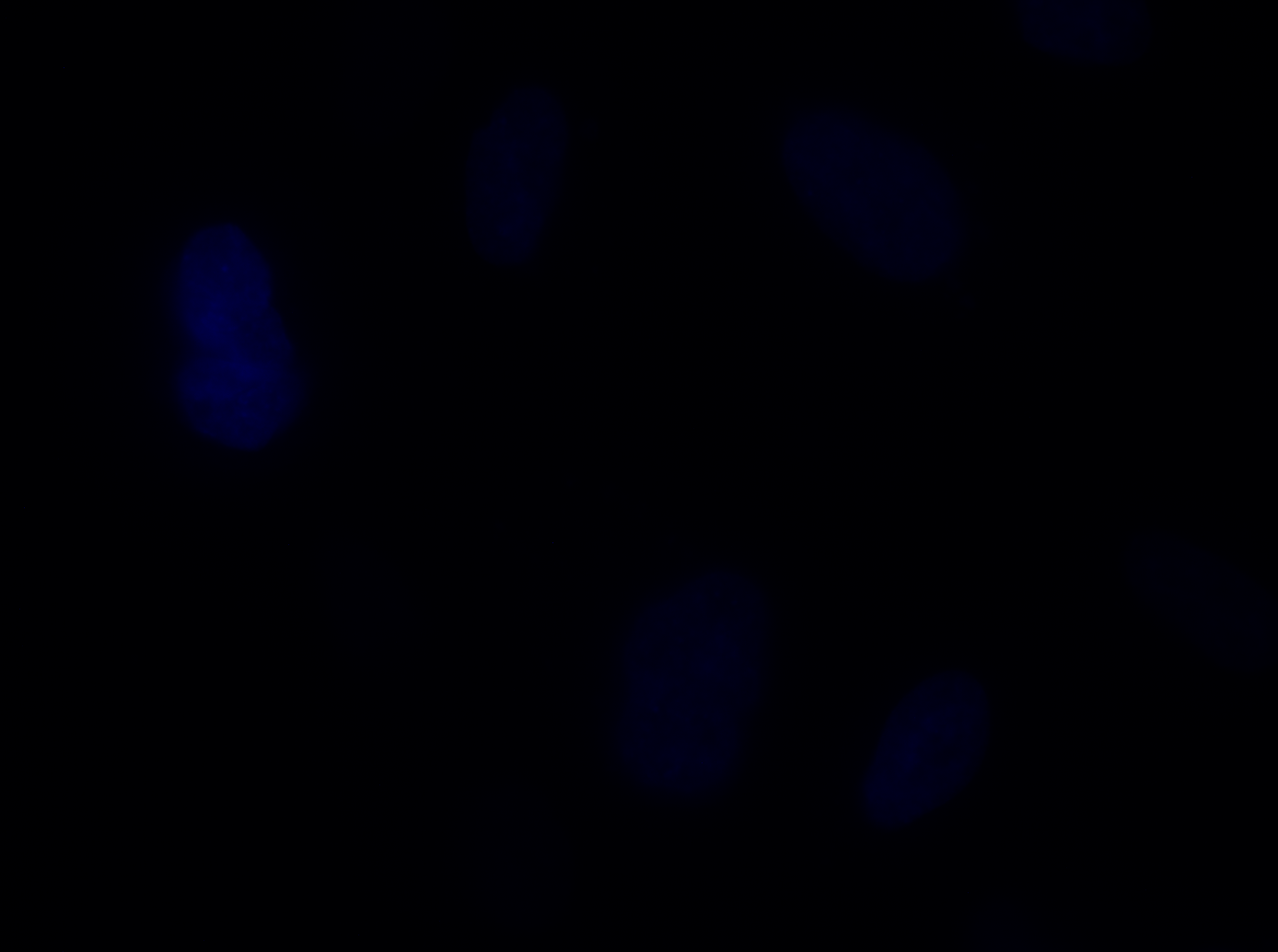

Supplement: Supplementary file 4 — Source data Fig. 2 [file 44321_2025_254_MOESM4_ESM.zip › Figure 2/2E/A549-DMSO_DAPI.TIF]

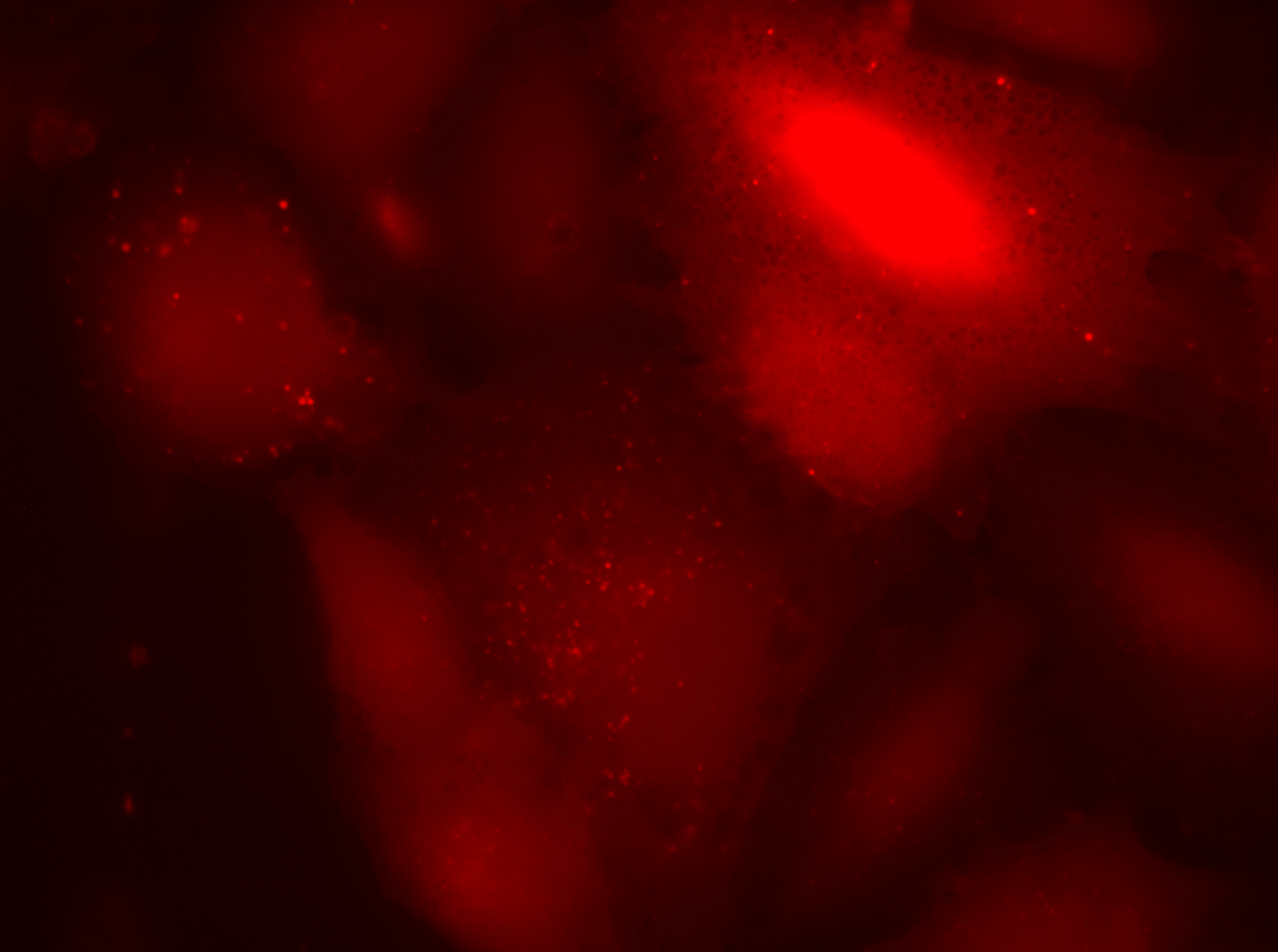

Supplement: Supplementary file 4 — Source data Fig. 2 [file 44321_2025_254_MOESM4_ESM.zip › Figure 2/2E/A549-DMSO_Rhodamine.TIF]

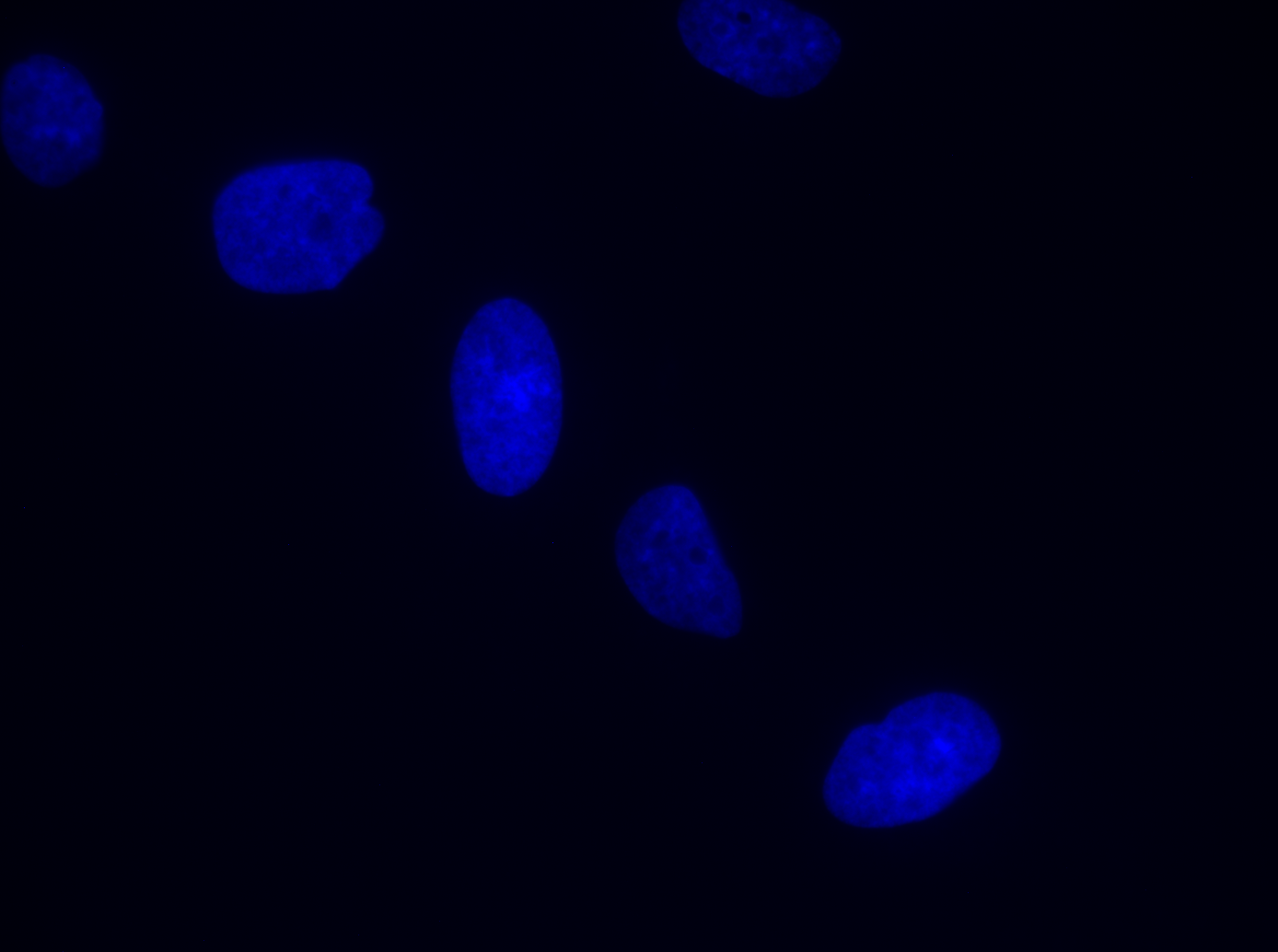

Supplement: Supplementary file 4 — Source data Fig. 2 [file 44321_2025_254_MOESM4_ESM.zip › Figure 2/2F/BEASD-5uMCIM7_DAPI.TIF]

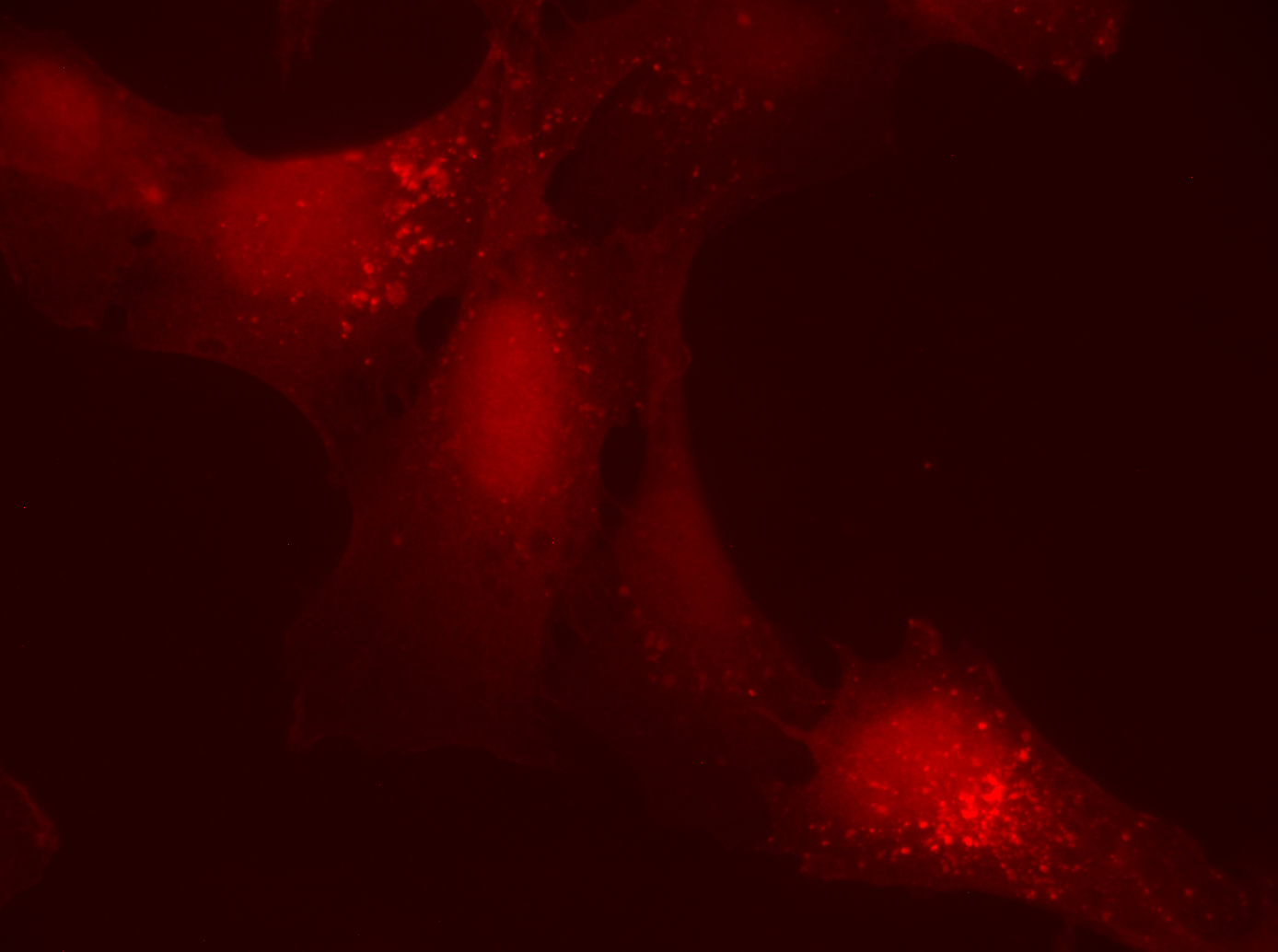

Supplement: Supplementary file 4 — Source data Fig. 2 [file 44321_2025_254_MOESM4_ESM.zip › Figure 2/2F/BEASD-5uMCIM7_Rhodamine.TIF]

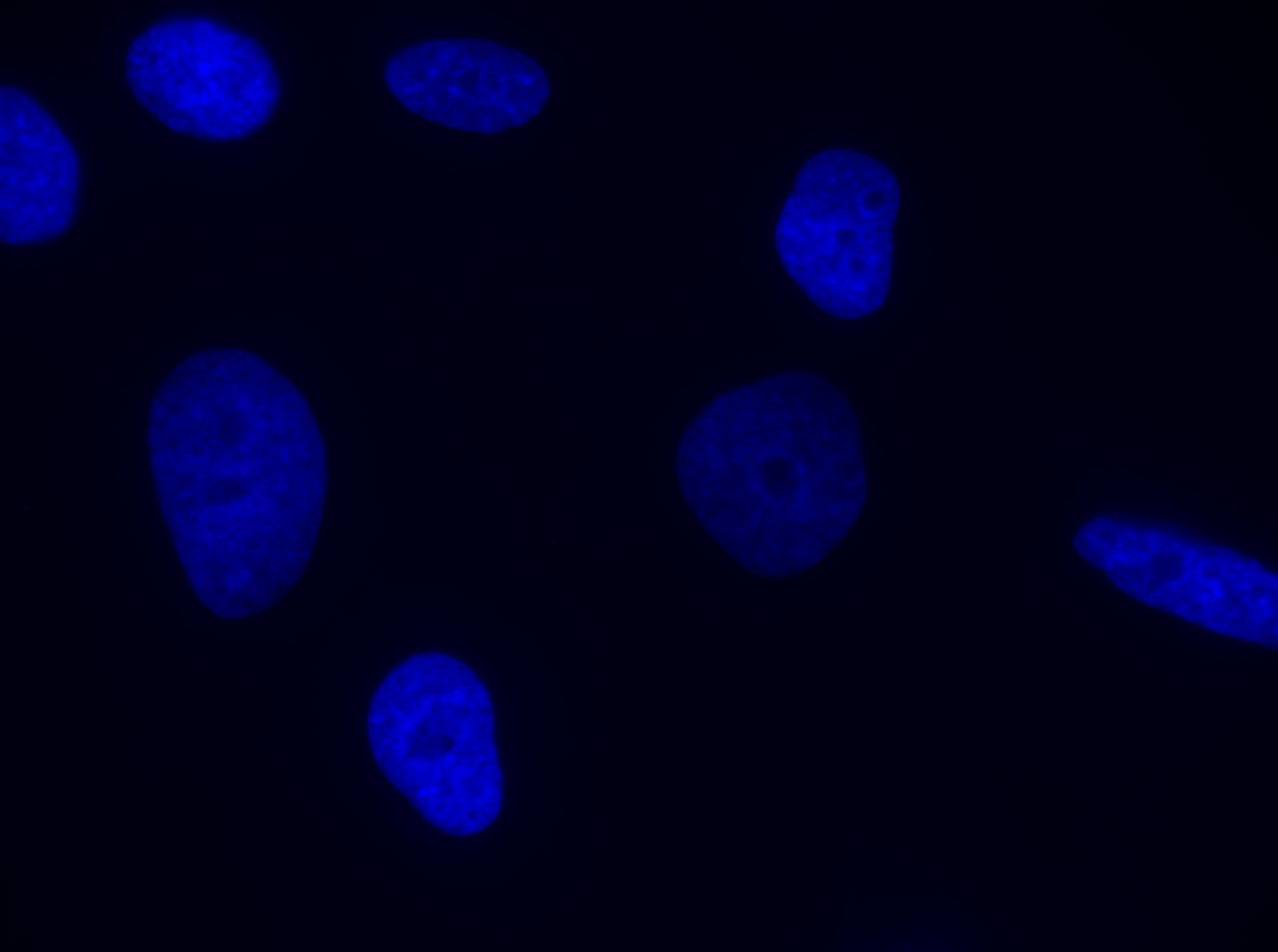

Supplement: Supplementary file 4 — Source data Fig. 2 [file 44321_2025_254_MOESM4_ESM.zip › Figure 2/2F/BEASD-DMSO_DAPI.TIF]

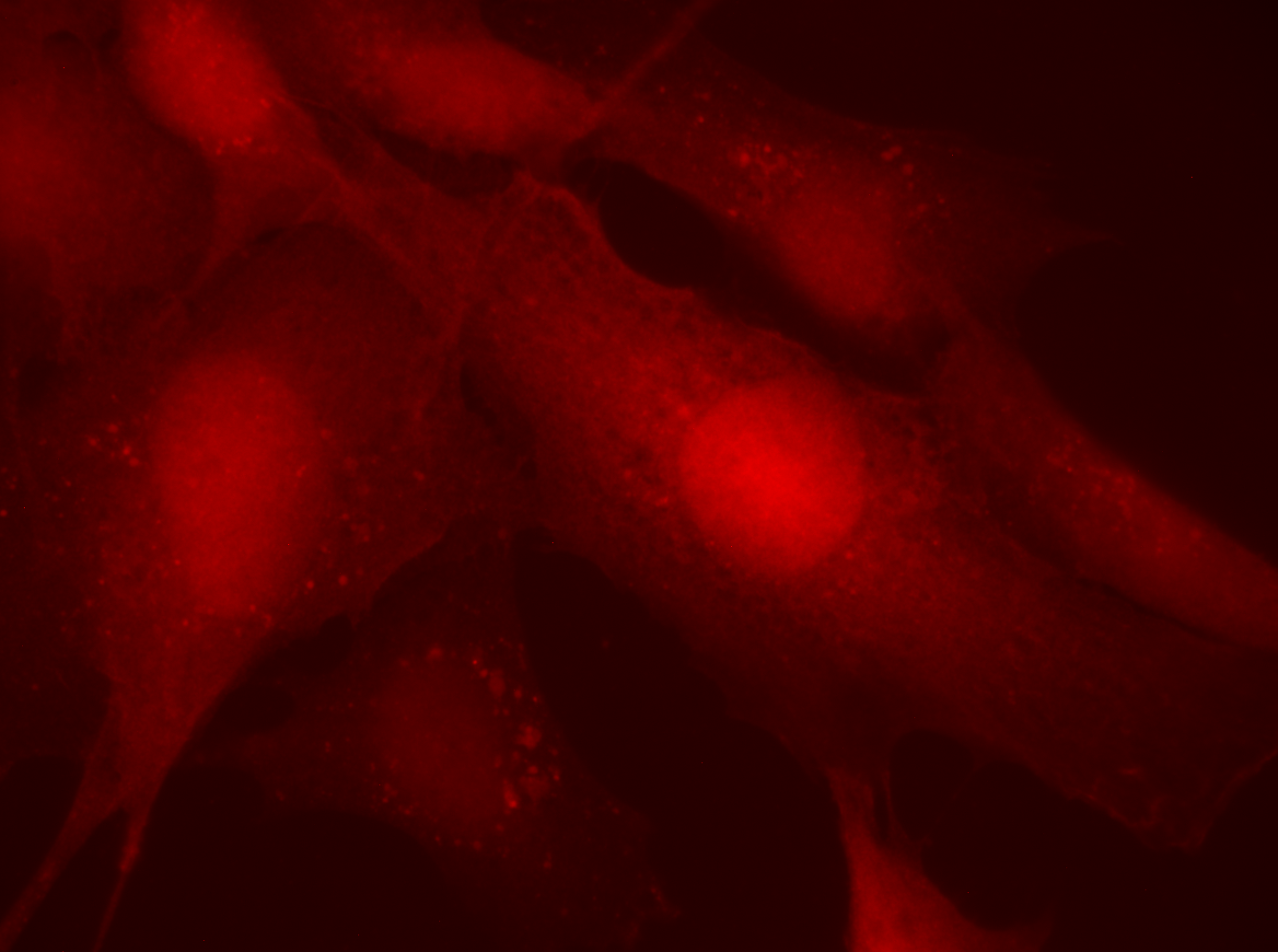

Supplement: Supplementary file 4 — Source data Fig. 2 [file 44321_2025_254_MOESM4_ESM.zip › Figure 2/2F/BEASD-DMSO_Rhodamine.TIF]

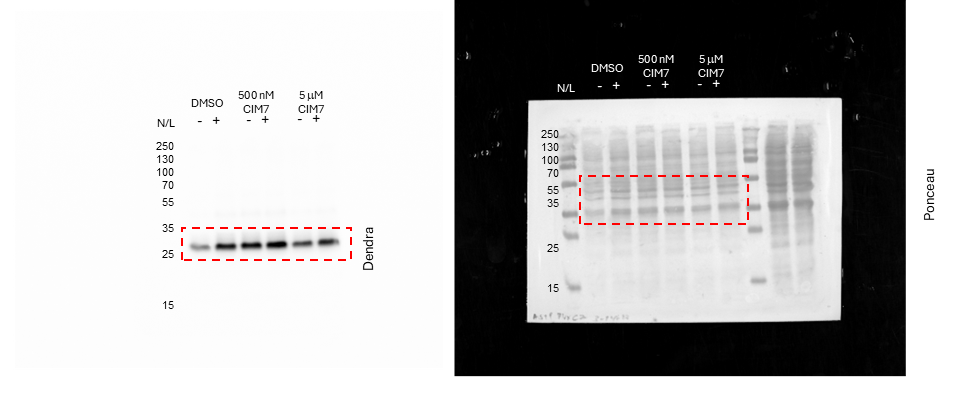

Supplement: Supplementary file 4 — Source data Fig. 2 [file 44321_2025_254_MOESM4_ESM.zip › Figure 2/2G/AnnotatedBlots.png]

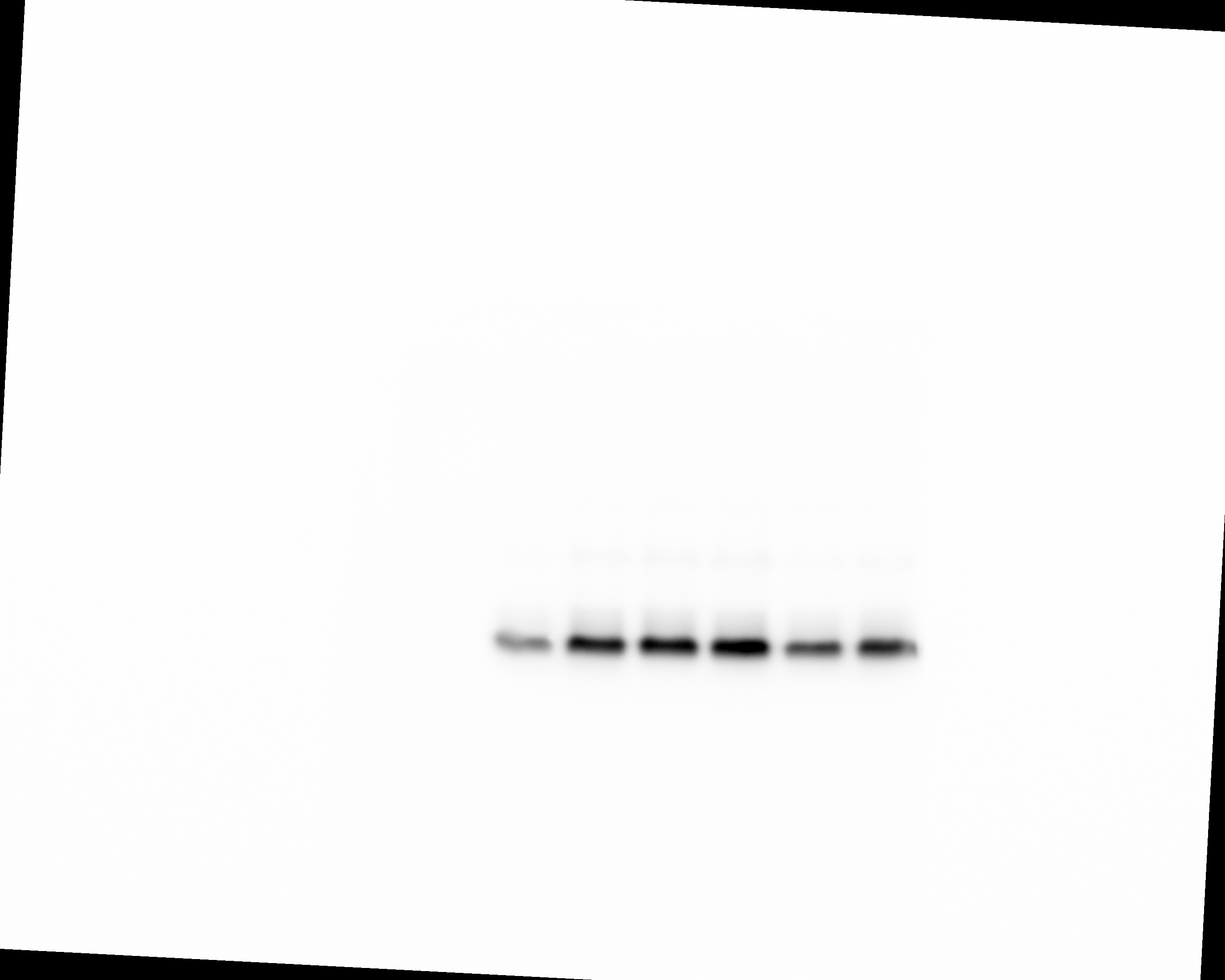

Supplement: Supplementary file 4 — Source data Fig. 2 [file 44321_2025_254_MOESM4_ESM.zip › Figure 2/2G/dendra-bands.tif]

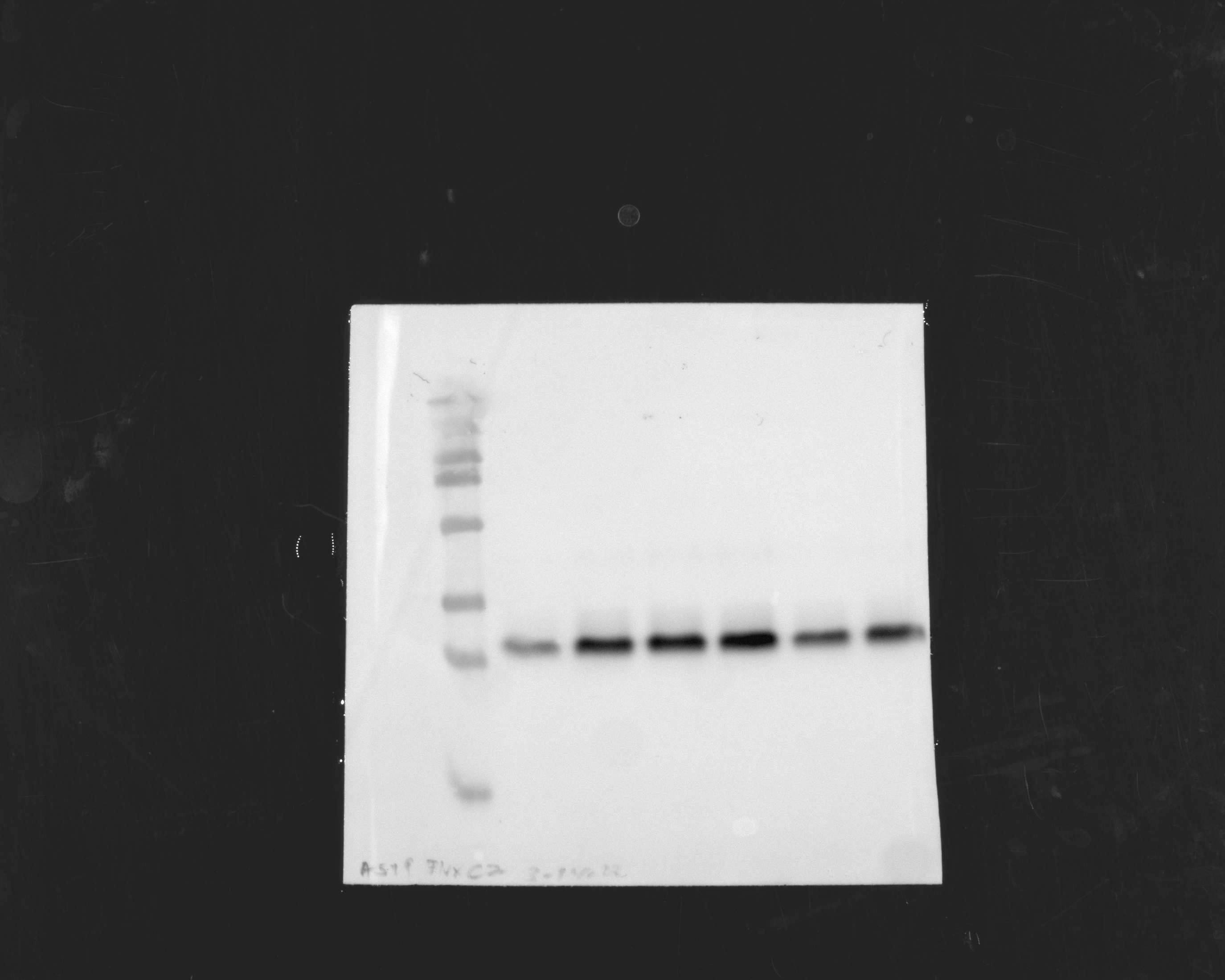

Supplement: Supplementary file 4 — Source data Fig. 2 [file 44321_2025_254_MOESM4_ESM.zip › Figure 2/2G/dendra-full.tif]

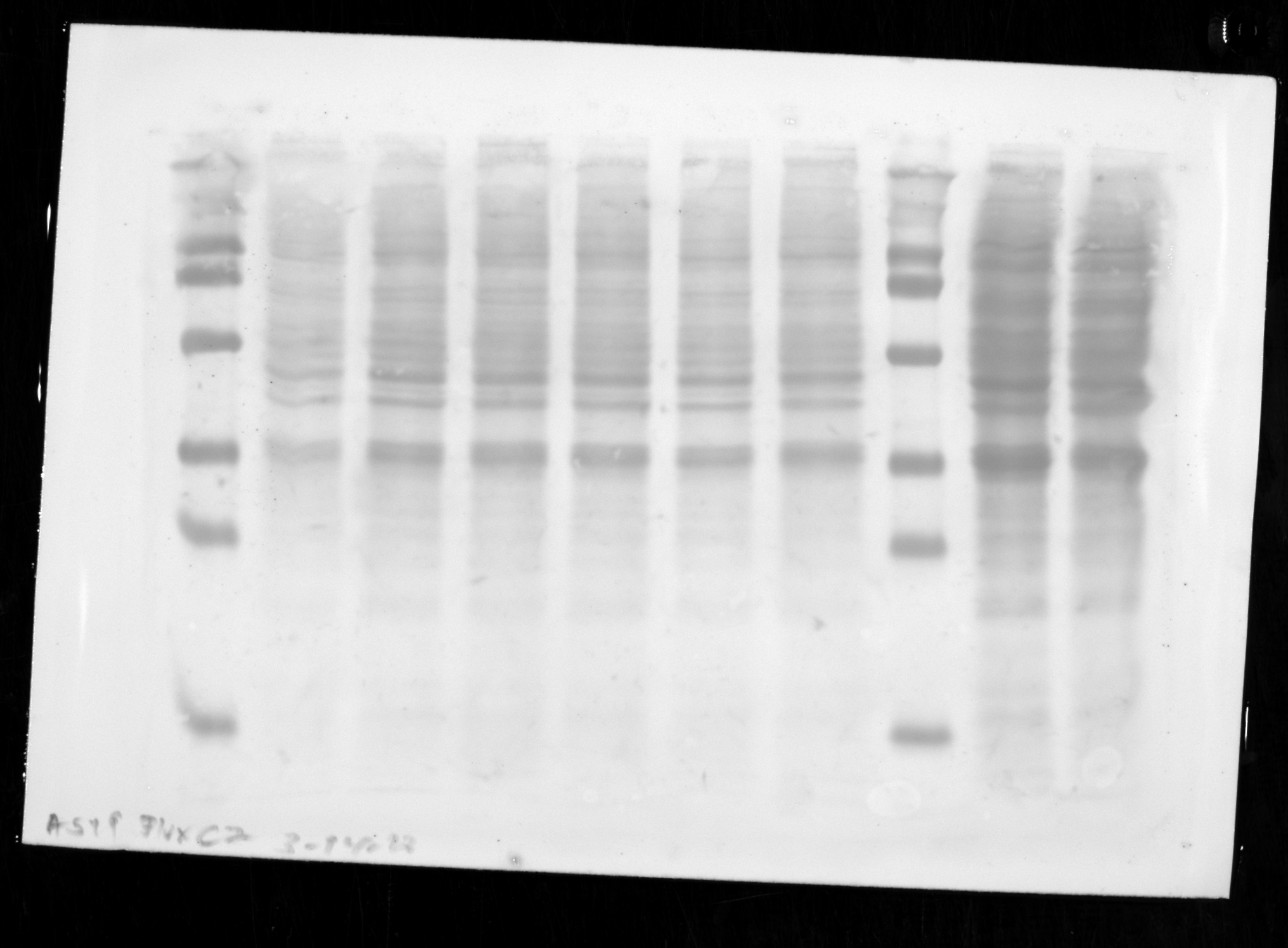

Supplement: Supplementary file 4 — Source data Fig. 2 [file 44321_2025_254_MOESM4_ESM.zip › Figure 2/2G/PONCEAU.tif]

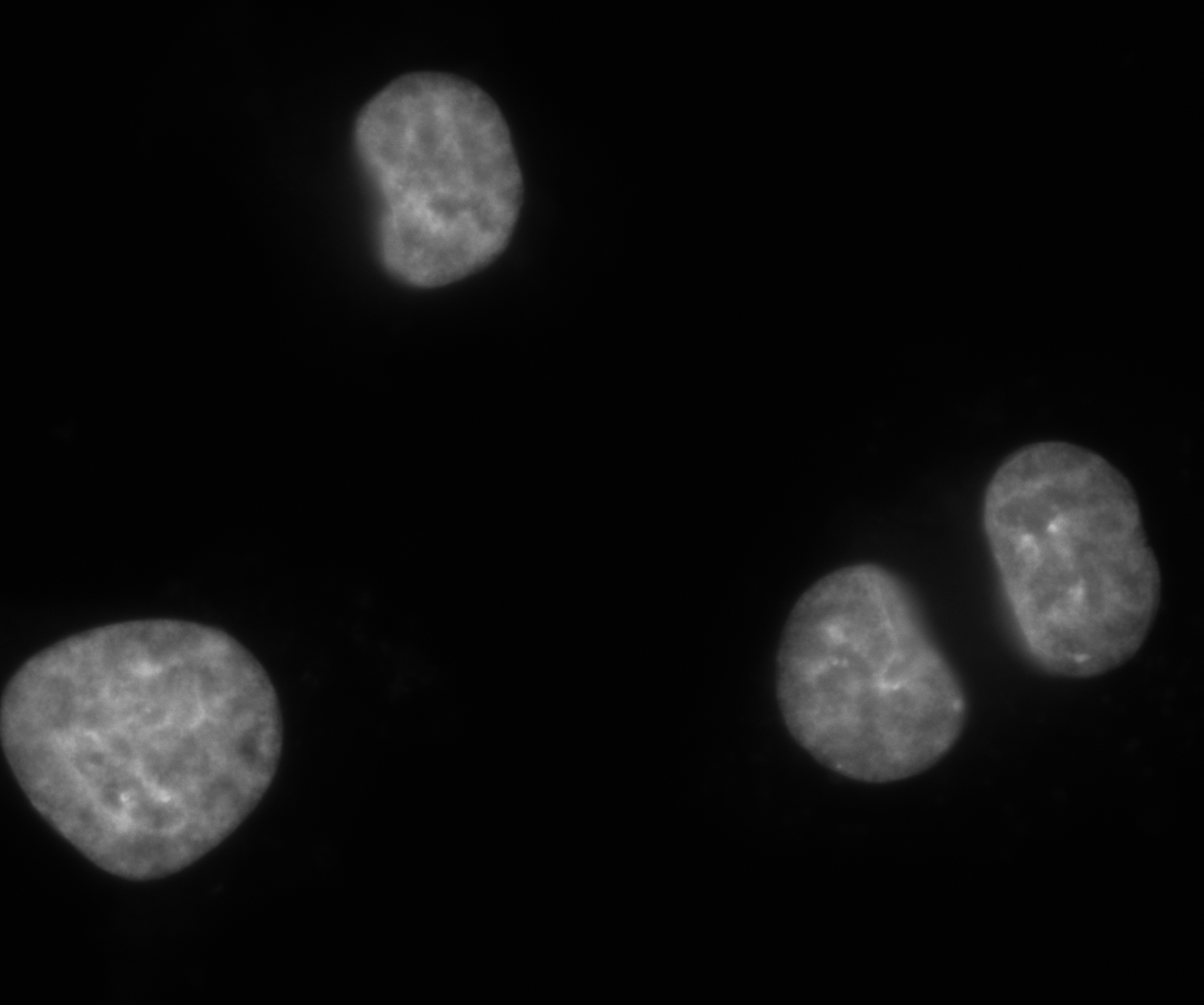

Supplement: Supplementary file 6 — Source data Fig. 4 [file 44321_2025_254_MOESM6_ESM.zip › Figure 4/4A/A549-CTsi_DAPI.tif]

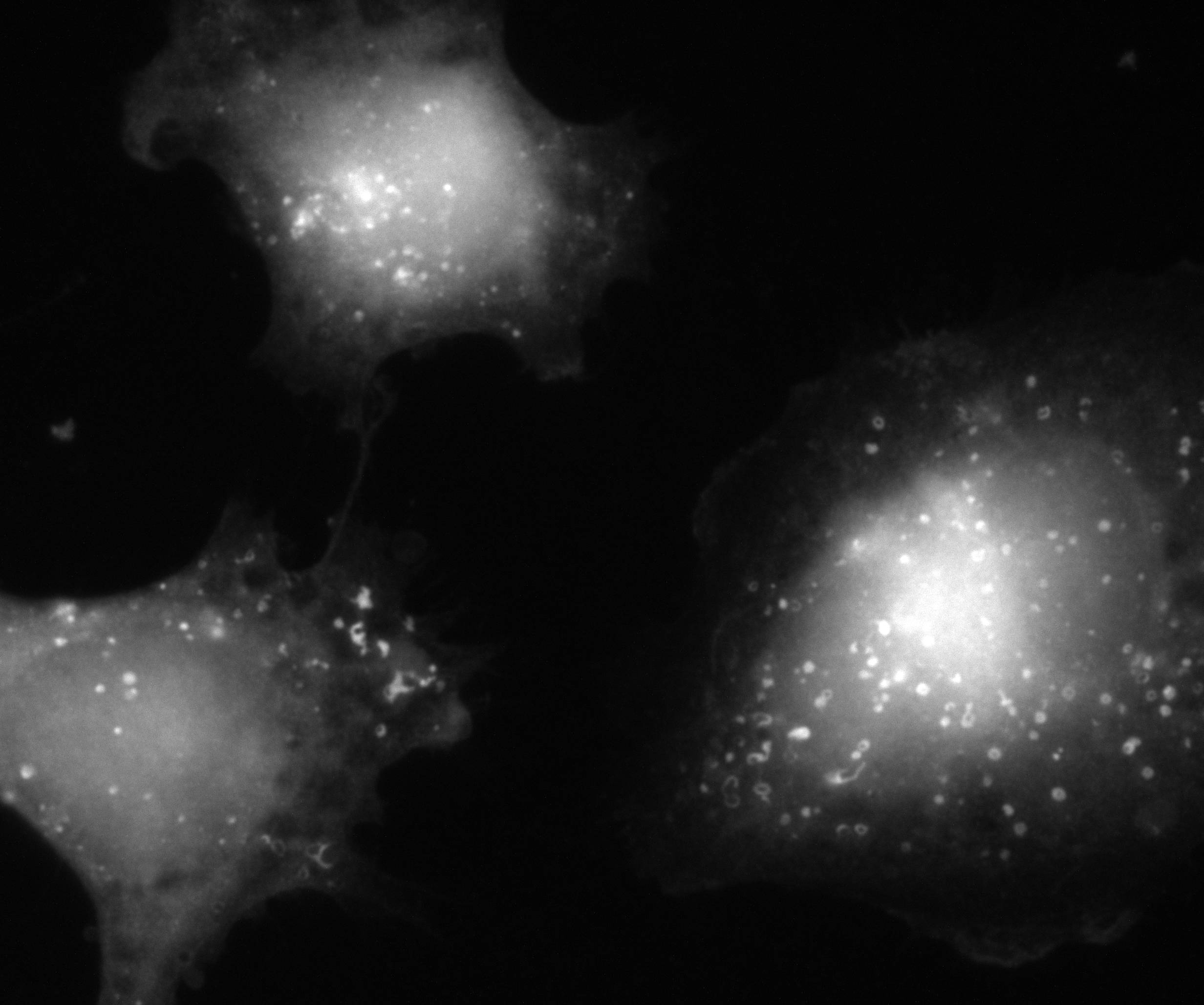

Supplement: Supplementary file 6 — Source data Fig. 4 [file 44321_2025_254_MOESM6_ESM.zip › Figure 4/4A/A549-CTsi_Rhodamine.tif]

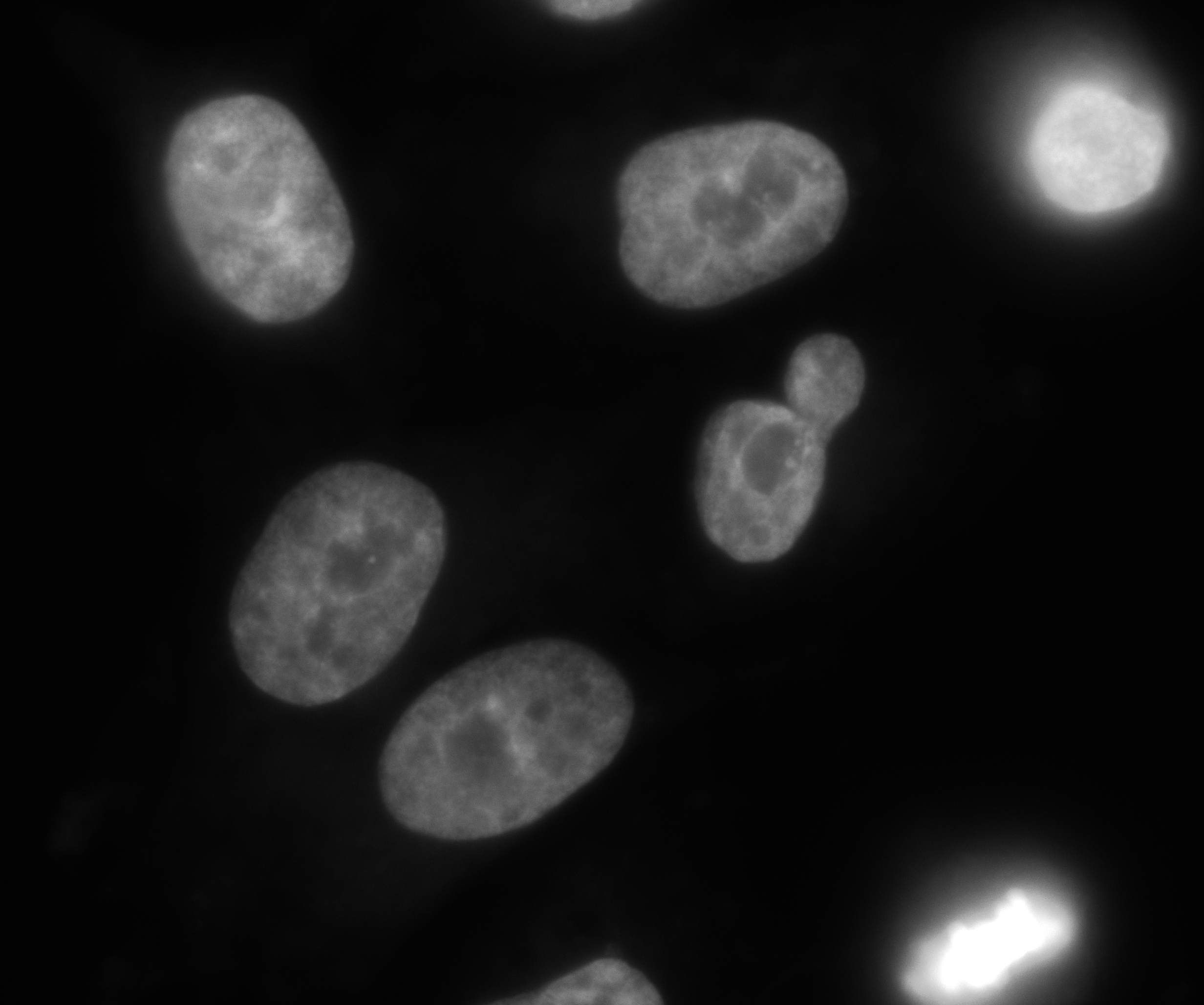

Supplement: Supplementary file 6 — Source data Fig. 4 [file 44321_2025_254_MOESM6_ESM.zip › Figure 4/4A/A549-CTsi-5CIM7_DAPI.tif]

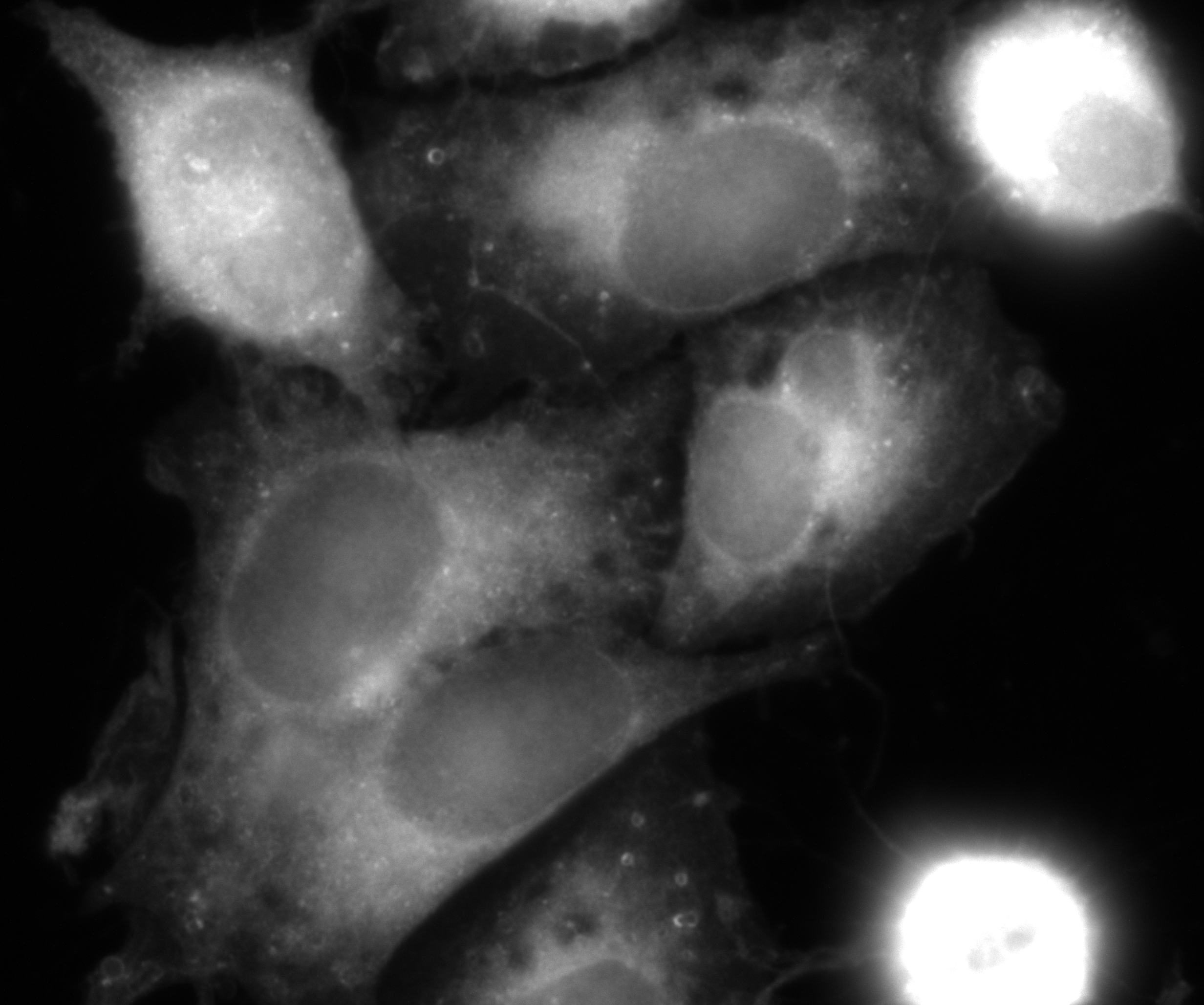

Supplement: Supplementary file 6 — Source data Fig. 4 [file 44321_2025_254_MOESM6_ESM.zip › Figure 4/4A/A549-CTsi-5CIM7_Rhodamine.tif]

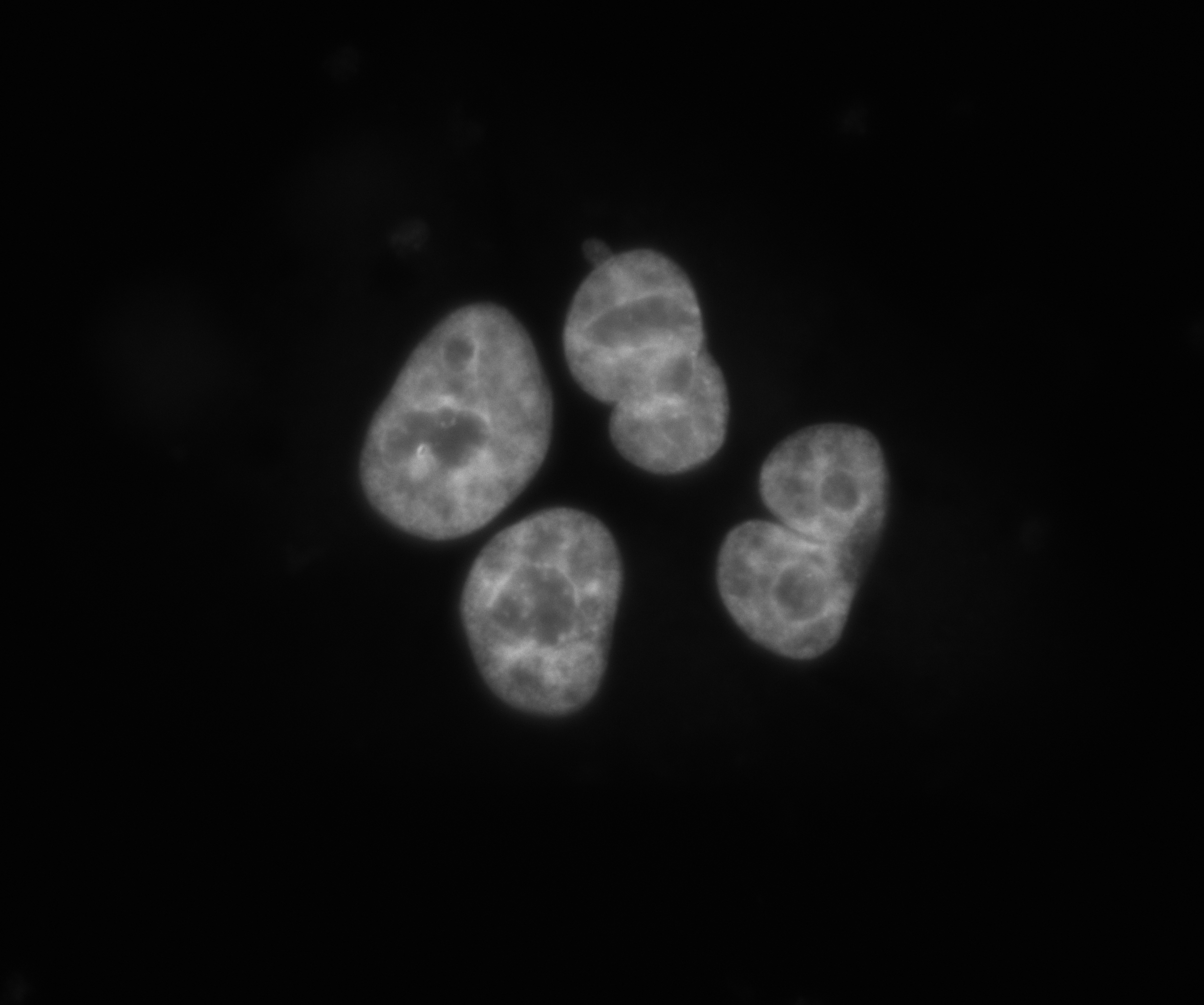

Supplement: Supplementary file 6 — Source data Fig. 4 [file 44321_2025_254_MOESM6_ESM.zip › Figure 4/4A/A549-NCoRsi_DAPI.tif]

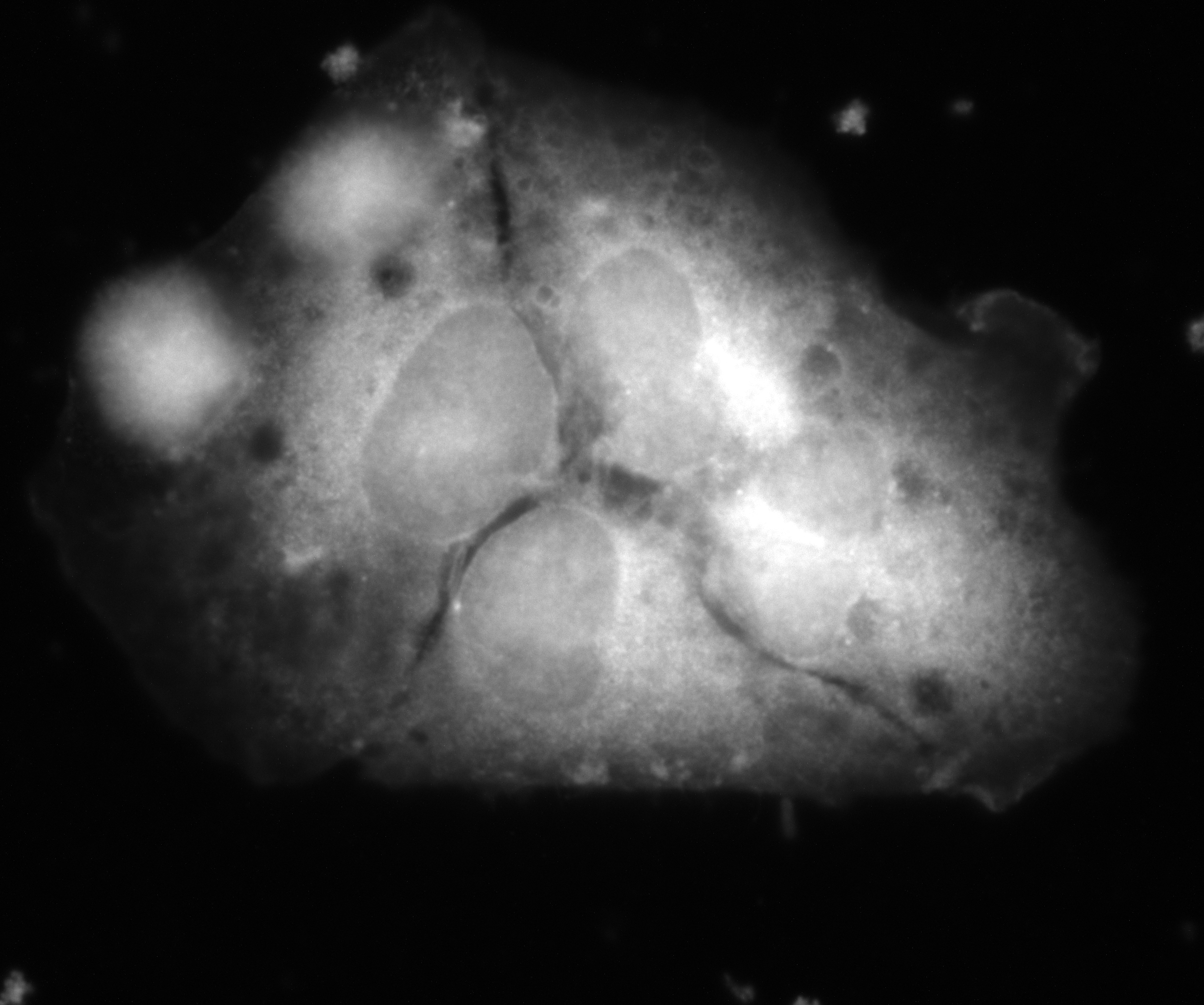

Supplement: Supplementary file 6 — Source data Fig. 4 [file 44321_2025_254_MOESM6_ESM.zip › Figure 4/4A/A549-NCoRsi_Rhodamine.tif]

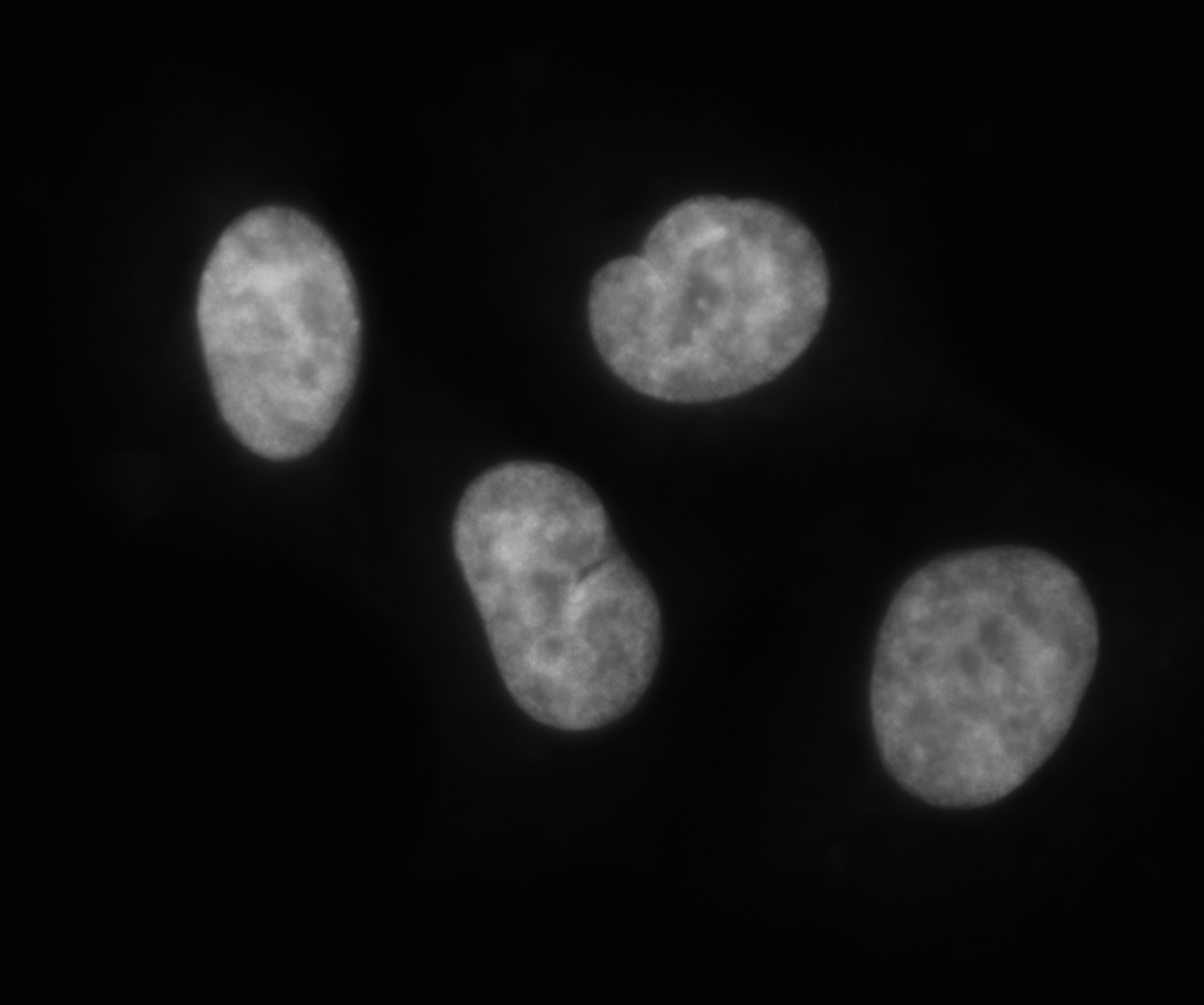

Supplement: Supplementary file 6 — Source data Fig. 4 [file 44321_2025_254_MOESM6_ESM.zip › Figure 4/4A/A549-NCoRsi-5uMCIM7_DAPI.tif]

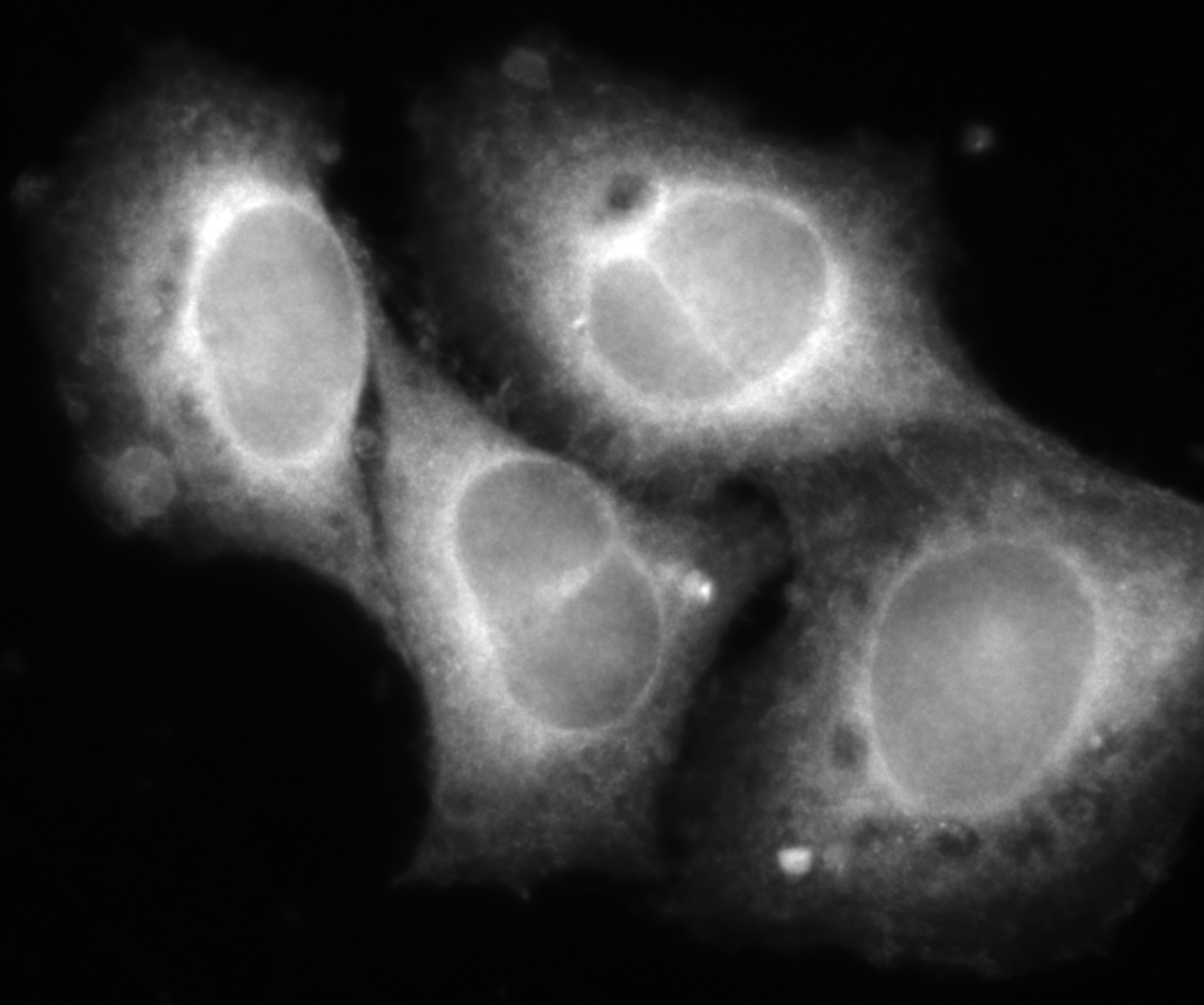

Supplement: Supplementary file 6 — Source data Fig. 4 [file 44321_2025_254_MOESM6_ESM.zip › Figure 4/4A/A549-NCoRsi-5uMCIM7_Rhodamine.tif]

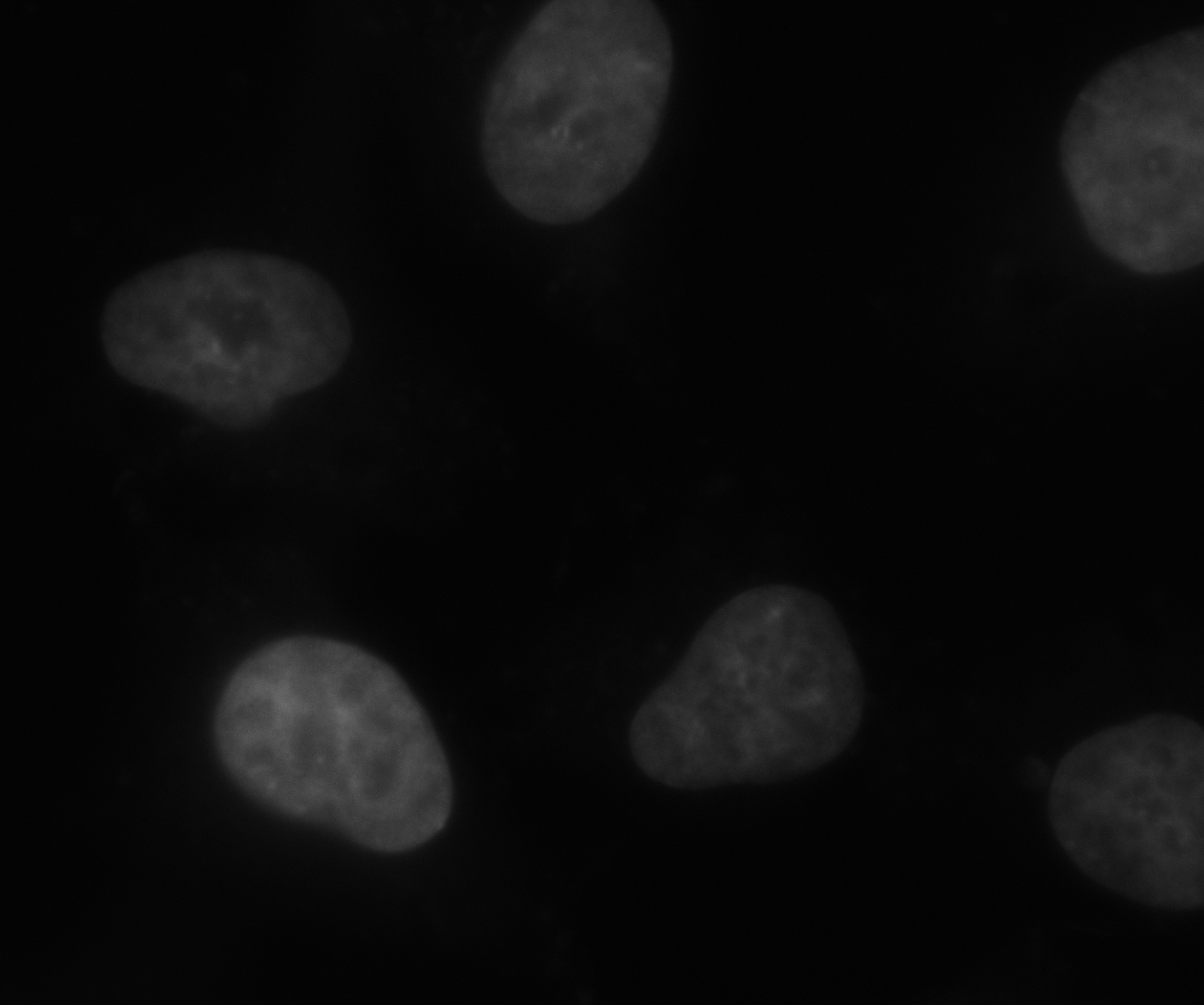

Supplement: Supplementary file 6 — Source data Fig. 4 [file 44321_2025_254_MOESM6_ESM.zip › Figure 4/4A/A549-RARsi_DAPI.tif]

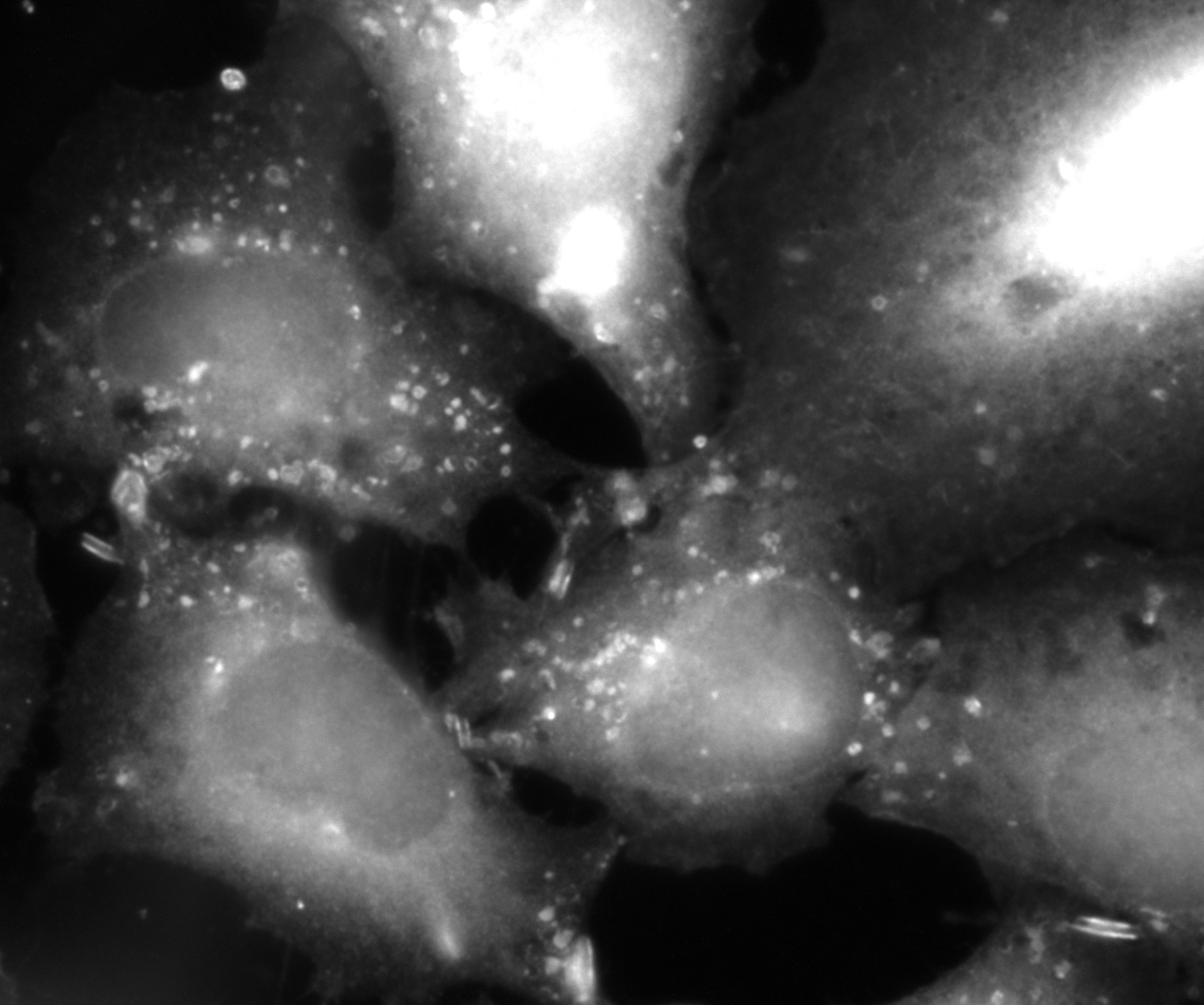

Supplement: Supplementary file 6 — Source data Fig. 4 [file 44321_2025_254_MOESM6_ESM.zip › Figure 4/4A/A549-RARsi_Rhodamine.tif]

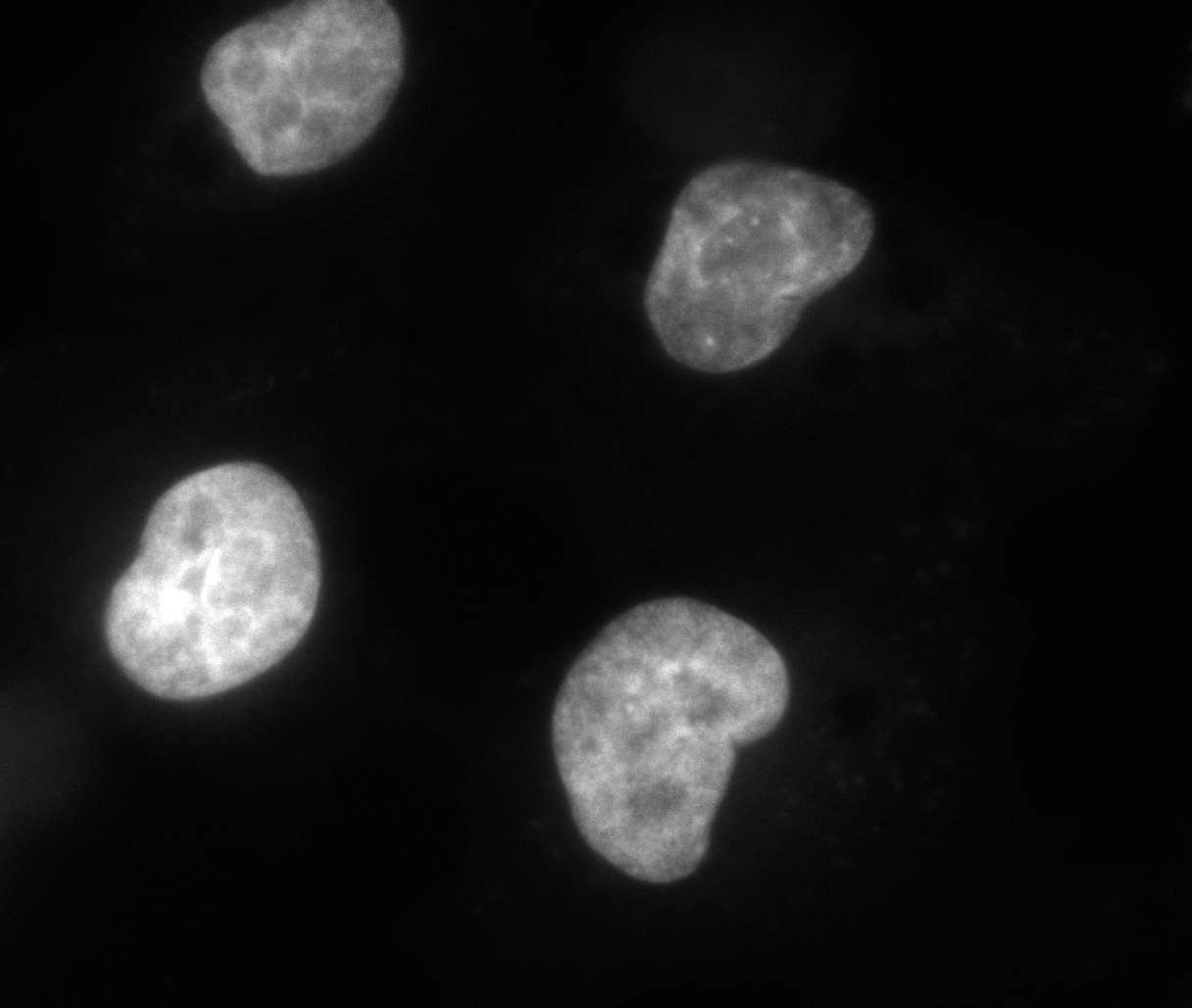

Supplement: Supplementary file 6 — Source data Fig. 4 [file 44321_2025_254_MOESM6_ESM.zip › Figure 4/4A/A549-RARsi-5uMCIM7_DMSO.tif]

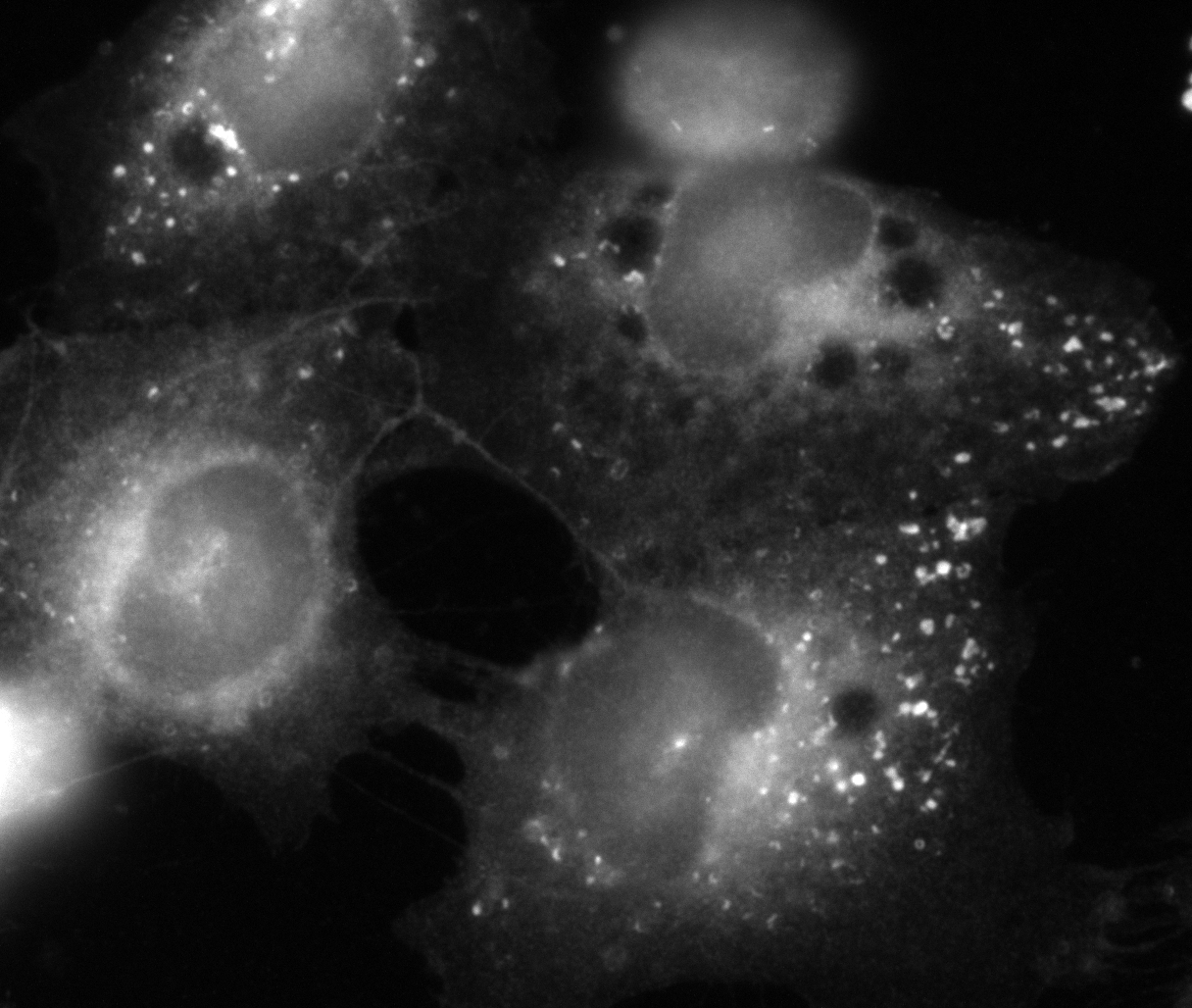

Supplement: Supplementary file 6 — Source data Fig. 4 [file 44321_2025_254_MOESM6_ESM.zip › Figure 4/4A/A549-RARsi-5uMCIM7_Rhodamine.tif]

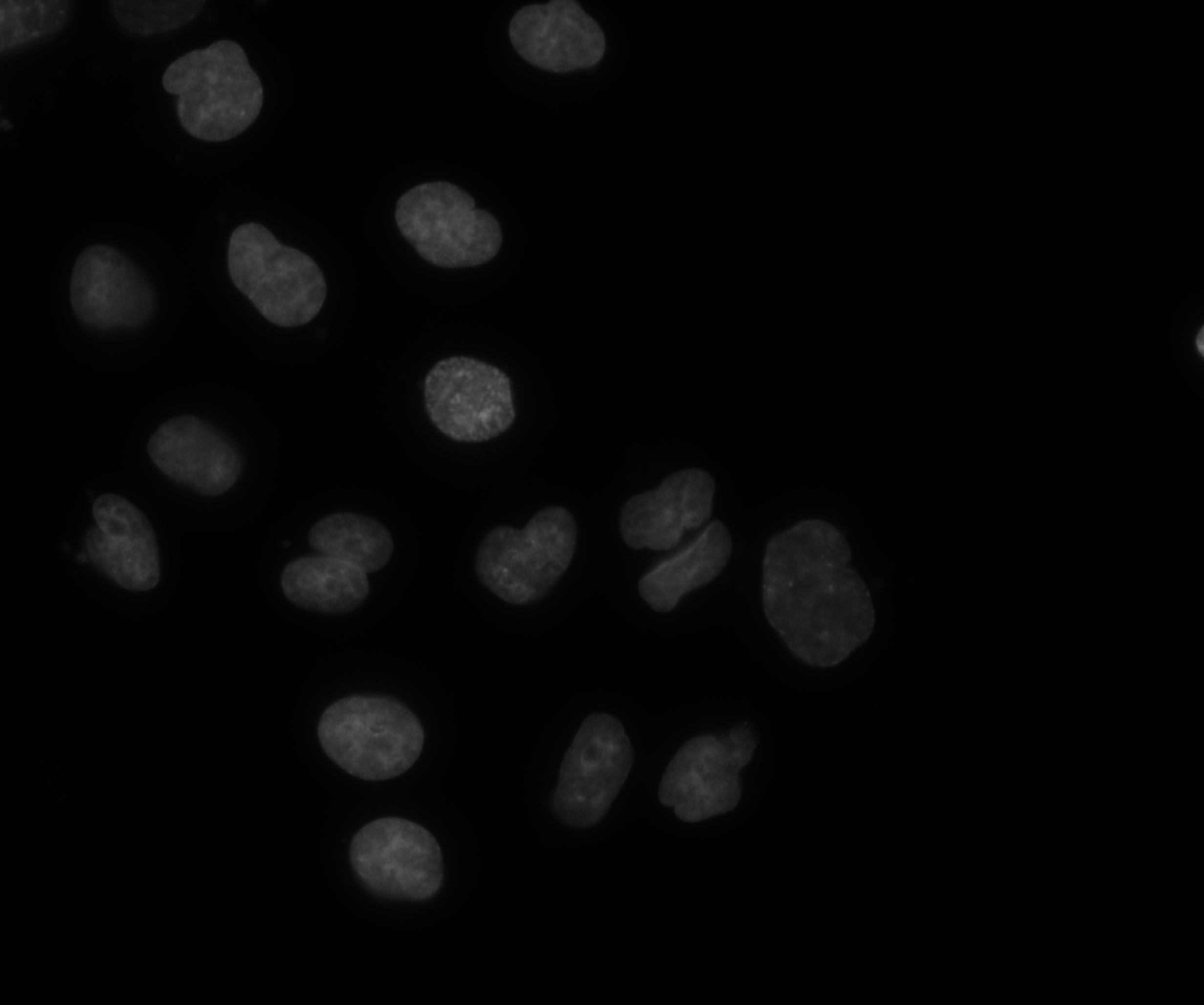

Supplement: Supplementary file 6 — Source data Fig. 4 [file 44321_2025_254_MOESM6_ESM.zip › Figure 4/4B/5uMCIM7_DAPI.tif]

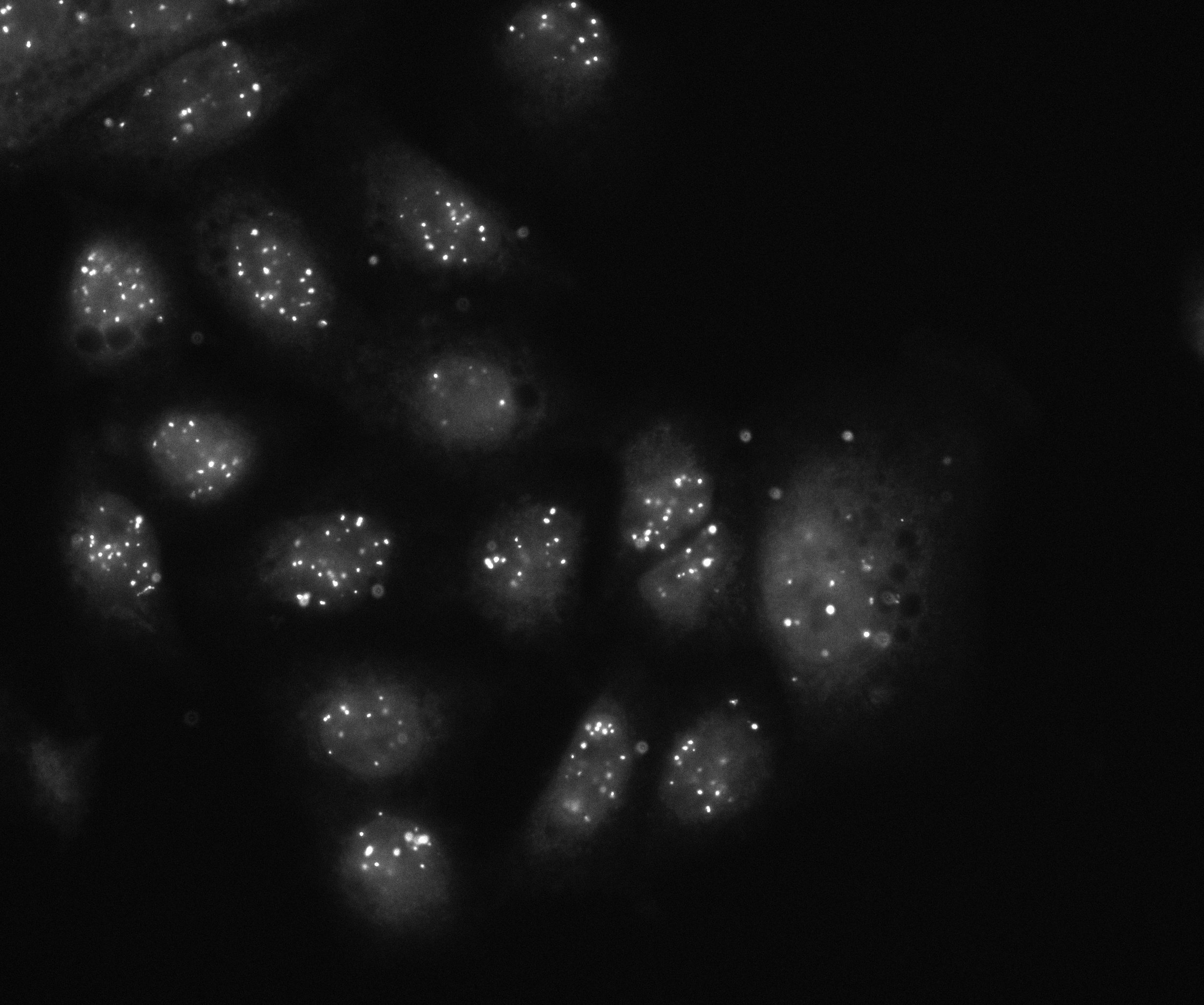

Supplement: Supplementary file 6 — Source data Fig. 4 [file 44321_2025_254_MOESM6_ESM.zip › Figure 4/4B/5uMCIM7_Rhodamine_NCoRRAR.tif]

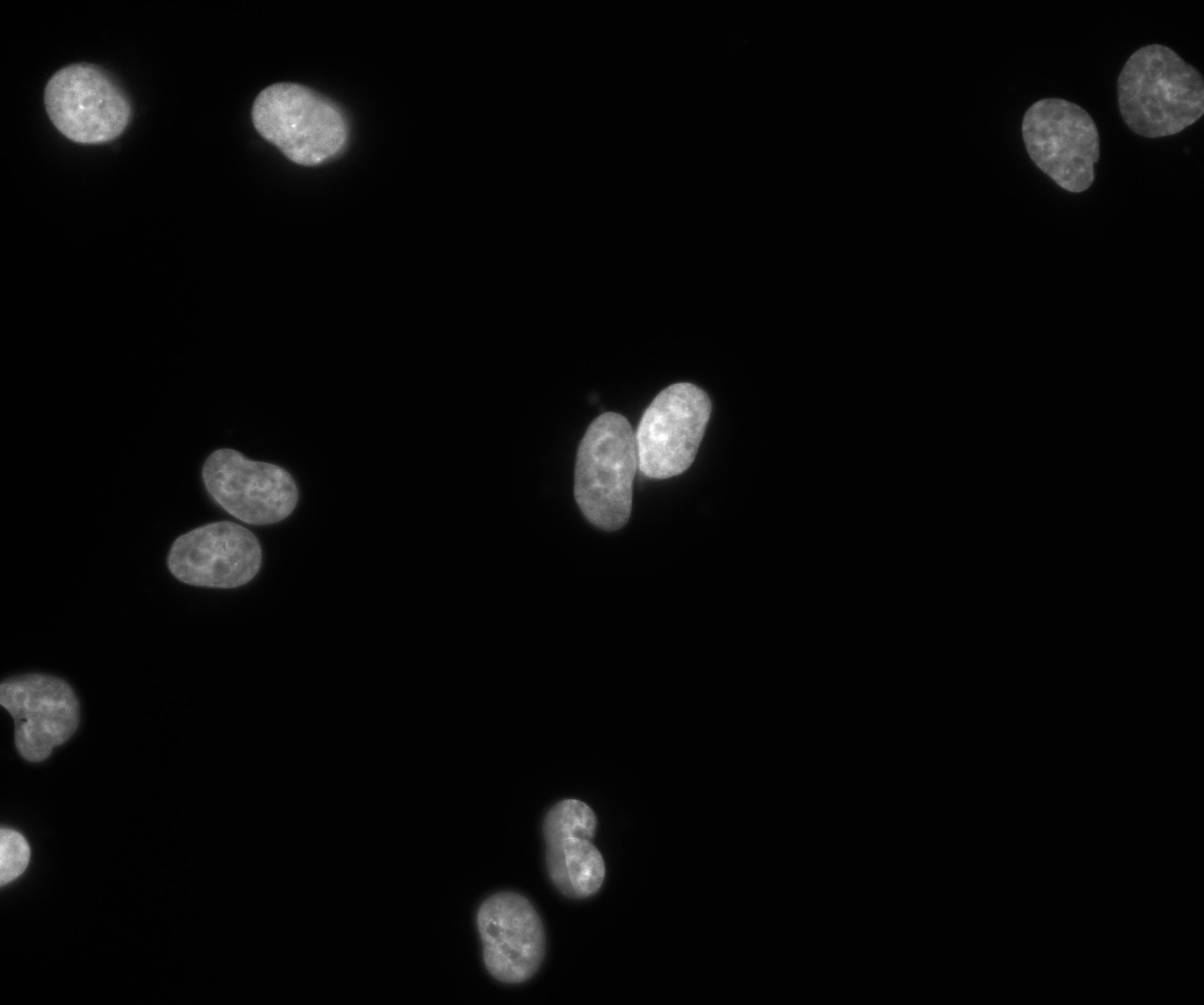

Supplement: Supplementary file 6 — Source data Fig. 4 [file 44321_2025_254_MOESM6_ESM.zip › Figure 4/4B/DMSO_DAPI.tif]

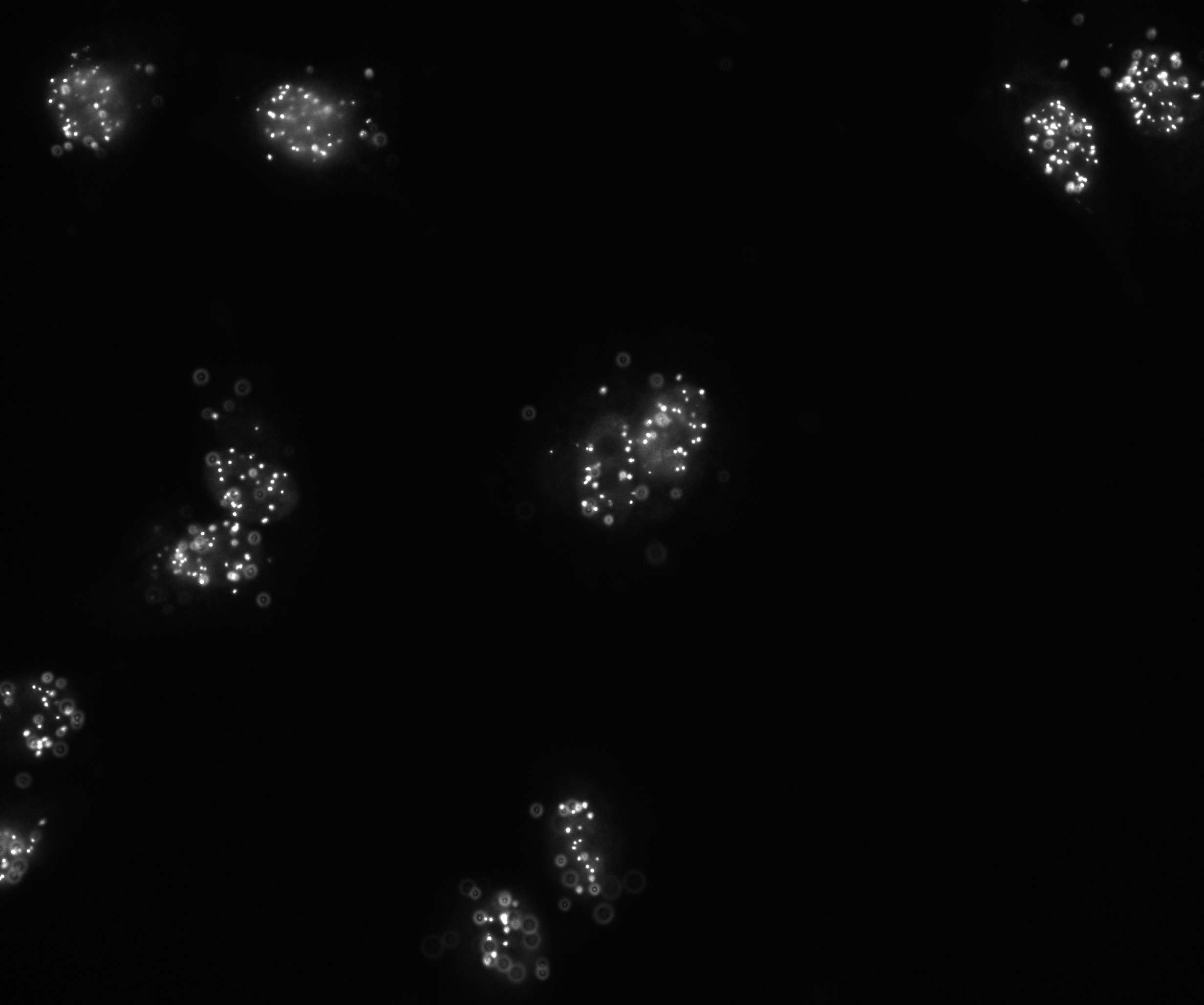

Supplement: Supplementary file 6 — Source data Fig. 4 [file 44321_2025_254_MOESM6_ESM.zip › Figure 4/4B/DMSO_Rhodamine_NCoRRAR.tif]

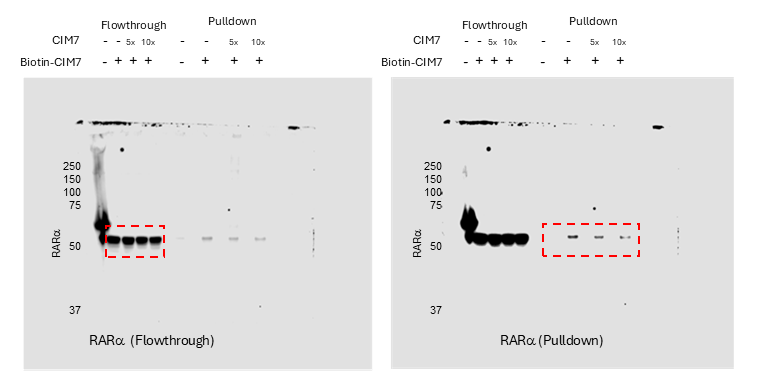

Supplement: Supplementary file 6 — Source data Fig. 4 [file 44321_2025_254_MOESM6_ESM.zip › Figure 4/4C/Annoted-Blots.png]
