# Supplementary figures and images for: Small molecule disruption of RARα/NCoR1 interaction inhibits chaperone-mediated autophagy in cancer (part 2 of 2)
Source: EMBO Mol Med. 2025 Jun 9;17(7):1716–55. doi: 10.1038/s44321-025-00254-y (PMC12254369; doi:10.1038/s44321-025-00254-y)

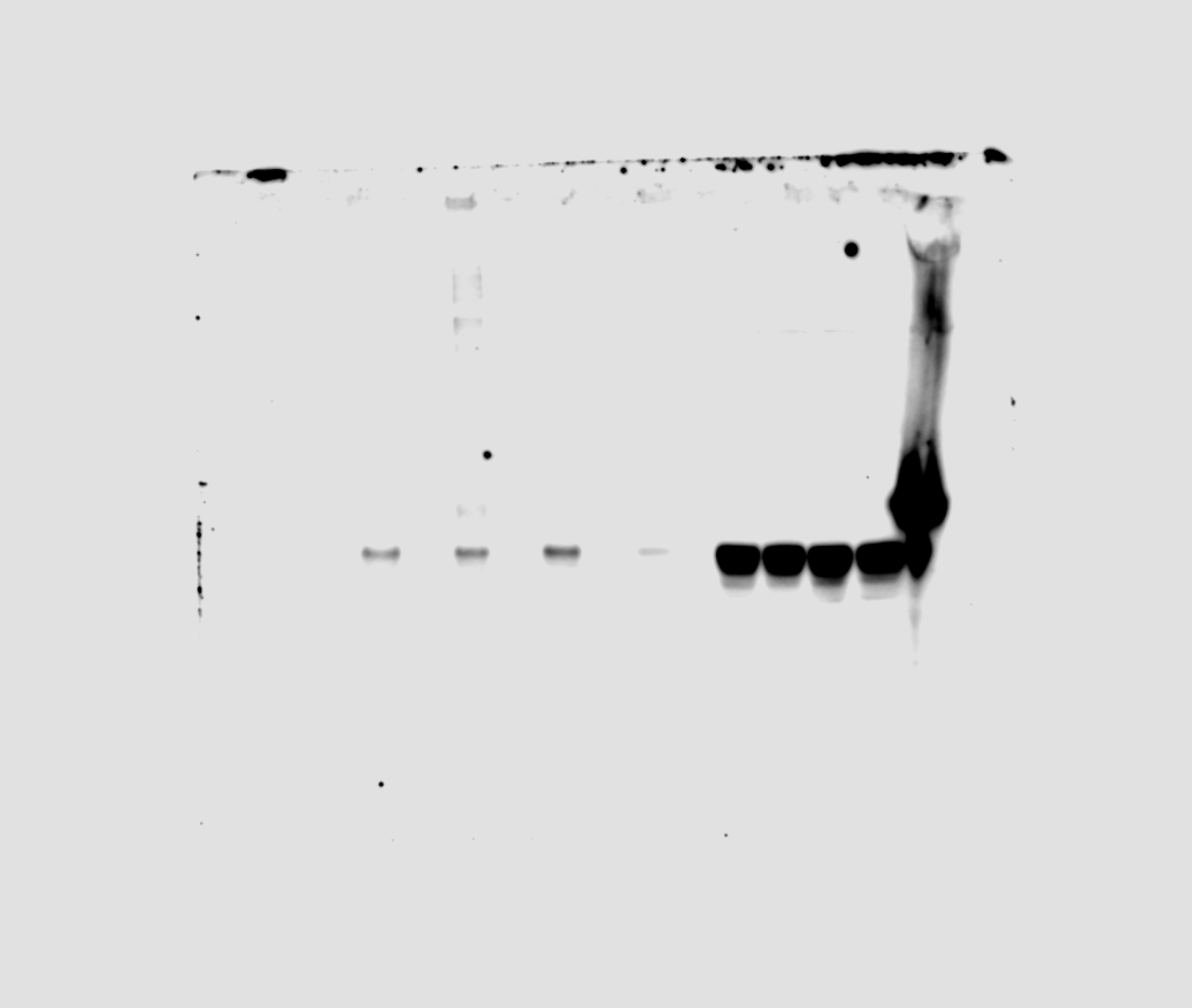

Supplement: Supplementary file 6 — Source data Fig. 4 [file 44321_2025_254_MOESM6_ESM.zip › Figure 4/4C/RARa.tif.tif]

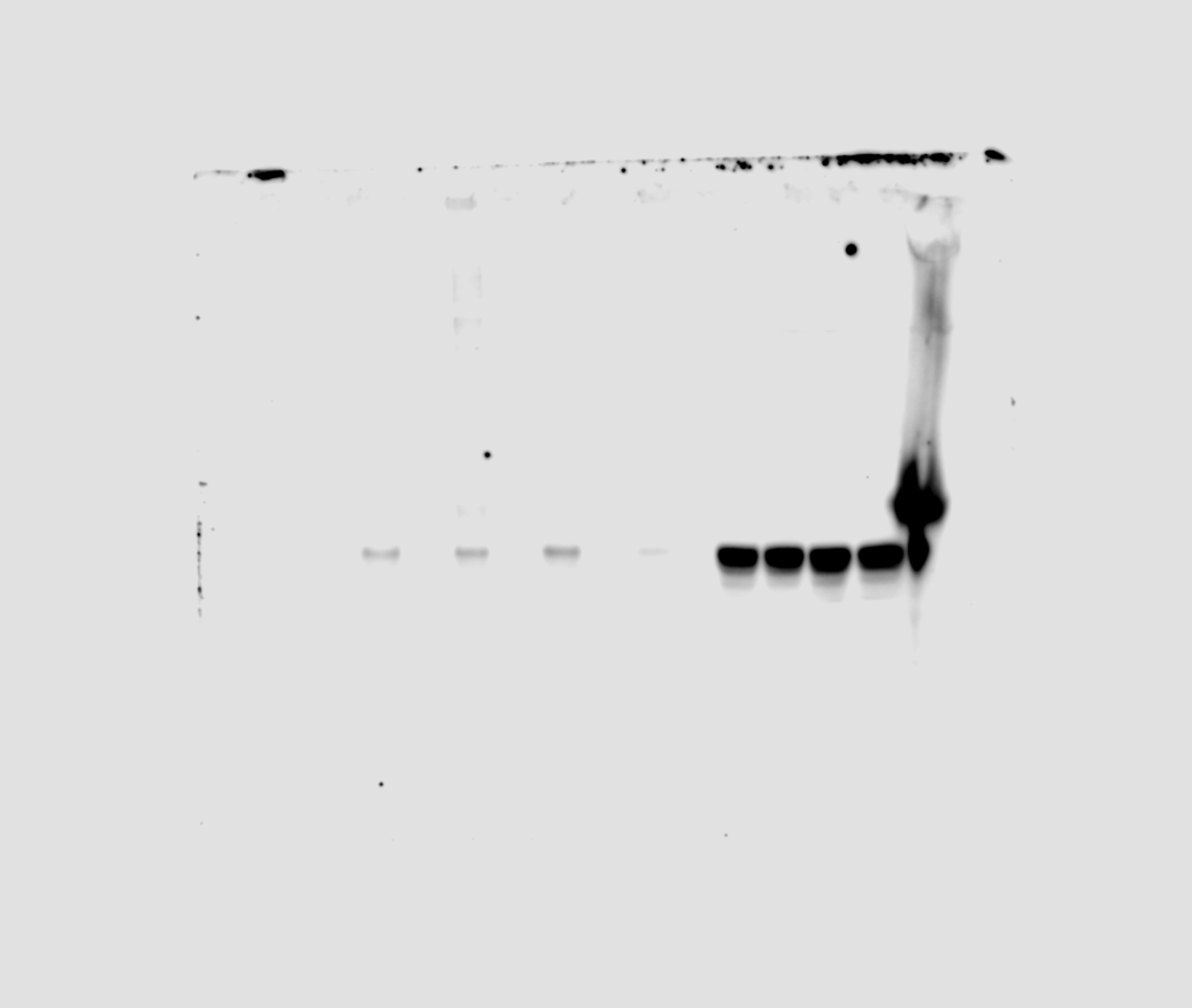

Supplement: Supplementary file 6 — Source data Fig. 4 [file 44321_2025_254_MOESM6_ESM.zip › Figure 4/4C/RARa-Flowthrough.tif]

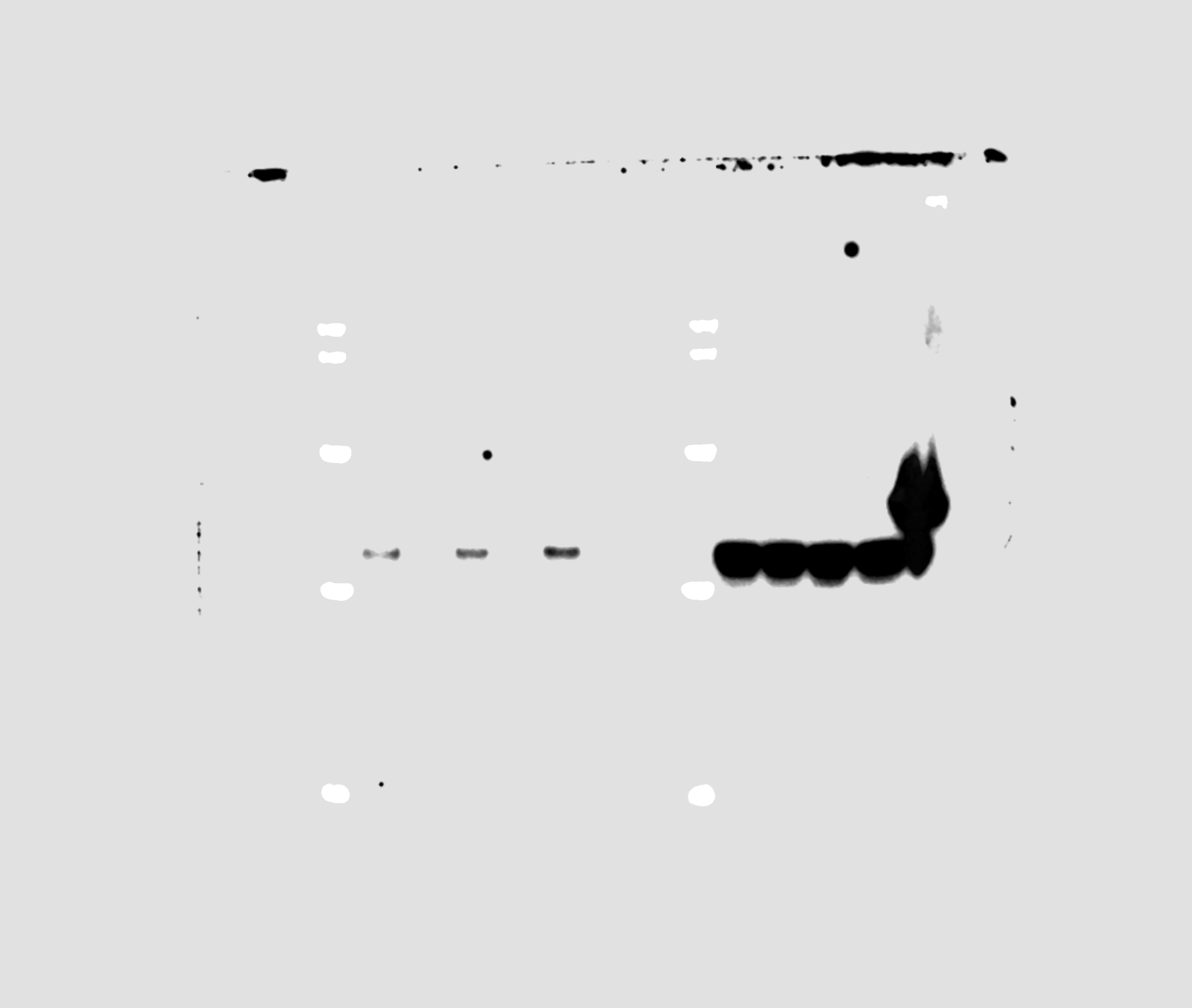

Supplement: Supplementary file 6 — Source data Fig. 4 [file 44321_2025_254_MOESM6_ESM.zip › Figure 4/4C/RARa-ladder.tif]

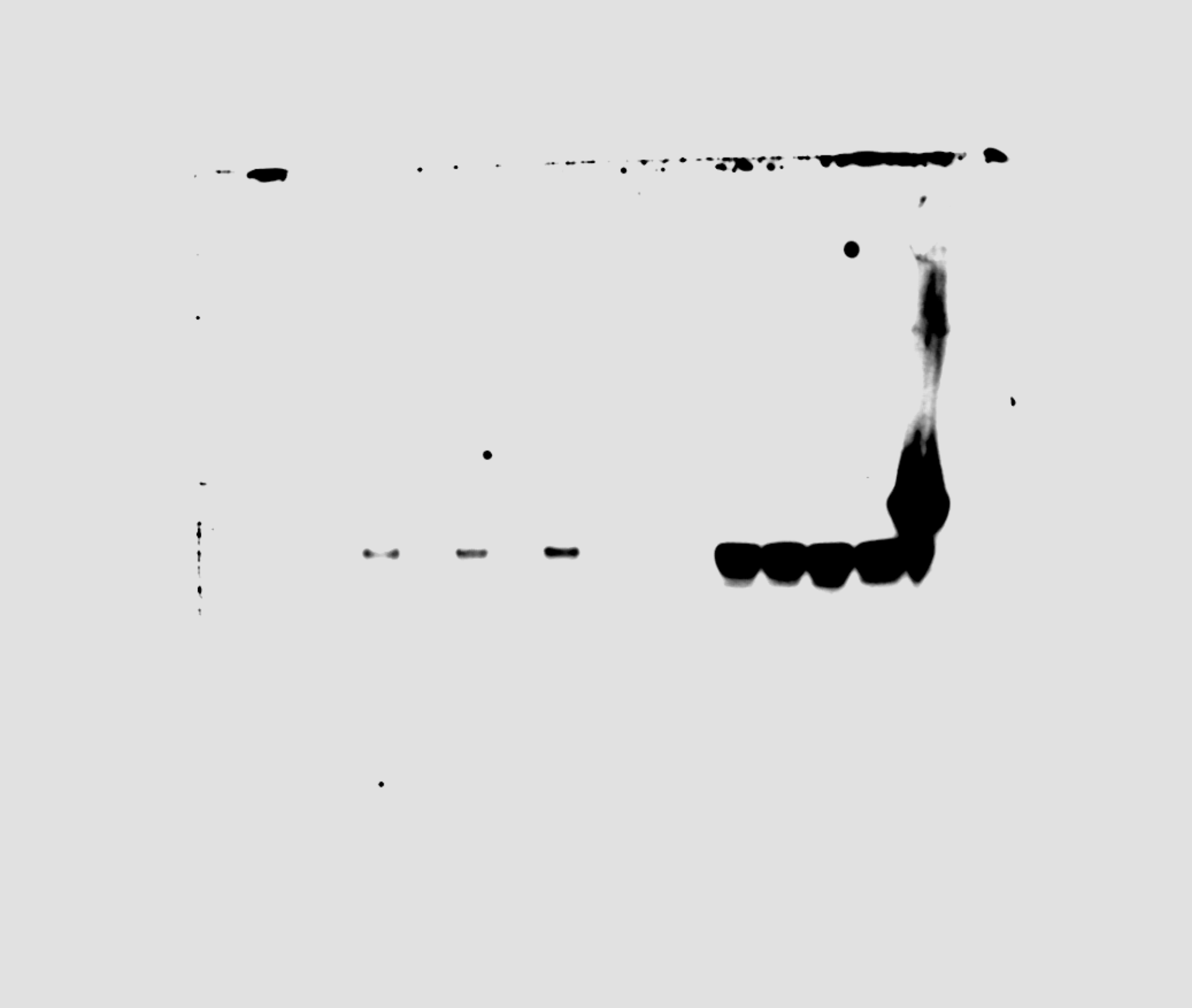

Supplement: Supplementary file 6 — Source data Fig. 4 [file 44321_2025_254_MOESM6_ESM.zip › Figure 4/4C/RARa-Pulldown.tif]

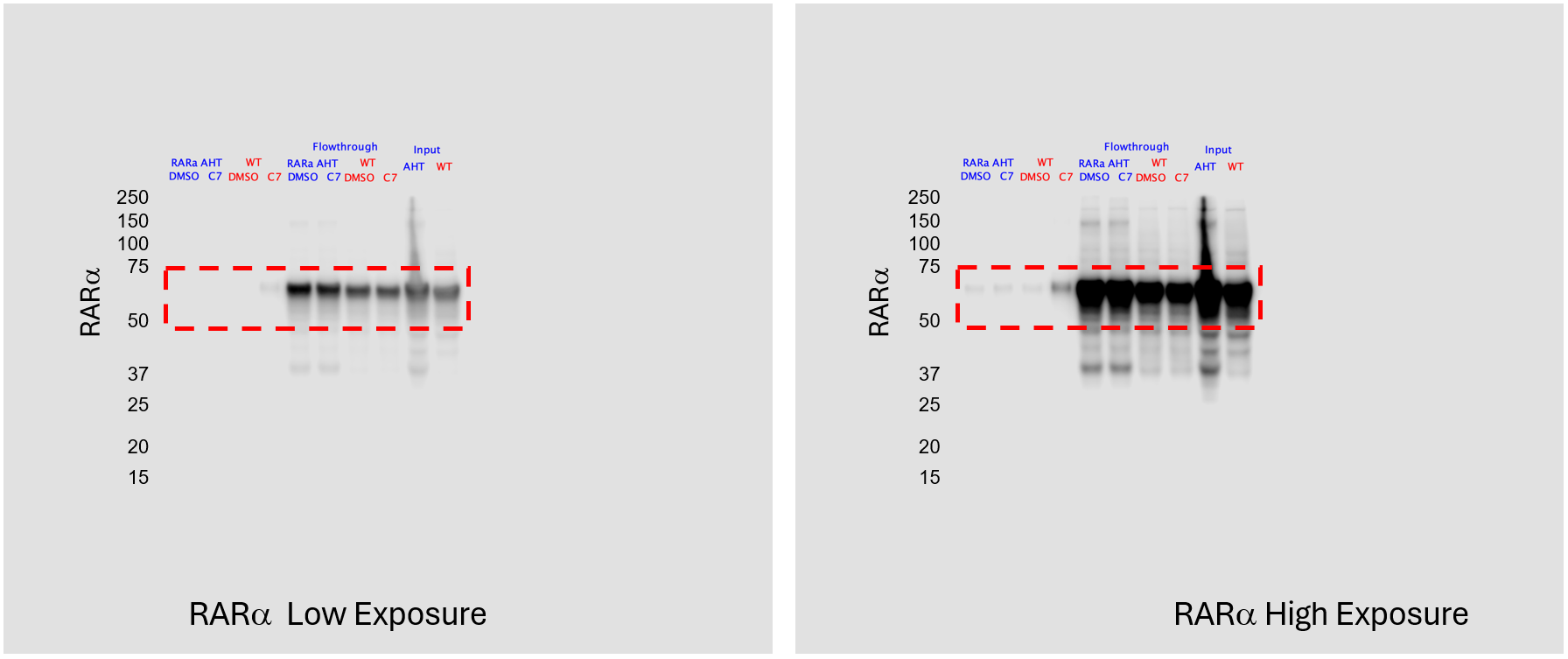

Supplement: Supplementary file 6 — Source data Fig. 4 [file 44321_2025_254_MOESM6_ESM.zip › Figure 4/4D/AnnotatedBlots.png]

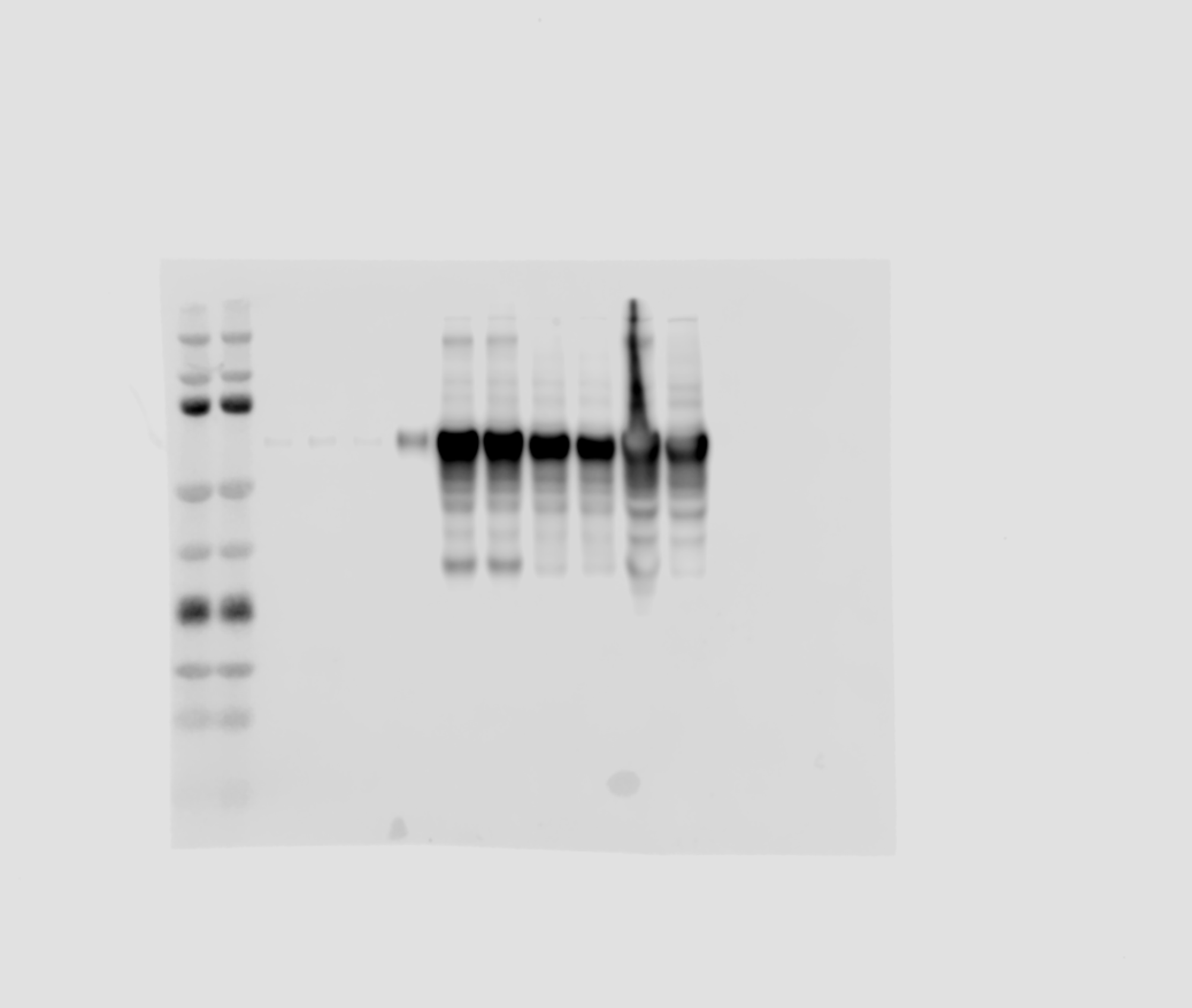

Supplement: Supplementary file 6 — Source data Fig. 4 [file 44321_2025_254_MOESM6_ESM.zip › Figure 4/4D/RARa overexpression_marker.tif.tif]

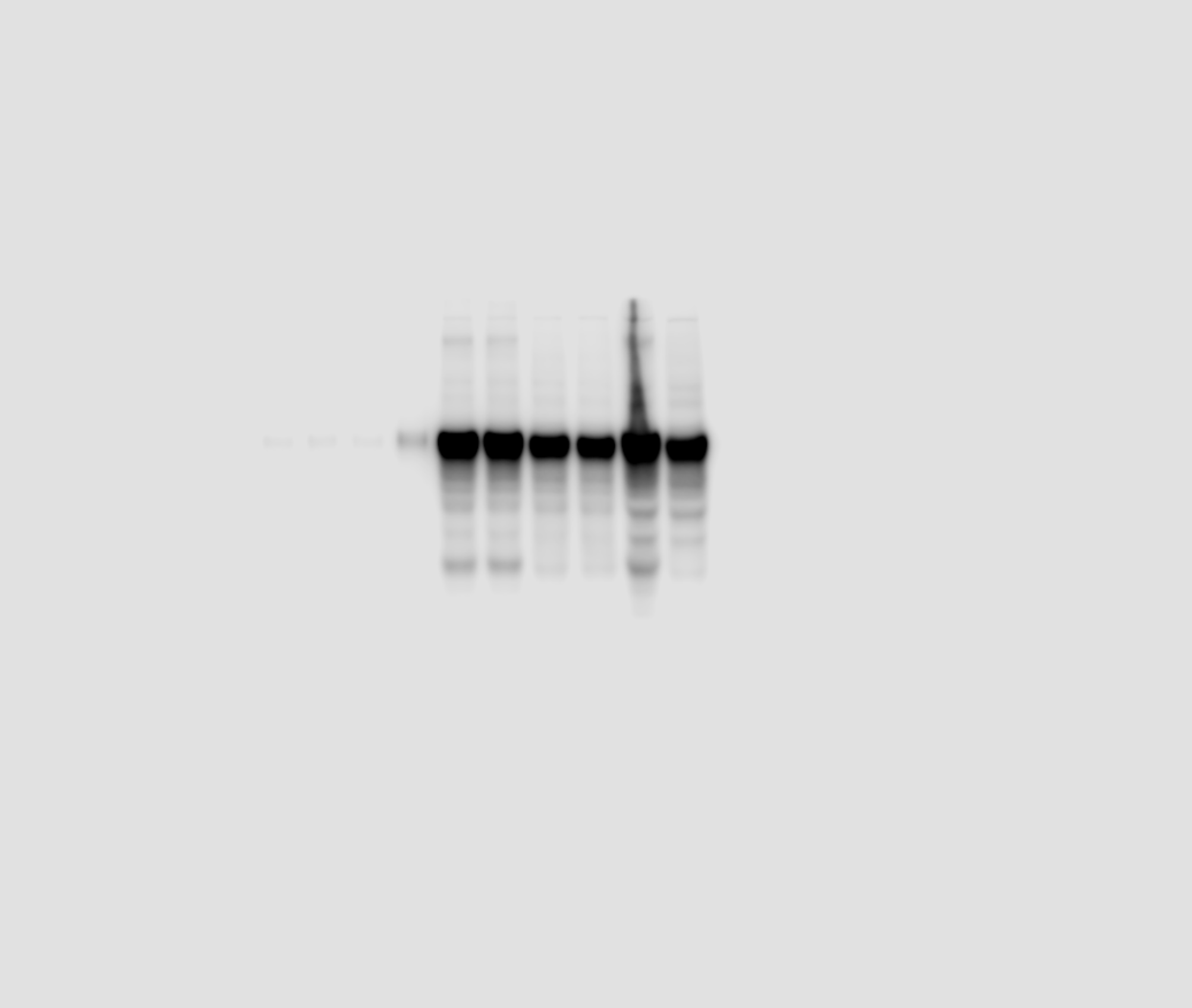

Supplement: Supplementary file 6 — Source data Fig. 4 [file 44321_2025_254_MOESM6_ESM.zip › Figure 4/4D/RARa-LowExposure.tif]

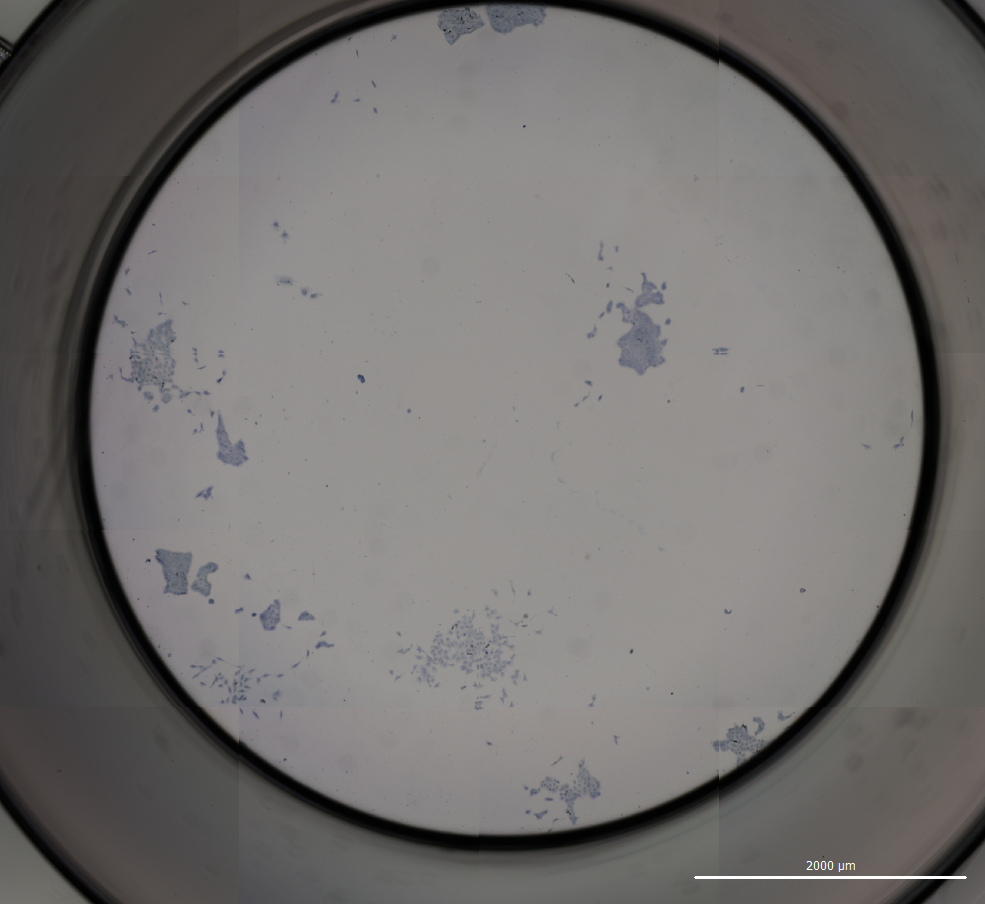

Supplement: Supplementary file 8 — Source data Fig. 6 [file 44321_2025_254_MOESM8_ESM.zip › Figure 6/6E/10uMCIM7.tif]

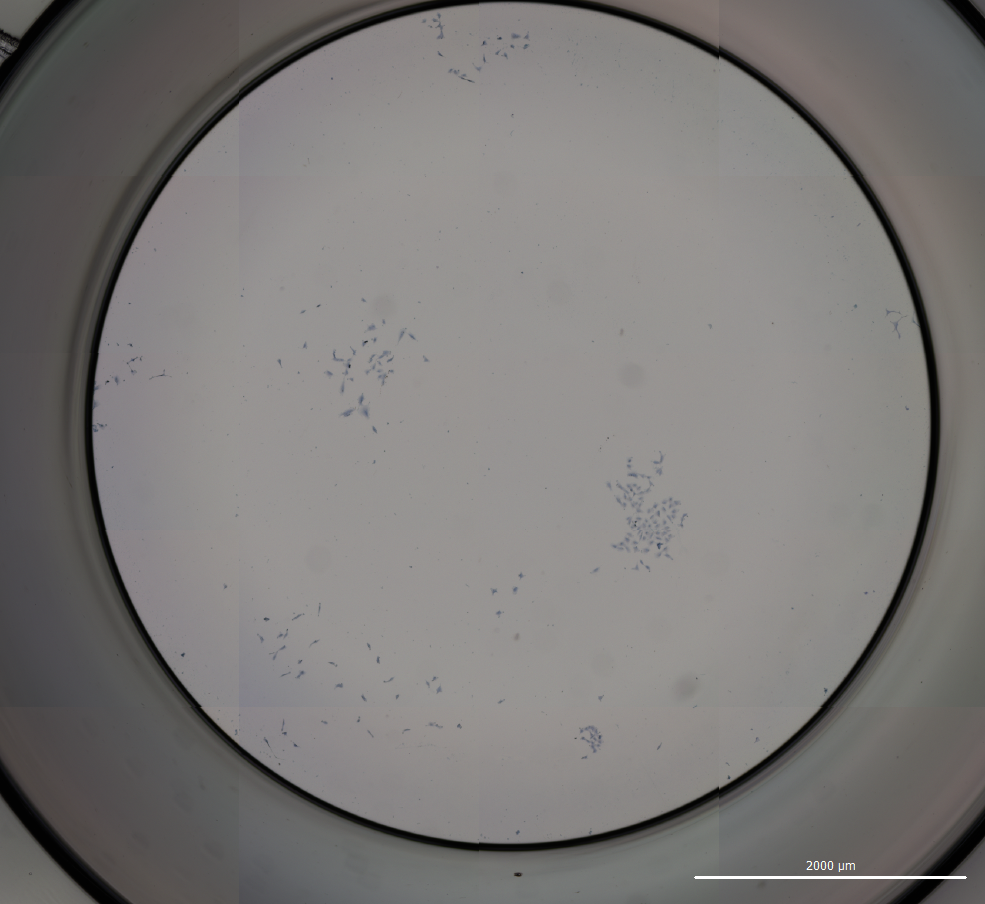

Supplement: Supplementary file 8 — Source data Fig. 6 [file 44321_2025_254_MOESM8_ESM.zip › Figure 6/6E/25uMCIM7.tif]

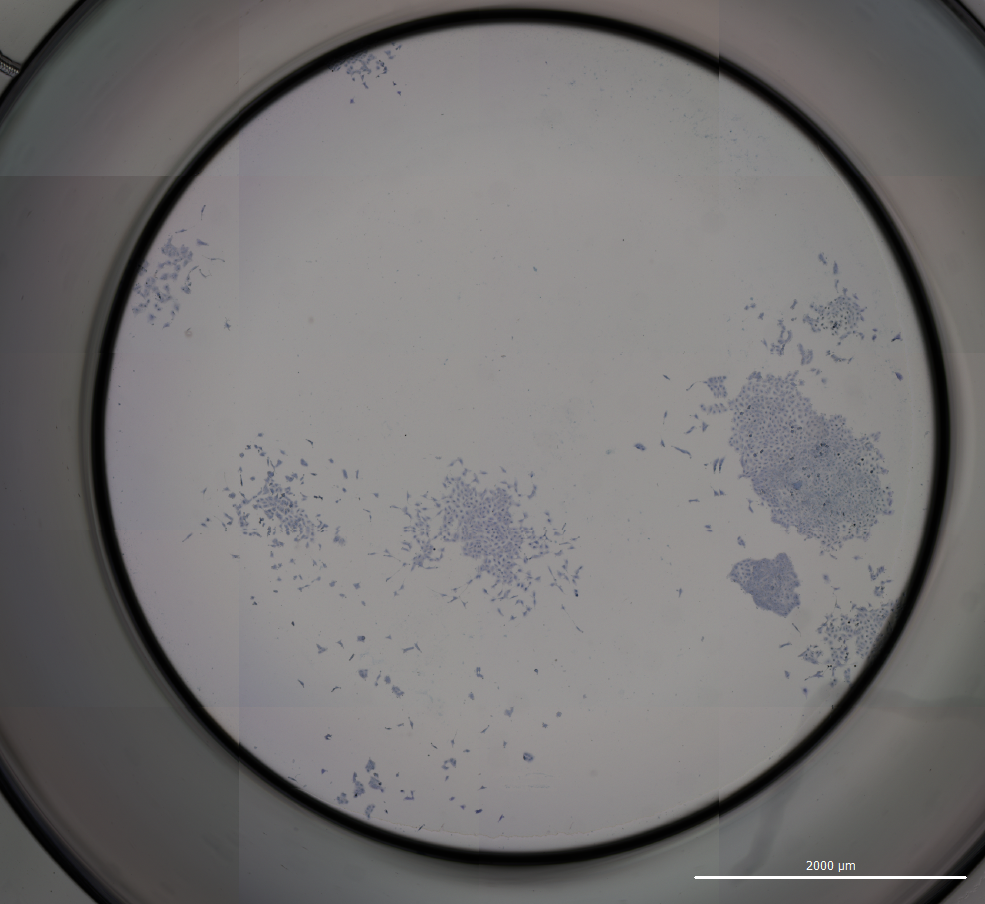

Supplement: Supplementary file 8 — Source data Fig. 6 [file 44321_2025_254_MOESM8_ESM.zip › Figure 6/6E/5uMCIM7.tif]

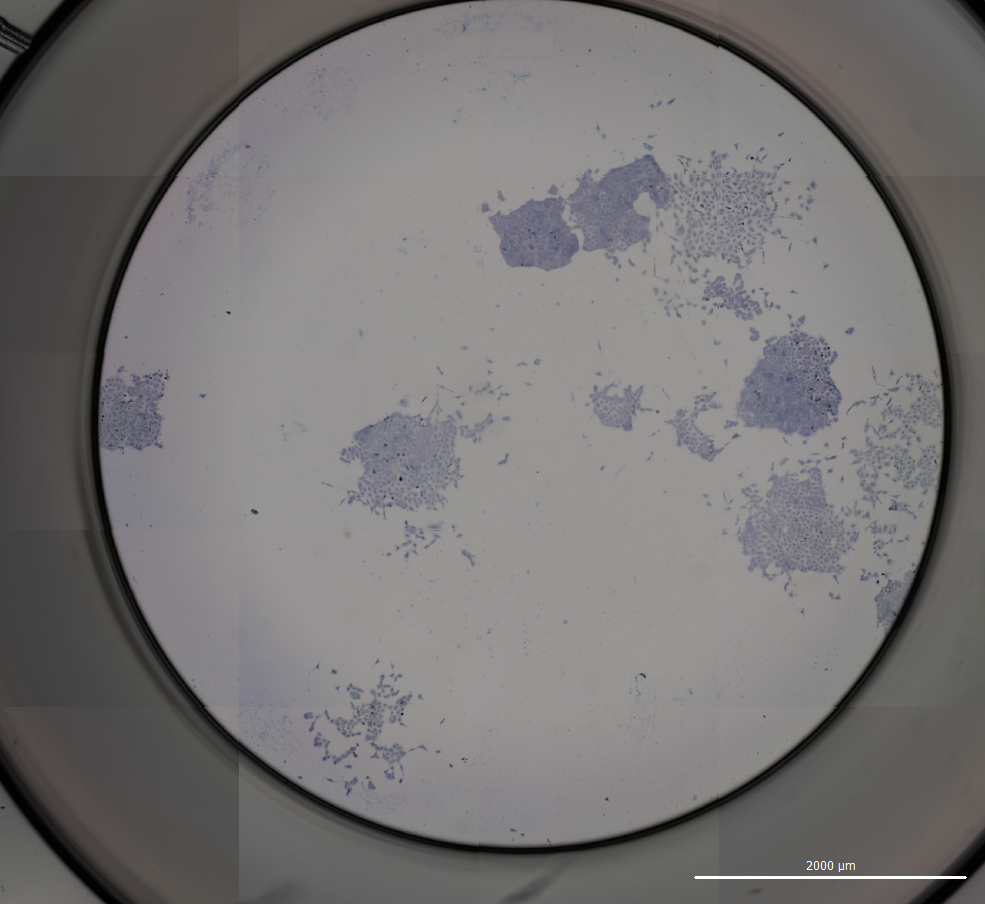

Supplement: Supplementary file 8 — Source data Fig. 6 [file 44321_2025_254_MOESM8_ESM.zip › Figure 6/6E/DMSO.tif]

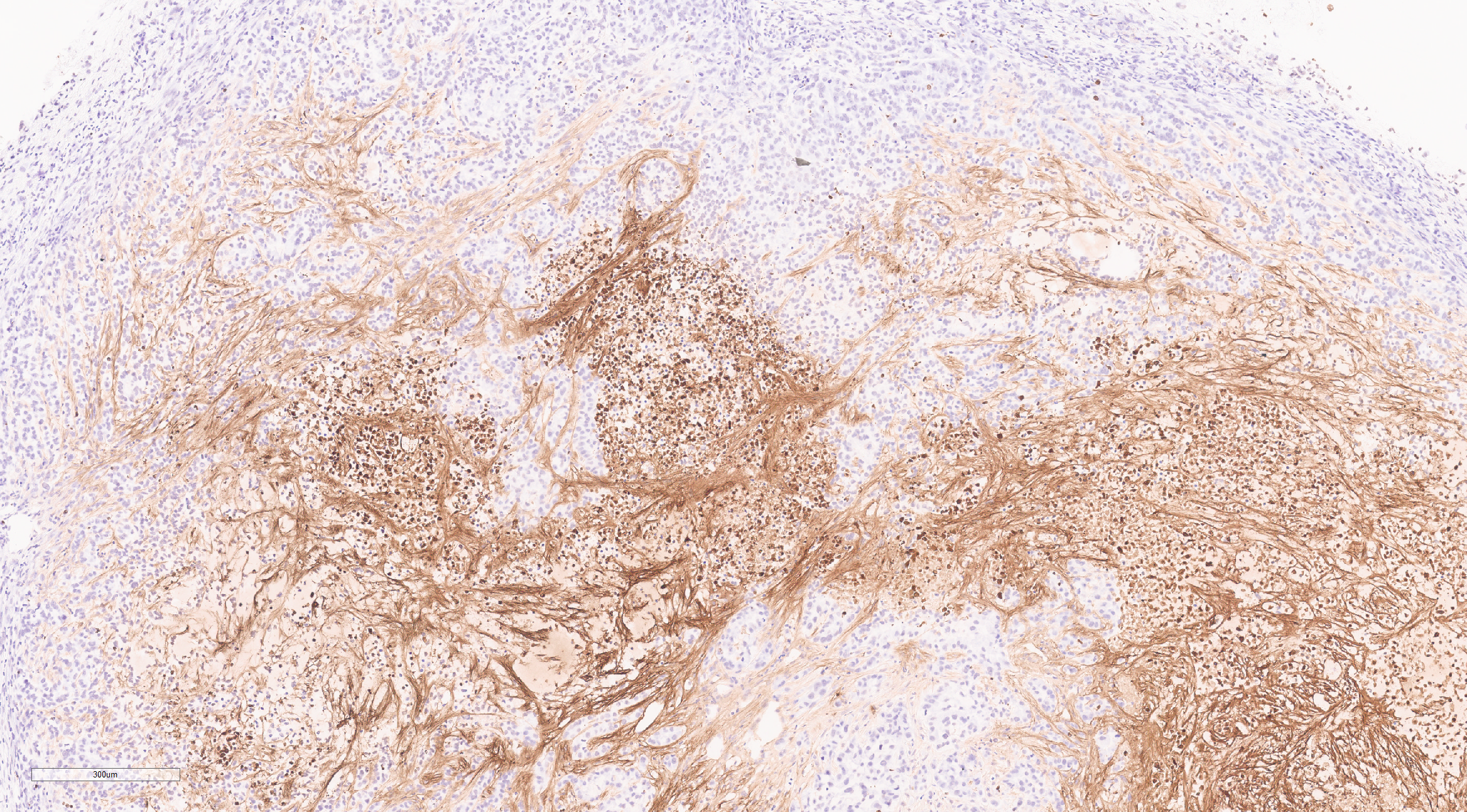

Supplement: Supplementary file 8 — Source data Fig. 6 [file 44321_2025_254_MOESM8_ESM.zip › Figure 6/6I/25mgkg_CIM7.tif]

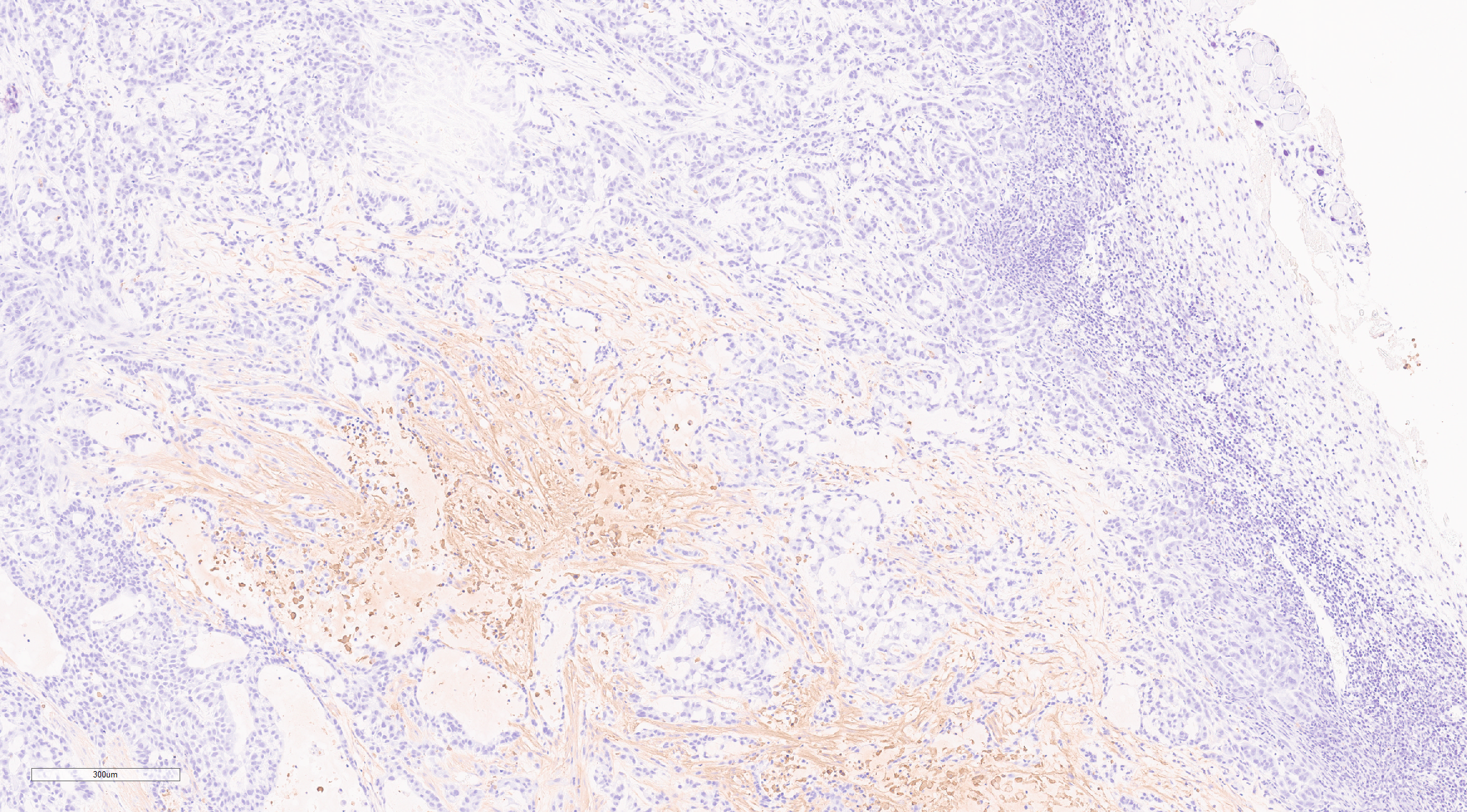

Supplement: Supplementary file 8 — Source data Fig. 6 [file 44321_2025_254_MOESM8_ESM.zip › Figure 6/6I/Vehicle.tif]

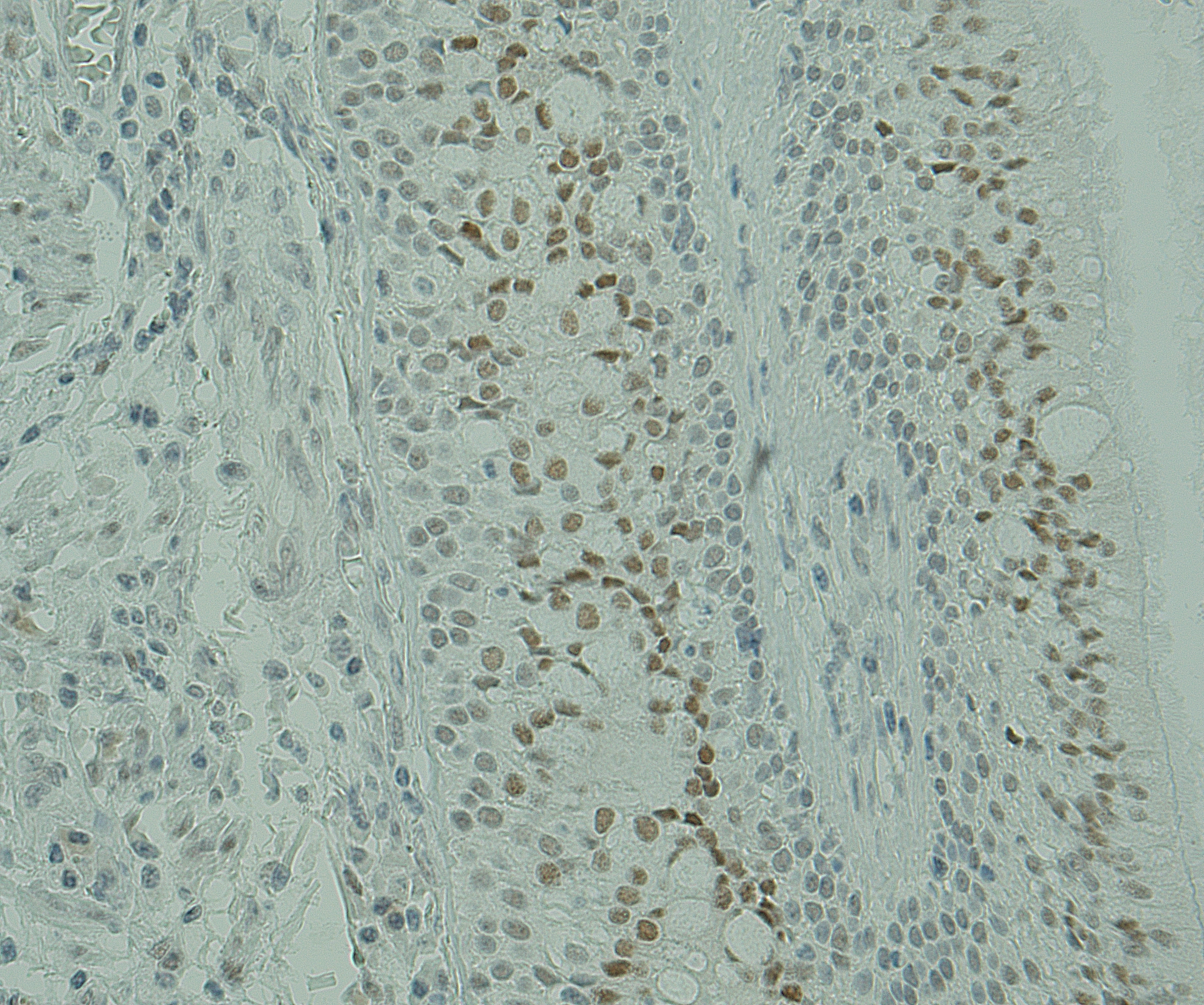

Supplement: Supplementary file 9 — Source data Fig. 7 [file 44321_2025_254_MOESM9_ESM.zip › Figure 7/7A/Grade2-3_Adeno_NCoR1.png]

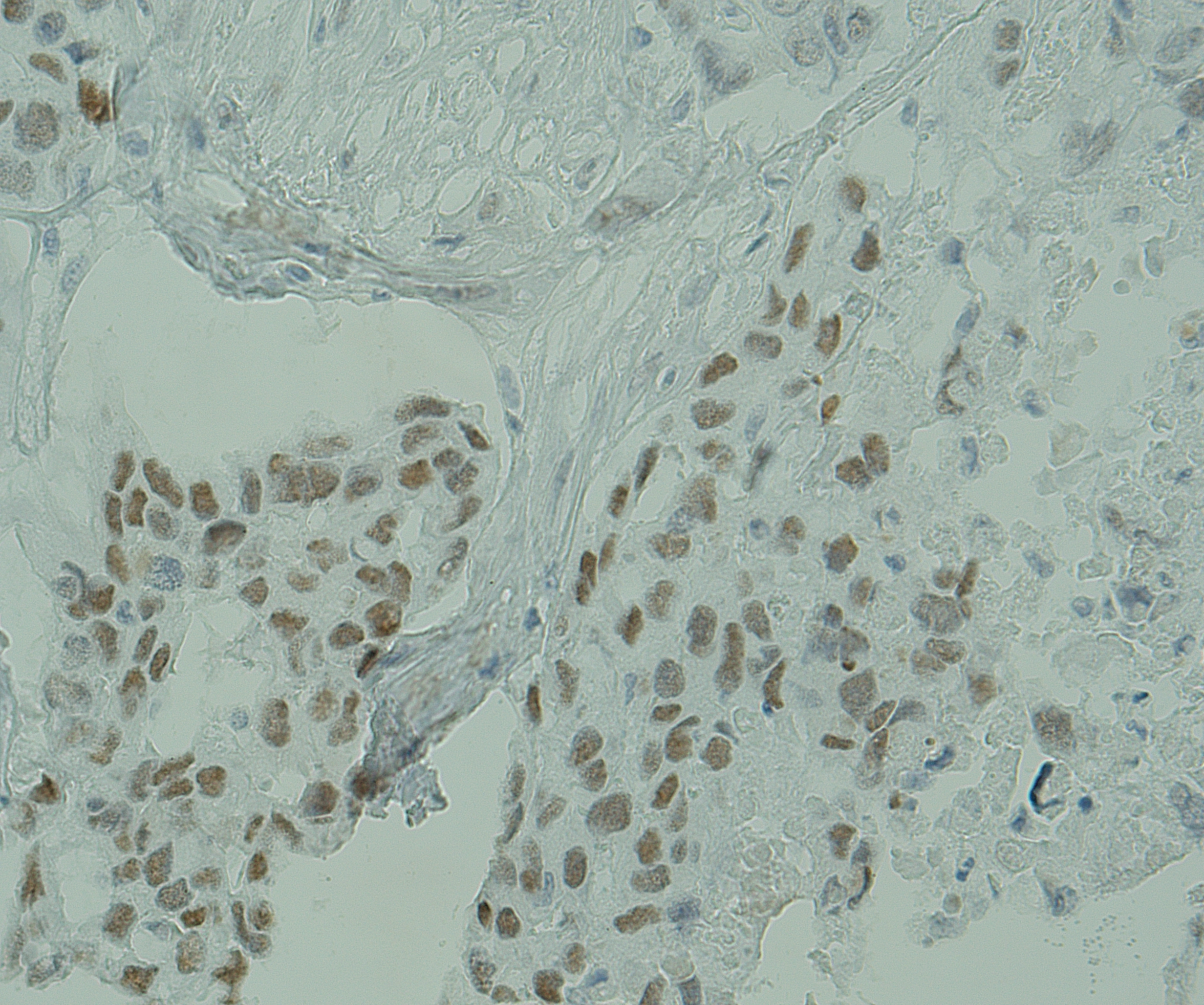

Supplement: Supplementary file 9 — Source data Fig. 7 [file 44321_2025_254_MOESM9_ESM.zip › Figure 7/7A/Grade3_SCC_NCoR1.png]

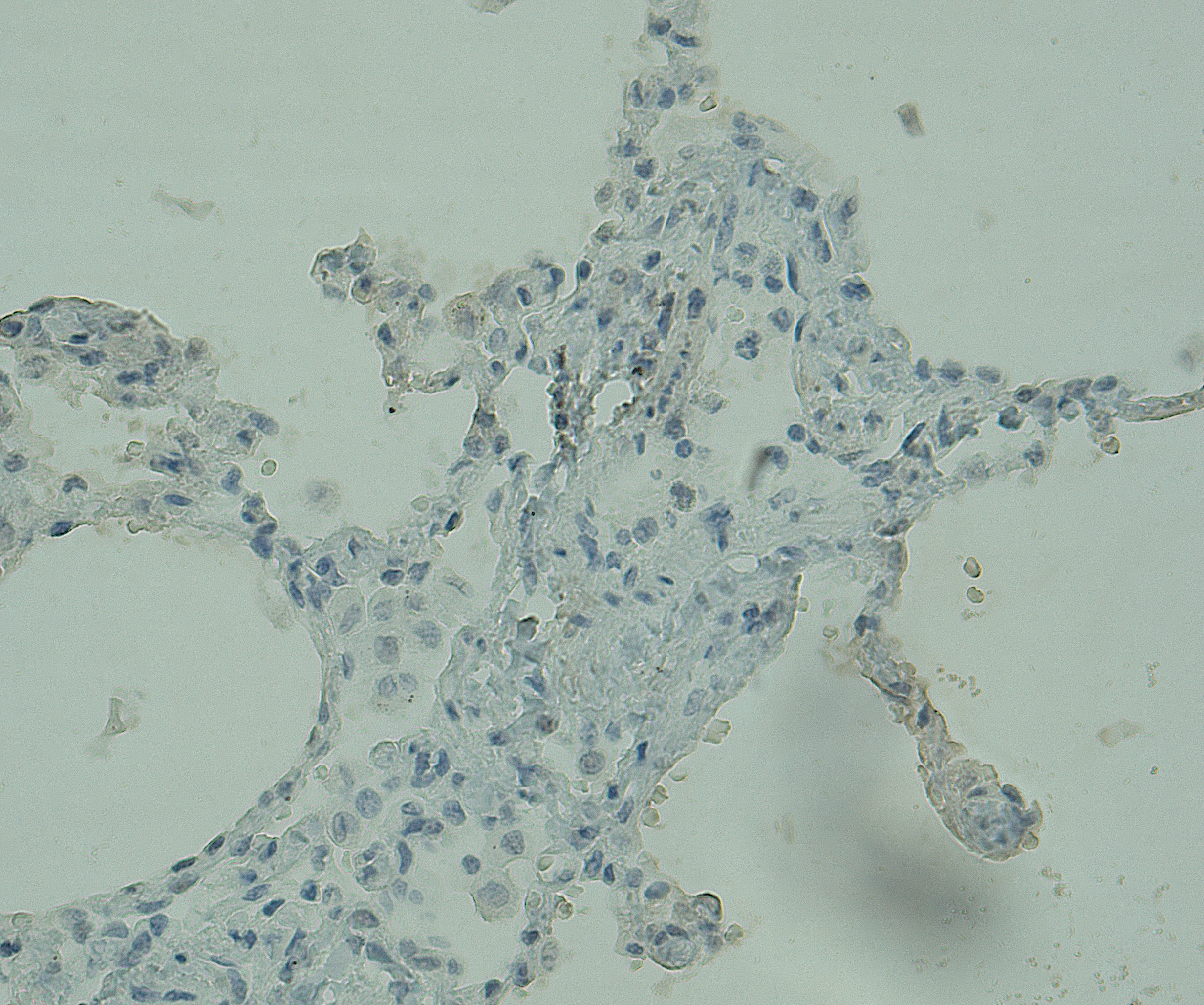

Supplement: Supplementary file 9 — Source data Fig. 7 [file 44321_2025_254_MOESM9_ESM.zip › Figure 7/7A/HealthyLung_NCoR1.png]

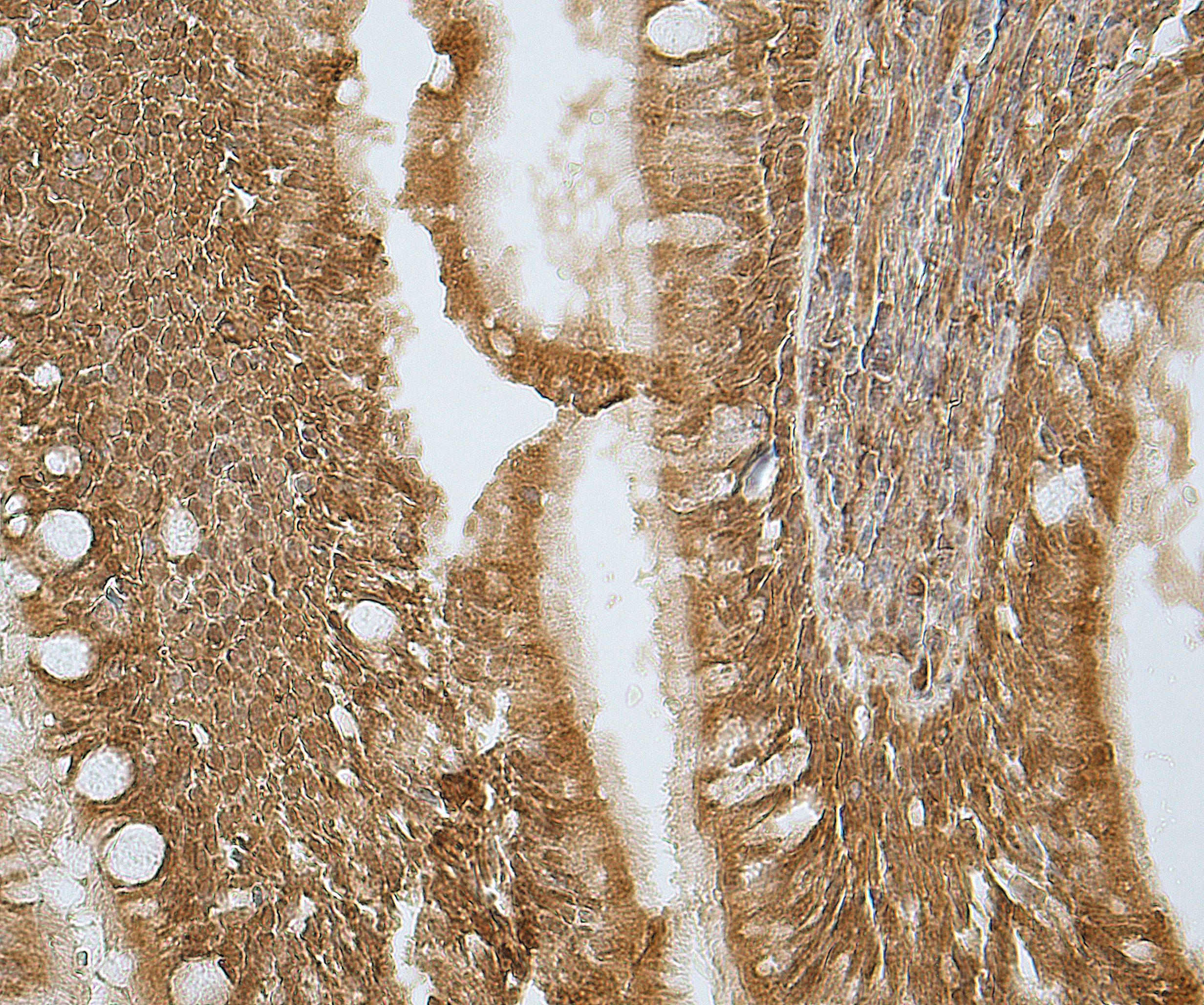

Supplement: Supplementary file 9 — Source data Fig. 7 [file 44321_2025_254_MOESM9_ESM.zip › Figure 7/7B/Grade2-3_Adeno_RARa.tif]

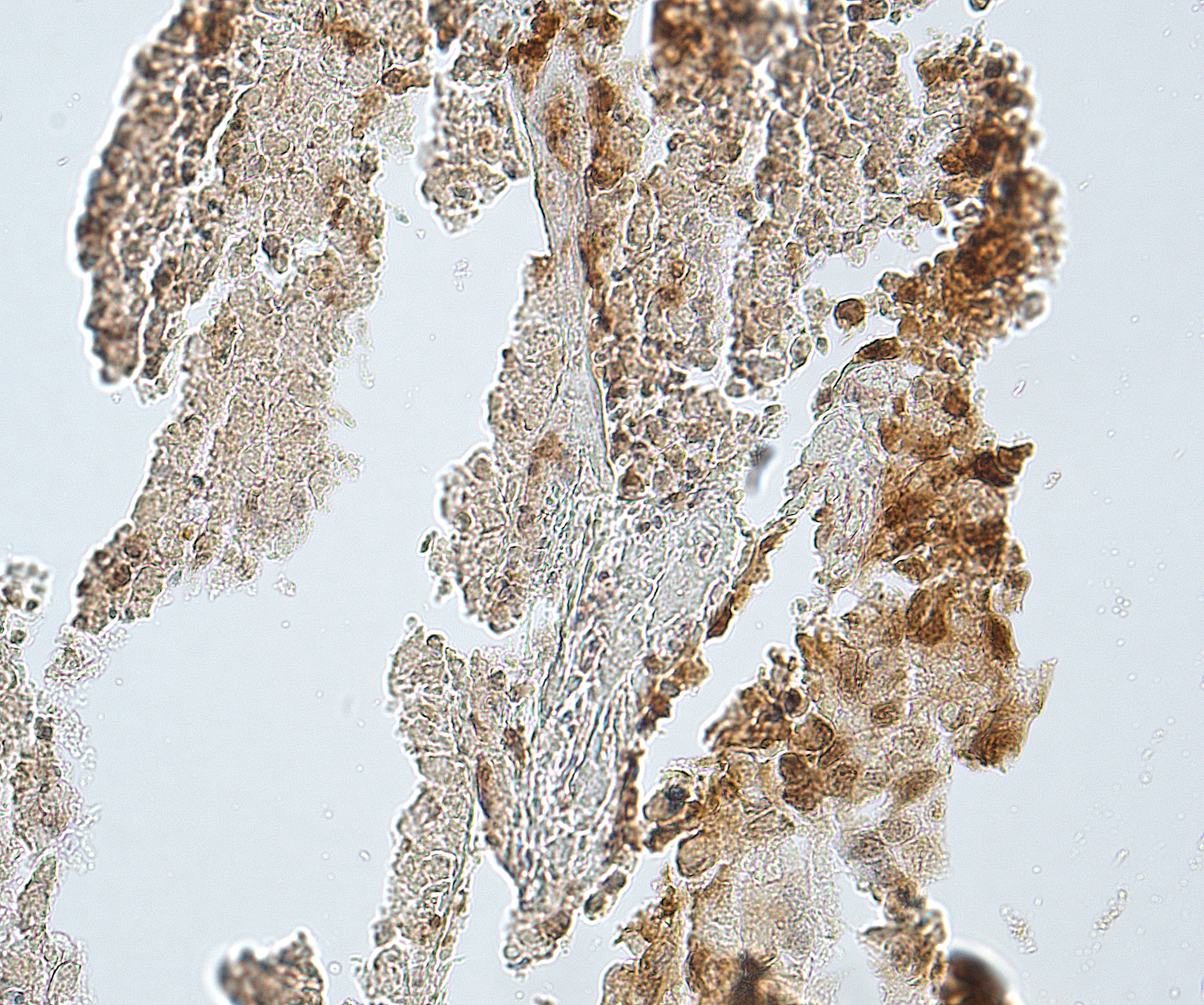

Supplement: Supplementary file 9 — Source data Fig. 7 [file 44321_2025_254_MOESM9_ESM.zip › Figure 7/7B/Grade3_SCC_RARa.tif]

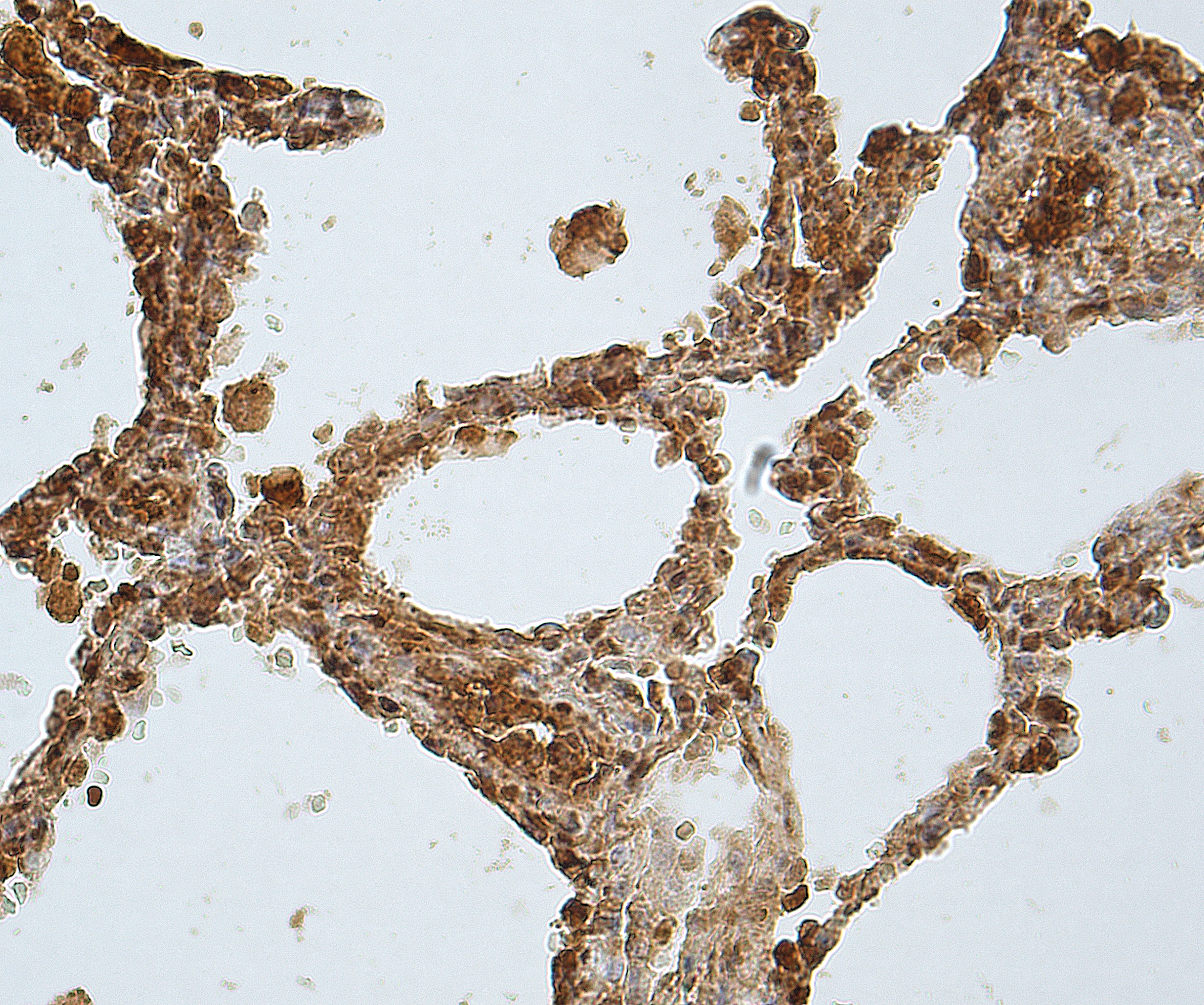

Supplement: Supplementary file 9 — Source data Fig. 7 [file 44321_2025_254_MOESM9_ESM.zip › Figure 7/7B/HealthyLung_RARa.tif]

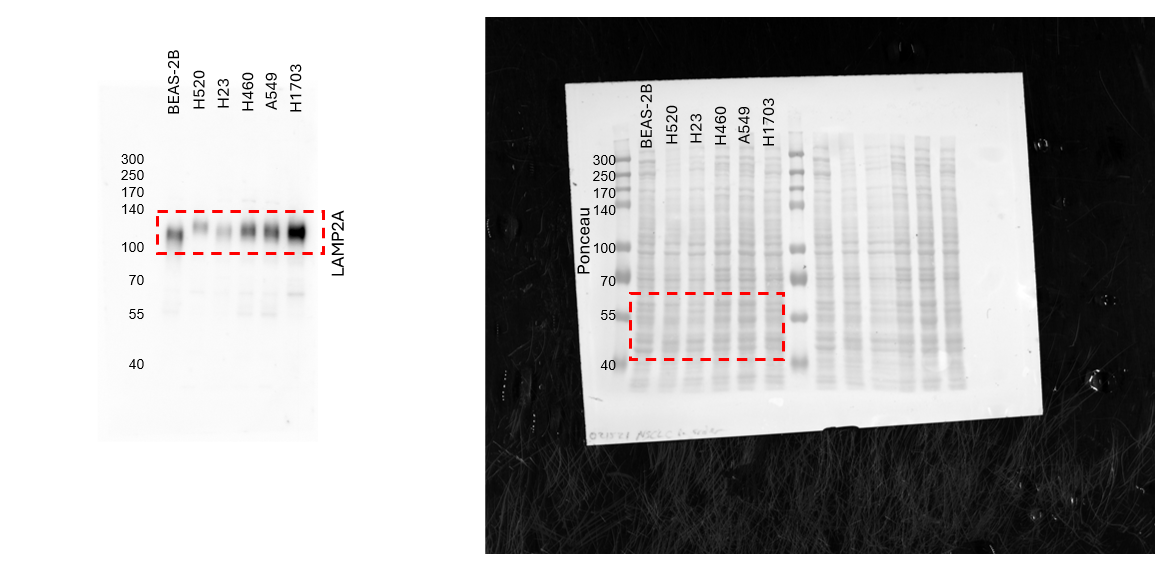

Supplement: Supplementary file 10 — Figure EV1 Source Data [file 44321_2025_254_MOESM10_ESM.zip › EV1/EV1B/AnnotatedBlots.png]

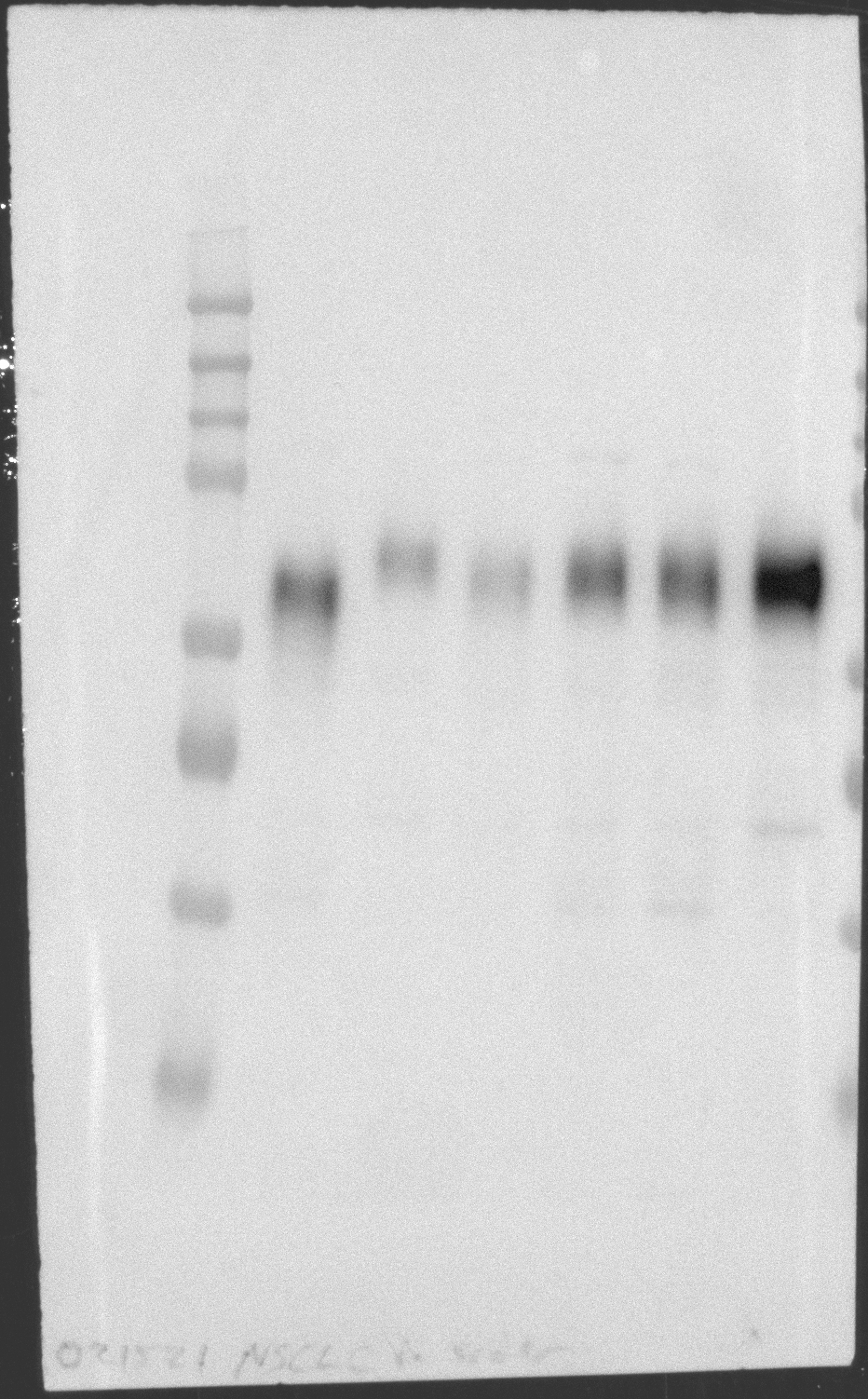

Supplement: Supplementary file 10 — Figure EV1 Source Data [file 44321_2025_254_MOESM10_ESM.zip › EV1/EV1B/L2A.tif]

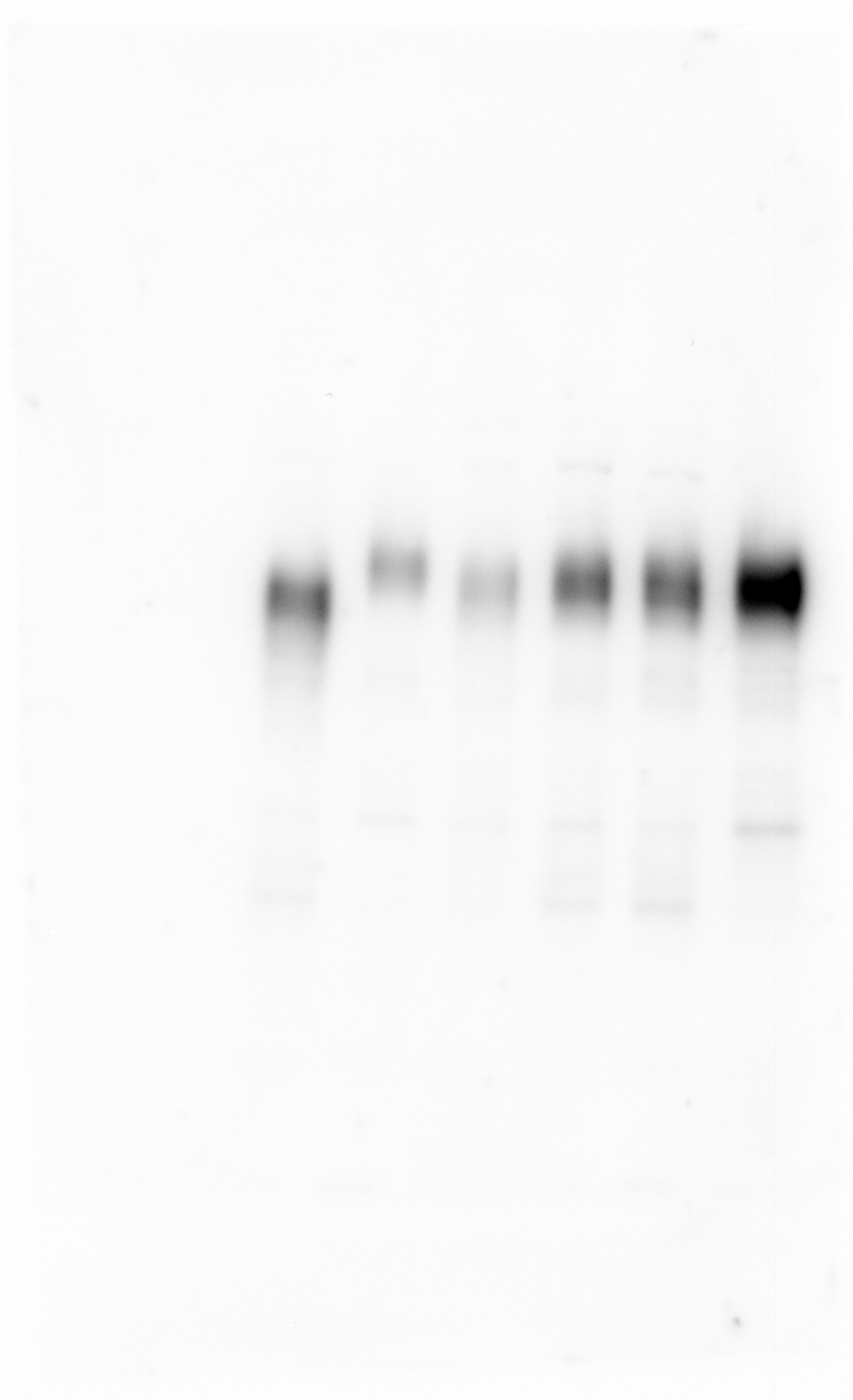

Supplement: Supplementary file 10 — Figure EV1 Source Data [file 44321_2025_254_MOESM10_ESM.zip › EV1/EV1B/L2A-Bands.tif]

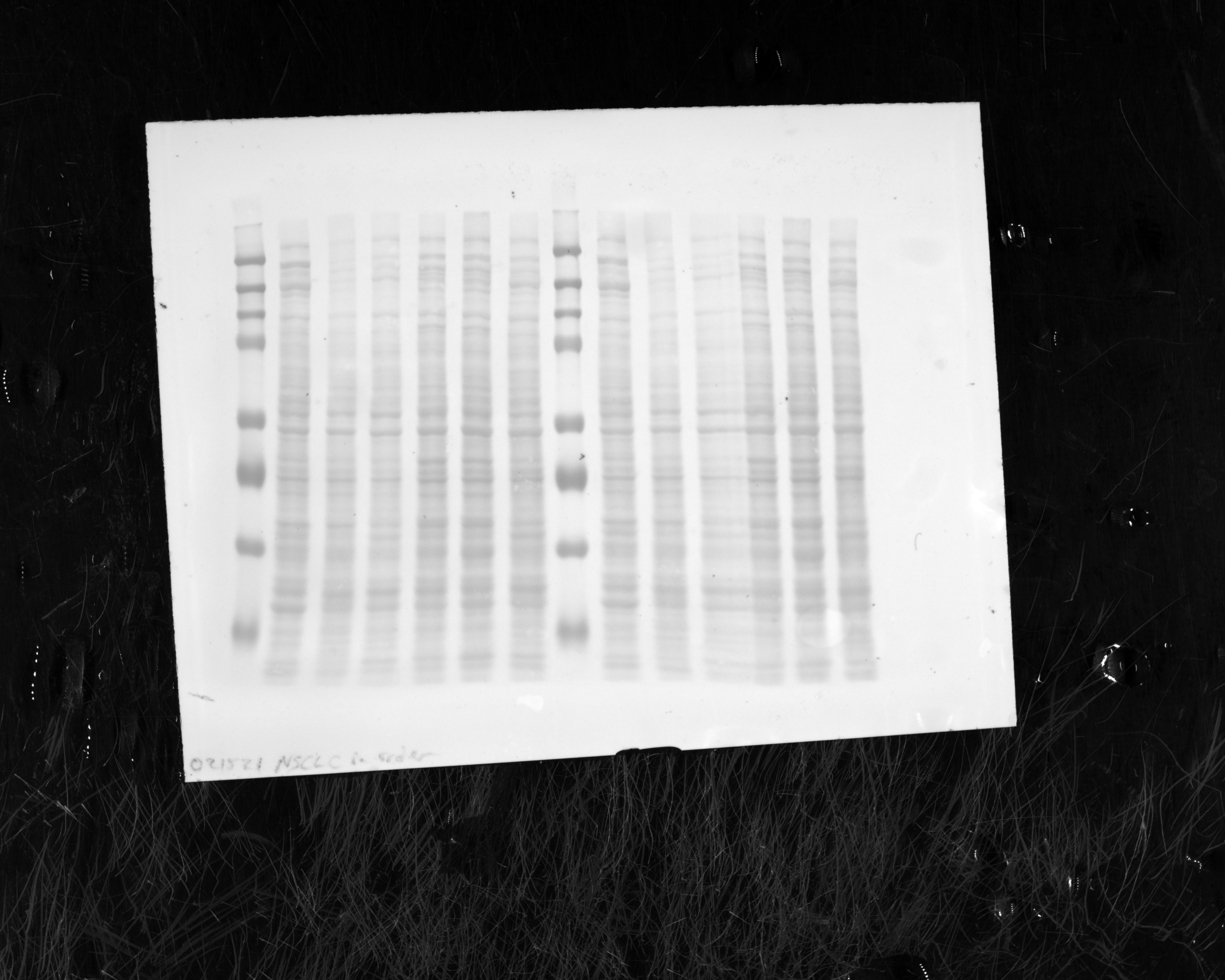

Supplement: Supplementary file 10 — Figure EV1 Source Data [file 44321_2025_254_MOESM10_ESM.zip › EV1/EV1B/PON.tif]

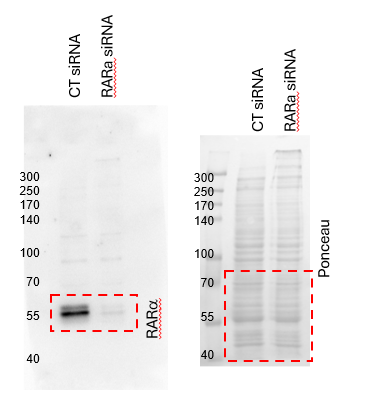

Supplement: Supplementary file 11 — Figure EV2 Source Data [file 44321_2025_254_MOESM11_ESM.zip › EV2/EV2A/Annotated-Blots.png]

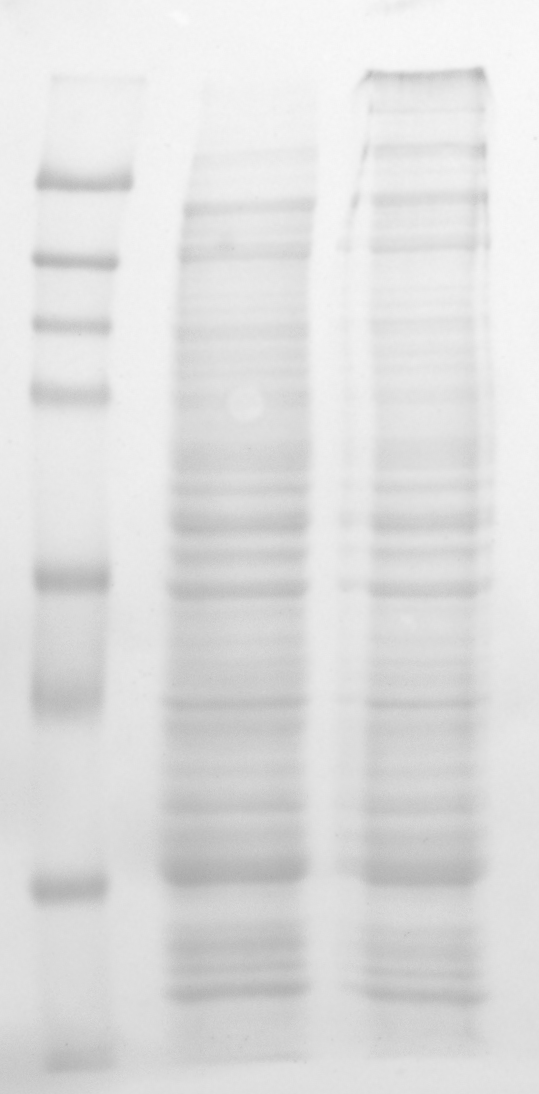

Supplement: Supplementary file 11 — Figure EV2 Source Data [file 44321_2025_254_MOESM11_ESM.zip › EV2/EV2A/Ponceau.tif]

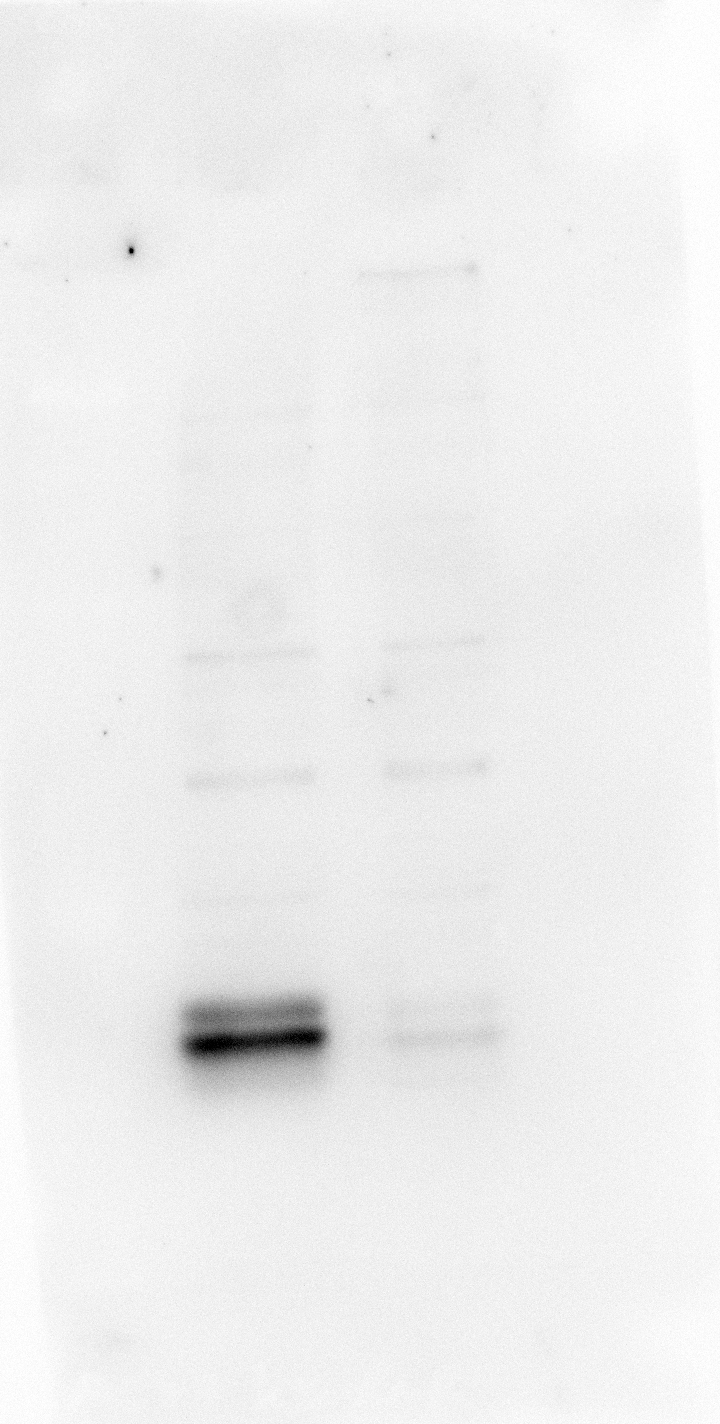

Supplement: Supplementary file 11 — Figure EV2 Source Data [file 44321_2025_254_MOESM11_ESM.zip › EV2/EV2A/RARa.tif]

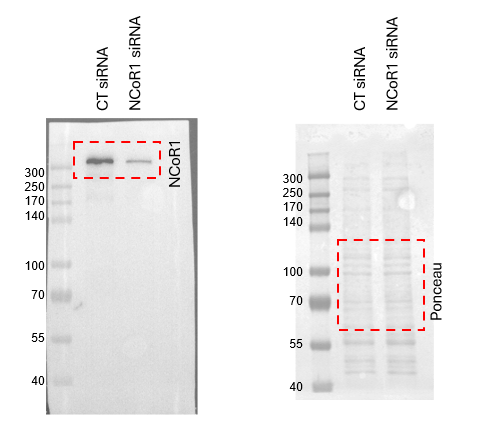

Supplement: Supplementary file 11 — Figure EV2 Source Data [file 44321_2025_254_MOESM11_ESM.zip › EV2/EV2D/Annotated-Blots.png]

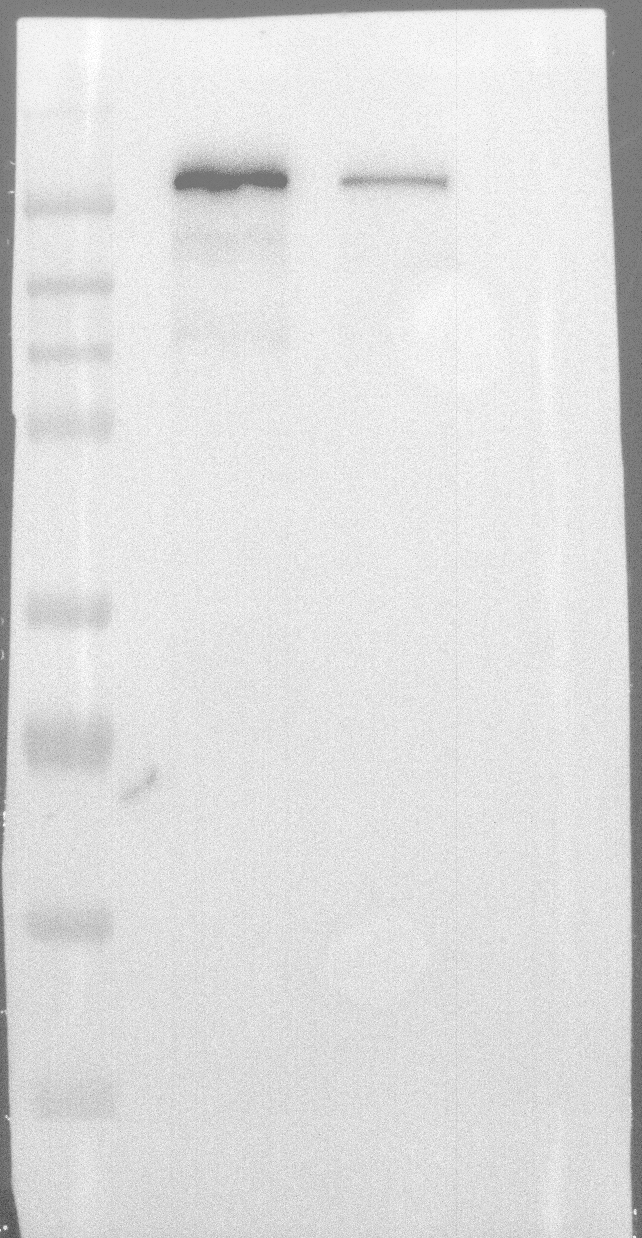

Supplement: Supplementary file 11 — Figure EV2 Source Data [file 44321_2025_254_MOESM11_ESM.zip › EV2/EV2D/NCoR1.tif]

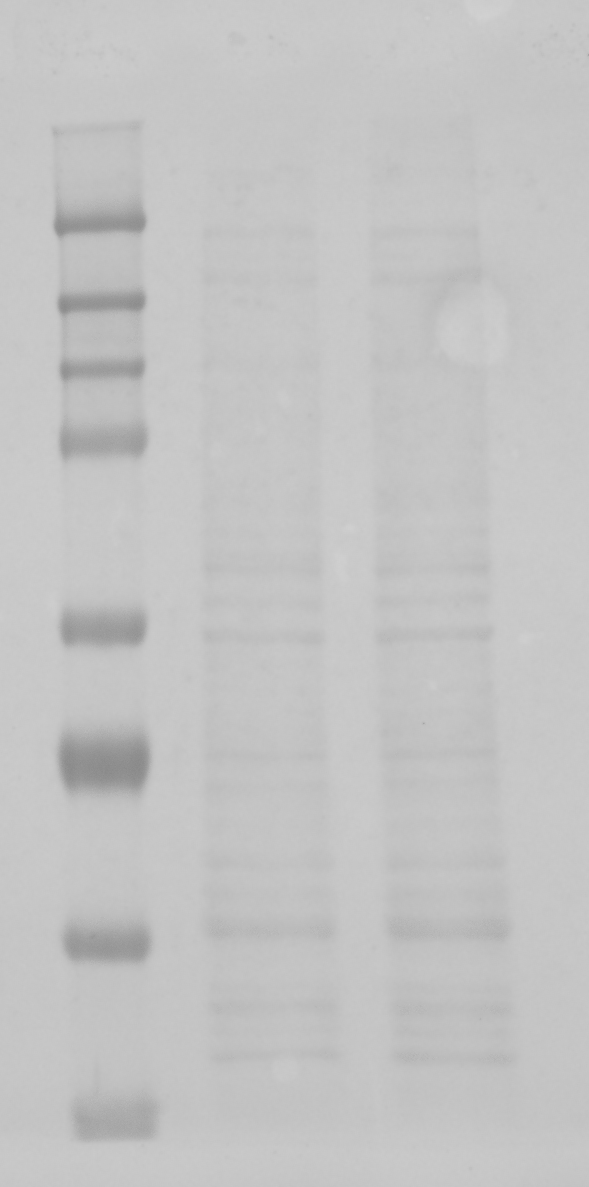

Supplement: Supplementary file 11 — Figure EV2 Source Data [file 44321_2025_254_MOESM11_ESM.zip › EV2/EV2D/Ponceau.tif]

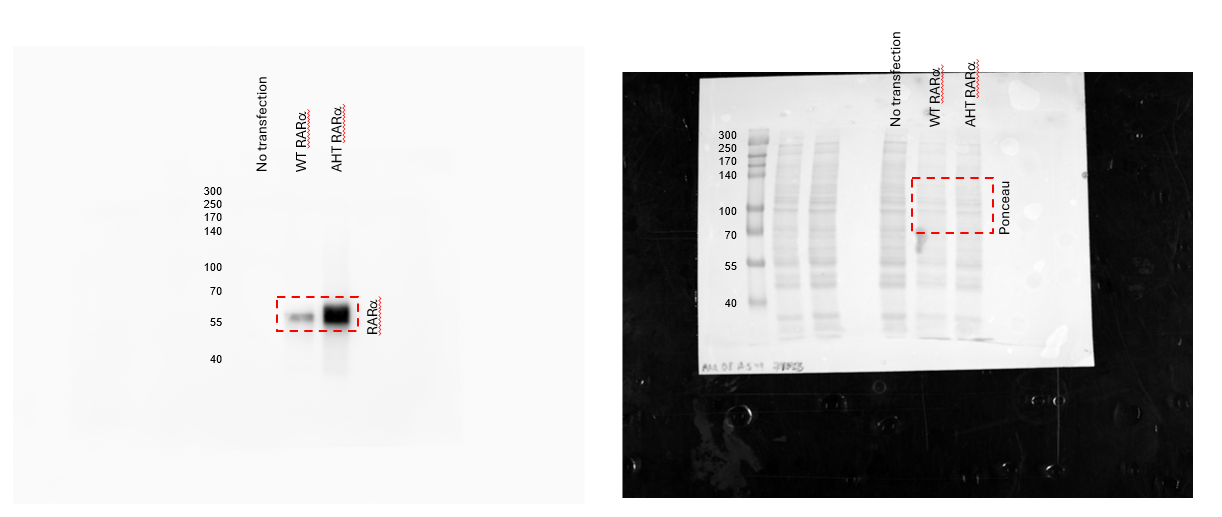

Supplement: Supplementary file 11 — Figure EV2 Source Data [file 44321_2025_254_MOESM11_ESM.zip › EV2/EV2E/Annotated-Blots.png]

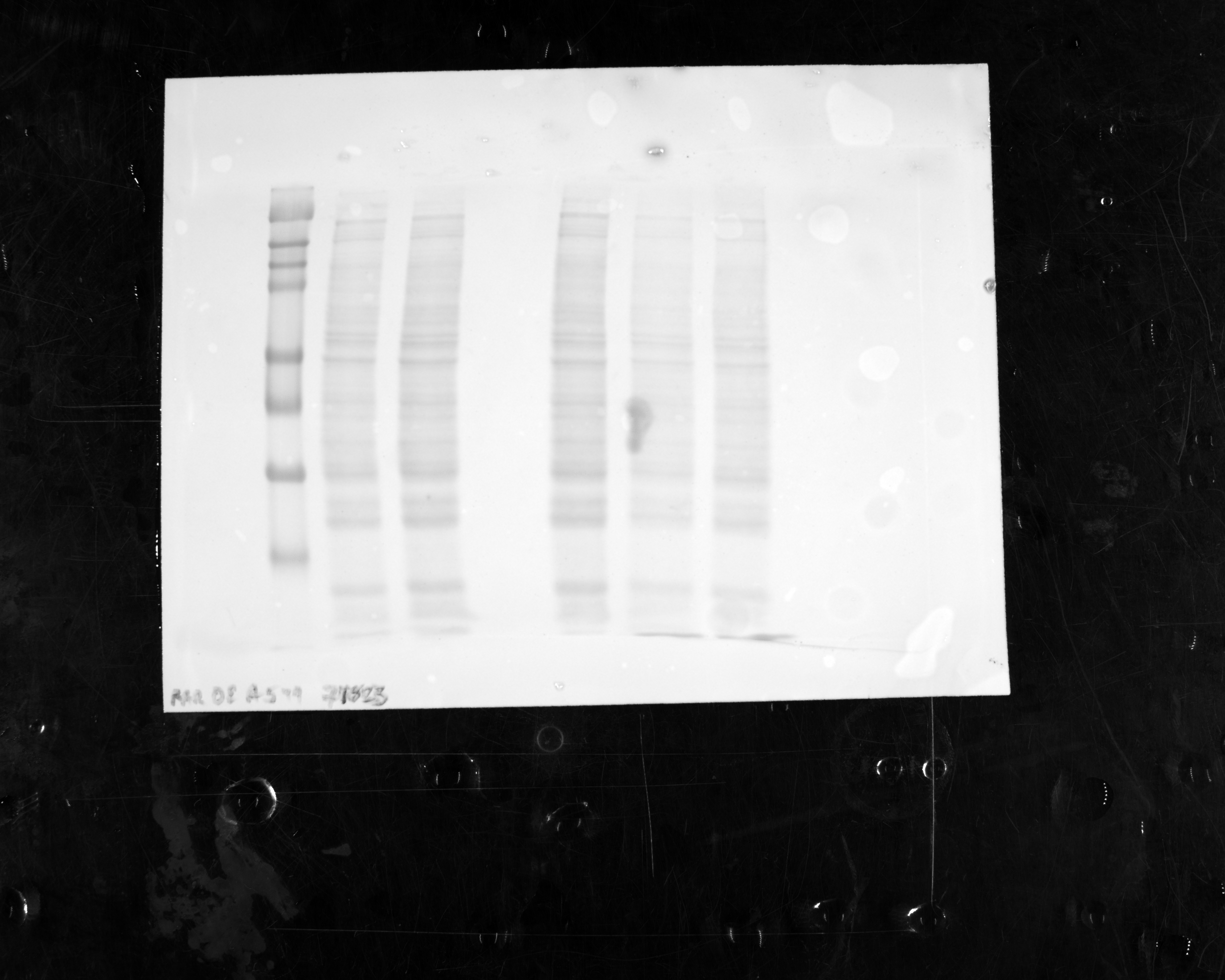

Supplement: Supplementary file 11 — Figure EV2 Source Data [file 44321_2025_254_MOESM11_ESM.zip › EV2/EV2E/Ponceau.tif]

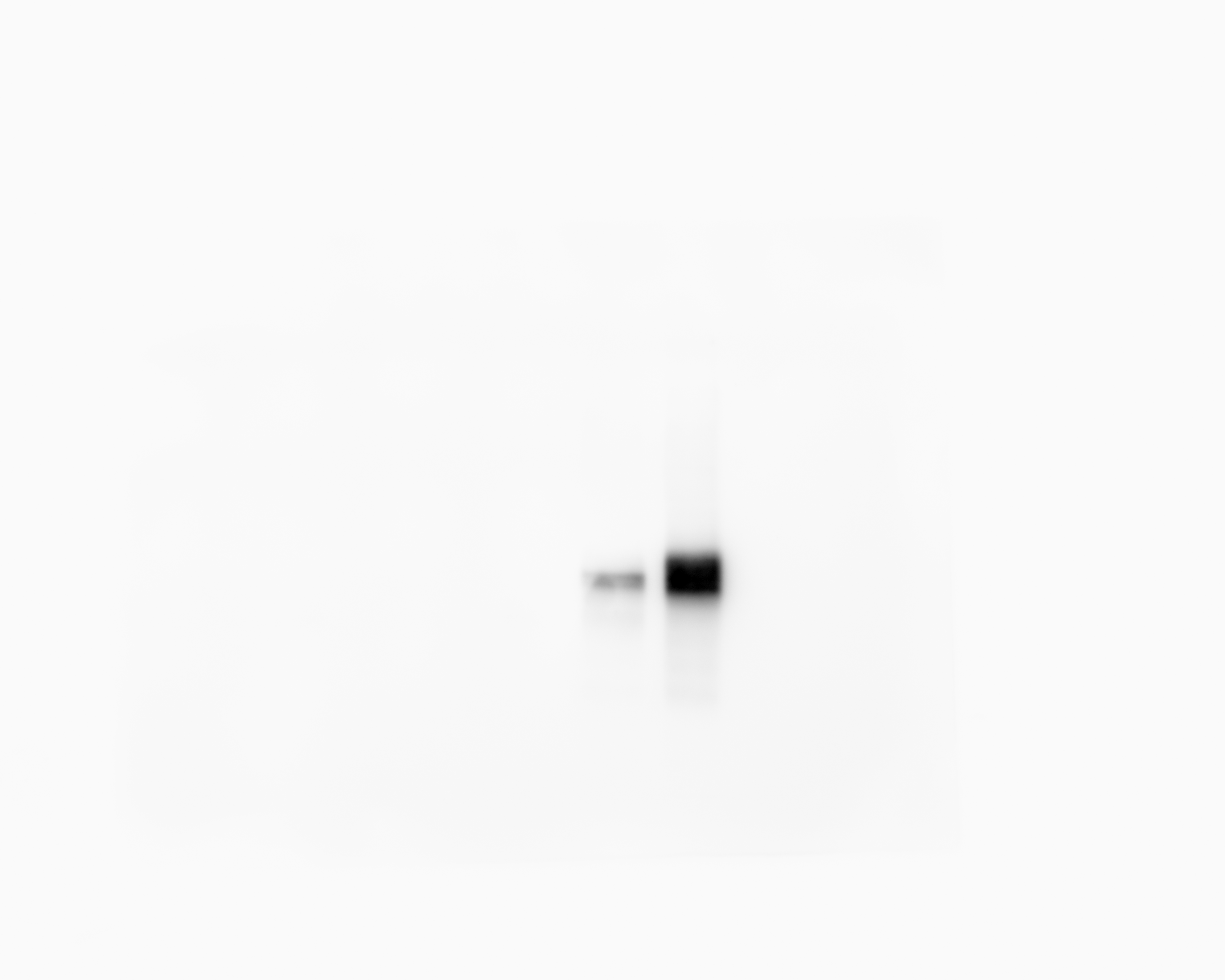

Supplement: Supplementary file 11 — Figure EV2 Source Data [file 44321_2025_254_MOESM11_ESM.zip › EV2/EV2E/RARa.tif]

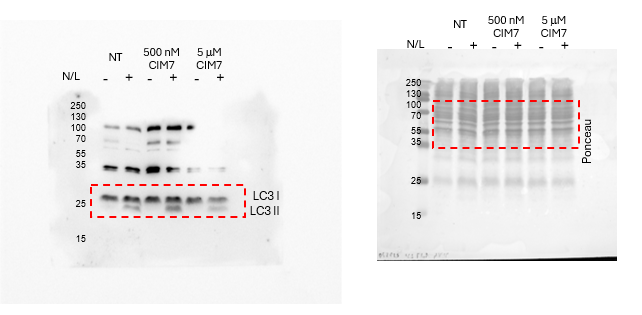

Supplement: Supplementary file 12 — Figure EV3 Source Data [file 44321_2025_254_MOESM12_ESM.zip › EV3/EV3G/AnnotatedBlots.png]

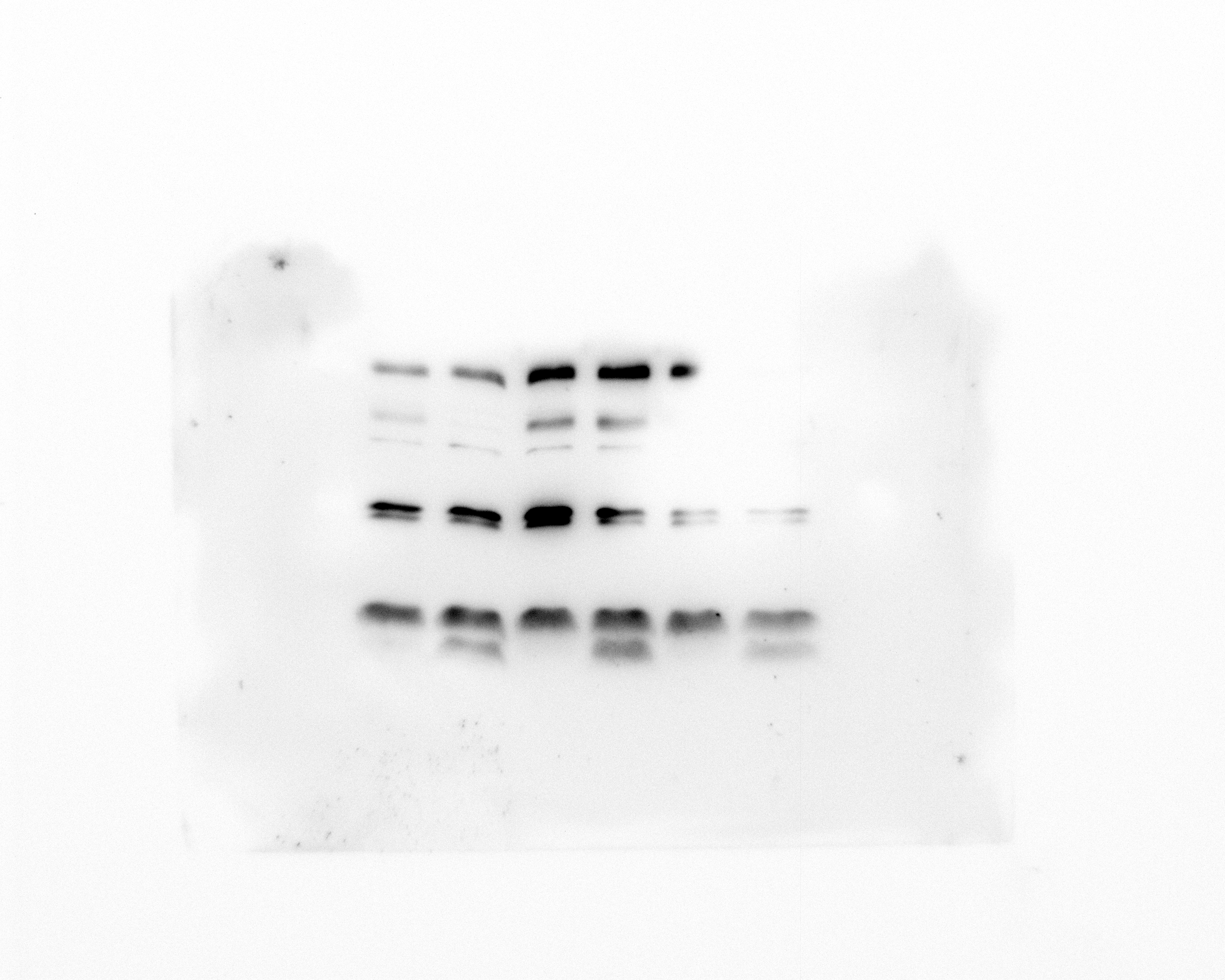

Supplement: Supplementary file 12 — Figure EV3 Source Data [file 44321_2025_254_MOESM12_ESM.zip › EV3/EV3G/lc3-bands.tif]

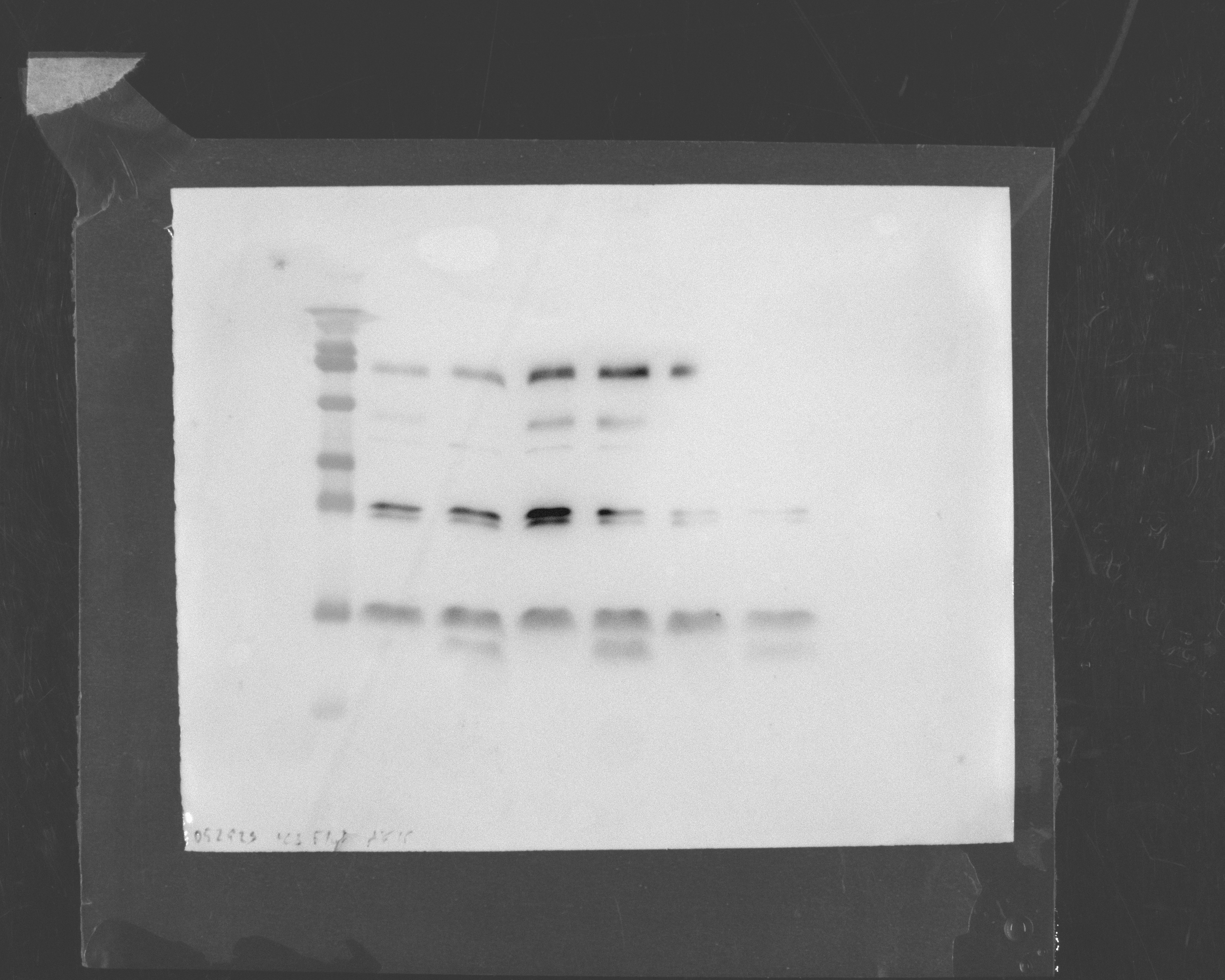

Supplement: Supplementary file 12 — Figure EV3 Source Data [file 44321_2025_254_MOESM12_ESM.zip › EV3/EV3G/lc3-total.tif]

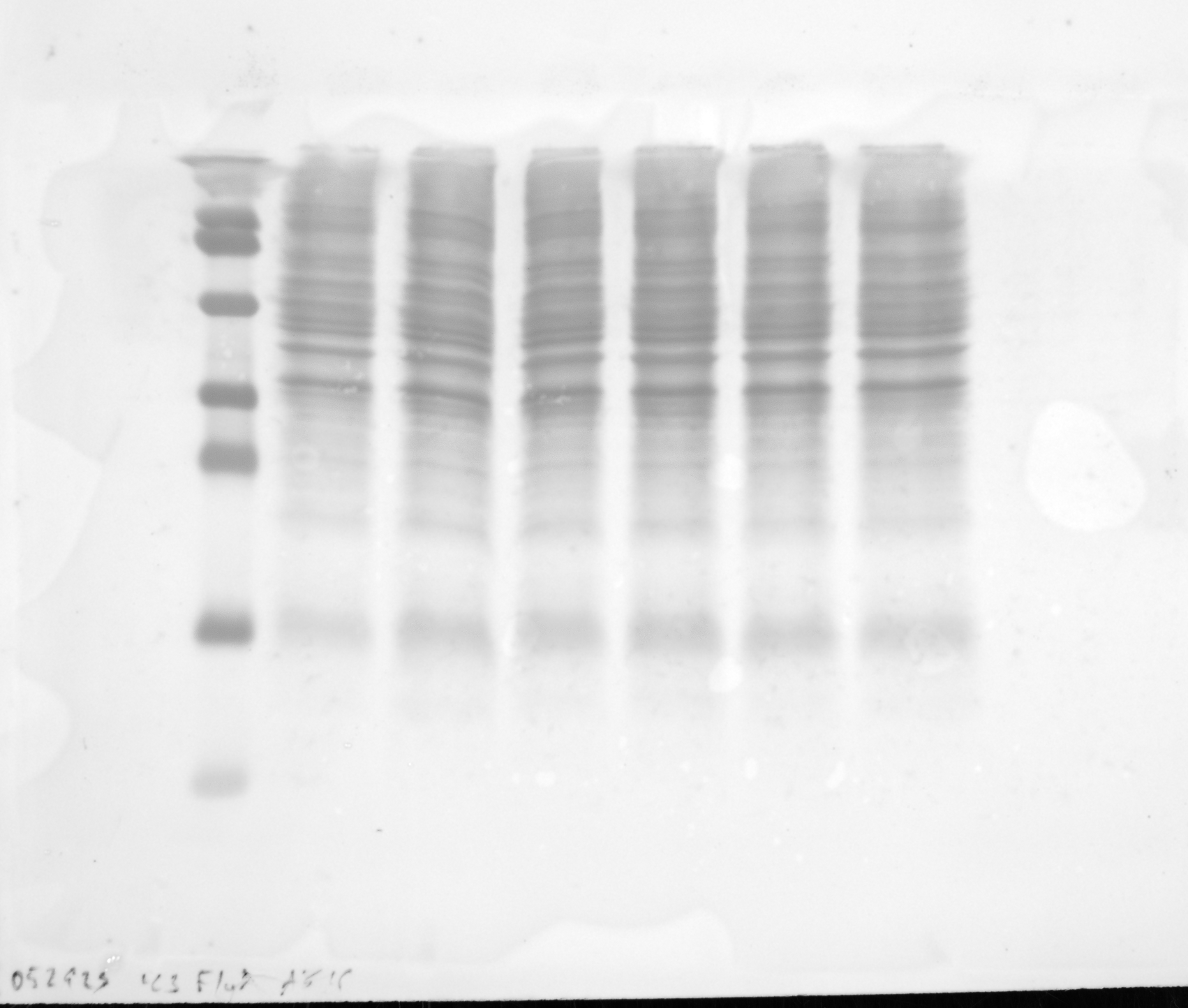

Supplement: Supplementary file 12 — Figure EV3 Source Data [file 44321_2025_254_MOESM12_ESM.zip › EV3/EV3G/ponceau.tif]

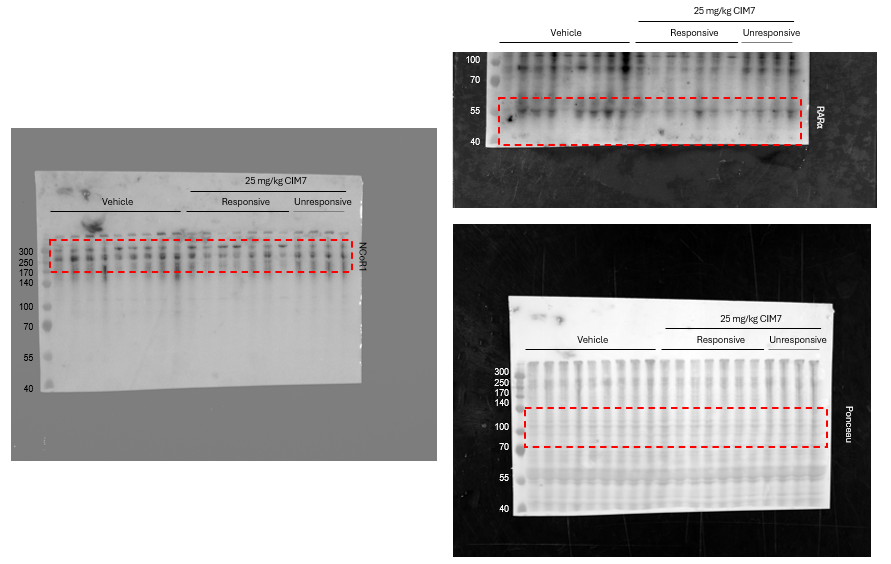

Supplement: Supplementary file 17 — Figures Appendix Source Data [file 44321_2025_254_MOESM17_ESM.zip › FigS11A/Annotated-Blots.png]

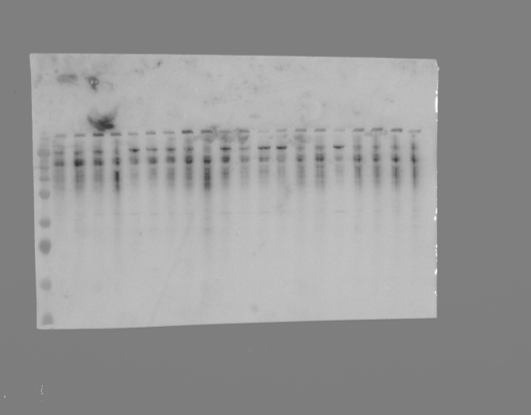

Supplement: Supplementary file 17 — Figures Appendix Source Data [file 44321_2025_254_MOESM17_ESM.zip › FigS11A/NCoR1.tif]

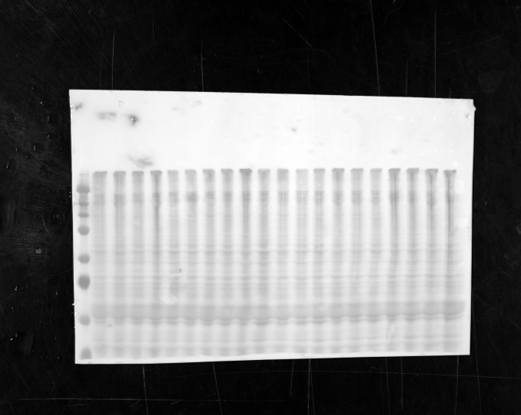

Supplement: Supplementary file 17 — Figures Appendix Source Data [file 44321_2025_254_MOESM17_ESM.zip › FigS11A/Ponceau.tif]

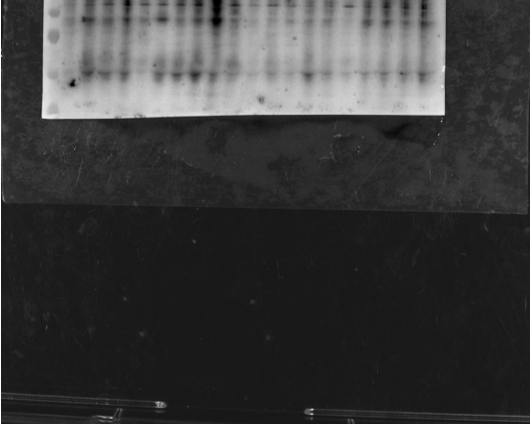

Supplement: Supplementary file 17 — Figures Appendix Source Data [file 44321_2025_254_MOESM17_ESM.zip › FigS11A/RARa.tif]

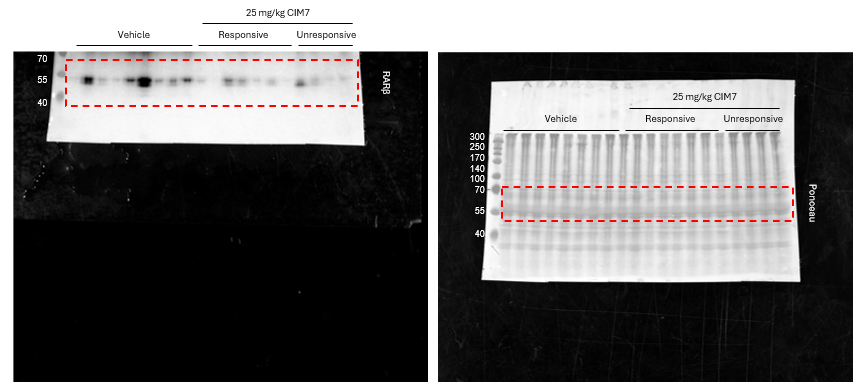

Supplement: Supplementary file 17 — Figures Appendix Source Data [file 44321_2025_254_MOESM17_ESM.zip › FigS11D/Annotated-Blots-RARb.png]

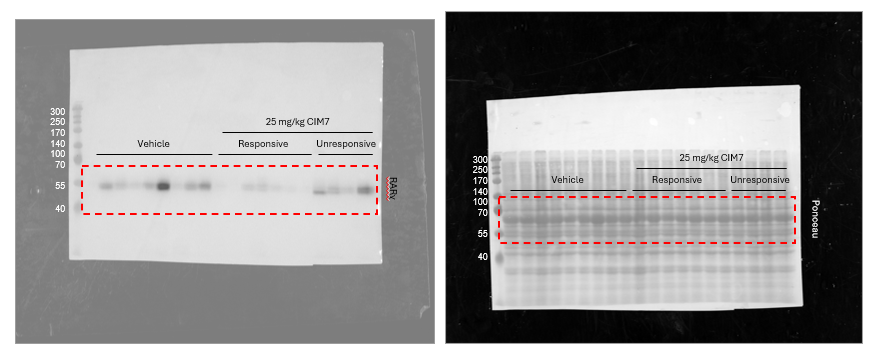

Supplement: Supplementary file 17 — Figures Appendix Source Data [file 44321_2025_254_MOESM17_ESM.zip › FigS11D/Annotated-Blots-RARy.png]

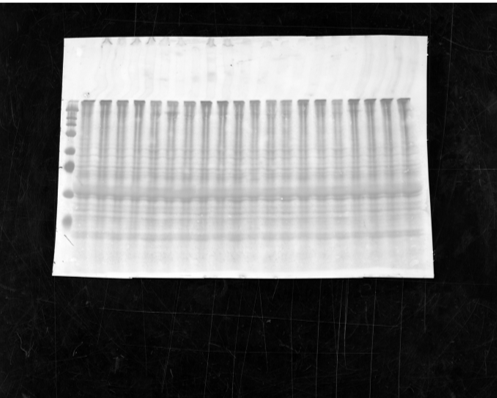

Supplement: Supplementary file 17 — Figures Appendix Source Data [file 44321_2025_254_MOESM17_ESM.zip › FigS11D/Ponceau-forRARb.tif]

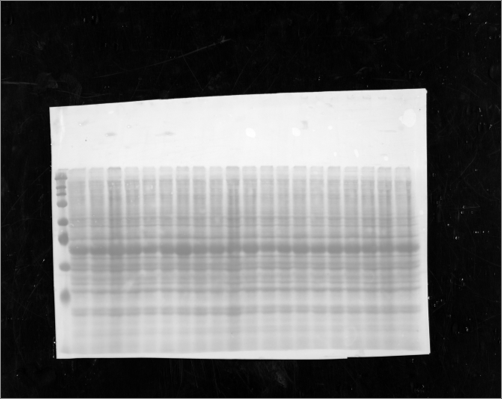

Supplement: Supplementary file 17 — Figures Appendix Source Data [file 44321_2025_254_MOESM17_ESM.zip › FigS11D/Ponceau-forRARy.tif]

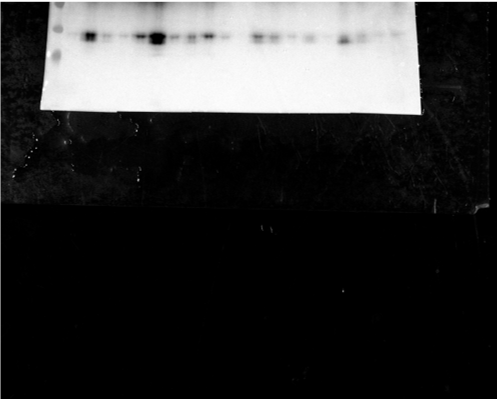

Supplement: Supplementary file 17 — Figures Appendix Source Data [file 44321_2025_254_MOESM17_ESM.zip › FigS11D/RARb.tif]

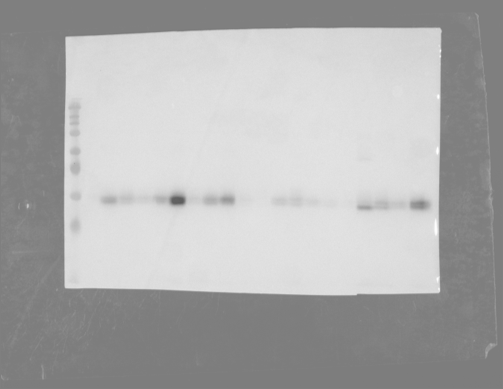

Supplement: Supplementary file 17 — Figures Appendix Source Data [file 44321_2025_254_MOESM17_ESM.zip › FigS11D/RARy.tif]

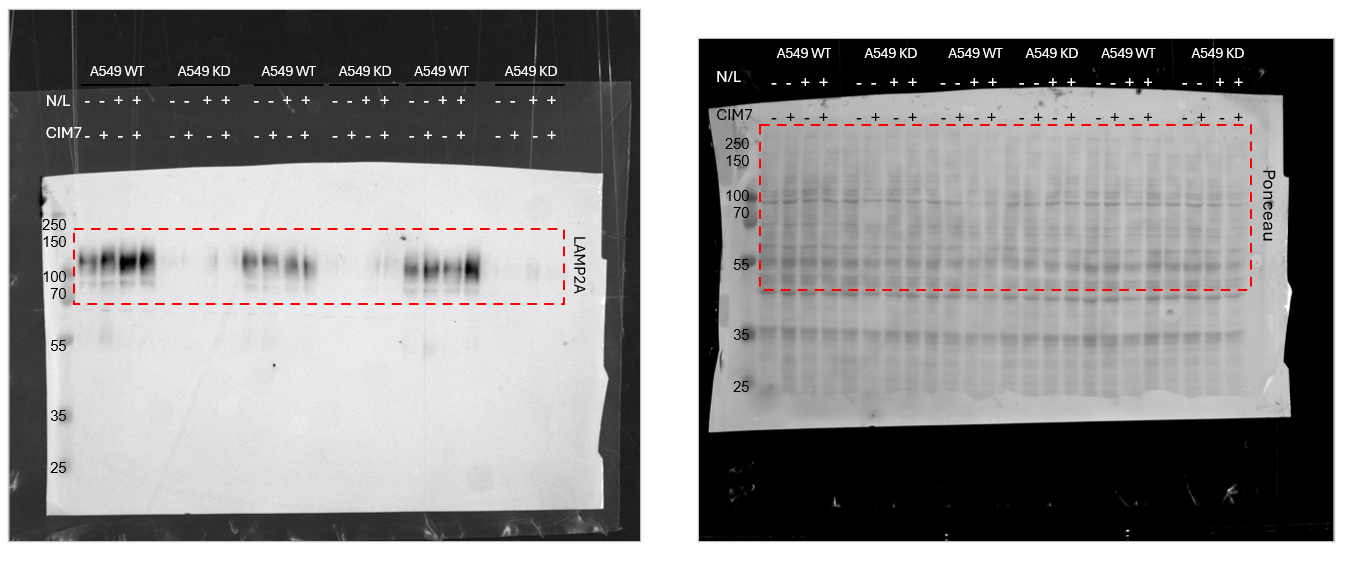

Supplement: Supplementary file 17 — Figures Appendix Source Data [file 44321_2025_254_MOESM17_ESM.zip › FigS7A/Annotated-Blots.png]

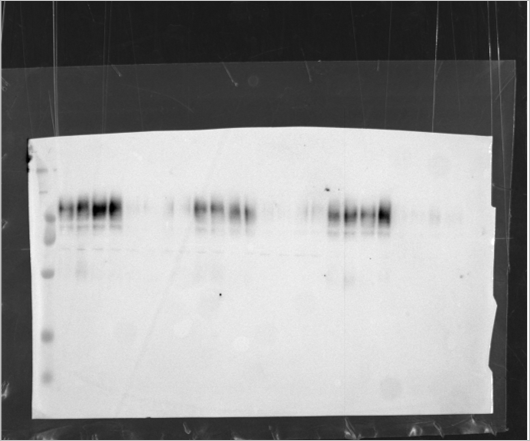

Supplement: Supplementary file 17 — Figures Appendix Source Data [file 44321_2025_254_MOESM17_ESM.zip › FigS7A/LAMP2A.tif]

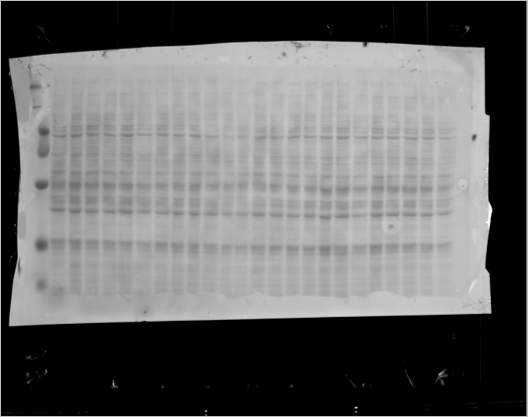

Supplement: Supplementary file 17 — Figures Appendix Source Data [file 44321_2025_254_MOESM17_ESM.zip › FigS7A/Ponceau.tif]

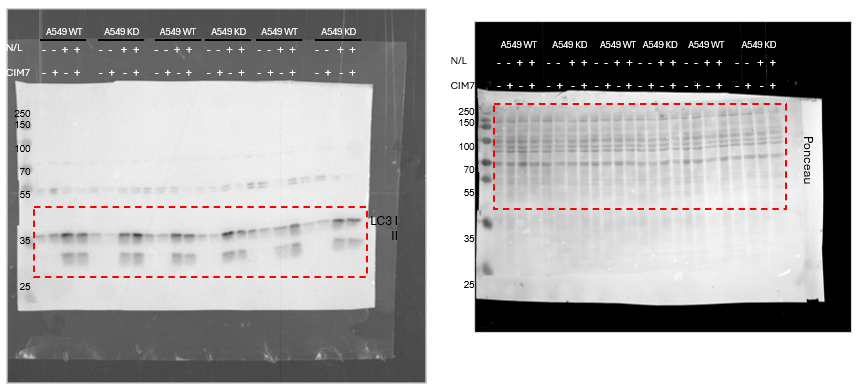

Supplement: Supplementary file 17 — Figures Appendix Source Data [file 44321_2025_254_MOESM17_ESM.zip › FigS7B/Annotated-Blots.png]

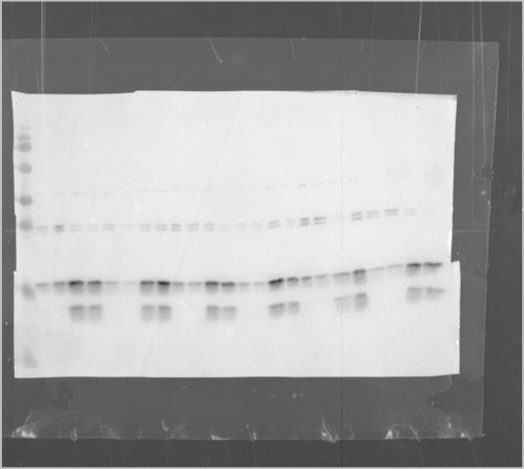

Supplement: Supplementary file 17 — Figures Appendix Source Data [file 44321_2025_254_MOESM17_ESM.zip › FigS7B/LC3.tif]

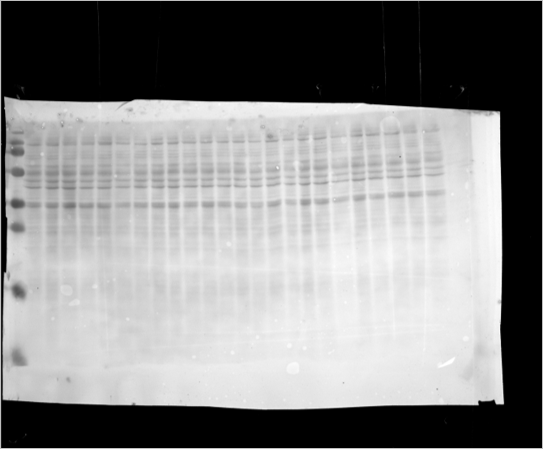

Supplement: Supplementary file 17 — Figures Appendix Source Data [file 44321_2025_254_MOESM17_ESM.zip › FigS7B/Ponceau.tif]

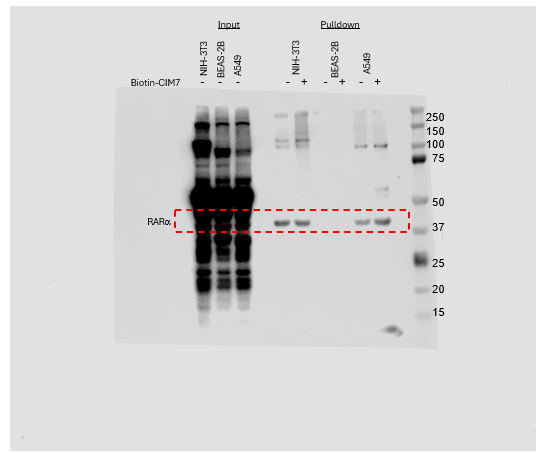

Supplement: Supplementary file 17 — Figures Appendix Source Data [file 44321_2025_254_MOESM17_ESM.zip › FigS9D/Annotated-Blot.png]

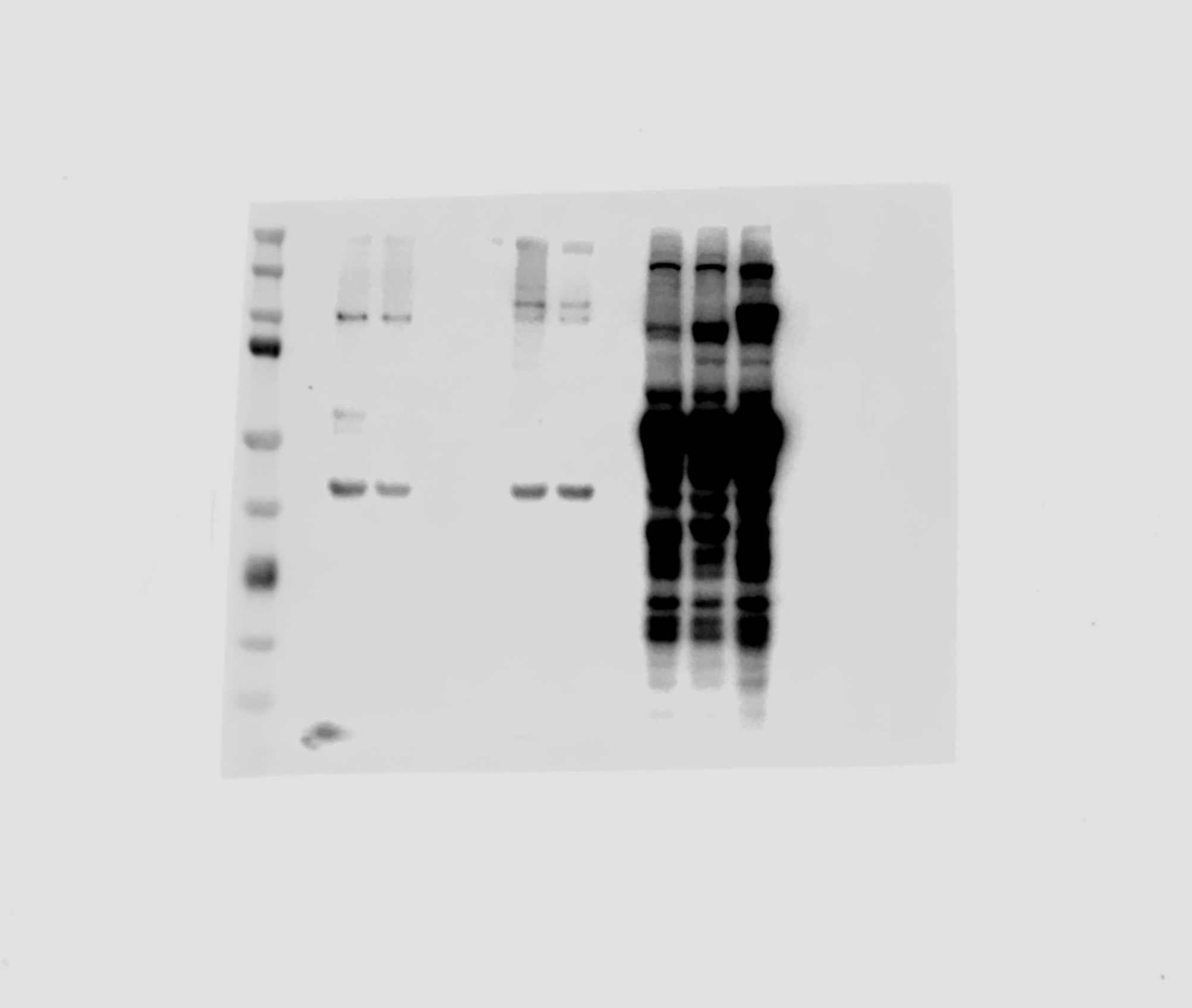

Supplement: Supplementary file 17 — Figures Appendix Source Data [file 44321_2025_254_MOESM17_ESM.zip › FigS9D/RARa.tif]
