# Supplementary material for: Small molecule disruption of RARα/NCoR1 interaction inhibits chaperone-mediated autophagy in cancer
Source: EMBO Mol Med. 2025 Jun 9;17(7):1716–55. doi: 10.1038/s44321-025-00254-y (PMC12254369; doi:10.1038/s44321-025-00254-y)
Supplement: Supplementary file 17 — Figures Appendix Source Data [file 44321_2025_254_MOESM17_ESM.zip › AppendixFigS6DE-S9B-Report.pdf]

FINAL REPORT

eurofins DISCOVERY

In Vitro Pharmacology and ADME-Tox - Study of CIM7

STUDY ID: FR095-0048774

STUDY NUMBER
100075307

January 17, 2025

CONFIDENTIAL

1. STUDY REFERENCES

**Study title**	**In Vitro Pharmacology and ADME-Tox - Study of CIM7**	
**Study number**	**100075307**	**FINAL REPORT**<br>**January 17, 2025**
**Study ID**	**FR095-0048774**	
**Experimental period**	December 24, 2024 - January 16, 2025	
**PO number**	P1030101	

2. PERSONS INVOLVED IN THE STUDY

**Technical contact**	**Eurofins Cerep**<br>2, rue du Professeur GARGOUÏL<br>B.P. 30001<br>86 600 Celle l'Evescault<br>France<br>Tel: +33 (0)5 49 89 30 00<br>Fax: +33 (0)5 49 43 21 70	**Thierry JOLAS, Ph.D.**<br>Principal Scientist, Pharmacology<br>scientificsupportpoitiers@discovery.eurofinseu.com
**Study sponsor**	**Albert Einstein College of Medicine**<br>Biochemistry & Medicine<br>1300 Morris Park Ave<br>New York, NY 10461<br>U.S.A.	**Dr. Evripidis GAVATHIOTIS**<br>Assistant Professor of Biochemistry and Medicine

3. APPROVAL

Head of laboratory statement

This study was conducted according to the procedures described in this report.

Eurofins Cerep
2, rue du Professeur GARGOUÏL
B.P. 30001
86 600 Celle l'Evescault
France

Sophie SEIGNEURIN, Mrs
Operations Director
Sophie.Seigneurin@discovery.eurofinseu.com

Signature

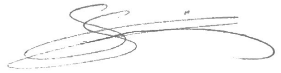

Quality assurance statement

This study was inspected by Eurofins Cerep Quality Control Unit, the results and methods presented in this report accurately reflect the methods used and the data collected for this study.

Eurofins Cerep
2, rue du Professeur GARGOUÏL
B.P. 30001
86 600 Celle l'Evescault
France

Eric BOUCHET
Quality Site Group Leader
Eric.Bouchet@discovery.eurofinseu.com

Signature

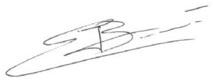

4. TABLE OF CONTENTS

1. STUDY REFERENCES 2
2. PERSONS INVOLVED IN THE STUDY 2
3. APPROVAL 3
4. TABLE OF CONTENTS 4
5. SUMMARY 5
  5.1. Study Design 5
  5.2. Measurements 5
  5.3. Results 5
6. COMPOUNDS 6
  6.1. Test Compounds 6
  6.2. Reference Compounds 6
7. RESULTS 7
  7.1. *In Vitro* Pharmacology: Binding Assays 7
    7.1.1. Test Compound Results 7
    7.1.2. Reference Compound Results 9
  7.2. *In Vitro* Pharmacology: Enzyme and Uptake Assays 11
    7.2.1. Test Compound Results 11
    7.2.2. Reference Compound Results 11
  7.3. ADME-Tox: Solution Properties 12
    7.3.1. Test Compound Results 12
    7.3.2. Reference Compound Results 12
8. RESULTS INTERPRETATION GUIDE 13
9. MATERIALS AND METHODS 14
  9.1. Experimental Conditions 14
    9.1.1. *In Vitro* Pharmacology: Binding Assays 14
    9.1.2. *In Vitro* Pharmacology: Enzyme and Uptake Assays 17
    9.1.3. ADME-Tox: Solution Properties 18
  9.2. Analysis and expression of results 19
    9.2.1. *In Vitro* Pharmacology: Binding Assays 19
    9.2.2. *In Vitro* Pharmacology: Enzyme and Uptake Assays 20
    9.2.3. ADME-Tox: Solution Properties 20
10. BIBLIOGRAPHY 21

5. SUMMARY

The purpose of this study was to test CIM7 in Binding, enzyme and uptake and Solution Properties assays.

5.1. Study Design

CIM7 was tested at 1.0E-05 M.

5.2. Measurements

Compound binding was calculated as a % inhibition of the binding of a ligand specific for each target.
Compound enzyme inhibition effect was calculated as a % inhibition of control enzyme activity.

5.3. Results

Results showing an inhibition or stimulation higher than 50% are considered to represent significant effects of the test compounds. Such effects were observed here and are listed in the following tables.

Assay	1.0E-05 M
Sodium Channel Site2 (Non-selective) Rat Ion Channel Batrachotoxin Mass Spectrometry Binding	86.4%
5-HT_{2A}*(h)* (agonist radioligand)	69.3%
5-HT_{2B}*(h)* (agonist radioligand)	66.8%
Ca^{2+} channel (L dihydropyridine site) (antagonist radioligand)	87.3%
norepinephrine transporter*(h)* (antagonist radioligand)	52.2%
dopamine transporter*(h)* (antagonist radioligand)	98%

6. COMPOUNDS

6.1. Test Compounds

Client Compound ID	Compound ID	Reference Number	Batch Number	FW	MW	Purity	Received Form	Stock solution	Flag
CIM7	100075307-1	-	-	455.43	455.43	100.0	Liquid	1.E-02 M DMSO	-

FW: Formula Weight - MW: Molecular Weight

6.2. Reference Compounds

In each experiment and if applicable, the respective reference compound was tested concurrently with CIM7, and the data were compared with historical values determined at Eurofins. The experiment was accepted in accordance with Eurofins validation Standard Operating Procedure.

7. RESULTS

7.1. *In Vitro* Pharmacology: Binding Assays

7.1.1. Test Compound Results

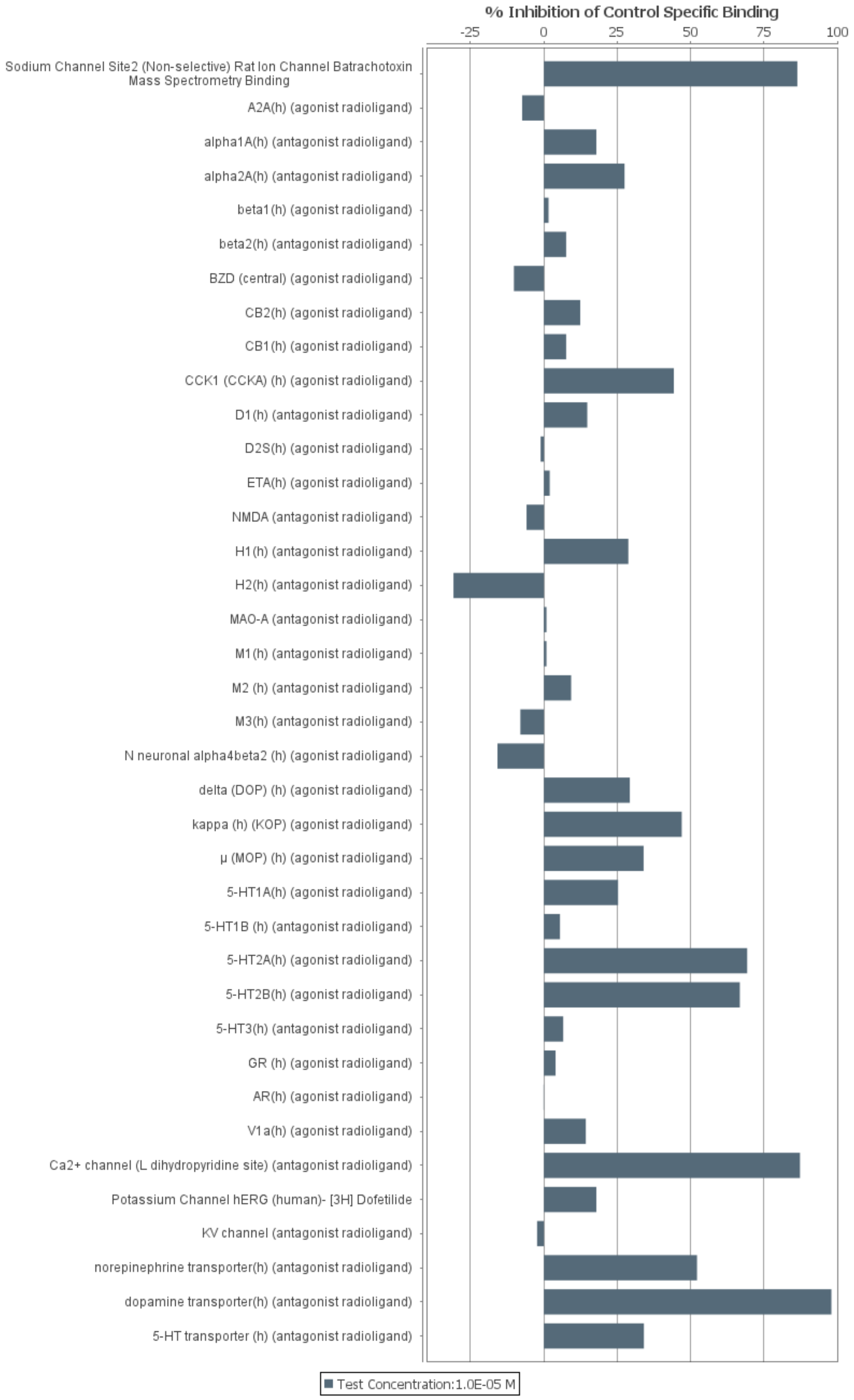

Figure 1. Histogram for CIM7

Compound I.D.	Client Compound I.D.	Test Concentration	% Inhibition of Control Specific Binding 1st	2nd	Mean	Flags 1st	2nd
**Sodium Channel Site2 (Non-selective) Rat Ion Channel Batrachotoxin Mass Spectrometry Binding**							
100075307-1	CIM7	1.0E-05 M	85.8	86.9	**86.4**		
**A_{2A}*(h)* (agonist radioligand)**							
100075307-1	CIM7	1.0E-05 M	-5.8	-9.1	-7.4		
**alpha$_{1A}$*(h)* (antagonist radioligand)**							
100075307-1	CIM7	1.0E-05 M	12.3	23.5	17.9		
**alpha$_{2A}$*(h)* (antagonist radioligand)**							
100075307-1	CIM7	1.0E-05 M	30.5	24.6	27.5		
**beta$_1$*(h)* (agonist radioligand)**							
100075307-1	CIM7	1.0E-05 M	0.6	2.6	1.6		
**beta$_2$*(h)* (antagonist radioligand)**							
100075307-1	CIM7	1.0E-05 M	9.0	6.2	7.6		
**BZD (central) (agonist radioligand)**							
100075307-1	CIM7	1.0E-05 M	-10.1	-10.3	-10.2		
**CB_2*(h)* (agonist radioligand)**							
100075307-1	CIM7	1.0E-05 M	4.8	20.0	12.4		
**CB_1*(h)* (agonist radioligand)**							
100075307-1	CIM7	1.0E-05 M	4.3	10.9	7.6		
**CCK_1 (CCK_A) *(h)* (agonist radioligand)**							
100075307-1	CIM7	1.0E-05 M	42.7	45.9	44.3		
**D_1*(h)* (antagonist radioligand)**							
100075307-1	CIM7	1.0E-05 M	14.3	15.2	14.8		
**D_{2S}*(h)* (agonist radioligand)**							
100075307-1	CIM7	1.0E-05 M	23.8	-26.0	-1.1		
**ET_A*(h)* (agonist radioligand)**							
100075307-1	CIM7	1.0E-05 M	-6.0	10.0	2.0		
**NMDA (antagonist radioligand)**							
100075307-1	CIM7	1.0E-05 M	0.5	-12.4	-5.9		
**H_1*(h)* (antagonist radioligand)**							
100075307-1	CIM7	1.0E-05 M	30.8	26.9	28.8		
**H_2*(h)* (antagonist radioligand)**							
100075307-1	CIM7	1.0E-05 M	-40.5	-21.2	-30.8		
**MAO-A (antagonist radioligand)**							
100075307-1	CIM7	1.0E-05 M	-0.3	2.1	0.9		
**M_1*(h)* (antagonist radioligand)**							
100075307-1	CIM7	1.0E-05 M	-3.6	5.5	0.9		
**M_2 *(h)* (antagonist radioligand)**							
100075307-1	CIM7	1.0E-05 M	8.0	10.5	9.3		
**M_3*(h)* (antagonist radioligand)**							
100075307-1	CIM7	1.0E-05 M	-8.3	-7.8	-8.0		
**N neuronal alpha4beta2 *(h)* (agonist radioligand)**							
100075307-1	CIM7	1.0E-05 M	-19.0	-12.5	-15.8		
**delta (DOP) *(h)* (agonist radioligand)**							
100075307-1	CIM7	1.0E-05 M	23.4	35.2	29.3		
**kappa *(h)* (KOP) (agonist radioligand)**							
100075307-1	CIM7	1.0E-05 M	42.1	51.9	47.0		
**μ (MOP) *(h)* (agonist radioligand)**							
100075307-1	CIM7	1.0E-05 M	30.1	37.9	34.0		
**5-HT_{1A}*(h)* (agonist radioligand)**							
100075307-1	CIM7	1.0E-05 M	14.1	36.4	25.3		
**5-HT_{1B} *(h)* (antagonist radioligand)**							
100075307-1	CIM7	1.0E-05 M	11.3	-0.3	5.5		
**5-HT_{2A}*(h)* (agonist radioligand)**							
100075307-1	CIM7	1.0E-05 M	68.9	69.8	**69.3**		
**5-HT_{2B}*(h)* (agonist radioligand)**							
100075307-1	CIM7	1.0E-05 M	66.3	67.4	**66.8**		

Compound I.D.	Client Compound I.D.	Test Concentration	% Inhibition of Control Specific Binding			Flags	
			1st	2nd	Mean	1st	2nd
**5-HT$_3$*(h)* (antagonist radioligand)**							
100075307-1	CIM7	1.0E-05 M	6.3	7.0	6.6		
**GR *(h)* (agonist radioligand)**							
100075307-1	CIM7	1.0E-05 M	5.6	2.3	4.0		
**AR(h) (agonist radioligand)**							
100075307-1	CIM7	1.0E-05 M	1.1	-1.3	-0.1		
**V$_{1a}$*(h)* (agonist radioligand)**							
100075307-1	CIM7	1.0E-05 M	16.2	12.5	14.3		
**Ca^{2+} channel (L dihydropyridine site) (antagonist radioligand)**							
100075307-1	CIM7	1.0E-05 M	87.9	86.6	**87.3**		
**Potassium Channel hERG (human)- [3H] Dofetilide**							
100075307-1	CIM7	1.0E-05 M	19.9	16.0	17.9		
**K$_V$ channel (antagonist radioligand)**							
100075307-1	CIM7	1.0E-05 M	-3.5	-1.2	-2.3		
**norepinephrine transporter*(h)* (antagonist radioligand)**							
100075307-1	CIM7	1.0E-05 M	52.1	52.3	**52.2**		
**dopamine transporter*(h)* (antagonist radioligand)**							
100075307-1	CIM7	1.0E-05 M	97.6	98.3	**98.0**		
**5-HT transporter *(h)* (antagonist radioligand)**							
100075307-1	CIM7	1.0E-05 M	38.5	29.8	34.1		

7.1.2. Reference Compound Results

Compound I.D.	IC$_{50}$(M)	K$_i$(M)	nH
**Sodium Channel Site2 (Non-selective) Rat Ion Channel Batrachotoxin Mass Spectrometry Binding**			
Veratridine	1.5E-05 M	5.4E-06 M	0.7
**A$_{2A}$*(h)* (agonist radioligand)**			
NECA	4.2E-08 M	3.5E-08 M	0.7
**alpha$_{1A}$*(h)* (antagonist radioligand)**			
WB 4101	5.1E-10 M	2.6E-10 M	1.9
**alpha$_{2A}$*(h)* (antagonist radioligand)**			
yohimbine	7.3E-09 M	3.2E-09 M	1.0
**beta$_1$*(h)* (agonist radioligand)**			
atenolol	2.3E-07 M	1.3E-07 M	1.1
**beta$_2$*(h)* (antagonist radioligand)**			
ICI 118551	1.2E-09 M	3.9E-10 M	1.2
**BZD (central) (agonist radioligand)**			
diazepam	1.2E-08 M	1.0E-08 M	0.7
**CB$_2$*(h)* (agonist radioligand)**			
WIN 55212-2	4.5E-09 M	2.9E-09 M	1.2
**CB$_1$*(h)* (agonist radioligand)**			
CP 55940	2.1E-09 M	6.6E-10 M	0.9
**CCK$_1$ (CCK$_A$) *(h)* (agonist radioligand)**			
CCK-8s	1.0E-10 M	7.7E-11 M	1.1
**D$_1$*(h)* (antagonist radioligand)**			
SCH 23390	6.3E-10 M	2.5E-10 M	1.3
**D$_{2S}$*(h)* (agonist radioligand)**			
7-OH-DPAT	3.2E-09 M	1.3E-09 M	0.7
**ET$_A$*(h)* (agonist radioligand)**			
endothelin-1	7.1E-11 M	3.6E-11 M	1.0
**NMDA (antagonist radioligand)**			
CGS 19755	4.5E-07 M	3.7E-07 M	0.8
**H$_1$*(h)* (antagonist radioligand)**			
pyrilamine	2.8E-09 M	1.8E-09 M	1.0
**H$_2$*(h)* (antagonist radioligand)**			
cimetidine	5.3E-07 M	5.2E-07 M	2.2

Compound I.D.	IC_{50}(M)	K_i(M)	nH
**MAO-A (antagonist radioligand)**			
clorgyline	1.0E-09 M	6.0E-10 M	1.3
**M_1*(h)* (antagonist radioligand)**			
pirenzepine	3.9E-08 M	3.4E-08 M	1.1
**M_2 *(h)* (antagonist radioligand)**			
methoctramine	6.3E-08 M	4.4E-08 M	1.2
**M_3*(h)* (antagonist radioligand)**			
4-DAMP	1.4E-09 M	9.7E-10 M	1.3
**N neuronal alpha4beta2 *(h)* (agonist radioligand)**			
nicotine	6.9E-09 M	2.3E-09 M	1.1
**delta (DOP) *(h)* (agonist radioligand)**			
DPDPE	2.7E-09 M	1.5E-09 M	0.9
**kappa *(h)* (KOP) (agonist radioligand)**			
U50488	7.6E-10 M	4.1E-10 M	1.4
**μ (MOP) *(h)* (agonist radioligand)**			
DAMGO	1.0E-09 M	4.2E-10 M	>3
**5-HT_{1A}*(h)* (agonist radioligand)**			
8-OH-DPAT	8.7E-10 M	4.4E-10 M	0.9
**5-HT_{1B} *(h)* (antagonist radioligand)**			
Serotonine	3.1E-07 M	1.4E-07 M	1.0
**5-HT_{2A}*(h)* (agonist radioligand)**			
(±)DOI	3.7E-10 M	2.8E-10 M	1.3
**5-HT_{2B}*(h)* (agonist radioligand)**			
(±)DOI	3.5E-09 M	1.7E-09 M	1.1
**5-HT_3*(h)* (antagonist radioligand)**			
MDL 72222	8.7E-09 M	6.1E-09 M	1.1
**GR *(h)* (agonist radioligand)**			
dexamethasone	3.8E-09 M	1.9E-09 M	1.2
**AR(h) (agonist radioligand)**			
testosterone	5.2E-09 M	2.0E-09 M	1.1
**V_{1a}*(h)* (agonist radioligand)**			
[d(CH_2)$_5^1$,Tyr(Me)$_2$]-AVP	9.9E-10 M	6.2E-10 M	1.1
**Ca^{2+} channel (L dihydropyridine site) (antagonist radioligand)**			
nitrendipine	4.6E-10 M	2.4E-10 M	1.0
**Potassium Channel hERG (human)- [3H] Dofetilide**			
Terfenadine	1.6E-07 M	1.1E-07 M	1.2
**K_V channel (antagonist radioligand)**			
α-dendrotoxin	1.8E-10 M	1.4E-10 M	1.1
**norepinephrine transporter*(h)* (antagonist radioligand)**			
protriptyline	5.0E-09 M	3.7E-09 M	1.3
**dopamine transporter*(h)* (antagonist radioligand)**			
BTCP	1.7E-08 M	9.0E-09 M	1.1
**5-HT transporter *(h)* (antagonist radioligand)**			
imipramine	3.9E-09 M	1.8E-09 M	0.8

7.2. *In Vitro* Pharmacology: Enzyme and Uptake Assays

7.2.1. Test Compound Results

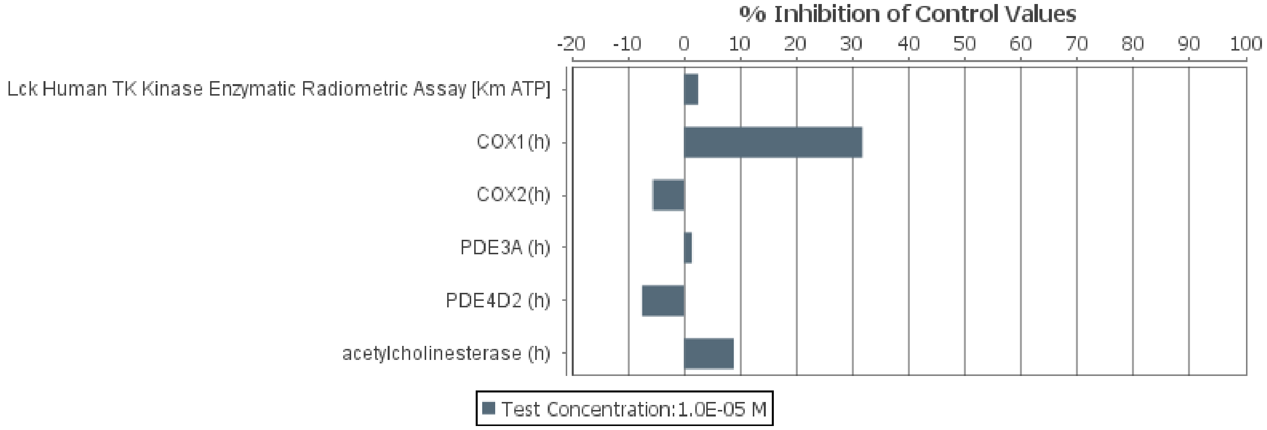

Figure 2. Histogram for CIM7

Compound I.D.	Client Compound I.D.	Test Concentration	% Inhibition of Control Values			Flags	
			1st	2nd	Mean	1st	2nd
**Lck Human TK Kinase Enzymatic Radiometric Assay [Km ATP]**							
100075307-1	CIM7	1.0E-05 M	6.7	-1.6	2.5		
**COX1(h)**							
100075307-1	CIM7	1.0E-05 M	31.5	31.9	31.7		
**COX2(h)**							
100075307-1	CIM7	1.0E-05 M	-6.2	-5.0	-5.6		
**PDE3A *(h)***							
100075307-1	CIM7	1.0E-05 M	-2.5	5.4	1.4		
**PDE4D2 *(h)***							
100075307-1	CIM7	1.0E-05 M	-7.6	-7.4	-7.5		
**acetylcholinesterase *(h)***							
100075307-1	CIM7	1.0E-05 M	10.4	7.2	8.8		

7.2.2. Reference Compound Results

Compound I.D.	IC_{50}(M)	nH
**Lck Human TK Kinase Enzymatic Radiometric Assay [Km ATP]**		
Staurosporine	1.7E-09 M	1.1
**COX1(h)**		
Diclofenac	1.0E-08 M	1.5
**COX2(h)**		
NS398	2.1E-07 M	1.0
**PDE3A *(h)***		
milrinone	4.9E-07 M	0.9
**PDE4D2 *(h)***		
Ro 20-1724	3.9E-07 M	0.8
**acetylcholinesterase *(h)***		
galanthamine	7.3E-07 M	1.2

7.3. ADME-Tox: Solution Properties

7.3.1. Test Compound Results

Compound I.D.	Client Compound I.D.	Test Concentration	% Protein Bound			% Recovery			Flags
			1st	2nd	Mean	1st	2nd	Mean	
**Protein binding (plasma, mouse, CD-1)**									
100075307-1	CIM7	1.0E-05 M	21.50	-206.24	-92.37	0.97	1.54	1.26	BLQ

BLQ: Below the Limit of Quantitation. Test compound was well detected in donor samples but not detected in receiver samples. The concentration of test compound in receiver sample was below the limit of quantitation.

7.3.2. Reference Compound Results

Compound I.D.	Test Concentration	% Protein Bound			% Recovery			Flags
		1st	2nd	Mean	1st	2nd	Mean	
**Protein binding (plasma, mouse, CD-1)**								
Acebutolol	1.0E-05 M	0.80	10.49	5.64	71.08	77.50	74.29	
Quinidine	1.0E-05 M	73.04	77.01	75.03	113.96	124.13	119.05	
Sertraline	1.0E-05 M	98.17	98.18	98.17	104.78	113.39	109.09	
Warfarin	1.0E-05 M	87.55	88.00	87.78	107.58	107.62	107.60	

8. RESULTS INTERPRETATION GUIDE

In Vitro Pharmacology

Results showing an inhibition (or stimulation for assays run in basal conditions) higher than 50% are considered to represent significant effects of the test compounds. 50% is the most common cut-off value for further investigation (determination of IC_{50} or EC_{50} values from concentration-response curves) that we would recommend.
Results showing an inhibition (or stimulation) between 25% and 50% are indicative of weak to moderate effects (in most assays, they should be confirmed by further testing as they are within a range where more inter-experimental variability can occur).
Results showing an inhibition (or stimulation) lower than 25% are not considered significant and mostly attributable to variability of the signal around the control level.
Low to moderate negative values have no real meaning and are attributable to variability of the signal around the control level. High negative values (≥ 50%) that are sometimes obtained with high concentrations of test compounds are generally attributable to non-specific effects of the test compounds in the assays. On rare occasion they could suggest an allosteric effect of the test compound.

9. MATERIALS AND METHODS

9.1. Experimental Conditions

Minor variations to the experimental protocol described below may have occurred during the testing, they have no impact on the quality of the results obtained.

9.1.1. *In Vitro* Pharmacology: Binding Assays

Assay	Source	Ligand	Conc.	Kd	Non Specific	Incubation	Detection Method	Bibl.
**Receptors**								
**A_{2A} *(h)* (agonist radioligand)**	human recombinant (HEK-293 cells)	[^{3}H]CGS 21680	6 nM	27 nM	NECA (10 µM)	120 min RT	Scintillation counting	141
**alpha$_{1A}$ *(h)* (antagonist radioligand)**	human recombinant (CHO cells)	[^{3}H]prazosin	0.1 nM	0.1 nM	epinephrine (0.1 mM)	60 min RT	Scintillation counting	897
**alpha$_{2A}$ *(h)* (antagonist radioligand)**	human recombinant (CHO cells)	[^{3}H]RX 821002	1 nM	0.8 nM	(-)epinephrine (100 µM)	60 min RT	Scintillation counting	542
**beta$_1$ *(h)* (agonist radioligand)**	human recombinant (HEK-293 cells)	[^{3}H](-)CGP 12177	0.3 nM	0.39 nM	alprenolol (50 µM)	60 min RT	Scintillation counting	548
**beta$_2$ *(h)* (antagonist radioligand)**	human recombinant (CHO cells)	[^{3}H](-)CGP 12177	0.3 nM	0.15 nM	alprenolol (50 µM)	120 min RT	Scintillation counting	794
**CB_2 *(h)* (agonist radioligand)**	human recombinant (CHO cells)	[^{3}H]WIN 55212-2	0.8 nM	1.5 nM	WIN 55212-2 (5 µM)	120 min 37°C	Scintillation counting	165
**CB_1 *(h)* (agonist radioligand)**	human recombinant (Chem-RBL cells)	[^{3}H]CP 55940	2 nM	0.9 nM	AM281 (10 µM)	30 min 22°C	Scintillation counting	657
**CCK_1 (CCK_A) *(h)* (agonist radioligand)**	human recombinant (CHO cells)	[^{125}I]CCK-8s	0.08 nM	0.24 nM	CCK-8s (1 µM)	60 min RT	Scintillation counting	562
**D_1 *(h)* (antagonist radioligand)**	human recombinant (CHO cells)	[^{3}H]SCH 23390	0.3 nM	0.2 nM	SCH 23390 (1 µM)	60 min RT	Scintillation counting	281
**D_{2S} *(h)* (agonist radioligand)**	human recombinant (HEK-293 cells)	[^{3}H]7-OH-DPAT	1 nM	0.68 nM	butaclamol (10 µM)	60 min RT	Scintillation counting	87
**ET_A *(h)* (agonist radioligand)**	human recombinant (CHO cells)	[^{125}I]endothelin-1	0.03 nM	0.03 nM	endothelin-1 (100 nM)	120 min 37°C	Scintillation counting	30
**H_1 *(h)* (antagonist radioligand)**	human recombinant (HEK-293 cells)	[^{3}H]pyrilamine	1 nM	1.7 nM	pyrilamine (1 µM)	60 min RT	Scintillation counting	492
**H_2 *(h)* (antagonist radioligand)**	human recombinant (CHO cells)	[^{125}I]APT	0.075 nM	2.9 nM	tiotidine (100 µM)	120 min RT	Scintillation counting	540
**M_1 *(h)* (antagonist radioligand)**	human recombinant (CHO cells)	[^{3}H]pirenzepine	2 nM	13 nM	atropine (1 µM)	60 min RT	Scintillation counting	59
**M_2 *(h)* (antagonist radioligand)**	human recombinant (CHO cells)	[^{3}H]AF-DX 384	2 nM	4.6 nM	atropine (1 µM)	60 min RT	Scintillation counting	59
**M_3 *(h)* (antagonist radioligand)**	human recombinant (CHO cells)	[^{3}H]4-DAMP	0.2 nM	0.5 nM	atropine (1 µM)	60 min RT	Scintillation counting	546
**N neuronal alpha4beta2 *(h)* (agonist radioligand)**	human recombinant (SH-SY5Y cells)	[^{3}H]cytisine	0.6 nM	0.3 nM	nicotine (10 µM)	120 min 4°C	Scintillation counting	1084
**delta (DOP) *(h)* (agonist radioligand)**	human recombinant (Chem-1 (RBL) cells)	[^{3}H]DADLE	0.5 nM	0.6 nM	naltrexone (10 µM)	60 min RT	Scintillation counting	501

Assay	Source	Ligand	Conc.	Kd	Non Specific	Incubation	Detection Method	Bibl.
**kappa *(h)* (KOP) (agonist radioligand)**	human recombinant (RBL cells)	[^{3}H]U69593	0.5 nM	0.6 nM	naloxone (10µM)	60 min RT	Scintillation counting	222
**µ (MOP) *(h)* (agonist radioligand)**	human recombinant (HEK-293 cells)	[^{3}H]DAMGO	0.5 nM	0.35 nM	naloxone (10 µM)	120 min RT	Scintillation counting	260
**5-HT$_{1A}$ *(h)* (agonist radioligand)**	human recombinant (HEK-293 cells)	[^{3}H]8-OH-DPAT	0.5 nM	0.5 nM	8-OH-DPAT (10 µM)	60 min RT	Scintillation counting	164
**5-HT$_{1B}$ *(h)* (antagonist radioligand)**	human recombinant (Chem-1 (RBL) cells)	[^{3}H]GR125743	1 nM	0.8 nM	Serotonine (30 µM)	60 min 37°C	Scintillation counting	1451
**5-HT$_{2A}$ *(h)* (agonist radioligand)**	human recombinant (HEK-293 cells)	[^{125}I](±)DOI	0.1 nM	0.3 nM	(±)DOI (1 µM)	60 min RT	Scintillation counting	288
**5-HT$_{2B}$ *(h)* (agonist radioligand)**	human recombinant (CHO cells)	[^{125}I](±)DOI	0.2 nM	0.2 nM	(±)DOI (1 µM)	60 min RT	Scintillation counting	571
**GR *(h)* (agonist radioligand)**	human endogenous (IM-9 cells)	[^{3}H]dexamethasone	1.5 nM	1.5 nM	triamcinolone (10 µM)	24 hr 4°C	Scintillation counting	283
**AR(h) (agonist radioligand)**	human endogenous (LNCaP cells)	[^{3}H]methyltrienolone	1 nM	0.6 nM	testostérone (1 µM)	4 hr 22°C	Scintillation counting	498
**V$_{1a}$ *(h)* (agonist radioligand)**	human recombinant (CHO cells)	[^{3}H]AVP	0.3 nM	0.5 nM	AVP (1 µM)	60 min RT	Scintillation counting	343
**Ion channels**								
**Sodium Channel Site2 (Non-selective) Rat Ion Channel Batrachotoxin Mass Spectrometry Binding**	rat brain	Batrachotoxin	15 nM	8.9 nM	Veratridine (1 mM)	60 minutes at 37°C	MS	28
**BZD (central) (agonist radioligand)**	rat cerebral cortex	[^{3}H]flunitrazepam	0.4 nM	2.1 nM	diazepam (3 µM)	60 min 4°C	Scintillation counting	227
**NMDA (antagonist radioligand)**	rat cerebral cortex	[^{3}H]CGP 39653	5 nM	23 nM	L-glutamate (100 µM)	60 min 4°C	Scintillation counting	221
**5-HT$_3$ *(h)* (antagonist radioligand)**	human recombinant (CHO cells)	[^{3}H]BRL 43694	0.5 nM	1.15 nM	MDL 72222 (10 µM)	120 min RT	Scintillation counting	109
**Ca^{2+} channel (L dihydropyridine site) (antagonist radioligand)**	rat cerebral cortex	[^{3}H]nitrendipine	0.25 nM	0.27 nM	nitrendipine (1 µM)	90 min RT	Scintillation counting	996
**Potassium Channel hERG (human)- [3H] Dofetilide**	human recombinant (HEK-293 cells)	[^{3}H]Dofetilide	3 nM	6.6 nM	Terfenadine (25 µM)	60 min RT	Scintillation counting	1398
**K$_V$ channel (antagonist radioligand)**	rat cerebral cortex	[^{125}I]α-dendrotoxin	0.01 nM	0.04 nM	α-dendrotoxin (50 nM)	60 min RT	Scintillation counting	225
**Transporters**								
**norepinephrine transporter *(h)* (antagonist radioligand)**	human recombinant (CHO cells)	[^{3}H]nisoxetine	1 nM	2.9 nM	desipramine (1 µM)	120 min 4°C	Scintillation counting	180
**dopamine transporter *(h)* (antagonist radioligand)**	human recombinant (CHO cells)	[^{3}H]BTCP	4 nM	4.5 nM	BTCP (10 µM)	120 min 4°C	Scintillation counting	190
**5-HT transporter *(h)* (antagonist radioligand)**	human recombinant (CHO cells)	[^{3}H]imipramine	2 nM	1.7 nM	imipramine (10 µM)	60 min RT	Scintillation counting	566

Assay	Source	Ligand	Conc.	Kd	Non Specific	Incubation	Detection Method	Bibl.
**Other enzymes**								
**MAO-A (antagonist radioligand)**	rat cerebral cortex	[^{3}H]Ro 41-1049	10 nM	14 nM	clorgyline (1 µM)	60 min 37°C	Scintillation counting	36

9.1.2. *In Vitro* Pharmacology: Enzyme and Uptake Assays

Assay	Source	Substrate/Stimulus/Tracer	Incubation	Measured Component	Detection Method	Bibl.
**Customized assays**						
**Lck Human TK Kinase Enzymatic Radiometric Assay [Km ATP]**	recombinant humain (cellules d'insectes)	33P	40 min RT	ATP + KVEKIGEGTYGVVYK Cdc2 peptide (250 µM)	Scintillation counting	1646, 1645
**Other enzymes**						
**COX1(h)**	human recombinant	Arachidonic acid (3µM) + ADHP (25 µM)	3 min RT	Resorufin (oxydized ADHP)	Fluorimetry	1480
**COX2(h)**	human recombinant (Sf9 cells)	arachidonic acid (1.2 µM)+ ADHP (25 µM)	5 min RT	Resorufin (oxydized ADHP)	Fluorimetry	1480
**PDE3A *(h)***	human recombinant (Sf21 cells)	[^{3}H]cAMP + cAMP (0.5µM)	15 min RT	[3H]5'AMP	Scintillation counting	1399
**PDE4D2 *(h)***	human recombinant (Sf9 cells)	[^{3}H]cAMP + cAMP (0.5µM)	20 min RT	[3H]5'AMP	Scintillation counting	1399
**acetylcholinesterase *(h)***	human recombinant (HEK-293 cells)	Acetylthiocholine (400 µM)	30 min RT	5 thio 2 nitrobenzoic acid	Photometry	63

9.1.3. ADME-Tox: Solution Properties

Assay	Technique	Incubation	Detection Method	Bibl.
**ADME**				
**Protein binding (plasma, mouse, CD-1)**	Equilibrium dialysis	4 hr<br>37°C	HPLC-MS/MS	640

9.2. Analysis and expression of results

9.2.1. *In Vitro* Pharmacology: Binding Assays

The results are expressed as a percent of control specific binding

$$\frac{\text{measured specific binding}}{\text{control specific binding}} * 100$$

and as a percent inhibition of control specific binding

$$100 - \left(\frac{\text{measured specific binding}}{\text{control specific binding}} * 100\right)$$

obtained in the presence of CIM7.

The IC_{50} values (concentration causing a half-maximal inhibition of control specific binding) and Hill coefficients (nH) were determined by non-linear regression analysis of the competition curves generated with mean replicate values using Hill equation curve fitting

$$Y = D + \left[\frac{A - D}{1 + (C/C_{50})^{nH}}\right]$$

where Y = specific binding, A = left asymptote of the curve, D = right asymptote of the curve, C = compound concentration, C_{50} = IC_{50}, and nH = slope factor. This analysis was performed using software developed at Cerep (Hill software) and validated by comparison with data generated by the commercial software SigmaPlot® 4.0 for Windows® (© 1997 by SPSS Inc.).
The inhibition constants (K_i) were calculated using the Cheng Prusoff equation

$$K_i = \frac{IC_{50}}{(1 + L/K_D)}$$

where L = concentration of ligand in the assay, and K_D = affinity of the ligand for the receptor.

9.2.2. *In Vitro* Pharmacology: Enzyme and Uptake Assays

The results are expressed as a percent of control specific activity

$$\frac{\text{measured specific activity}}{\text{control specific activity}} * 100$$

and as a percent inhibition of control specific activity

$$100 - \left(\frac{\text{measured specific activity}}{\text{control specific activity}} * 100\right)$$

obtained in the presence of CIM7.

The IC_{50} values (concentration causing a half-maximal inhibition of control specific activity), EC_{50} values (concentration producing a half-maximal increase in control basal activity), and Hill coefficients (nH) were determined by non-linear regression analysis of the inhibition/concentration-response curves generated with mean replicate values using Hill equation curve fitting

$$Y = D + \left[\frac{A - D}{1 + (C/C_{50})^{nH}}\right]$$

where Y = specific activity, A = left asymptote of the curve, D = right asymptote of the curve, C = compound concentration, C_{50} = IC_{50} or EC_{50} , and nH = slope factor.
This analysis was performed using software developed at Cerep (Hill software) and validated by comparison with data generated by the commercial software SigmaPlot® 4.0 for Windows® (© 1997 by SPSS Inc.).

9.2.3. ADME-Tox: Solution Properties

Protein Binding
The peak areas of the test compound in the buffer and test samples were used to calculate percent binding and recovery according to the following formulas:

$$\text{Protein binding(\%)} = \frac{Area_p - Area_b}{Area_p} * 100$$

$$\text{Recovery(\%)} = \frac{Area_p + Area_b}{Area_c} * 100$$

where
$Area_p$ = Peak area of analyte in protein matrix
$Area_b$ = Peak area of analyte in buffer
$Area_c$ = Peak area of analyte in control sample

10. BIBLIOGRAPHY

28. Brown, G.B. (1986), *J. Neurosci., 6: 2064-2070.*
30. Buchan, K.W. et al. (1994), *Brit. J. Pharmacol., 112: 1251-1257.*
36. Cesura, A.M. et al. (1990), *Mol. Pharmacol., 37: 358-366.*
59. Dorje, F. et al. (1991), *J. Pharmacol. Exp. Ther., 256: 727-733.*
63. Ellman, G.L. et al. (1961), *Biochem. Pharmacol., 7: 88-95.*
87. Grandy, D.K. et al. (1989), *Proc. Natl. Acad. Sci. U.S.A., 86: 9762-9766.*
109. Hope, A.G. et al. (1996), *Brit. J. Pharmacol., 118: 1237-1245.*
141. Luthin, D.R. et al. (1995), *Mol. Pharmacol., 47: 307-313.*
164. Mulheron, J.G. et al. (1994), *J. Biol. Chem., 269: 12954-12962.*
165. Munro, S. et al. (1993), *Nature, 365: 61-65.*
180. Pacholczyk, T. et al. (1991), *Nature, 350: 350-354.*
190. Pristupa, Z.B. et al. (1994), *Mol. Pharmacol., 45: 125-135.*
221. Sills, M.A. et al. (1991), *Eur. J. Pharmacol., 192: 19-24.*
222. Simonin, F. et al. (1995), *Proc. Natl. Acad. Sci. U.S.A., 92: 7006-7010.*
225. Sorensen, R.G. and Blaustein, M.P. (1989), *Mol. Pharmacol., 36 : 689-698.*
227. Speth, R.C. et al. (1979), *Life Sci., 24: 351-358.*
260. Wang, J.B. et al. (1994), *FEBS Lett., 338: 217-222.*
281. Zhou, Q.Y. et al. (1990), *Nature, 347: 76-80.*
283. Clark, A.F. et al. (1996), *Invest. Ophtalmol. Vis. Sci., 37: 805-813.*
288. Bryant, H.U. et al. (1996), *Life Sci., 15: 1259-1268.*
343. Tahara, A. et al. (1998), *Brit. J. Pharmacol., 125: 1463-1470.*
492. Smit, M.J. et al. (1996), *Brit. J. Pharmacol., 117: 1071-1080.*
498. Zava, D.T. et al. (1979), *Endocrinology, 104: 1007-1012.*
501. Simonin, F. et al. (1994), *Mol. Pharmacol., 46: 1015-1021.*
540. Leurs, R. et al. (1994), *Brit. J. Pharmacol., 112: 847-854.*
542. Langin, D. et al. (1989), *Eur. J. Pharmacol., 167: 95-104.*
546. Peralta, E. G. et al. (1987), *Embo. J., 6: 3923-3929.*
548. Levin, M.C. et al. (2002), *J. Biol.Chem., 277: 30429-30435.*
562. Bignon, E. et al. (1999), *J. Pharmacol. Exp. Ther. 289: 742-751.*
566. Tatsumi, M. et al. (1999), *Eur. J. Pharmacol., 368: 277-283.*
571. Choi, D.S. et al. (1994), *FEBS Lett., 352: 393-399.*
640. Banker, M.J. et al. (2003), *J. Pharm. Sci., 92: 967-974.*
657. Rinaldi-Carmona, M. et al. (1996), *J. Pharmacol. Exp. Ther., 278: 871-878.*
794. Joseph, S.S. et al. (2004), *Naun.-Sch. Arch. Pharm., 369: 525-532.*
897. Schwinn, D.A. et al. (1990), *J. Biol. Chem., 265: 8183-8189.*
996. Gould, R.J. et al. (1982), *Proc. Natl. Acad. Sci. U S A., 79: 3656-3660.*
1084. Gopalakrishnan, M. et al. (1996), *J. Pharmacol. Exp. Ther., 276: 289-297.*
1398. Huang XP1, Mangano T, Hufeisen S, Setola V, Roth BL., *Assay Drug Dev Technol. 2010 Dec;8(6):727-42*
1399. Maurice D.H. et al. (2014), *Nat Rev Drug Discov., 13: 290-314*
1451. Maier DL, Sobotka-Briner C, Ding M, Powell ME, Jiang Q, Hill G, Heys JR, Elmore CS, Pierson ME and Mrzljak L (2009), Maier DL et al. J Pharmacol Exp Ther. 330(1):342, 2009.
1480. Pattaraporn Vanachayangkul and William H.Tolleson (2012), *Hindawi Publishing Corporation, Enzyme Research, Volume 2012, Article ID 416062, 7*
1645. Davies SP, Reddy H, Caivano M and Cohen P, Biochem J. 351(Pt 1): 95, 2000.
1646. Gao Y, Davies SP, Augustin M, Woodward A, Patel UA, Kovelman R, Harvey KJ, Biochem J. 451, 313-28, 2013.
