# Supplementary material for: Small molecule disruption of RARα/NCoR1 interaction inhibits chaperone-mediated autophagy in cancer
Source: EMBO Mol Med. 2025 Jun 9;17(7):1716–55. doi: 10.1038/s44321-025-00254-y (PMC12254369; doi:10.1038/s44321-025-00254-y)
Supplement: Supplementary file 18 — Expanded View Figures [file 44321_2025_254_MOESM18_ESM.pdf]

Expanded View Figures

Figure EV1. Profiling of CMA in NSCLC.

(**A**) Table of our five NSCLC cells lines, their CMA activity, and status of oncogenes. (**B**) Representative immunoblot for LAMP2A (left) and quantification of LAMP2A levels (right) in the indicated NSCLC cell lines and non-tumorigenic control BEAS-2B cells normalized to levels of BEAS-2B cells in each experiment (right). $n = 5$–6 independent experiments. (****$P \le 0.0001$). (**C–F**) Transcriptional analysis of CMA-related genes (**C**), calculated CMA z-score (**D**), expression (as z-score) of CMA effectors, positive regulators, and negative regulators (**E**), and NCoR1/RARα ratio (**F**), in 17 non-tumorigenic lung cell lines and 15 NSCLC-derived cell lines. The heat map in (**C**) shows differential gene expression. Cell lines are listed in Appendix Table S1. Data from (Hruz et al, 2008). (**D**): **$P = 0.0031$; (**E**): ***$P = 0.0001$, ****$P \le 0.0001$, *$P = 0.0403$, ***$P = 0.0004$, ***$P = 0.0005$, *$P = 0.0108$, **$P = 0.0012$; (**F**): **$P = 0.0029$). Data information: All values are mean + SEM or individual data points to represent individual samples. One-way ANOVA (**B**), unpaired two-tailed *t* test (**D**, **F**), or multiple unpaired *t* tests with individual variances and Bonferroni post-hoc analysis (**E**) were performed. Source data are available online for this figure.

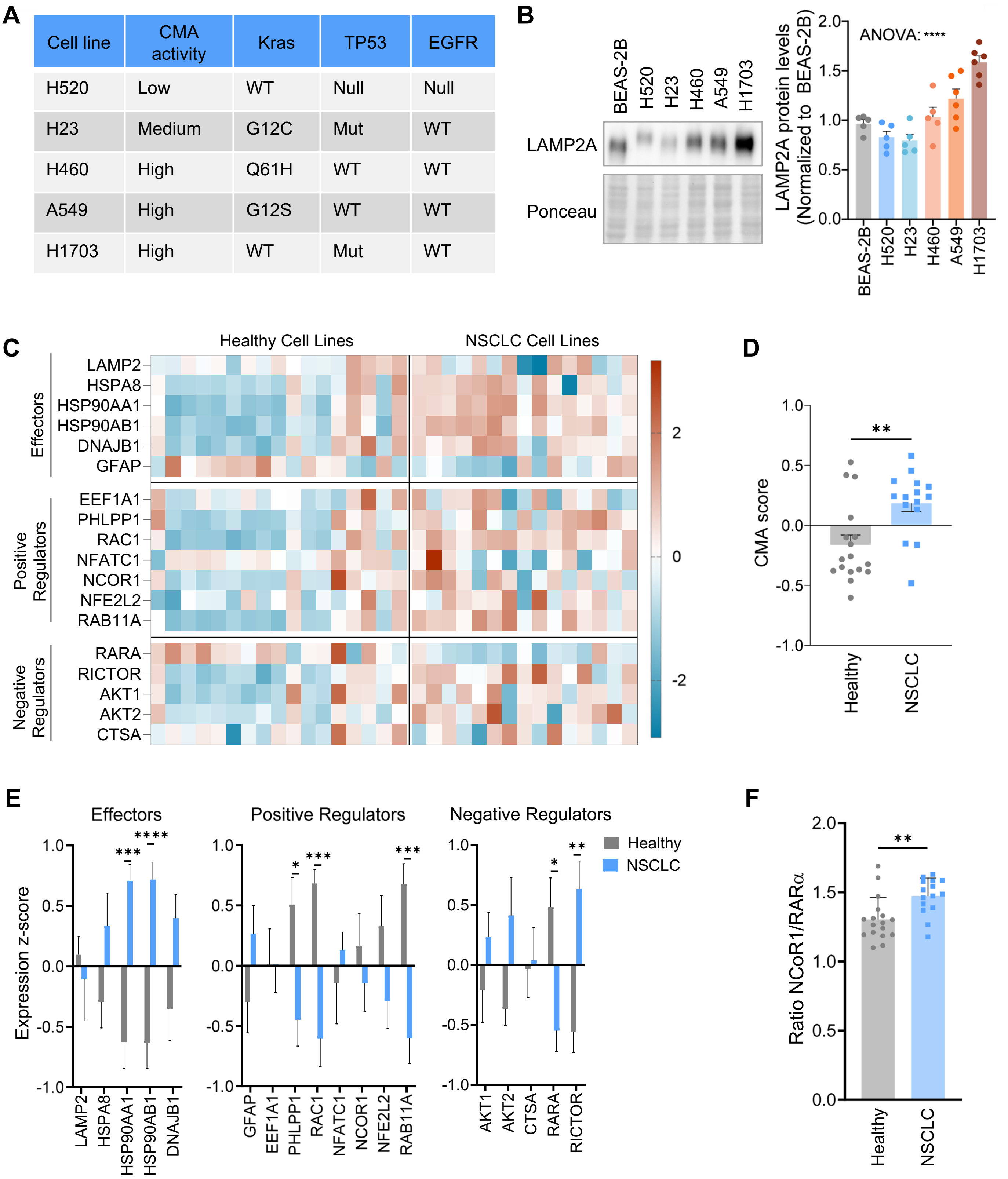
A
Cell line | CMA activity | Kras | TP53 | EGFR
H520 | Low | WT | Null | Null
H23 | Medium | G12C | Mut | WT
H460 | High | Q61H | WT | WT
A549 | High | G12S | WT | WT
H1703 | High | WT | Mut | WT
B
BEAS-2B
H520
H23
H460
A549
H1703
LAMP2A
Ponceau
LAMP2A protein levels (Normalized to BEAS-2B)
ANOVA: ****
2.0
1.5
1.0
0.5
0.0
C
Healthy Cell Lines
NSCLC Cell Lines
Effectors
LAMP2
HSPA8
HSP90AA1
HSP90AB1
DNAJB1
GFAP
Positive Regulators
EEF1A1
PHLPP1
RAC1
NFATC1
NCOR1
NFE2L2
RAB11A
Negative Regulators
RARA
RICTOR
AKT1
AKT2
CTSA
2
0
-2
D
CMA score
**
1.0
0.5
0.0
-0.5
-1.0
Healthy
NSCLC
E
Effectors
Positive Regulators
Negative Regulators
Expression z-score
Healthy
NSCLC
***
****
*
***
***
*
**
LAMP2
HSPA8
HSP90AA1
HSP90AB1
DNAJB1
GFAP
EEF1A1
PHLPP1
RAC1
NFATC1
NCOR1
NFE2L2
RAB11A
AKT1
AKT2
CTSA
RARA
RICTOR
F
Ratio NCoR1/RARα
**
2.0
1.5
1.0
0.5
0.0
Healthy
NSCLC

 © The Author(s)

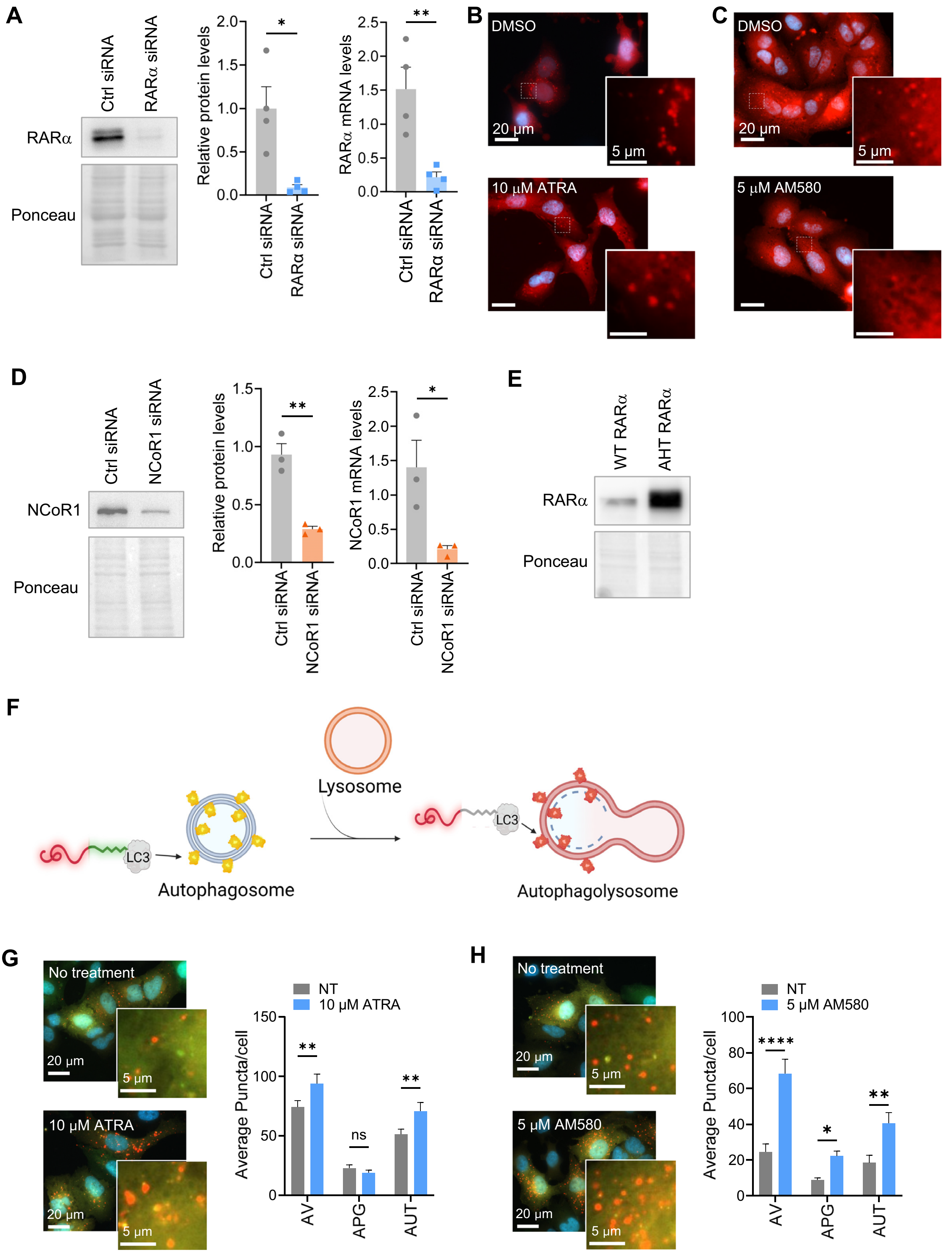
A
Ctrl siRNA
RARα siRNA
RARα
Ponceau
Relative protein levels
RARα mRNA levels
*
**
B
DMSO
10 μM ATRA
20 μm
5 μm
C
DMSO
5 μM AM580
D
Ctrl siRNA
NCoR1 siRNA
NCoR1
Ponceau
Relative protein levels
NCoR1 mRNA levels
E
WT RARα
AHT RARα
RARα
Ponceau
F
Lysosome
LC3
Autophagosome
Autophagolysosome
G
No treatment
10 μM ATRA
NT
10 μM ATRA
Average Puncta/cell
AV
APG
AUT
ns
H
No treatment
5 μM AM580
NT
5 μM AM580
Average Puncta/cell
****

Figure EV2. Validation of knockdowns and effect of RAR agonists on macroautophagy.

(**A**) RARα protein levels and RNA expression in A549 cells transfected with control (ctrl) or RARα siRNA. Representative immunoblot (left), protein level quantification (center), and RNA expression normalized to control siRNA (right) are shown. $n = 4$ independent experiments. (*$P = 0.0107$, **$P = 0.0081$). (**B**) Representative fluorescence images of A549 cells expressing KFERQ-PS-Dendra treated with 10 μM ATRA or equal volume DMSO in serum-free media for 24 h. Quantification is shown in Fig. 1E. (**C**) Representative fluorescence images of A549 cells expressing KFERQ-PS-Dendra treated with 5 μM AM580 or equal volume DMSO for 24 h. Quantification is shown in Fig. 1F. (**D**) NCoR1 protein levels and RNA expression in A549 cells transfected with control (ctrl) or NCoR1 siRNA. Representative immunoblot (left), protein level quantification (center), and RNA expression normalized to control siRNA (right) are shown. $n = 3$ independent experiments. (**$P = 0.0028$, *$P = 0.0400$).
(**E**) Representative immunoblot (left) of A549 cells expressing KFERQ-PS-Dendra transfected with equal amounts of DNA of WT RARα or AHT RARα. (**F**) Schematic of mCherry-GFP-LC3 reporter (Kimura et al, 2007). When the reporter is associated to autophagosomes, both mCherry and GFP fluoresce and autophagosomes are visualized as yellow puncta. Once the autophagosome fuses with a lysosome GFP fluorescence is quenched and autophagolysosomes appear as red fluorescent puncta. Created in BioRender. https://BioRender.com/oqvzi51. (**G**, **H**) Macroautophagy activity in A549 cells expressing mCherry-GFP-LC3 treated with 10 μM ATRA (**G**) or 5 μM AM580 (**H**) or equal volume DMSO (No treatment; NT) for 24 h. Representative images (left) and quantification of number of the indicated autophagic compartments (right). AV: autophagic vacuoles (total number of fluorescent red puncta), APG: autophagosomes (total number of green fluorescent puncta) and AUT: autolysosomes (total number of red – green fluorescent puncta). $n = 35–45$ cells from 2 independent experiments. (**G**: **$P = 0.0086$, ns = not significant, **$P = 0.0098$, **H**: ****$P \leq 0.0001$, *$P = 0.0448$, **$P = 0.0015$). Data Information: All values are mean + SEM. Unpaired two-tailed *t* test (**A**, **D**) and two-way ANOVA (**G**, **H**) were used. Insets show higher magnification and nuclei are highlighted with DAPI. Source data are available online for this figure.

© The Author(s)

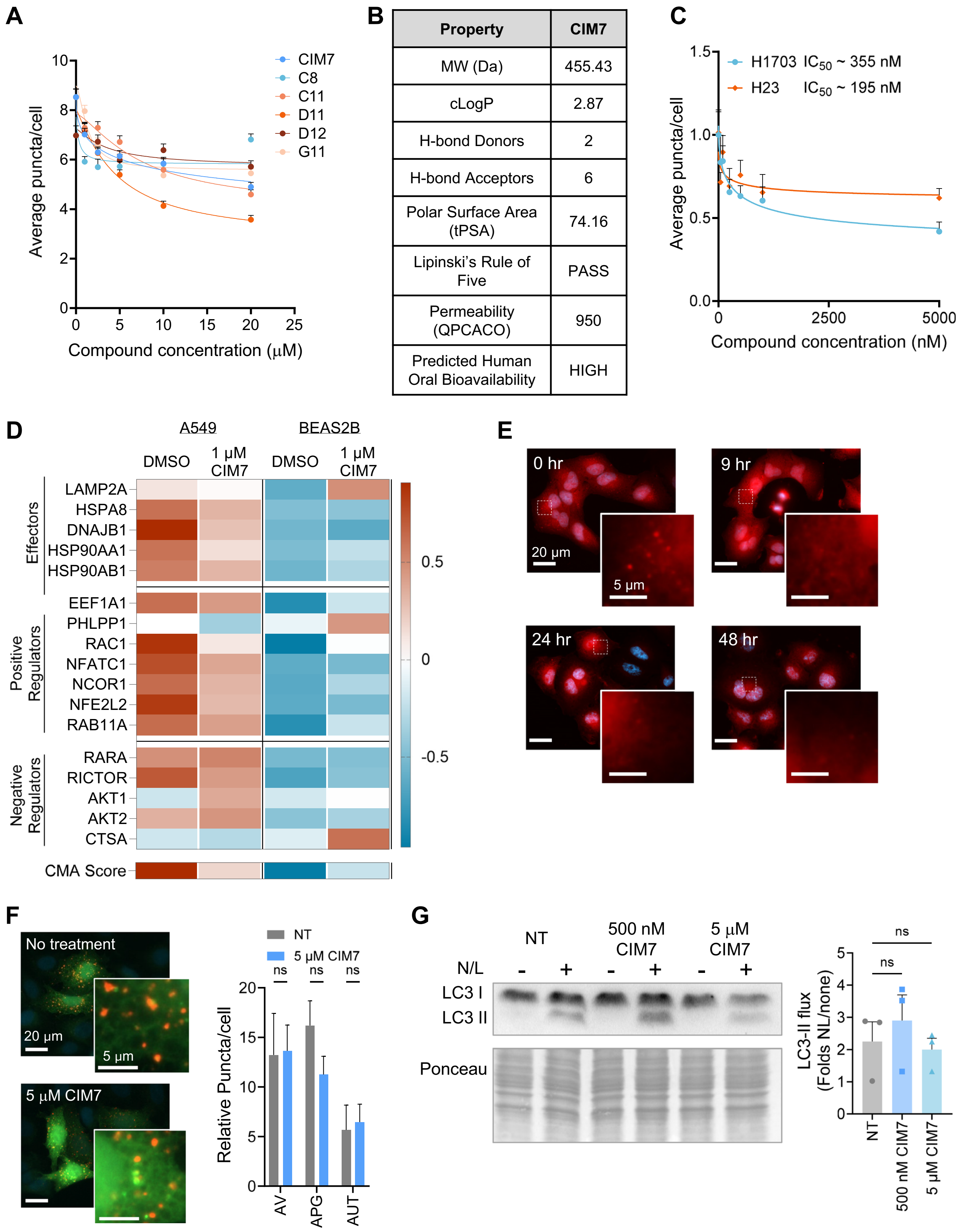

Property	CIM7
MW (Da)	455.43
cLogP	2.87
H-bond Donors	2
H-bond Acceptors	6
Polar Surface Area (tPSA)	74.16
Lipinski's Rule of Five	PASS
Permeability (QPCACO)	950
Predicted Human Oral Bioavailability	HIGH

Figure EV3. CIM7 selectively inhibits CMA in NSCLC cells.

(**A**) CMA activity in mouse fibroblasts (NIH-3T3) expressing KFERQ-PS-Dendra and treated with increasing doses of the indicated compounds in media lacking serum quantified as fluorescent puncta per cell. $n = 9$ fields. (**B**) Calculated chemical properties and predicted ADME properties of CIM7, from QikProp (Schrödinger, LLC) analysis. (**C**) CMA activity in H1703 and H23 expressing KFERQ-PS-Dendra treated with increasing concentrations of CIM7 based on quantification of fluorescent puncta per cell. $n > 40$ individual cells from 1–3 independent experiments. (**D**) Heatmap of the z-scores of the transcriptional differences of CMA-related genes (top) and calculated CMA score (bottom) in A549 and BEAS-2B cells 3 h after addition of 1 µM CIM7. $n = 4$ independent experiments. (**E**) Representative images of A549 cells expressing KFERQ-PS-Dendra treated with 5 µM CIM7 for the indicated times. Insets show higher magnification and nuclei are highlighted with DAPI. Quantification is in Fig. 2I. (**F**) Macroautophagy activity in A549 cells expressing mCherry-GFP-LC3 treated with DMSO (no treatment: NT) or 5 µM CIM7 for 24 h. Representative images (left) and quantification of number of the indicated autophagic compartments (right). AV autophagic vacuoles, APG autophagosomes, AUT autolysosomes. $n = 85$–87 cells from 3 independent experiments. (ns = not significant). (**G**) Immunoblot for LC3 in A549 cells treated with the indicated doses of CIM7 in the absence or presence of ammonia chloride and leupeptin (N/L). Representative immunoblot (left) and quantification of LC3-II flux (calculated by levels of LC3-II in the presence of N/L over levels of LC3-II in the absence of N/L) (right). Ponceau staining is shown as loading control. $n = 3$ independent experiments. (ns = not significant). Data information: All values are mean + SEM with individual data points to represent individual experiments. Nonlin fit (**A**, **C**), two-way ANOVA (**F**), or ordinary one-way ANOVA (**G**) followed by Bonferroni's multiple comparisons post-hoc test were used. Source data are available online for this figure.

 © The Author(s)

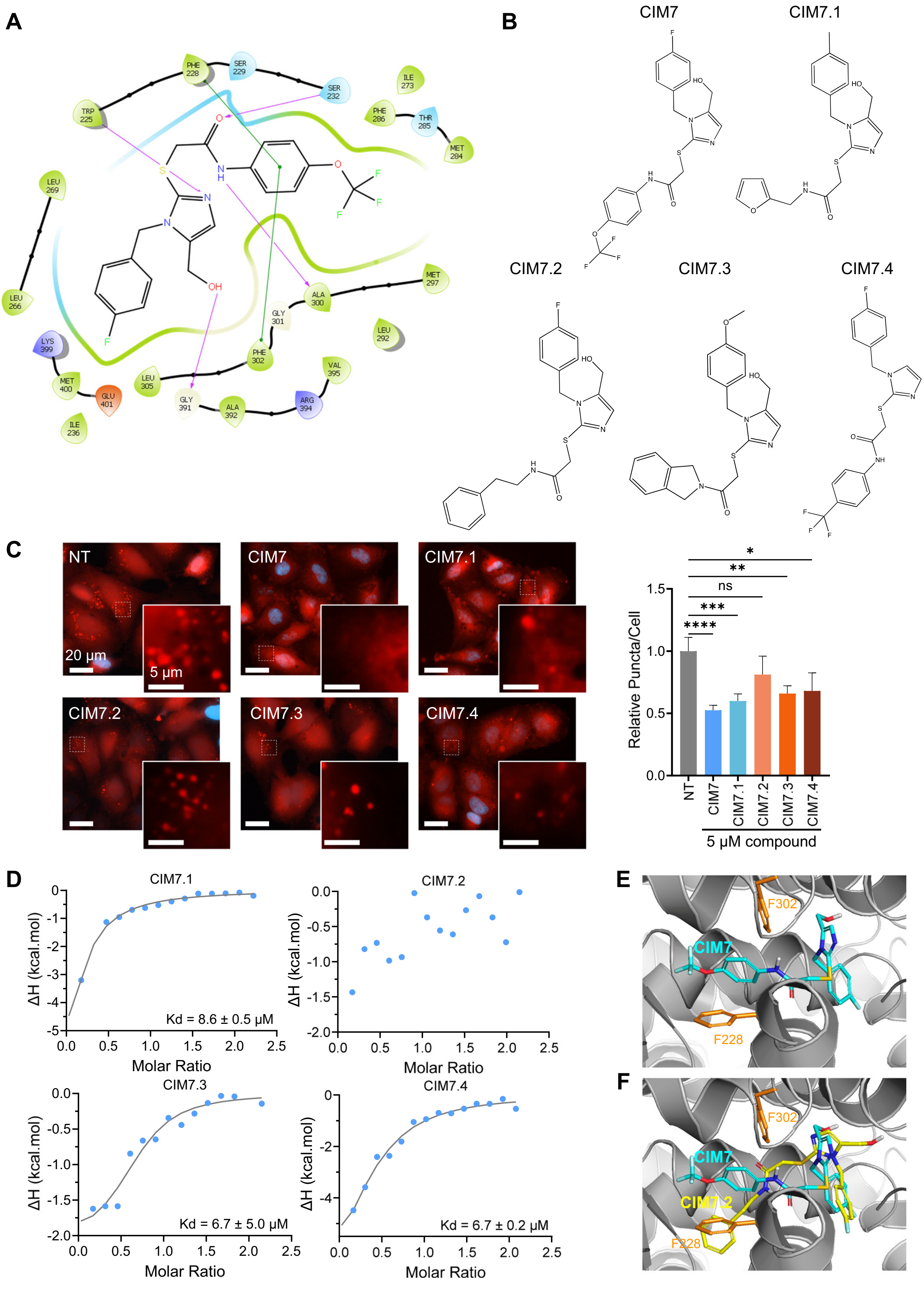
A
B
CIM7
CIM7.1
CIM7.2
CIM7.3
CIM7.4
C
NT
20 µm
5 µm
Relative Puncta/Cell
ns
5 µM compound
D
CIM7.1
Kd = 8.6 ± 0.5 µM
CIM7.2
CIM7.3
Kd = 6.7 ± 5.0 µM
CIM7.4
Kd = 6.7 ± 0.2 µM
ΔH (kcal.mol)
Molar Ratio
E
F
F302
F228

Figure EV4. Evaluation of CIM7 analogs.

(**A**) Predicted ligand interactions occurring between CIM7 and RARα based on 1000 ns molecular dynamic simulation. (**B**) Compound structures of CIM7 and four analogs. (**C**) CMA activity, relative to no treatment (NT), in A549 cells expressing KFERQ-PS-Dendra treated with 5 μM compound for 24 h. Representative images (left), with insets showing higher magnification. Nuclei are highlighted with DAPI. CMA activity quantified by number of fluorescent puncta/cell (right). $n = 63$–132 cells from 2–3 independent experiments. (****$P \leq 0.0001$, ***$P = 0.0004$, ns=not significant, **$P = 0.0036$, *$P = 0.0195$). (**D**) Representative isothermal titration calorimetry curves for the four CIM7 analogs binding to recombinant RARα. These experiments were repeated 2–3 times with consistent results. (**E**, **F**) CIM7 (cyan) binding pose generated after 1000 ns molecular dynamic simulation (**E**) or with the overlay of CIM7.2 (yellow) induced fit docking binding pose (**F**). Phe228 and Phe302 residues are highlighted in orange. Data information: Values are mean + SEM. Ordinary one-way ANOVA (**C**) was used. Source data are available online for this figure.

 © The Author(s)

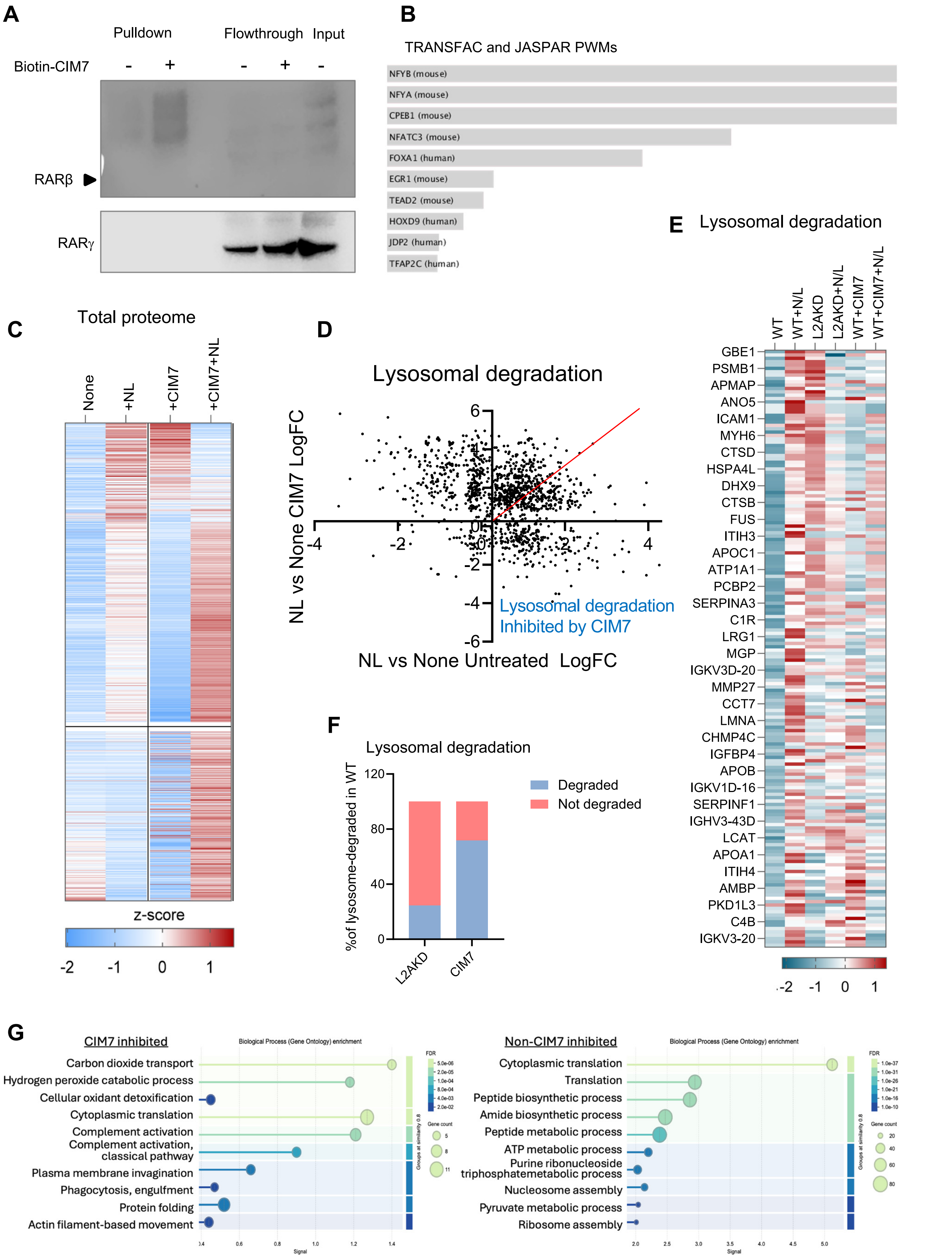
A
Pulldown
Flowthrough
Input
Biotin-CIM7
RARβ
RARγ
B
TRANSFAC and JASPAR PWMs
NFYB (mouse)
NFYA (mouse)
CPEB1 (mouse)
NFATC3 (mouse)
FOXA1 (human)
EGR1 (mouse)
TEAD2 (mouse)
HOXD9 (human)
JDP2 (human)
TFAP2C (human)
C
Total proteome
None
+NL
+CIM7
+CIM7+NL
z-score
D
Lysosomal degradation
NL vs None CIM7 LogFC
NL vs None Untreated LogFC
Lysosomal degradation
Inhibited by CIM7
E
Lysosomal degradation
WT
WT+N/L
L2AKD
L2AKD+N/L
WT+CIM7
WT+CIM7+N/L
GBE1
PSMB1
APMAP
ANO5
ICAM1
MYH6
CTSD
HSPA4L
DHX9
CTSB
FUS
ITIH3
APOC1
ATP1A1
PCBP2
SERPINA3
C1R
LRG1
MGP
IGKV3D-20
MMP27
CCT7
LMNA
CHMP4C
IGFBP4
APOB
IGKV1D-16
SERPINF1
IGHV3-43D
LCAT
APOA1
ITIH4
AMBP
PKD1L3
C4B
IGKV3-20
F
Lysosomal degradation
%of lysosome-degraded in WT
Degraded
Not degraded
L2AKD
CIM7
G
CIM7 inhibited
Biological Process (Gene Ontology) enrichment
Carbon dioxide transport
Hydrogen peroxide catabolic process
Cellular oxidant detoxification
Cytoplasmic translation
Complement activation
Complement activation, classical pathway
Plasma membrane invagination
Phagocytosis, engulfment
Protein folding
Actin filament-based movement
Signal
Non-CIM7 inhibited
Cytoplasmic translation
Translation
Peptide biosynthetic process
Amide biosynthetic process
Peptide metabolic process
ATP metabolic process
Purine ribonucleoside triphosphatemetabolic process
Nucleosome assembly
Pyruvate metabolic process
Ribosome assembly

Figure EV5. CIM7 selectively targets RARα and affects degradation of a subset of CMA substrates.

(**A**) Representative immunoblot for RARβ (top) and RARγ (bottom) of streptavidin pulldowns (left) and flowthrough (right) of A549 cellular lysate incubated without additions or with biotin-CIM7 (50 μM). This experiment was repeated twice with consistent results. (**B**) TRANSFAC and JASPAR PWMs obtained through analysis with Enrichr of genes significantly altered upon CIM7 treatment in the presence of AHT RARα in A549 cells. (**C**) Heat map as Z-score of the full proteome of A549 cells untreated (none) or treated with CIM7 in presence or not of lysosomal proteolysis inhibitors (N/L). Top quadrant is shown in Fig. 5A. (**D**) Log2 fold changes (logFC) in rates of lysosomal degradation in untreated against CIM7-treted A549 cells. (**E**) Heat map (of z-scores) of changes in the subset of proteins degraded in lysosomes in A549 cells untreated (WT), in presence of CIM7 or upon LAMP2A knockdown (L2AKD). (**F**) Percentage of lysosome-degraded proteins in A549 cells displaying inhibited lysosomal degradation in the same cells as in (**E**). (**G**) Gene Ontology enrichment of CMA substrates whose degradation is inhibited (left) or not (right) by CIM7 treatment in WT A549 cells, as predicted by STRING analysis. Bar coloring corresponds to false discovery rate (FDR). Source data are available online for this figure.

© The Author(s)

A

		Vehicle	25 mg/kg CIM7	Normal Range
WBC (K/mL)	Day 0	4.8 ± 0.5	3.7 ± 0.4	1.42 – 10.25
	Day 14	4.6 ± 0.3	4.9 ± 0.4	
	Day 28	5.0 ± 0.6	6.2 ± 0.7	
RBC (K/mL)	Day 0	10.1 ± 0.2	10.8 ± 0.2	6.82 – 10.53
	Day 14	10.0 ± 0.2	9.3 ± 0.2	
	Day 28	9.7 ± 0.2	9.5 ± 0.3	
Platelets (K/mL)	Day 0	1034 ± 95	958 ± 107	376 – 1796
	Day 14	964 ± 64	871 ± 89	
	Day 28	881 ± 30	836 ± 96	

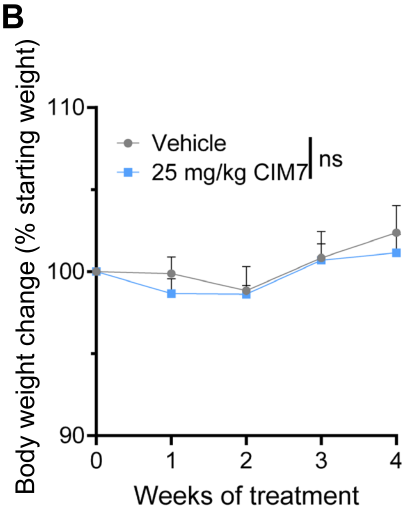

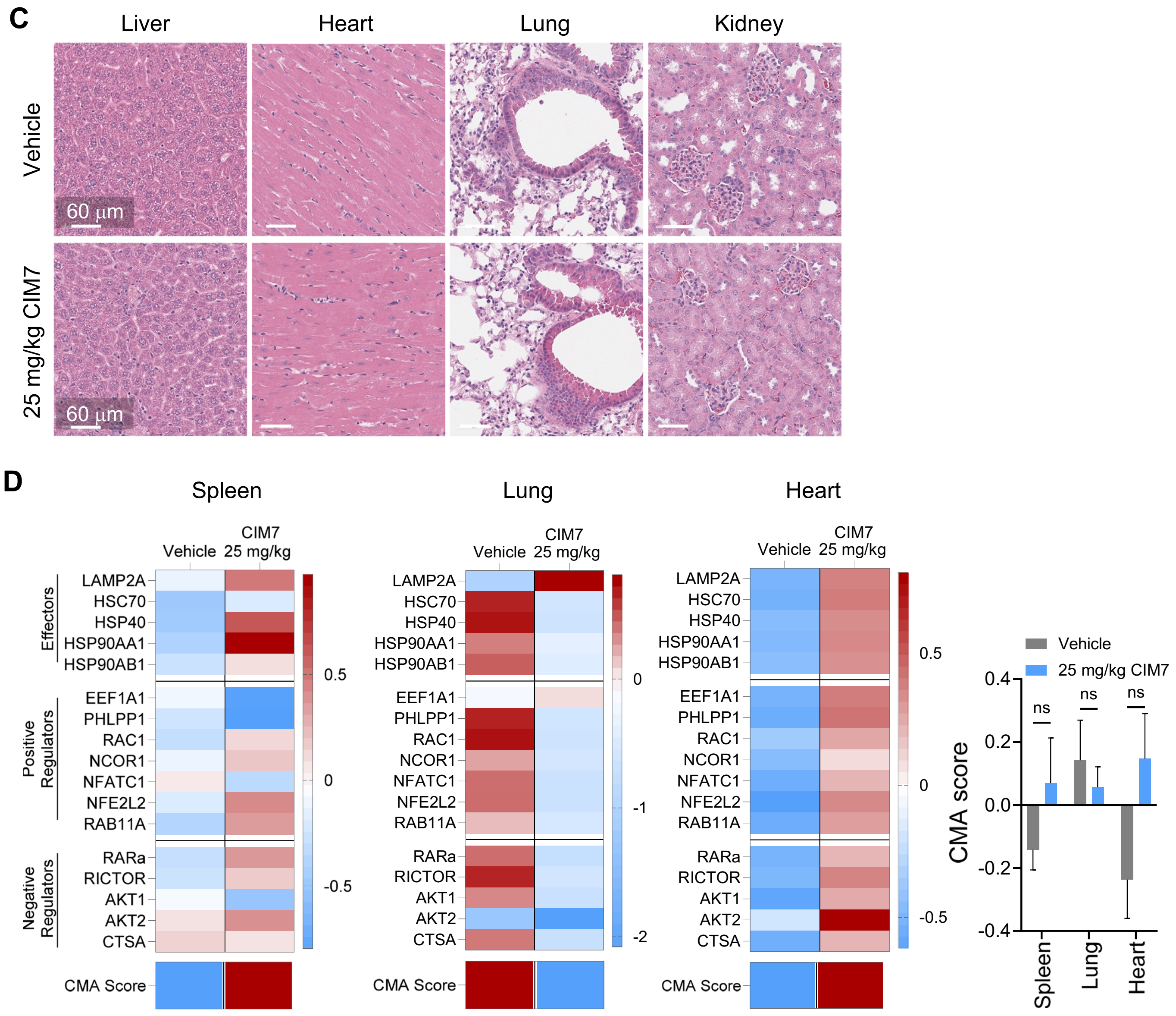

Figure EV6. CIM7 has no apparent toxicity in vivo.

(**A**) Complete blood cell counts of vehicle- or 25 mg/kg CIM7-treated mice at the indicated days of treatment. The normal range for each blood count is shown in the right column. $n = 9$–11 mice per treatment group. (**B**) Changes in weight, as a percentage of initial body weight, over time in vehicle- or 25 mg/kg CIM7-treated mice, measured weekly. $n = 9$–11 mice per treatment group. (ns = not significant). (**C**) H&E staining of liver, heart, lung, and kidney from representative vehicle- and 25 mg/kg CIM7-treated mice. (**D**) Heatmaps (left) and quantification (right) of CMA score changes occurring in the spleen, lung, and heart of vehicle- or 25 mg/kg CIM7-treated mice. $n = 9$–11 mice per treatment group. (ns = not significant). Data information: All values are mean + SEM. Ordinary two-way ANOVA followed by multiple comparisons post-hoc test (**B**, **D**) was performed. RBC red blood cell, WBC white blood cell. Source data are available online for this figure.

 © The Author(s)

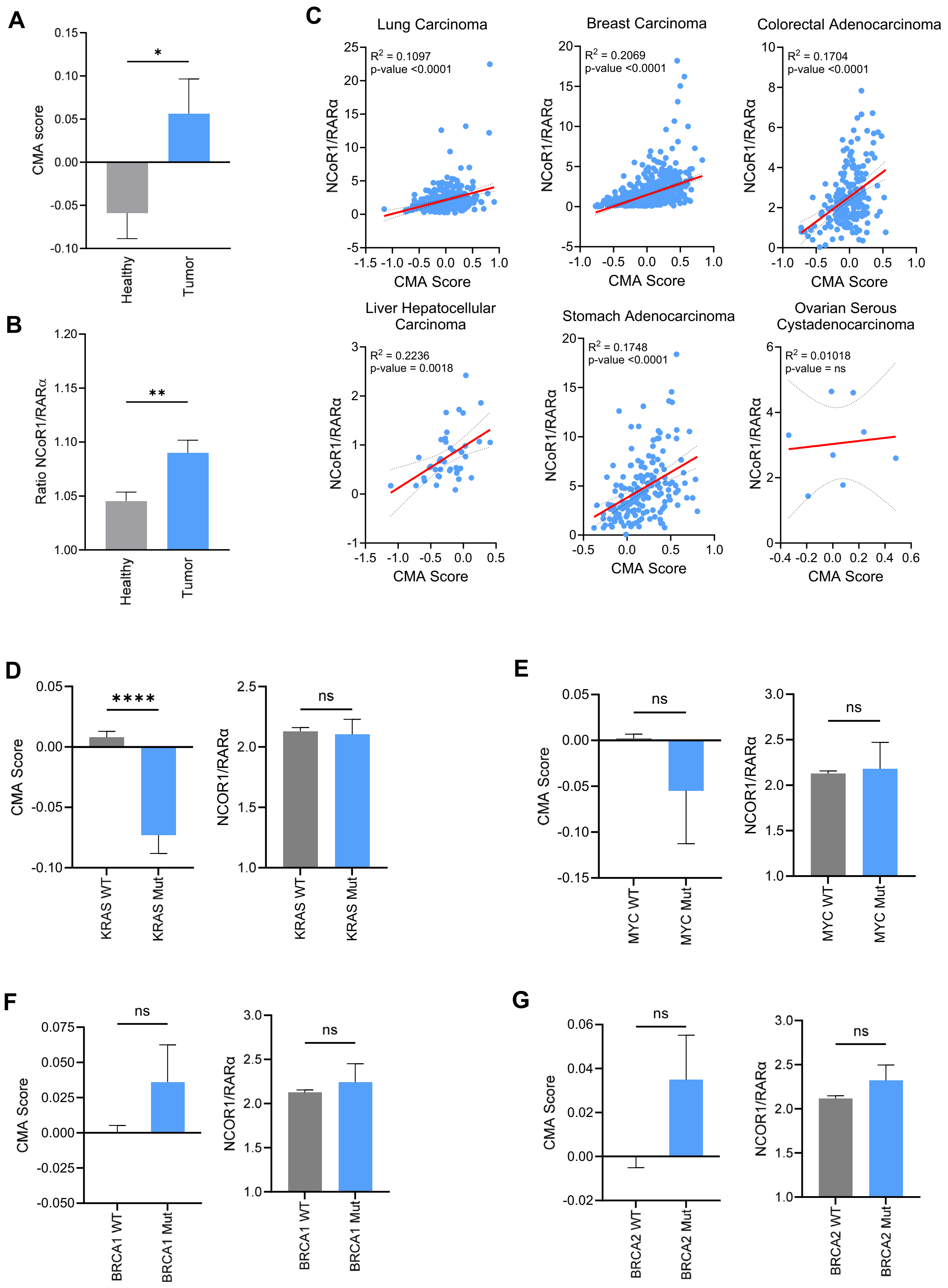
A
CMA score
Healthy
Tumor
*
B
Ratio NCoR1/RARα
Healthy
Tumor
**
C
Lung Carcinoma
R^2 = 0.1097
p-value <0.0001
Breast Carcinoma
R^2 = 0.2069
p-value <0.0001
Colorectal Adenocarcinoma
R^2 = 0.1704
p-value <0.0001
Liver Hepatocellular Carcinoma
R^2 = 0.2236
p-value = 0.0018
Stomach Adenocarcinoma
R^2 = 0.1748
p-value <0.0001
Ovarian Serous Cystadenocarcinoma
R^2 = 0.01018
p-value = ns
NCoR1/RARα
CMA Score
D
CMA Score
NCOR1/RARα
KRAS WT
KRAS Mut
****
ns
E
CMA Score
NCOR1/RARα
MYC WT
MYC Mut
ns
F
CMA Score
NCOR1/RARα
BRCA1 WT
BRCA1 Mut
ns
G
CMA Score
NCOR1/RARα
BRCA2 WT
BRCA2 Mut
ns

Figure EV7. Evaluation of CMA score in various cancer types.

(**A**, **B**) CMA score (**A**) and NCoR1/RARα ratio (**B**) calculated from RNA-seq of tumor and surrounding healthy lung tissue from NSCLC patients. Data from (Sanchez-Palencia et al, 2011). $n > 44$ individual patients. (**A**): $^{*}P = 0.0248$, (**B**): $^{**}P = 0.0029$). (**C**) Correlation of NCoR1/RARα ratio and CMA score in select cancers. Each individual data point represents an individual patient sample. Data from the TCGA Pan Cancer Altas (Liu et al, 2018). $n > 8$ individual tumors. (**D–G**) CMA score (left) and NCoR1/RARα ratio (right) calculated in tumors with wild-type (WT) or mutant (Mut) KRAS (**D**), MYC (**E**), BRCA1 (**F**), or BRCA2 (**G**). Data from the TCGA Pan Cancer Altas (Liu et al, 2018). $n > 38$ individual tumors. ($^{****}P < 0.0001$, ns = not significant). Data information: All bar values are mean + SEM. Unpaired two-tailed *t* test (**A**, **B**, **D–G**) and simple linear regression (**C**) were used. Source data are available online for this figure.

 © The Author(s)
